# Interventions to improve quantitative measures of parent satisfaction in neonatal care: a systematic review

**DOI:** 10.1136/bmjpo-2019-000613

**Published:** 2020-03-15

**Authors:** Susanna Sakonidou, Izabela Andrzejewska, James Webbe, Neena Modi, Derek Bell, Chris Gale

**Affiliations:** 1 Neonatal Medicine, School of Public Health, Faculty of Medicine, Imperial College London, London, UK; 2 NIHR CLAHRC for Northwest London, London, UK

**Keywords:** neonatology, outcomes research, patient perspective

## Abstract

**Objective:**

Interventions improving parent satisfaction can reduce parent stress, may improve parent-infant bonding and infant outcomes. Our objective was to systematically review neonatal interventions relating to parents of infants of all gestations where an outcome was parent satisfaction.

**Methods:**

We searched the databases MEDLINE, EMBASE, PsychINFO, Cochrane Central Register of Controlled Trials, CINAHL, HMIC, Maternity and Infant Care between 1 January 1946 and 1 October 2017. Inclusion criteria were randomised controlled trials (RCT), cohort studies and other non-randomised studies if participants were parents of infants receiving neonatal care, interventions were implemented in neonatal units (of any care level) and ≥1 quantitative outcome of parent satisfaction was measured. Included studies were limited to the English language only. We extracted study characteristics, interventions, outcomes and parent involvement in intervention design. Included studies were not sufficiently homogenous to enable quantitative synthesis. We assessed quality with the Cochrane Collaboration risk of bias tool (randomised) and the ROBINS-I tool (Risk Of Bias In Non-randomised Studies - of Interventions) (non-randomised studies).

**Results:**

We identified 32 studies with satisfaction measures from over 2800 parents and grouped interventions into 5 themes. Most studies were non-randomised involving preterm infants. Parent satisfaction was measured by 334 different questions in 29 questionnaires (only 6/29 fully validated). 18/32 studies reported higher parent satisfaction in the intervention group. The intervention theme with most studies reporting higher satisfaction was parent involvement (10/14). Five (5/32) studies reported involving parents in intervention design. All studies had high risk of bias.

**Conclusions:**

Many interventions, commonly relating to parent involvement, are reported to improve parent satisfaction. Inconsistency in satisfaction measurements and high risk of bias makes this low-quality evidence. Standardised, validated parent satisfaction measures are needed, as well as higher quality trials of parent experience involving parents in intervention design.

**PROSPERO registration number:**

CRD42017072388.

**What is known about the subject?**

- Neonatal care significantly affects parents’ mental health; parent satisfaction is increasingly being used as a parent experience measure.
- Parent satisfaction is inversely related to parent stress; interventions improving parent satisfaction have the potential to reduce parent stress, improve parent-infant bonding and infant outcomes.
- Use of interventions measuring parent satisfaction as an outcome in neonatal units is increasing, although few are formally evaluated and wider uptake is limited; it is not known the degree to which parents are involved in intervention design.

**What this study adds?**

- There is inconsistency in how parent satisfaction in neonatal care is defined and measured, and the majority of studies do not include parents in intervention design.
- There is low-quality evidence that interventions relating to parent involvement may improve parent satisfaction with neonatal care.
- Standardised, validated measures of parent satisfaction and higher quality trials, involving parents in intervention design, are needed.

## Introduction

One in 10 newborn babies in high-income countries require neonatal care.^1^ This is stressful for parents, who often develop anxiety, depression and post-traumatic stress disorder symptoms.^2–4^ Parental stress interferes with parent-child bonding^5^ and there is a well-established link between maternal mental health and infant development.^6^ Parent satisfaction, defined as ‘*the perception of parents’ needs and expectations being met*’ is inversely related to parental stress.^7^ As such, it is increasingly being used as a parent experience measure and neonatal service quality indicator. Interventions aimed at improving parent satisfaction have the potential to reduce parent stress, improve parent-infant bonding^8^ and infant outcomes.^9^

A range of parent-centred interventions, such as including parents on ward rounds, have recently become widespread in neonatal practice. Many are implemented on a small scale, without evaluating their impact on parent experience, making long-term integration into neonatal services challenging, while many others are using parent questionnaires. ‘Parent satisfaction’ as an outcome is gaining momentum, as neonatal trusts attempt to match more ‘business-like models’ where effectiveness of interventions (and evidence for change) is measured by quantitative outcomes. Moreover, where parent experience is measured as ‘parent satisfaction’, some studies include it as a primary outcome, whereas others use it as a secondary indicator to explore the parent point of view.

Furthermore, there are multiple experience measures available in addition to parent satisfaction, including parent stress, anxiety and depression scales; both quantitative and qualitative. Finally, it is not known the degree to which parents are involved in the design of such interventions. There have been no previous systematic evaluations focused on interventions measuring parent satisfaction with neonatal care as an outcome.

The aim of this review is to identify and describe neonatal interventions relating to parents of infants of all gestations where an outcome was parent satisfaction. For the reasons outlined above, we have only included studies that reported ≥1 quantitative measure of parent satisfaction. We aim to report each intervention’s effect on parent satisfaction, as well as parent input in intervention design.

## Methods

We reported this study using Preferred Reporting Items for Systematic Reviews and Meta-Analyses guidelines.^10 11^ We searched MEDLINE, EMBASE, PsychINFO, Cochrane Central Register of Controlled Trials, CINAHL, HMIC, Maternity and Infant Care (online supplementary file 1) for English papers published between 1 January 1946 and 1 October 2017, with update searches on 1 September 2018.

10.1136/bmjpo-2019-000613.supp1 Supplementary data

Inclusion criteria were: randomised controlled trials (RCT) and non-randomised studies (non-RCT) if participants were parents of infants receiving neonatal care, interventions were implemented in neonatal units and ≥1 quantitative outcome of parent satisfaction was measured. We have restricted our review to studies where ≥1 quantitative outcome of parent satisfaction was measured, in order to enable comparison of interventions, which has previously not been possible in any published review. Including studies with all available measures of parent experience (in addition to parent satisfaction), as well as those only qualitatively evaluated, would make any comparison very difficult. By using these preregistered search criteria, we also ensured we would capture studies measuring parent satisfaction both as primary and as secondary outcomes. We included studies from all neonatal care level units and all healthcare settings, without excluding studies in low-income or middle-income settings. This was because definitions of neonatal care levels differ between different countries and healthcare settings, making them not easily comparable. Moreover, different levels of care are found within the same hospital settings. We excluded systematic reviews, entirely qualitative studies, grey literature (eg, conference abstracts), studies only reporting protocols or abstracts and full reports not in English.

Two authors (SS, IA) independently double-screened titles and abstracts, reviewed full texts for eligibility and resolved any discrepancies with a third reviewer (JW). We extracted data using a pilot-tested, standardised data extraction form including study characteristics, interventions, outcomes and parent input into interventions’ design. We assessed methodological quality with the Cochrane Collaboration risk of bias tool^12^ for RCT and the ROBINS-I tool (Risk Of Bias In Non-randomised Studies - of Interventions)^13^ for non-RCT.

We presented individual study aggregate data in a narrative synthesis, grouped studies into themes using a Grounded Theory Approach^14^ and planned meta-analysis where data were appropriate for quantitative synthesis.

### Patient and public involvement

This review was conceived in response to the clinical need identified by parents with neonatal care experience; a partnership including families with experience of preterm birth identified ‘what emotional and practical support improves attachment and bonding, and does the provision of such support improve outcomes for premature babies and their families?’ as a top 10 research priority.^15^ Additionally, this review was conceived as part of planning a wider project to pilot a neonatal intervention, with parents’ full input.^16^ Patients were not directly involved in the design, conduct, reporting or dissemination plans of our research.

## Results

**Figure 1:**
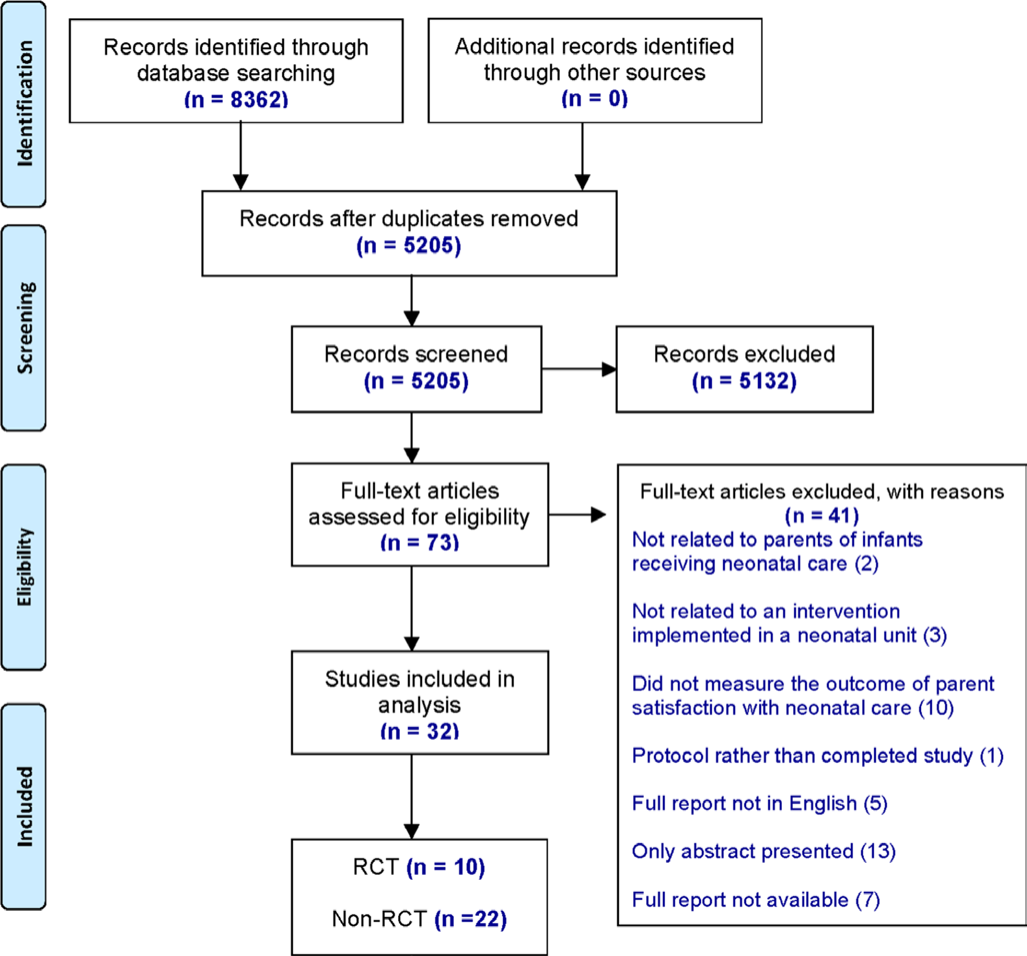
Preferred Reporting Items for Systematic Reviews and Meta-Analyses flow diagram of selected studies.

We identified 8362 studies for screening and assessed 73 full-text articles for eligibility (figure 1). A total of 32 studies describing interventions that measured parent satisfaction in neonatal care as an outcome met the inclusion criteria, reporting data from over 2866 parents, 1 study did not report number of parents. Our analysis included 10 RCT and 22 non-RCT: 3 cohort trials, 18 unspecified designs and 1 implementation project (tables 1–3). We further classified the unspecified non-RCT into two types, depending on how they defined their control groups and how they evaluated parent satisfaction (table 3).

**Table 1:** Included randomised controlled trials (RCTs)

RCT by publication year										
Author (date), country	Parents’ gender/ sample size	Infants’ gestation age in weeks/NICU level	Study design	Intervention	Outcome measures	Timing of measurement	Method of measurement	Results	Parent co-design?	Improved parent satisfaction?
1. Northrup *et al* (2016), USA	Mothers and fathers /116	<28/level III	RCT	Intervention: free parking. Parents received seven parking vouchers at a time (value: US$10 each) and continued to receive vouchers until infant discharge. Each voucher allowed free entry and exit for 24 hours. Control: parents received the standard care and did not receive vouchers.	*Parent satisfaction with NICU care.*	After babies were discharged (once) During the first high-risk infant clinic visit after discharge. No preintervention parent satisfaction data available for comparison.	*Satisfaction questionnaire* Validation: no content validity or reliability testing reported. Eleven questions: Seven items were summed (score 7–35) to measure ‘support’ (eg, information sharing). Three items measured ‘emotional connection’ to the infant (score 3–15). One item assessed ‘family involvement in infant care’ (responses: not enough, just right, too much). Greater scores indicated higher perceived support, connection and satisfaction.	*The groups did not differ significantly with respect to satisfaction*. **Intervention Control P value** NICU support **Mean** (**SD**) 30 (2.7) 28.7 (3.7) 0.07 Emotional connection 12.3 (1.7) 12.3 (1.7) 0.96 Family involvement ‘Just right’ 81.4%–85% 0.07	**No**	**2**
2. Abdel-Latif *et al* (2015), Australia	Mothers and fathers /63	25–42/level III	Cross-over RCT	Intervention: parental Presence at Clinical Bedside Rounds (PPCBR). Parents attended bedside clinical rounds. Parents had opportunity to ask questions about their baby’s condition and management. Control: parents received the standard care with no parental presence at bedside clinical rounds.	*Parent satisfaction assessed by questions of three domains:* 1. Knowledge and understanding. 2. Communication and collaboration. 3. Privacy and confidentiality.	During babies’ admission (once): At the end of each study arm, separated by a washout period. No preintervention parent satisfaction data available for comparison.	*Satisfaction questionnaire* The authors stated ‘the research team designed the questionnaire’. Validation: no content validity or reliability testing reported. Number and format of questions: not stated.	*PPCBR had significantly higher adjusted mean (95% CI) scores for some questions from domains 1 and 2*. Domain 3 was comparable between the two study groups. **Intervention Control p-value** Domain 1 question: *“I have received adequate information about my baby’s condition and management”* **Mean** 4.321 3.947 0.03 Domain 2 questions: *“In the last week I have been able to communicate effectively with my baby’s healthcare team”* **Mean** 4.407 4.250 0.05 *“In the last week I have collaborated with my baby’s healthcare team in the planning of care for my baby”* **Mean** 3.843 3.426 0.02 *“In the last week I have been able to ask the healthcare team questions about my baby’s care”* **Mean** 4.642 4.259 0.004	**No**	**1**
3. Bastani *et al* (2015), Iran	Mothers /100	30–37 Mean (SD) Control: 33.90 (2.33) Intervention: 34 (1.9) /level not stated	RCT (block randomisation)	Intervention: family centred care (FCC). Mothers allowed access to their baby at any time, participated in the care process and were provided with information about neonatal care. Control: mothers received the standard care where they were only allowed to be present at the time of the infant’s entry to the NICU, and were only routinely informed.	*Maternal satisfaction* relating to three themes: Parental presence. Participation in neonatal care. Information about neonatal care.	During babies’ admission (twice): 24 hours after admission. At the time of discharge.	*Satisfaction questionnaire* (validated) A modified satisfaction questionnaire was used, based on a parental satisfaction instrument developed for measuring satisfaction in paediatric intensive care units. 18 questions Graded 0 (very dissatisfied) to 4 (very satisfied). The overall satisfaction rate was classified based on the mean scores (score <50%, between 75%–50% and >75%).	*In the FCC group, preintervention and postintervention difference in maternal satisfaction was statistically significant p<0.001.* **Intervention Control P value** **Mean (SD**) **At 24** hours 22.36 (8.90) 22.06 (9.77) 0.87 **At discharge** 59.28 (6.86) 30.18 (14.09)<0.01	**Unclear** Mothers determined the reliability of the satisfaction tool and approved the educational pamphlet. Authors did not report if mothers had direct input in the intervention design.	**1**
4. Clarke-Pounder *et al* (2015), USA	Mothers and fathers /19 families	23–39/level III	RCT	Intervention: sharing information obtained from parent interviews with the primary NICU provider. Parents interviewed using the *NICU- adapted Decision-Making Tool (N-DMT*). Information obtained was placed in the electronic medical record (EMR) and shared with the primary neonatal provider via email. Daily rounds on all infants were audio-recorded for 3 days after enrolment to see if information from the N-DMT was incorporated into daily care planning. Control: the content of a recent social work note was communicated with the primary provider via email, creating an attentional control group.	*Parent satisfaction with care.*	During babies’ admission (once): 2 weeks after study entry. No preintervention parent satisfaction data available for comparison.	*Satisfaction questionnaire* An *N-DMT*-specific questionnaire was used. Validation: partially reported. Authors stated reliability testing took place; no information on content validity provided. Eight questions: for example,‘‘My baby’s doctors considered my goals and hopes for my baby during decision-making’’. Likert scale (1 strongly agree–4 strongly disagree). Total N-DMT score range 8–32.	*There was no significant difference in satisfaction with care as measured by the N-DMT scale between the control group and intervention groups in a univariable model or multiple variable model controlling for gestational age*. **Intervention Control** **Median** (range) 26 (15–28) 28.8 (19–32) *No P value reported.* There was, however, a pattern of decreased satisfaction with care among the intervention group compared with the control group across the N-DMT-specific survey questions, although the differences were not statistically significant.	**Yes** Information obtained from parents using the N-DMT was placed in the EMR and shared with the primary NICU provider via email (forming the intervention).	**2**
5. Holditch-Davis *et al* (2013), USA	Mothers /208	Preterm infants Mean (SD) Overall group 27.2 (3.0) /4 centres, levels II–III	RCT three groups (two intervention and one control) Postintervention testing only	Interventions: 1. Mothers were taught how to massage infants with auditory, tactile, visual and vestibular stimulation. 2. Kangaroo care. Control: attention control group. Mothers spent a similar amount of time with the study nurse discussing the equipment needed for preterm infant care at home. Study nurses provided education and support for all three groups. Mothers were not prevented from engaging in interventions of the other groups but did not receive formal education from the study nurse on the other interventions.	*Parent (mother) satisfaction with the intervention.* *Satisfaction with the helpfulness of the study nurse.* *Whether the mother would recommend the study to others and the degree of change in the mother as a person and as a mother as a result of being in the study.*	During admission period and postdischarge: At the time of discharge. At 2 months corrected age. No preintervention parent satisfaction data available for comparison.	*Satisfaction questionnaire* The questionnaire was designed by the study team. Validation: partially reported. Authors stated reliability testing took place; no information on content validity provided. 26 questions: relating to three dimensions of satisfaction: efficacy, caring and technical quality. Likert (1 least satisfied–5 most satisfied).	*No significant differences occurred between the groups*. Mothers in all three groups were satisfied with the intervention (mean scores of 3.3 or higher on a 5-point scale) and the helpfulness of the nurse (mean scores of 4.6 or higher on a 5-point scale).	**No**	**2**
6. Franck *et al* (2011), UK	Mothers and fathers /169	Mean (SD) Control: 31.94 (5.17) Interv: 29.40 (3.17) /4 centres, level III	Cluster RCT	Intervention: increasing parental involvement in infant pain management in the NICU. Parents received a booklet providing evidence-based information about pain and comforting infants in the NICU setting. Parents received two visits from a research nurse showing them how to apply the comforting techniques described in the booklet. Control: as part of usual care, parents in both the intervention and control groups received a detailed booklet with generic information about NICU care. Parents in the control group also received two visits from a research nurse listening to what parents had to say about their NICU experience (attention placebo).	At baseline: *Parent satisfaction with NICU care.* One week after the intervention: Satisfaction with information about pain control. Satisfied nurses make infant comfortable. Satisfied pain medicines help infant.	During babies’ admission (twice): At baseline (within 3–7 days of admission). 1 week after the intervention.	*Individual questions* Validation: no content validity or reliability testing reported. 1. At baseline: parent satisfaction was measured by one question: *‘satisfaction with NICU care’* (1 very satisfied–6 very unsatisfied) as part of the baseline parent characteristics questionnaire. 2. One week after the intervention: Three questions using the word ‘satisfied’ were selected from the validated *Parent Attitudes About Infant Nociception* survey (Likert scale 1 very satisfied–6 very unsatisfied).	*At baseline: there was no significant difference in satisfaction between intervention and control group.* **Intervention Control** **Mean** 1.45 (0.71) 1.51 (0.76) (**SD)** **P value** missing *1 week after the intervention: intervention parents were more satisfied with the information about pain control received than control parents*. **Intervention Control** **Mean** 2.10 (0.97) 3.28 (1.27) (**SD)** **P value**<0.001	**Yes** The booklet was reviewed by 12 parents of infants who had been cared for in NICUs in the UK.	**1**
7. Livingston *et al* (2009), USA	Mothers /12	Mean (SD) Control: 33.4 (6.4) Intervention: 38.5 (3.1) /level III	RCT	Intervention: touch and massage. Mothers attended a 1 hour massage class taught by a nurse certified infant massage instructor (CIMI) and were asked to participate in at least three bedside massage instruction sessions taught within the next week. Infants received massage for seven consecutive days, from the mother or a CIMI. The touch procedure lasted 20 min. Control: infants received all usual hospital services including medical care, physical and occupational therapy services and developmentally supportive nursing care.	*Caregiver (mother) satisfaction with their infant’s care.* *Caregiver satisfaction with the neonatal unit and the massage therapist*.	During babies’ admission (three times): At baseline On completing the 7 day massage programme. 1 month following intervention.	*Satisfaction questionnaire* Two questionnaires were developed by the research team. Validation: no content validity or reliability testing reported. First questionnaire (at baseline): a brief self-report questionnaire about caregiver satisfaction with their infant’s care until that moment. No further details reported. Second questionnaire (on completing the 7-day massage programme and 1 month following intervention): a 10 min satisfaction questionnaire relating to infant’s response and caregiver satisfaction with the neonatal unit and the massage therapist. Number of questions: not stated. Likert scale (1 very dissatisfied–4 very satisfied). Sample statements: *“How satisfied do you feel giving massage to your infant?”; “I feel that massage improved my infant’s hospital stay”.*	*It is unclear in the report if specific between-group comparisons and statistical analysis were conducted*. At baseline and day 7: All caregivers were highly satisfied with the medical treatment their infant received. At day 7 and 1 month follow-up: All caregivers participating in the massage group reported high levels of satisfaction regarding their relationship with their infant and the massage programme’s impact on that relationship. Slight improvements in satisfaction regarding time the caregiver spent with the infant and involvement in the infant’s care were observed between day 7 and the 1 month follow-up (no further information reported).	**No**	**3**
8. Koh *et al* (2007), Australia	Mothers /200	Not stated/not stated	RCT	Intervention: provision of taped conversations with neonatologists to mothers. The initial conversation and subsequent conversations of significance with a neonatologist were taped and analysed (for both groups). Mothers received a tape of each conversation and a tape recorder. Control: usual care. Mothers were not given the tape or recorder.	*Satisfaction with conversations held with the neonatologist.* *Satisfaction with the tape.*	During admission period and postdischarge: At 10 days. At 4 months. At 12 months. No preintervention parent satisfaction data available for comparison.	*Individual questions and a satisfaction scale* Validation: no content validity or reliability testing reported. Number of questions: not stated. Likert scale (1–5 most satisfied) Questions related to: satisfaction with amount and quality of information presented, doctors’ communication skills, patient’s participation in the conversation. A satisfaction scale was used to assess: satisfaction with the tape.	No differences were found between the two groups in satisfaction with conversations. *Mothers of babies with a poor outcome in the tape group were, however, significantly more satisfied with the conversations*: **Intervention Control** **Mean** (**95% CI**) 115 (104–123.2) 100.5 (94.1–109.4) **P value** 0.0051 Most (71%–92%) of the mothers given the tapes stated that they helped their understanding, reminded them of what had been said, and helped their family to understand and recall information.	**No**	**1**
9. Mitchell-DiCenso *et al* (1996), Canada	Mothers and fathers/ 482	Mean (SD) Intervention: 35.1 (4.5) Control: 35 (4.3) /level III	RCT	Intervention: clinical nurse specialist/neonatal practitioner team (CNS/NP) care. Infants of intervention parents were assigned to be cared for by the CNS/NP team during the day and by paediatric residents during the night. Control: paediatric residents cared for infants of control parents around the clock. Neonatologists supervised both teams.	*Parent satisfaction with care.*	During admission period and postdischarge (twice): On fifth day after admission (full survey). After discharge over the phone (only questions related to satisfaction with discharge process). No preintervention parent satisfaction data available for comparison.	*Satisfaction questionnaire (validated*) The study team developed and used the validated *NIPS* questionnaire. Number of questions: not stated. NIPS score range (27–189); higher scores indicating greater satisfaction with care.	*No statistically significant difference between groups*. **Intervention Control P value** **NIPS** 140 139 0.67 **Mean** **Difference in means** 1.0, 95% CI (−3.6 to 5.6)	**No**	**2**
10. Broyles *et al* (1992), USA	Mothers /25	Mean (SD) Control: 34 (4) Intervention: 33.4 (4) /level III	RCT	Intervention: Detailed consent. Mothers were given information about mechanical ventilation. Detailed risk/benefit disclosure was provided both verbally and in writing. Control: mothers were given a brief verbal description about mechanical ventilation supplemented with detailed verbal and written disclosure if desired by them (flexible consent).	*Maternal satisfaction with the information provided about mechanical ventilation.*	During babies’ admission (once): 24–48 hours after the intervention No preintervention parent satisfaction data available for comparison.	*An interview* evaluating maternal satisfaction with the information provided about mechanical ventilation. Validation: a psychiatrist with a special interest in interviewing techniques was consulted in designing and standardising this assessment. A research nurse conducted the interview, ‘checking’ each mother against one option regarding: Amount of information: Right amount–too much–too little. Information made coping: more difficult–easier–no effect–uncertain.	This study is measuring and comparing satisfaction with two different interventions (detailed vs flexible consent process), neither of which formally represent the usual routine care for all babies (no control). *Small numbers. No data indicating statistical analysis conducted or evidence of statistically significant results*. **Detailed Flexible** **Right** 75% mothers 100% **amount of information** **Too** 25% mothers **little information** **Made** 67% mothers 69% **coping easier**	**No**	**3**

NICU, neonatal intensive care unit; NIPS, Neonatal Index of Parent Satisfaction.

**Table 2:** Included prospective cohort studies

Prospective cohort studies by publication year										
Author (date), country	Parents’ gender/sample size	Infants’ gestation age in weeks/NICU level	Study design	Intervention	Outcome measures	Timing of measurement	Method of measurement	Results	Parent co-design?	Improved parent satisfaction?
1. De Bernardo *et al* (2017), Italy	Mothers and fathers/96	Mean (SD) Control: 34.2 (5.25) Intervention: 32.7 (5.25) /level III	Non-randomised, prospective cohort pilot study *Unit level effect*: Two different time periods	Intervention: FCC. Parents had access to NICU for 8 hours/day. The NICU was widened and paediatric nurses taught parents procedures/practices for 10 days. Parents could observe clinical bedside rounds, hold meetings with the physicians, use the rooms and kitchen. Control: parents were permitted to visit their baby in NICU for 1 hour a day.	*Parent satisfaction relating to three specific domains*: Knowledge and Understanding. Communication and collaboration. Privacy and confidentiality.	During babies’ admission (once) At discharge (pre-FCC cohort and post-FCC cohort). No preintervention parent satisfaction data available for comparison (different parent groups preintervention and postintervention).	*Satisfaction questionnaire*. Validation: the authors state the survey ‘*was designed and validated by Abdel-Latif et al’*. No content validity or reliability testing reported in the original paper. Nine questions three questions: Related to adequate and timely information about the baby’s condition. three questions: Related to communication and collaboration with the healthcare team. Three questions: Related to respect of patient privacy. Likert (1 strongly disagree–5 strongly agree).	*7/9 individual statements in the parent satisfaction questionnaire scored higher in the FCC compared with the NFCC (statistically significant difference)*. Example statement: *“I have received adequate information about my baby’s condition and management”.* **Intervention Control** **Median** 5 (3.45–5) 4 (3–5) **P value**<0.05	**No**	**1**
2. Petteys *et al* (2015), USA	Not stated/10 parents included in sample analysis	24–36+/level III	A prospective cohort design. A feasibility study. *Group level effect:* Intervention/ control groups Postintervention testing only	Intervention: PC. PC nurses provided important continuity of care for NICU infants clinically requiring PC and at least weekly verbal support of parents. The PC service also coordinated family conferences, provided or requested orders to improve infant symptom management and comfort and addressed parental coping and self-care. Control: usual clinical care for infants not requiring PC.	*Overall satisfaction with care received.*	During babies’ admission (once) At discharge (or study closure for infants who remained hospitalised). No preintervention parent satisfaction data available for comparison.	*Satisfaction questionnaire* A researcher-created questionnaire based on extensive current literature review. Validation: Partially reported. Authors stated content validity testing took place; no information on reliability testing provided. One question Likert (1 extremely dissatisfied–4 to extremely satisfied). Optional free text (description of specific experiences impacting satisfaction with care).	*Parent satisfaction response numbers were small (n=10), thus statistical comparison of parental satisfaction between cohorts was not possible*. However, 100% of responding PC parents (n=2) reported being ‘extremely satisfied’ with care, whereas only 50% of responding usual care parents (n=4) reported extreme satisfaction.	**No**	**3**
3. Stevens *et al* (2011), USA	Mothers/147. For the OPBY NICU, 58 surveys were returned. For the SFR NICU, 89 were returned	Mean (SD) Control: 35 (4) Intervention: 34 (3) /level not stated	Cohort trial. This research was part of a large prospective evaluation. *Unit level effect:* Two different time periods	Intervention: SFR NICU for neonatal care. Parents could visit their baby, room-in, do kangaroo care and breast feed at any time, in individual rooms (containing bed, desk, closet, telephone, chair, refrigerator for breast-milk storage). Control: OPBY NICU. The traditional OPBY NICU was typical of facilities built before 1980. All neonates, family members, staff, monitors and equipment were visible for all neonates in each room. Portable partitions were placed around the incubator for breast feeding and kangaroo care.	*Parent satisfaction with different elements of NICU*: Delivery. Environment. Nurses. Physicians. Discharge. Personal. Overall assessment.	After babies were discharged (once) Mailed within 60 days of discharge of parents’ infants from the NICU. No preintervention parent satisfaction data available for comparison (different parent groups preintervention and postintervention).	*Satisfaction questionnaire* A questionnaire from Press Ganey Associates was used. Also included were three questions added by the investigators. Validation: Partially reported. The original questionnaire was validated questionnaire but no content validity or reliability testing was reported regarding the three questions added by the study team. Forty-two questions in total (seven categories): delivery, environment, nurses, physicians, discharge, personal, overall assessment. Likert (1 very poor–5 very good).	*Statistically significant improvement was found for the survey categories of Environment, Overall and the Total survey*. Estimated numbers from report’s figures as numbers not provided): **Median SFR OPBY P value** Environment 4.7 3.7<0.001 Overall 5 4.8 0.018 Total 4.7 4.5 0.045 16 items composite score for FCC: 4.4 4.0 0.017	**Yes** Former NICU parents were involved in all phases of planning for the new SFR NICU.	**1**

FCC, family centred care; NICU, neonatal intensive care unit; OPBY, Open-bay; PC, palliative care; SFR, single-family room.

**Table 3:** Included ‘other’ non-randomised controlled trials (non-RCT)

‘Other’ non-RCT by publication year										
Author (date), country	Parents’ gender/sample Size	Infants’ gestation age in weeks/NICU level	Study design	Intervention	Outcome measures	Timing of measurement	Method of measurement	Results	Parent co-design?	Improved parent satisfaction?
1. Kadivar *et al* (2017), Iran	Mothers/68	≤30–36 /level not stated	Non-randomised, convenience sampling. *Group level effect:* Intervention/control groups. Preintervention and postintervention testing.	Intervention: **internet-based education**. Mothers used an educational website set up by the research team (files and clips). Mothers could visit the website from 17:00 to 18:00 for 10 days. They were also allowed to use the website outside of the above hours and to report the duration of using the website to the researcher. Mothers had to use the website at least 3 times during 10 days, each time for at least 30 min. Control: mothers in the control group received the routine education provided in the NICU.	*Maternal satisfaction*	During babies’ admission (twice): Day 1 of intervention. Day 10 of intervention.	*Satisfaction questionnaire (Validated*) The ‘*What Being The Parent of a Baby is Like-Revised’ Questionnaire* was used. The original English version by Pridham and Chang was translated to Persian. Eleven questions. Total satisfaction score range (11–99).	There was a significant difference in the mean score of satisfaction between cases and controls while the mean score of satisfaction increased in both groups. **Comparison of the mean score between the two groups showed that the level of satisfaction was significantly higher in the case group vs the control group**. **Intervention Control** **before intervention** **Mean (SD)** 81.62 (13.50) 85.71 (9.46) **P value** 0.993 **after intervention** **Mean** (**SD)** 93.88 (5.38) 90.12 (7.78) **P value** 0.024	**No**	**1**
2. Kadivar *et al*. (2017), Iran	Mothers/70	Mean (SD) Control 31.6 (2.4) Interv: 32.9 (3.1) /level not stated	Non-randomised, convenience sampling. *Unit level effect*: Two different time periods.	Intervention: **narrative writing**. Mothers did narrative writing at least three times until the 10th day of admission. Control: mothers in the control group received the routine NICU treatment and care.	*Mothers’ satisfaction with medical care provided by physicians, medical students and nurses during neonatal admission to the NICU.*	During babies’ admission (twice): Day 3 of intervention. Day 10 of intervention.	*Satisfaction questionnaire (Validated*) The *NIPS questionnaire* by Mitchell *et al* was used and translated to Persian. 24 questions (Likert scale) Likert (1 always or not satisfied–7 never or completely satisfied). A higher score indicates more satisfaction.	**The satisfaction level of the mothers in the intervention group increased significantly during the study**. The results of independent t-test showed a significant difference in the satisfaction changes of the mothers on the 3rd and 10th day of NICU admission between intervention and control groups, indicating the effectiveness of narrative writing. The results of paired t-test also showed a significant difference in the mean satisfaction level of the mothers between the 3rd and the 10th day in the intervention group. **Interv Control** **After intervention** **Mean** 137 (15.2) 102.3 (25.6) (**SD**) **P value** 0.001	**No**	**1**
3. Garingo *et al*. (2016), USA	Not stated/9	23–39/level III	Non-randomised, convenience sampling. *Group level effect:* Intervention/control groups Postintervention group testing only.	Intervention: **tele-rounding**. Infants of intervention parents were cared for by an OFFSN who was present via a remote-controlled robot. The OFFSN assessed infants via the robot’s integrated stethoscope, with assistance from the nursing staff. During routine hours, the OFFSN was called to discuss any issues with the patient. Emergencies/out of hours were covered by an ONSN. Control: infants of control parents received ONSN care. The attending neonatologist made daily patient rounds with the NICU team. After patient rounds, the NICU staff, under the supervision of the attending neonatologist implemented the care plan.	*Satisfaction with telemedicine.*	During babies’ admission (once): At the time of discharge. No preintervention parent satisfaction data available for comparison.	*Satisfaction questionnaire* Validation: no content validity or reliability testing reported. Number of questions: not stated. Likert (1 excellent–5 very poor).	**Only the intervention group was assessed and only postintervention**. The authors reported that the parents surveyed were ‘*satisfied with their experience. 100% responded that they felt comfortable talking to the OFFSN on the mobile robot and would allow their infant or themselves to be cared for by a physician via telemedicine in the future’.*	**No**	**4**
4. Globus *et al*. (2016), Israel	Mothers and fathers/total surveys returned: 178	~40% in each group <32/level III	Non-randomised, convenience sampling. *Unit level effect:* Two different time periods.	Intervention: **SMSi**. Parents were updated daily regarding the health status of their infant via SMS from the EMR. All SMS messages were sent at 09:00, including one-sentence sections with updated information (eg, location of the infant’s crib and current weight). Information regarding acute events/deterioration of the infant’s medical condition was not included in the SMS, but was delivered personally to the parents in real time. Control: routine care pre-SMS implementation.	*Parent satisfaction related to parent communication with the medical staff.* *Overall parent satisfaction with treatment and staff attitudes throughout hospitalisation.*	During babies’ admission (once): pre-SMS cohort and post-SMS cohort. No preintervention parent satisfaction data available for comparison (different parent groups preintervention and postintervention).	*Satisfaction questionnaire* The ‘*Parents' attitudes regarding their experience during their infants' hospitalisation in the NICU’* questionnaire was used, as well as selected items from a literature review of similar questionnaires, including that by York Hospital and by Conner and Nelson. Validation: no content validity or reliability testing reported. Selected items related to four aspects of the NICU experience. Two out of four directly assessed parent satisfaction: 1. Parental assessment of their communication with the medical staff. Likert scale (1 do not agree at all–5 strongly agree). 2. Overall satisfaction with treatment and staff attitudes throughout hospitalisation. Visual analogue scale (scores range 0–10). Higher scores reflect greater satisfaction.	Overall, in both periods, parents expressed a high degree of satisfaction regarding the medical treatment, the information given and the communication with the medical staff. Overall satisfaction with treatment and with staff attitudes throughout hospitalisation was slightly greater in the post-SMS cohort but did not reach statistical significance. **In the post-SMS cohort, a statistically significant improvement was noted regarding physician availability and patience, parental feelings of comfort in approaching the physicians and nurses and regularly receiving information regarding the infants' medical status from the physicians**. **Post-SMS Pre-SMS** **Mean (SD**) 4.1 (1.0) 3.7 (1.3) **P value** 0.03 *Specific question: “I was pleased with the frequency with which I received information regarding my infant”*. Although improvement in all other categories was documented, it did not reach statistical significance.	**No**	**1**
5. Kazemian *et al*. (2016), Iran	Mothers/220 newborns (assumed 220 mothers)	>37/level not stated	Non-randomised, convenience sampling. *Group level effect:* Intervention/control groups Postintervention testing only.	Intervention: **rooming-in care**. Mothers and babies were admitted to a different atmosphere to the routine care. This facilitated the mothers and neonates with separate beds along with phototherapy devices and nursing clinical supervision. Control: the routine care practised in this neonatal unit supported partial stay of mothers beside their neonates, while sitting on chairs; however, most of the time the mother-infant dyad was separated.	*Maternal satisfaction with the neonatal care services and hospital stay comfort.*	During babies’ admission (once): Not stated exactly when. No preintervention parent satisfaction data available for comparison.	*Satisfaction questionnaire* Validation: no content validity or reliability testing reported. The authors state, ‘*a validated self-made questionnaire was employed, which was filled in by some trained midwives’.* No further information on validation processes, number of questions or name of the questionnaire was provided. Likert (5 very satisfied–1 dissatisfied).	**The level of satisfaction was significantly higher in the intervention group, compared with that in the control group**. **Interv Control** **Satisfaction %** 26.6 18.8 **P value** 0.027	**No**	**1**
6. Van de Vijver and Evans (2015), UK	Not stated/105	Not stated/not stated	Non-randomised, convenience sampling. *Unit level effect:* Three different time periods.	Intervention: **baby diary**. Each parent received a communication diary on their infant’s admission to the unit. Staff wrote-in infant status updates and kept an infant interaction log with parents. Parents wrote in memories and questions for staff to address during face-to-face communication. Control: routine care, before implementation of the diaries.	*Satisfaction with communication from neonatal staff.*	During babies’ admission (three times): On the day of babies’ discharge at study baseline. On the day of babies’ discharge at 1 month. On the day of babies’ discharge at 15 months.	*Satisfaction questionnaire* The study team designed a questionnaire, based on the Department of Health and the National Institute for Health and Care Excellence quality standards for specialist neonatal care. Validation: no content validity or reliability testing reported. Five questions (‘yes or no’).	**Small numbers. No data indicating statistical analysis conducted or evidence of statistically significant results**. *“I was receiving regular communication from staff”* 94%—1 month postdiary cohort 93%—15 months postdiary cohort 77%—prediary cohort *“My questions and concerns were being addressed”* 100%—1 month postdiary cohort 93%—15 months postdiary cohort 91%—prediary cohort “*I feel more involved in my baby's care*” 92%—1 month postdiary cohort 100%—15 months postdiary cohort 88%—prediary cohort	**Yes**. The intervention concept was created by the project leaders following analysis of baseline survey results and used after multidisciplinary input and discussion with staff and parents.	**3**
7. Voos and Park. (2014), USA	Not stated/62	Not stated/level III	Non-randomised, convenience sampling. *Unit level effect:* Two different time periods.	Intervention: **OUpolicy**. Parents were allowed access to their baby 24 hours a day, 7 days a week. Control: parents pre-OU implementation received routine care. The unit was closed to parents during nurse change of shift in mornings and evenings.	*Parent satisfaction with how much time parents get to spend with their baby.*	After babies were discharged (once): After pre-OU parents were discharged. After post-OU parents were discharged.	*Single question (from a validated questionnaire*) The question *“Did you get to spend as much time as you wanted with your baby?”* was used from the National Research Corporation Picker parent survey. One question (‘yes or no’).	**Small numbers. No data indicating statistical analysis conducted or evidence of statistically significant results**. *“Did you get to spend as much time as you wanted with your baby?” Yes*. Pre-OU 78% (18/23) Post-OU 92% (36/39)	**Yes**. The NICU has a Family-centred care committee including parents, which conducted this project.	**3**
8. Segre *et al*. (2013), USA	Mothers/23	Mean (SD) 31.57 (5.30)/level III	For the outcome of parent satisfaction: Non-randomised, convenience sampling. *Group level effect:* Intervention/control groups Postintervention group testing only.	Intervention: **LV**. Mothers met with the LV provider for up to six 50 min LV sessions, conducted in a private hospital, every 2–3 days, within 1 month. Visits entailed greeting, debriefing, updating on current issues, working an agenda through listening and problem solving and providing closure through summary. Control: women who did not meet the specific criteria (eg, minimum score on depression scale) were not invited to join the treatment trial and received routine NICU care/support instead.	*Satisfaction with the treatment and the outcome.*	During babies’ admission (once): Not stated exactly when. No preintervention parent satisfaction data available for comparison.	*Satisfaction questionnaire* The *Client Satisfaction Questionnaire* was used. Validation: partially reported. Authors stated reliability testing took place; no information on content validity provided. Eight questions. Format of questions: not stated.	**Only the intervention group was assessed and only postintervention**. The authors reported: *“The majority of women who received LVs were highly satisfied with the intervention”*. *“The average score for the Client Satisfaction Questionnaire was 29.91, comparable to levels of satisfaction reported by clients receiving depression treatment from a mental health professional”.* *“91.3% of our participants rated the quality of help they received as excellent”.*	**No**	**4**
9. Palma *et al*. (2012), USA	Not stated/26 families returned the survey containing the satisfaction measure)	Not stated/level II	Non-randomised, convenience sampling. *Unit level effect:* Two different time periods.	Intervention: **YBDU. A daily parent update letter generated from the EMR**. Parents were given daily YBDU reports, printed automatically from the EMR. The YBDU included information about an infant’s status during the past 24 hours and a hand-written update by the infant’s care provider. Control: parents received routine care and usual verbal updates (6 months pre-adoption of YBDU).	*Satisfaction with YBDU.*	During babies’ admission (once): Not stated exactly when. No preintervention parent satisfaction data available for comparison (different parent groups preintervention and postintervention).	*Satisfaction questionnaire* A questionnaire including items regarding adoption of and satisfaction with YBDU was used. Validation: no content validity or reliability testing reported. Number and format of questions: not stated.	**Only the intervention group was assessed and only postintervention**. The authors reported: *“When asked to rate the statement ‘I like receiving Your Baby’s Daily Update’, 96% of families who used YBDU as an information source responded with the highest rating, ‘always’”.*	**No**	**4**
10. Voos *et al*. (2011), USA	Not stated/28	Not stated/level not stated	Non-randomised, convenience sampling. *Unit level effect:* Two different time periods.	Intervention: **FCRs**. Parents were invited to attend rounds and choose their level of involvement (attend every day/not at all/periodically). For confidentiality concerns, parents were asked to step outside while rounds of others’ infants took place. The staff augmented FCRs by meeting with parents again after rounds if needed. Control: parents received routine care. Prior to FCR implementation parents were asked to leave the unit during rounds.	*Global satisfaction with the NICU experience.*	During babies’ admission (twice): Prior to FCR. 6 months after starting FCR.	*Satisfaction questionnaire (Validated*) *The NIPS* questionnaire. 24 questions: looking at satisfaction in different areas of the NICU (medical caregivers, communication, tests and procedures). Likert scale (1–7 points).	**A subset of NIPS items related to communication (ie, being kept informed as to changes in the infant’s condition, meeting with physicians, and information about long-term expectations) yielded a significant increase from pre-FCR to post-FCR scores**. **post-FCR pre-FCR P value** **NIPS** 5.5 4.4<0.01 **score** The average score on the NIPS did not change significantly.	**No**	**1**
11. Weiss *et al*. (2010), USA	Mothers/84	Mean (SD) Preintervention group: 32 (4.4) Postintervention group: 32 (9)/level III	Non-randomised, convenience sampling *Unit level effect:* Two different time periods.	Intervention: **an intervention to increase PMP availability and communication frequency**. (1) A brief education module for PMPs was introduced, (2) parents received a contact card with PMP names, job descriptions and contact information, (3) a poster of the faces, names and titles of the PMPs was placed at NICU entrance. Control: parents received routine care in the preintervention cohort, without the above.	*Parent satisfaction with physician and nurse practitioner communication.*	During babies’ admission (twice): Preintervention. Postintervention.	*Satisfaction Questionnaire (Validated*) A pilot survey written by Press Ganey and the Picker Institute was used and revised based on parent responses. Six open-ended questions (quantity of communication) Six Likert scale questions (range questions (availability, understanding, reciprocity, empathy, overall satisfaction).	**Overall satisfaction, based on the ordinal analysis of the 5-point Likert scale, was significantly higher after the intervention (p<0.01**). **Overall satisfaction, dichotomised into a satisfied subgroup and a dissatisfied subgroup for each cohort, was also significantly increased after the intervention**. **Post-interv Preinterv** **Very** 97% (32/33)74% (37/50) **satisfied/** **Somewhat satisfied** **P value**<0.01	**No** Authors stated that only after trialling the intervention many parents (both satisfied and unsatisfied) gave suggestions to improve it.	**1**
12. Foster *et al*. (2008), Australia	Mothers and fathers/93 5 special care nurseries	Mean (SD) Headbox: 36.5 (2.6) CPAP: 36 (3) /level I	Non-randomised, convenience sampling *Group level effect:* Intervention 1/intervention 2 groups Postintervention testing only.	Intervention 1: **infants received headbox oxygen treatment for respiratory distress**. Intervention 2: **infants received CPAP treatment for respiratory distress**.	*Satisfaction with treatment (ie, headbox oxygen or CPAP).*	During babies’ admission (once): Within 5 days of the babies’ admission. No preintervention parent satisfaction data available for comparison.	*Single question* Validation: no content validity or reliability testing reported. One Likert scale question (1 not at all satisfied–5 extremely satisfied).	**Parents with babies receiving CPAP rated their satisfaction with the baby’s treatment statistically significantly higher than the headbox group mean rating**. **Headbox CPAP** **Mean** 3.71 (1.31) 4.51 (0.79) (**SD)** **P value** 0.001 The CPAP group averaged between *very and extremely satisfied* compared with parents of babies receiving headbox, who averaged between *satisfied and very satisfied ratings*.	**No**	**1**
13. Byers *et al*. (2006), USA	Only mothers reported/35	Preterm infants Mean (SD) Control: 28.9 (3.44) Interv: 28.6 (3.37) /level II/III	For the outcome of parent satisfaction: Non-randomised, convenience sampling *Group level effect:* Intervention/control groups Postintervention testing only.	Intervention: **infants received individualised, developmentally supportive FCC**. Infants received care within the framework and philosophy of individualised, developmentally supportive family centred interventions. Control: infants received the traditional NICU standard of care.	*Parent satisfaction relating to:* *Parental perceptions of staff caring* *Education received* *Preparation for the parental role* *Overall satisfaction with the NICU experience.*	During babies’ admission (once): On the day before discharge. No preintervention parent satisfaction data available for comparison.	*Satisfaction questionnaire* The *NICU’s parental satisfaction tool* was used. Validation: partially reported. Authors stated content validity testing took place, but ‘because of the disparate nature of the items, survey reliability was not assessed’. Eleven questions Likert scale (1–5 strongly agree).	**Independent t-test analysis of parent satisfaction/perception scores showed no significant difference between groups**. Example statement: “*I was satisfied with the car my baby and I received in the NICU”* **Interv Control** **Mean** 4.94 (0.23) 4.71 (0.47) (**SD)** **P value** 0.064 Both groups reported very high satisfaction with their NICU experience (4.4–5.0)	**No**	**2**
14. Mills *et al*. (2006), USA	Not stated/not stated Parents of infants from six hospitals	Not stated/level not stated	Implementation project PDSA quality improvement testing.	Intervention: **5 PBPs in the area of discharge planning**. The project team iteratively implemented 5 PBPs: Created an easy-to-use, easy-to-access discharge planning tool kit. Restructured communication tools and processes to reflect a ‘plan for the day, the stay and the way’ to discharge. Maximised the impact and use of caregiver educational tools, and updated materials and delivery systems for caregiver education. Used various continuous quality improvement tools and processes to ensure parent/caregiver and staff satisfaction. Analysed and enhanced interactions with and transfers into the community. Control: N/A. No discrete control group. PDSA quality improvement methodology was applied to parent participants.	*General satisfaction*: *With care* *Parents’ feelings about preparedness for discharge* *Ability and confidence in feeding* *Familiarity with their infant* *Feeling like a parent* *Participation in care* *Adequacy of information from staff about medical and care issues.*	During babies’ admission (four times): Not reported exactly when.	*Satisfaction questionnaire* The internet-based parent satisfaction survey ‘*howsyourbaby.com’* that was developed, especially for this NICU population was used. Validation: no content validity or reliability testing reported. Number and format of questions: not stated.	Through multiple rapid-cycle projects, the project’s collaborative group made changes within the 5 PBP plans. **Parent satisfaction measures were used to longitudinally monitor the changes made, rather than make direct group comparison. No data indicating statistical analysis conducted or evidence of statistically significant results**. Parent satisfaction survey results (all centres combined) were high across four measurement quartiles. No specific interquartile analysis was reported. Parent readiness for discharge was high at the beginning and throughout the collaborative. Parents’ receiving ‘*just the right amount of information’* regarding car seat trials and safe sleep demonstrated some variability throughout the collaborative.	**No**	**3**
15. Wielenga *et al*. (2006), The Netherlands	Mothers and fathers/46	Mean (SD) Control: 28.5 (26.0–29.9) Interv: 28.3 (25.6–29.9) /level III	Non-randomised, convenience sampling *Unit level effect:* two different time periods.	Intervention: **NIDCAP**. Infants received care according to NIDCAP principles and parents were taught how to provide it. Caregiving plans were designed based on the infant’s current developmental stage, medical condition and family needs. Caregivers learnt to watch sensitively and note the infant’s reactions to different types of handling and care, making continuous adjustments. Control: infants received traditional neonatal care practised at that time.	*Parent satisfaction relating to:* *Overall rating* *Care of the baby* *Communication with staff* *Involvement in care -Being prepared* *Support* *Being a parent* *Being near your baby* *Total score.*	After babies were discharged (on day of discharge/transfer): Pre-NIDCAP cohort. Post-NIDCAP cohort.	*Satisfaction questionnaire (Validated*) The *NICU-PSF* was used and translated from English to Dutch. Sixty-two questions. Closed and open-ended questions. Different rating scales used (5-point rating scale from ‘extremely satisfied’ to ‘not at all satisfied’ or ‘excellent’ to ‘poor’). Total score range (50–243 points).	**The intervention group’s mean total score was significantly higher than the control**. **Interv Control** **Mean (SD**) 185.67 (17.74) 174.04 (20.98) **P value** 0.041 Almost all separate concepts showed an increase in their mean scores. The concept of ‘*being a parent’* had a slightly lower mean score (9.39, SD=1.73) in the intervention group than in the control group (9.78, SD=2.09). **The concept of ‘*preparedness’* showed statistically significant difference**: **Interv Control** **Mean** 16.38 13.83 **P value** 0.038	**No**	**1**
16. Penticuff and Arheart (2005), USA	Dyads (both parents or mother with her support person)/122 mothers Results based only on mothers’ data.	Not stated/level III	A repeated measures design: First 2 years (control group data collection). Year 3 (staff training). Year 4 (implementing the intervention). Year 5 (collecting data from the intervention group). *Unit level effect:* Two different time periods.	Intervention: **The newborn individualised IPC- CPM intervention**. Both the mother and father (or the mother and her designated support person) were shown how to use the IPC and attended three CPM (with neonatologists/neonatal nurse practitioners). Control: during the control phase, professionals carried out usual communication and interaction with control group parents.	*Satisfaction with participation in decision making* was measured by five collaboration indices: Satisfaction with Care Relationships with professionals Decision input The process of decision making Decisions made.	During babies’ admission (three times): Within 0–3 days. 9–12 days. 25–28 days of an infant’s admission to the NICU.	*Three satisfaction questionnaires* 1. Two subscales of the investigator-designed ‘*Parents’ Understanding of Infant Care and Outcomes Questionnaire’* were used to measure Satisfaction with Care (1). Validation: partially reported. Authors stated content validity testing took place; no information on reliability testing provided. Thirty questions. Five-point Likert scale. 2. A subscale of the investigator-designed ‘*Relationships with Professional and Decision Input Questionnaire’* was used to measure satisfaction with relationships (2). Validation: partially reported. Authors stated content validity testing took place; no information on reliability testing provided. Twelve questions. Five-point Likert scale 3. Validated. The ‘*Collaboration and Satisfaction About Care Questionnaire’* developed by Baggs, was used to measure Satisfaction with decision input (3), with decision process (4) and with decisions made (5). Nine questions. 7-point scale (1 strongly disagree−7 strongly agree).	**The intervention group was more satisfied with the amount of decision input they had (3) and with the process by which medical decisions were made (4**). **Interv Control P value** Decision input amount (3) **Mean** 33.44 30.05 0.058 Process of decision making (4) **Mean** 120.20 104.95 0.012 There were no statistically significant differences between control and intervention groups in satisfaction with their infants’ care (1), with relationships with NICU professionals (2) and with the decisions made for infant treatment (5).	**No**	1
17. Byers *et al*. (2003), USA	Mothers/19	Mean (SD) Control: 29 (2.00) Interv: 28.9 (2.42) /level II–III	For the outcome of parent satisfaction: Non-randomised, convenience sampling *Group level effect:* Intervention/control groups Preintervention and postintervention testing.	Intervention: **co-bedding premature multiple-gestation infants in incubators**. Infants were nursed in the same incubator using a co-bedding protocol (eg, recording all of the care provided to one infant before providing care to the second infant). Control: single-bedding premature multiple-gestation infants in incubators.	*Parent satisfaction related to:* *Staff concern* *Support of family* *Staff explanations* *Infant environment,* *Comfort with feeding* *Kangaroo care encouragement* *Staff explanation of signs of infant stress* *Visiting schedule* *Overall satisfaction with the NICU experience.*	During babies’ admission (twice): At baseline. 5 days later.	*Satisfaction questionnaire* The NICU’s standard parental satisfaction tool was used. Validation: partially reported. Authors stated content validity testing took place, but because of the disparate nature of the items, survey reliability could not be assessed. Eleven questions. 5-point Likert-type scale.	**The only significant difference for a postintervention item was a higher score for the item ‘Attempts were made to create a quiet environment for my baby’.** **Interv Control P value** **Mean** 4.80 3.89 0.033 Independent t-tests comparing the co-bedded and control group parental scores found no significant differences in their parental satisfaction scores, except for higher baseline parental satisfaction scores (p=0.029) in the co-bedded group.	**No**	**1**
18. Polizzi *et al*. (2003), USA	Mothers and fathers/33	Mean (SD) Control: 32.97 (1.9) Interv: 33.08 (1.31) /level III	A retrospective, comparative, descriptive design. *Unit level effect.*	Intervention: **co-bedding multiple-gestation infants in the NICU**. Multiple-gestation infants were nursed in the same incubator or crib. The intervention was evaluated retrospectively after implementation of a co-bedding practice protocol. Control: traditionally bedded group (babies were routinely placed in separate incubators or cribs).	*Parental satisfaction as measured by nine questions relating to parent perceptions and their baby’s care.*	After babies were discharged (once): All parents were mailed the survey. A second survey was sent to those who did not respond after 2 months. No preintervention parent satisfaction data available for comparison.	*Satisfaction questionnaire* The *parental perception/satisfaction tool* was used. Validation: partially reported. Authors stated content validity testing took place; no information on reliability testing provided. 6/9 questions were from a similar tool that was validated by the Vermont Oxford NICU Quality Improvement Initiative. Nine questions (such as *“I was satisfied with the care my babies received in the hospital”*). Likert (1 strongly disagree–5 strongly agree).	Mothers reported overall satisfaction with the NICU care and staff, as well as adequacy of their ability to care for their infants after discharge, with scores ranging from 4.19 to 4.71. **The only survey item score that was significantly different between groups was for the item *“I was encouraged by the hospitalstaff to bond with my babies”.*** **Interv Control P value** **Mean** 4.71 4.36 0.049	**No**	**1**
19. Legault and Goulet (1995), Canada	Mothers/61 completed both tests	Mean (range) 30 (24–35) /level II	Time-series design *Group level effect:* Same group exposed to both methods with postmethod testing only.	Intervention: **Kangaroo method of removing an infant from an incubator**. Mothers were taught the ‘kangaroo method’ (skin-to-skin contact): infant wears a diaper/head cap and is placed in a vertical position on the parent’s bared chest. A blanket covers the infant and the parent’s clothing is fastened around the infant. The parent sits in a rocking chair, inclined so that the infant’s head is at 60. Control: traditional method. Newborns wearing a diaper and a head cap, are wrapped in a blanket and placed in their parent’s arms.	*Mothers’ satisfaction with:* *Each method of removing an infant from incubator* *Her feelings after each method.*	During babies’ admission (twice): After the intervention. After the control method. No preintervention parent satisfaction data available for comparison.	*Satisfaction questionnaire* The ‘*Maternal Satisfaction Questionnaire’* was used. It was developed by integrating components described by Affonso *et al* and the clinical experience of the investigators. Validation: partially reported. Authors stated content validity testing took place; no information on reliability testing provided. Fifteen questions Likert (1 very much–5 do not know). An open-ended question invited the mother to list and explain anything else related to her experience.	**Regardless of the method tested, mothers expressed high levels of satisfaction (it was the first time since giving birth that they could hold their infants**). Three statements proved more powerful in discriminating between the methods: **Rated higher after the kangaroo method test:** *“I like the contact with my baby’s skin”* (p=0.0001) **Rated higher after the traditional method test:** *“I like to talk to and whisper to my baby“ (p=0.015*) *“I looked into my baby’s eyes and stared at his/her face“ (p=0.0001*)	**No**	**1**

Number in last column illustrates each intervention’s reported effect on parent satisfaction: 1. Parent satisfaction was statistically significantly higher in the intervention group; 2. Parent satisfaction was not reported to be statistically significantly different in the intervention group; 3. Unclear if parent satisfaction improved (small study numbers and/or no statistical analysis performed); 4. Only the intervention group was assessed.

CPAP, continuous oxygen positive airway pressure; EMR, electronic medical record; FCR, family centred round; IPC-CPM, Infant Progress Chart-Care Planning Meetings; LV, listening visits; N/A, not available; NIDCAP, Newborn Individualised Developmental Care and Assessment Programme; NIPS, Neonatal Index of Parent Satisfaction; OFFSN, off-site neonatologist; ONSN, on-site neonatologist; OU, open unit; PBP, potentially better practice; PDSA, Plan Do Study Act; PMP, principal medical providers; SMSi, short message services implementation; YBDU, your baby’s daily update.

1. *‘Unit-level effect’:* studies that assessed parent satisfaction during a period of routine care (control group) and introduced the intervention at a later time, with a different group of parents. In these studies, improvement in parent satisfaction was evaluated between different parent groups, on a *unit level*.
2. *‘Group level effect’:* studies that formed intervention and control groups using convenience sampling during the same time period. Both groups (or sometimes only the intervention group) had satisfaction measured after the intervention period (postintervention testing). Baseline parent satisfaction was also measured in both groups (preintervention testing) in some studies. Improvement in parent satisfaction was demonstrated either by comparing outcomes between intervention/control groups following the intervention, or in comparison with the preintervention data.

Parent participants included mothers (14 studies), mothers and fathers (10 studies) or were not specified (7 studies). One study defined parent participants as a dyad of the mother with her designated support person. Median parent sample size was 63, ranging 7–482. This was higher for RCT (108 studies) compared with non-RCT (61 studies).

Study participants included parents of babies across the full range of gestations (23–42 weeks). Overall, 24/32 (75%) of studies involved preterm infants, 5/32 (16%) term infants and 7 studies did not state the gestational age of infants involved. Most studies (19, 59%) involved only preterm infants (up to 37 weeks); only one study (3%) involved only term infants and five studies (16%) involved both preterm and term infants. Preterm infants were included in 44% of RCT vs 63% of non-RCT.

Most studies were reported as conducted in level III neonatal units (17 studies), followed by level not stated (9 studies), level II–III (3 studies), level II (2 studies) and level I (1 study). Definitions of neonatal levels of care are not standardised but vary across different countries; none of the included studies have explicitly stated which definition applies to them.

Tables 1–3 show the key characteristics of included studies. They include a description of each study’s parent and infant sample, study design and intervention, outcome measures (timing and methods), results, parent input into intervention design and study impact on parent satisfaction.

### Parent satisfaction

#### Outcome measures

All 32 studies reported they measured parent satisfaction as an a priori outcome. Only one study confirmed this through a protocol. Overall, 18/32 (56%) of studies (4/10, 40% RCT and 14/22, 64% non-RCT) reported a higher level of parent satisfaction associated with the intervention studied. Multiple different outcome measures within the domain of parent satisfaction were used; we grouped these into four categories: i) parent satisfaction (no additional description); ii) parent satisfaction with NICU care; iii) parent satisfaction related to specific components such as communication, staff or information; iv) parent satisfaction with a specific intervention.

#### Timing of measurement

Parent satisfaction was mostly measured *‘during infant admission only’* (24 studies; between 1 and 4 times), followed by *‘after infant discharge only’* (5 studies; 1 time) and ‘*both during admission and after discharge’* (3 studies; between 1 and 3 times). In the majority of studies (19/32, 59%), no preintervention parent satisfaction measurements were conducted in the same parent groups with available postintervention data (ie, paired parent data for satisfaction levels did not exist). Instead, impact of interventions was determined comparing intervention/control group measurements in different time periods (tables 1–3).

#### Method of measurement

Parent satisfaction was assessed using 32 different methods: 29 different questionnaires, 2 different single questions and by structured interview in 1 study; in total, 334 different questions were used to assess parent satisfaction. Only 6/29 (21%) of questionnaires were reported to be fully validated (both content validation and reliability testing); 23/29 (79%) questionnaires were partially or completely unvalidated. The most commonly used questionnaire was the validated *Neonatal Index of Parent Satisfaction*^17^ questionnaire (three studies).

### Interventions and impact on parent satisfaction

We grouped included studies into five intervention themes: parent involvement (14 studies); information provision/communication (8 studies); clinical care (7 studies); parent emotional support (2 studies); other (1 study). Parent involvement interventions were more commonly assessed in RCT compared with non-RCT.

We categorised interventions *as effective* or *not effective* based on whether a statistically significant difference between intervention and control groups was reported for parent satisfaction (boxes 1 and 2). None of the studies reported statistically significantly lower parent satisfaction in the intervention group compared with the control group. We classified studies as *unclear if effective* if they included small sample numbers or if statistical analysis was not performed (box 3). Finally, we highlighted studies where *only the intervention group was assessed and only postintervention,* where comparison to a control group was not possible (box 4).

Box 1 **‘*Effective’* interventions in themes**

**Theme: parent involvement**

More NICU access, parents on WRs, education (De Bernardo *et al*, Italy, 2017)

Rooming-in care (Kazemian *et al*, Iran, 2016)

Parental presence at clinical bedside rounds (Abdel-Latif *et al*, Australia, 2015) *RCT*

More NICU access, care involvement, education (Bastani *et al*, Iran, 2015) *RCT*

Education regarding pain management (Franck *et al*, UK, 2011) *RCT*

Single-family NICU rooms (Stevens *et al*, USA, 2011)

Family centered rounds (Voos *et al*, USA, 2011)

Newborn Individualised Developmental Care and Assessment Programme (Wielenga *et al*, The Netherlands, 2006)

Infant progress charts filled by parents and three care planning meetings (Penticuff and Arheart, USA, 2005)

Kangaroo care (Legault and Goulet, Canada, 1995)

**Theme: information provision / communication**

Internet-based education (Kadivar *et al*, Iran, 2017)

Daily SMS from electronic patient record (Globus *et al*, Israel, 2016)

Staff education, staff contact card given to parents, staff poster at NICU reception (Weiss *et al*, USA, 2010)

Provision of taped conversations with neonatologists to mothers (Koh *et al*, Australia, 2007) *RCT*

**Theme: clinical care**

- Headbox oxygen for respiratory distress
- Continuous oxygen positive airway pressure for respiratory distress (Foster *et al*, Australia, 2008)

Co-bedding infants in incubators (prospective) (Byers *et al*, USA, 2003)

Co-bedding infants in incubators (retrospective) (Polizzi *et al*, USA, 2003)

**Theme: parent emotional support**

Narrative writing (Kadivar *et al*, Iran, 2017)

Interventions where parent satisfaction was reported to be statistically significantly higher in the intervention group.NICU, neonatal intensive care unit; RCT, randomised controlled trial; WR, ward round

Box 2 **‘*Ineffective’* interventions in themes**

**Theme: parent involvement**

- Massage with auditory, tactile, visual and vestibular stimulation
- Kangaroo care (Holditch-Davis *et al*, USA, 2013) *RCT*

Individualised, developmentally supportive family centred care interventions (Byers *et al*, USA, 2006)

**Theme: information provision/communication**

Sharing information obtained from parent interviews with the primary NICU provider (Clarke-Pounder *et al*, USA, 2015) *RCT*

**Theme: clinical care**

Clinical nurse specialist/neonatal practitioner team care (Mitchell-DiCenso *et al*, Canada, 1996) *RCT*

**Theme: other**

Free parking (Northrup *et al*, USA, 2016) *RCT*

Interventions where parent satisfaction was not reported to be statistically significantly different in the intervention group.RCT, randomised controlled trial.

Box 3 ***‘Unclear if effective’* interventions in themes**

**Theme: parent involvement**

Open unit policy: 24/7 NICU access (Voos and Park, USA, 2014)

Touch and massage for 7 days (Livingston *et al*, USA, 2009) *RCT*

**Theme: information provision/communication**

Clinical staff enter updates in baby diary (Van de Vijver and Evans, UK, 2015)

Detailed information provided during consenting (Broyles *et al*, USA, 1992) *RCT*

**Theme: clinical care**

Palliative care (Petteys *et al*, USA, 2015)

Five potentially better practices in the area of discharge planning (Mills *et al*, USA, 2006)

Interventions where small study numbers and/or no statistical analysis performed).RCT, randomised controlled trial.

Box 4 **Interventions in themes where ‘*only the intervention group was assessed and only postintervention’***

**Theme: information provision/communication**

Daily parent update letter from electronic patient record (Palma *et al*, USA, 2012)

**Theme: clinical care**

Tele-rounding robot, off-site neonatologist (Garingo *et al*, USA, 2016)

**Theme: parent emotional support**

Listening visits (Segre *et al*, USA, 2013)

Overall, 18/32 studies (56%) reported higher parent satisfaction in the intervention group; 4/10 RCT and 14/22 non-RCT. The intervention theme where higher satisfaction was most consistently reported was parent involvement (10/14 studies). Due to the large heterogeneity of outcome measure scales, a quantitative synthesis and meta-analysis was not possible.

### Parent input into design of interventions

Five studies (5/32, 16%) reported involving parents in intervention design, of which two reported improvement of parent satisfaction. The number of included studies was too small to estimate any effect of parent co-design on the success of interventions at study level.

### Methodological quality

**Figure 2:**
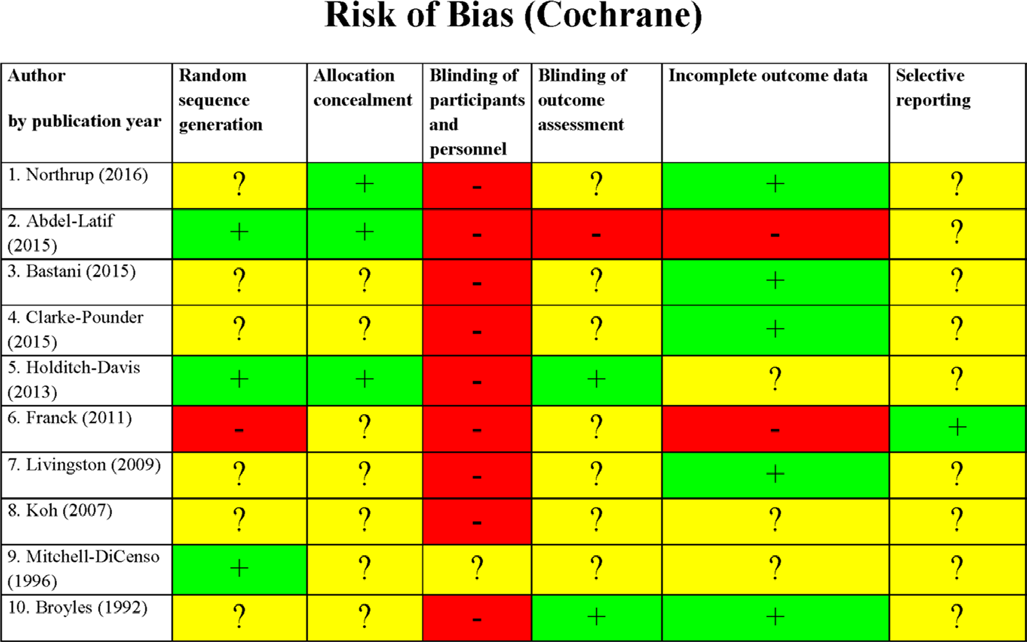
Cochrane Collaboration risk of bias tool assessment (randomised controlled trial). Green: low risk of bias; yellow: unclear risk of bias; red: high risk of bias.

For the majority of RCT, key study characteristics, such as randomisation, allocation concealment and blinding of outcome assessment, were either not stated or unclear (figure 2). Only one RCT had an available study protocol (retrospectively registered) and none described blinding of study participants and/or personnel. All RCT scored a high/unclear risk of bias in at least 4/6 Cochrane tool categories, except for one, which scored a high/unclear risk in 3/6 categories.

**Figure 3:**
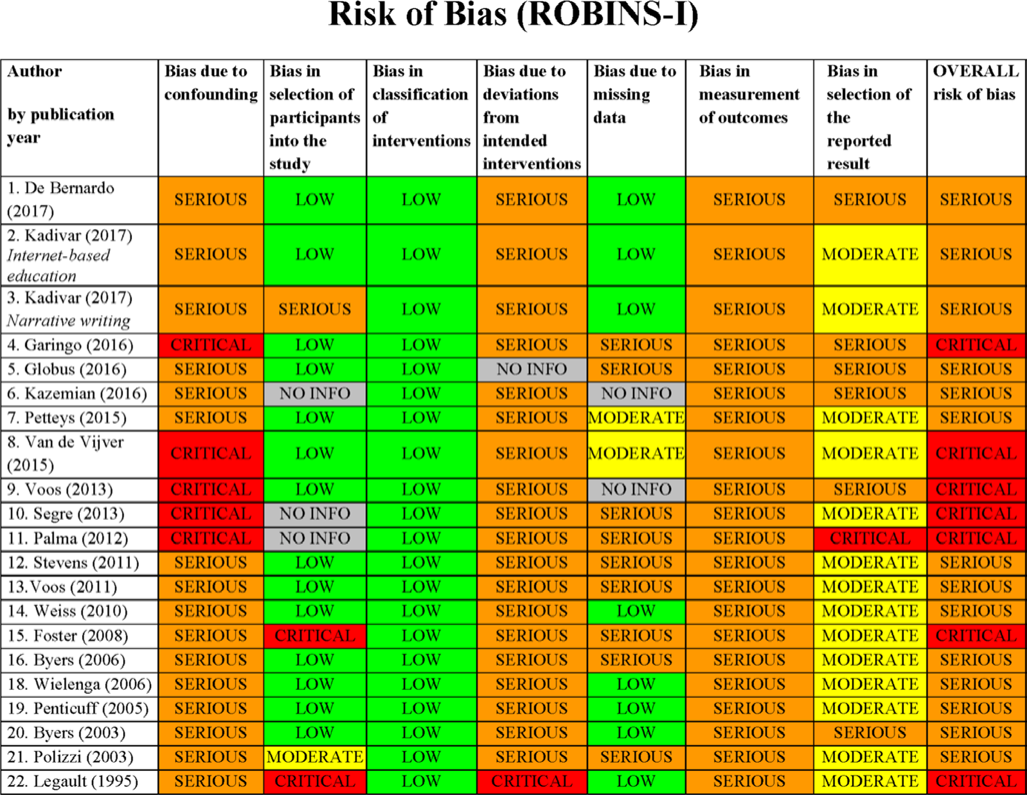
ROBINS-I risk of bias assessment (non-randomised controlled trial).

We assessed 21/22 non-RCT studies using the ROBINS-I tool^13^, excluding the implementation project. All 21 studies were assessed as having an overall *serious* risk of bias and 7/21 of studies (33%) were further categorised as having *critical* risk of bias (figure 3). Blinding of participants, personnel and outcome assessment was poorly reported across all non-RCT and no study reported a published study protocol. None of the included non-RCT measured or corrected for important parent/infant confounding variables, or other relevant neonatal unit co-interventions taking place at the same time as the intervention.

We were unable to use the *Standards for Reporting Implementation Studies (StaRI) Statement Tool*^18^ for assessing the implementation project, as the reporting was incomplete.

There was no association between methodological quality assessments and the studies’ reported effect on parent satisfaction. All 4/10 RCT that reported a higher level of parent satisfaction associated with their intervention, scored a high/unclear risk of bias in at least 4/6 Cochrane tool categories, one of which scored high/unclear *risk* in all categories. Out of the 14/22 non-RCT reporting an improved parent satisfaction, two were deemed to be at *critical risk* of bias on the ROBINS- I tool, while the rest we assessed to be at *serious risk* of bias.

## Discussion

Parent satisfaction with neonatal care is increasingly recognised as an important measure of parent experience and is being used to evaluate hospitals and healthcare providers; use of interventions to improve parent satisfaction in neonatal units is increasing. This is the largest review of interventions where an outcome was parent satisfaction with neonatal care and includes 32 studies. We find low-quality evidence that interventions targeting ‘parent involvement’ may improve parent satisfaction with neonatal care, but this result must be interpreted cautiously in view of the high risk of bias in included studies.

Overall, our review highlights the complexity of evaluating parent satisfaction. As a multidimensional construct, parent satisfaction can be affected just as much by interventions directly relating to infant care (eg, Kangaroo care) as well as interventions relating to neonatal care facilities (eg, Free parking). By grouping included interventions into themes (boxes 1–4), we have highlighted the variety of interventions available, as well as the majority of interventions being those relating to ‘parent involvement’.

A key reason for only selecting parent satisfaction as the outcome of interest was to focus on a single component of parent experience, in order to reduce outcome heterogeneity and allow direct comparison. Despite this approach, the key methodological limitation identified in this review was inconsistency in how parent satisfaction is defined and measured; it is notable that the majority of questionnaires (23/29) lack validation. In keeping with neonatal studies more widely,^19^ this study confirms inconsistent outcome selection as a major source of research waste in neonatal studies examining parent experience, and further finds that there is limited involvement of parents in study design.

Strengths of our review include identifying studies with both mother and father participants, inclusion of the full range of infant gestations and a wide range of interventions. We followed a preregistered protocol and report this review in line with PRISMA guidelines.^11^ To further aid direct comparison of interventions, we only included studies that evaluated parent experience using ≥1 quantitative outcome of parent satisfaction. One limitation of this approach is that by excluding studies which evaluated parent experience using other measures (eg, stress, anxiety and depression scales), we are unable to comment on interventions that targeted these other components of parent experience.

Another limitation is that we have only included studies in the English language, due to resource and time constraints. By not including studies in other languages, it is possible our results are more focused on work conducted in specific countries. Furthermore, we acknowledge that much of the research in parent experience is qualitatively evaluated. By restricting our review to studies where ≥1 quantitative outcome of parent satisfaction is measured, we have not included any interventions with solely qualitative outcomes. This was in an attempt to enable direct comparison of interventions, which has previously not been possible in any published review. By not including studies evaluated by qualitative measures only, it is possible our results are more focused on a particular type of interventions where quantitative evaluation would be preferable and/or easier. It also means we may not have included all studies ever conducted on a particular intervention, where some were only evaluated qualitatively, making some interventions appear more ‘widespread’ than others.

Brett *et al*^20^ systematically reviewed interventions aimed at improving the parent experience more widely, but only included parents of preterm infants. Their large number of outcome domains and heterogeneity of outcome measures (including studies that reported only qualitative outcomes) meant the **authors we unable to draw firm conclusions** about the efficacy of interventions and that comparison and meta-analysis was not possible. The majority of our review’s studies have been published in the 7 years since the review by Brett *et al*, highlighting the increasing interest in this area. However, despite including all gestations and focusing on a specific aspect of parent experience, heterogeneity in measurement of parent satisfaction meant we were also unable to conduct a quantitative synthesis. Inconsistency and lack of validation of instruments measuring parent satisfaction in neonatal care (specifically with family centred care) has also previously been highlighted by Dall’Oglio *et al*.^21^

Although 31% of included studies were RCT, all were assessed as having a high risk of bias. RCTs are traditionally considered the highest-ranking form of evidence, however it is worth considering whether such a design is feasible or desirable to evaluate interventions targeting parent satisfaction. Parents in neonatal care talk to each other, compare notes and invariably create parent-support communities; hence it is inherently difficult to avoid contamination between parents receiving an intervention and those who are not, meaning that blinding of parents or health professionals is near impossible. Furthermore, parent satisfaction is likely to be particularly susceptible to the Hawthorne effect,^22^ requiring longer-term follow-up. These factors may explain the low number of RCT identified in our review and the high risk of bias seen in those that were included. In non-RCT studies, the main methodological concern is the degree to which unmeasured and uncontrolled confounders may explain any differences seen between groups. The non-RCT studies included in this review were classed as having either a serious or critical risk of bias. The overwhelming majority of studies did not adequately report baseline variables or report other interventions during the study period, making it impossible to assess studies for selection bias or treatment bias. Furthermore, limitations such as contamination bias and the Hawthorne effect affect non-RCT as well. Only two non-RCT studies evaluated the outcome of interest (parent satisfaction) both before and after the intervention, in the same group of parents (*group level effect),* with most studies evaluating different parent groups preintervention and postintervention (*unit level effect*). An inherent weakness of this latter approach is that it assumes parent satisfaction is a static measure at the unit level, which is unlikely to be true. As a result of these numerous important limitations identified across all included studies, we find only low-quality evidence in support of interventions to improve parent satisfaction with neonatal care, despite a majority of studies reporting a beneficial effect of interventions. These limitations may explain the limited uptake of these interventions by the wider neonatal community.

Changing neonatal unit practices to incorporate any new intervention requires robust evidence. We demonstrate here that such evidence is not currently available for improving parent satisfaction. We highlight the use of non-randomised study designs, inconsistency in definition and measurement of parent satisfaction, the use of unvalidated questionnaires, methodological limitations and a lack of parent involvement as contributors. Our review empirically documents the extent of these issues in studies that use quantitative parent satisfaction surveys, and their contribution to research waste in neonatology.

Given the importance of parent satisfaction for both parent and offspring well-being, higher quality trials that involve parents, use of standardised definitions and validated parent satisfaction measures are needed. Given the nature and challenges of the neonatal care environment and the limitations we have identified in existing research, a cluster RCT may be the most appropriate study design to rigorously evaluate interventions to improve parent satisfaction with neonatal care.

## Conclusions

Many interventions, commonly relating to parent involvement, are reported to improve parent satisfaction with neonatal care but inconsistency in definition and measurement of parent satisfaction and high risk of bias in all studies makes this low-quality evidence. Standardised definitions and validated parent satisfaction measures are needed, as well as higher quality trials of parent experience, involving parents in intervention design.

## Supplementary Material

Reviewer comments

Author's manuscript

## Data Availability

All data relevant to the study are included in the article or uploaded as supplementary information.
