# Supplementary material for: Interventions to improve quantitative measures of parent satisfaction in neonatal care: a systematic review
Source: BMJ Paediatr Open. 2020 Mar 15;4(1):e000613. doi: 10.1136/bmjpo-2019-000613 (PMC7073789; doi:10.1136/bmjpo-2019-000613)
Supplement: Supplementary data [file bmjpo-2019-000613supp001.pdf]

1. intervention$.ti,ab.
2. tool$.ti,ab.
3. way$.ti,ab.
4. updat$.ti,ab.
5. method$.ti,ab.
6. information.ti,ab.
7. sms.ti,ab.
8. implement$.ti,ab.
9. 1 or 2 or 3 or 4 or 5 or 6 or 7 or 8
10. bab$3.mp.
11. preterm$.ti,ab.
12. pre term.ti,ab.
13. premature.ti,ab.
14. postterm.ti,ab.
15. post term.ti,ab.
16. infant$.ti,ab.
17. newborn$.ti,ab.
18. exp Infant, Newborn/
19. 10 or 11 or 12 or 13 or 14 or 15 or 16 or 17 or 18
20. neonatal intensive care.ti,ab.
21. neonatal unit$.ti,ab.
22. NICU.ti,ab.
23. SCBU.ti,ab.
24. neonatal itu.ti,ab.
25. special care baby unit$.ti,ab.
26. neonat$.ti,ab.
27. Intensive Care Units, Neonatal/
28. Intensive Care Units/
29. Critical Care/
30. Neonatal Nursing/
31. 20 or 21 or 22 or 23 or 24 or 25 or 26 or 27 or 28 or 29 or 30
32. parent$.ti,ab.
33. mother$.ti,ab.
34. father$.ti,ab.
35. exp Parents/

36. 32 or 33 or 34 or 35
37. satisfaction.ti,ab.
38. experience$.ti,ab.
39. Patient Satisfaction/
40. personal satisfaction/
41. communicat$.ti,ab.
42. exp Communication/
43. Health Communication/
44. Information Dissemination/
45. 37 or 38 or 39 or 40 or 41 or 42 or 43 or 44
46. 9 and 19 and 31 and 36 and 45
