# Supplementary material for: Interventions to improve quantitative measures of parent satisfaction in neonatal care: a systematic review
Source: BMJ Paediatr Open. 2020 Mar 15;4(1):e000613. doi: 10.1136/bmjpo-2019-000613 (PMC7073789; doi:10.1136/bmjpo-2019-000613)
Supplement: Author's manuscript [file bmjpo-2019-000613.draft_revisions.pdf]

BMJ Paediatrics Open

BMJ Paediatrics Open is committed to open peer review. As part of this commitment we make the peer review history of every article we publish publicly available.

When an article is published we post the peer reviewers' comments and the authors' responses online. We also post the versions of the paper that were used during peer review. These are the versions that the peer review comments apply to.

The versions of the paper that follow are the versions that were submitted during the peer review process. They are not the versions of record or the final published versions. They should not be cited or distributed as the published version of this manuscript.

BMJ Paediatrics Open is an open access journal and the full, final, typeset and author-corrected version of record of the manuscript is available on our site with no access controls, subscription charges or pay-per-view fees (http://bmjpaedsopen.bmj.com).

If you have any questions on BMJ Paediatrics Open's open peer review process please email info.bmjpo@bmj.com

BMJ Paediatrics Open

Interventions to improve parent satisfaction in neonatal care: a systematic review

Journal:	*BMJ Paediatrics Open*
Manuscript ID	bmjpo-2019-000613
Article Type:	Original research
Date Submitted by the Author:	18-Nov-2019
Complete List of Authors:	Sakonidou, Susanna; Imperial College London, Academic Neonatal Medicine<br>Andrzejewska, Izabela; Imperial College London, Academic Neonatal Medicine<br>Webbe, James; Imperial College London, Academic Neonatal Medicine<br>Modi, Neena; Imperial College London, Academic Neonatal Medicine<br>Bell, Derek; NIHR CLAHRC for Northwest London<br>Gale, Chris; Imperial College London, Academic Neonatal Medicine
Keywords:	Neonatology, Outcomes research, Patient perspective

SCHOLARONE™
Manuscripts

I, the Submitting Author has the right to grant and does grant on behalf of all authors of the Work (as defined in the below author licence), an exclusive licence and/or a non-exclusive licence for contributions from authors who are: i) UK Crown employees; ii) where BMJ has agreed a CC-BY licence shall apply, and/or iii) in accordance with the terms applicable for US Federal Government officers or employees acting as part of their official duties; on a worldwide, perpetual, irrevocable, royalty-free basis to BMJ Publishing Group Ltd ("BMJ") its licensees and where the relevant Journal is co-owned by BMJ to the co-owners of the Journal, to publish the Work in this journal and any other BMJ products and to exploit all rights, as set out in our licence.

The Submitting Author accepts and understands that any supply made under these terms is made by BMJ to the Submitting Author unless you are acting as an employee on behalf of your employer or a postgraduate student of an affiliated institution which is paying any applicable article publishing charge ("APC") for Open Access articles. Where the Submitting Author wishes to make the Work available on an Open Access basis (and intends to pay the relevant APC), the terms of reuse of such Open Access shall be governed by a Creative Commons licence – details of these licences and which Creative Commons licence will apply to this Work are set out in our licence referred to above.

Other than as permitted in any relevant BMJ Author's Self Archiving Policies, I confirm this Work has not been accepted for publication elsewhere, is not being considered for publication elsewhere and does not duplicate material already published. I confirm all authors consent to publication of this Work and authorise the granting of this licence.

Confidential: For Review Only

Interventions to improve parent satisfaction in neonatal care: a systematic review

Susanna Sakonidou[1], Izabela Andrzejewska[2], James Webbe[3], Neena Modi[4], Derek Bell[5], Chris Gale[6]

1 Susanna Sakonidou, Clinical Research Fellow

Highest academic degree: MBBS

Section of Neonatal Medicine, Imperial College London.

London, United Kingdom.

s.sakonidou@imperial.ac.uk

2 Izabela Andrzejewska, Neonatal Research Nurse

Highest academic degree: RN MSc

Section of Neonatal Medicine, Imperial College London.

London, United Kingdom.

Izabela.ukpl@gmail.com

3 James Webbe, Clinical Research Fellow

Highest academic degree: MB BChir

Section of Neonatal Medicine, Imperial College London.

London, United Kingdom.

j.webbe@imperial.ac.uk

4 Neena Modi, Professor of Neonatal Medicine

Confidential: For Review Only

Highest academic degree: MD

Section of Neonatal Medicine, Imperial College London.

London, United Kingdom.

n.modi@imperial.ac.uk

5 Derek Bell, Professor of Acute Medicine and NIHR CLAHRC Programme Director

Highest academic degree: MD

National Institute for Health Research Collaboration for Leadership in Applied Health Research and Care, Northwest London, United Kingdom.

d.bell@imperial.ac.uk

6 Chris Gale, Clinical Senior Lecturer

Highest academic degree: PhD

Section of Neonatal Medicine, Imperial College London.

London, United Kingdom.

christopher.gale@imperial.ac.uk

Corresponding Author

Susanna Sakonidou, Clinical Research Fellow

Section of Neonatal Medicine, Chelsea and Westminster campus, Imperial College London

369 Fulham Road, London, SW10 9NH, United Kingdom.

+ 44 (0) 203 315 5418, s.sakonidou@imperial.ac.uk

Manuscript word count: 2500

Confidential: For Review Only

ABSTRACT

Objective

Interventions improving parent satisfaction can reduce parent stress, may improve parent-infant bonding and infant outcomes. Our objective was to systematically review neonatal interventions relating to parents of infants of all gestations where an outcome was parent satisfaction.

Methods

We searched the databases MEDLINE, EMBASE, PsychINFO, Cochrane Central, CINAHL, HMIC, Maternity and Infant Care between 1/1/1946-1/10/2017. Inclusion criteria are randomised controlled trials (RCT), cohort studies and other non-randomised studies if participants were parents of infants receiving neonatal care, interventions were implemented in neonatal units (of any care level) and ≥1 quantitative outcome of parent satisfaction was measured. We extracted study characteristics, interventions, outcomes and parent involvement in intervention design. Included studies were not sufficiently homogenous to enable quantitative synthesis. We assessed quality with the Cochrane Collaboration risk of bias tool (randomised) and the ROBINS-I tool (non-randomised studies).

Results

We identified 32 studies with satisfaction measures from over 2800 parents and grouped interventions into 5 themes. Most studies were non-randomised involving preterm infants. Parent satisfaction was measured by 334 different questions in 29 questionnaires (only 6/29 fully validated). 18/32 studies reported higher parent

Confidential: For Review Only

satisfaction in the intervention group. The theme with most studies reporting higher satisfaction was parent involvement (10/14). Five (5/32) studies reported involving parents in intervention design. All studies had high risk of bias.

Conclusions

Many interventions, commonly relating to parent involvement, are reported to improve parent satisfaction. Inconsistency in satisfaction measurements and high risk of bias makes this low-quality evidence. Standardised, validated parent satisfaction measures are needed, as well as higher quality trials of parent experience involving parents in intervention design.

PROSPERO registration: CRD42017072388

Keywords: neonatology, parents, satisfaction

INTRODUCTION

One in 10 newborn babies in high-income countries require neonatal care (1). This is stressful for parents, who often develop anxiety, depression and Post Traumatic Stress Disorder symptoms (2-4). Parental stress interferes with parent-child bonding (5) and there is a well-established link between maternal mental health and infant development (6). Parent satisfaction, defined as *"the perception of parents' needs and expectations being met"* is inversely related to parental stress (7). As such, it is increasingly being used as a parent experience measure and neonatal service quality indicator. Interventions aimed at improving parent satisfaction have the potential to reduce parent stress, improve parent-infant bonding (8) and infant outcomes (9).

Confidential: For Review Only

A range of parent-centred interventions, such as including parents on ward rounds, have recently become widespread in neonatal practice. Many are implemented on a small scale, without evaluating their impact on parent experience, making long-term integration into neonatal services challenging. Moreover, where parent experience is measured, some studies include it as a primary outcome, whereas others use it as a secondary indicator to explore the parent point of view.

There are multiple experience measures available in addition to parent satisfaction, including parent stress, anxiety and depressions scales. Finally, it is not known the degree to which parents are involved in the design of such interventions. There have been no previous systematic evaluations focused on interventions measuring parent satisfaction with neonatal care as an outcome.

The aim of this review is to identify and describe neonatal interventions relating to parents of infants of all gestations where an outcome was parent satisfaction. We aim to report each intervention's effect on parent satisfaction, as well as parent input in intervention design.

METHODS

We prospectively registered this study on PROSPERO (11) (prospective register of systematic reviews-CRD42017072388) and reported it using PRISMA guidelines (12). We searched MEDLINE (Medical Literature Analysis and Retrieval System Online), EMBASE (Excerpta Medica database), PsychINFO (Psychological Information), Cochrane Central Register of Controlled Trials, CINAHL (CUMULATIVE Index to NURSING and Allied HEALTH LITERATURE), HMIC

Confidential: For Review Only

(Health Management Information Consortium), Maternity and Infant Care (online_ supplementary_ file_ 1) for English papers published between 1946-October 2017, with update searches on 1st September 2018.

Inclusion criteria were: randomised controlled trials (RCT) and non-randomised studies (non-RCT) if participants were parents of infants receiving neonatal care, interventions were implemented in neonatal units and ≥1 quantitative outcome of parent satisfaction was measured. We included studies from all neonatal care level units and all healthcare settings, without excluding studies in low or middle-income settings. We excluded systematic reviews, entirely qualitative studies, grey literature (e.g. conference abstracts), studies only reporting protocols or abstracts and full reports not in English.

Two authors (SS, IA) independently double-screened titles and abstracts, reviewed full texts for eligibility and resolved any discrepancies with a third reviewer (JW). We extracted data using a pilot-tested, standardised data extraction form including study characteristics, interventions, outcomes and parent input into interventions' design. We assessed methodological quality with the Cochrane Collaboration risk of bias tool (13) for RCT and the ROBINS-I tool (14) for non-RCT.

We presented individual study aggregate data in a narrative synthesis, grouped studies into themes using a Grounded Theory Approach (15) and planned meta-analysis where data were appropriate for quantitative synthesis.

Patient involvement

This review was conceived in response to the clinical need identified by parents with neonatal care experience; a partnership including families with experience of preterm birth identified "what emotional and practical support improves attachment and bonding, and does the provision of such support improve outcomes for premature babies and their families?" as a top 10 research priority (16). Additionally, this review was conceived as part of planning a wider project to pilot a neonatal intervention, with parents' full input. Patients were not directly involved in the design, conduct, reporting or dissemination plans of our research.

RESULTS

We identified 8362 studies for screening and assessed 73 full text articles for eligibility (Figure 1). A total of 32 studies describing interventions to improve parent satisfaction in neonatal care met the inclusion criteria, reporting data from over 2866 parents, 1 study did not report number of parents. Our analysis included 10 RCT and 22 non-RCT: 3 cohort trials, 18 unspecified designs and 1 implementation project. We classified the unspecified non-RCT into 2 types, depending on how they defined their control groups and how they evaluated parent satisfaction (eTable 1).

1. *"Unit- level effect":* Studies that assessed parent satisfaction during a period of routine care (control group) and introduced the intervention at a later time, with a different group of parents. In these studies improvement in parent satisfaction was evaluated between different parent groups, on a *unit level.*
2. *"Group level effect":* Studies that formed intervention and control groups using convenience sampling during the same time period. Both groups (or sometimes only the intervention group) had satisfaction measured after the

Confidential: For Review Only

intervention period (post intervention testing). Baseline parent satisfaction was also measured in both groups (pre intervention testing) in some studies. Improvement in parent satisfaction was demonstrated either by comparing outcomes between intervention/control groups following the intervention, or in comparison with the pre-intervention data.

Parent participants included mothers (14 studies), mothers and fathers (10 studies) or were not specified (7 studies). One study defined parent participants as a dyad of the mother with her designated support person. Median parent sample size was 63, range (7-482). This was higher for RCT (108) compared to non-RCT studies (61). Study participants included parents of babies across the full range of gestations (23-42 weeks). Overall, 24/32 (75%) of studies involved preterm infants, 5/32 (16%) term infants and 7 studies did not state the gestational age of infants involved. Most studies (19, 59%) involved only preterm infants (up to 37 weeks); only 1 study (3%) involved only term infants and 5 studies (16%) involved both preterm and term infants.. Preterm infants were included in 44% of RCT, versus 63% of non-RCT. eTable 1 shows the key characteristics of included studies.

<u>Parent satisfaction</u>

All 32 studies reported they measured parent satisfaction as an *a priori* outcome. Only one study confirmed this through a protocol. Overall 18/32 (56%) of studies (4/10, 40% RCT and 14/22, 64% non-RCT) reported a higher level of parent satisfaction associated with the intervention studied. Multiple different outcome measures within the domain of parent satisfaction were used; we grouped these into 4 categories: i) Parent satisfaction (no additional description); ii) Parent satisfaction

Confidential: For Review Only

with NICU care; iii) Parent satisfaction related to specific components such as communication, staff or information; iv) Parent satisfaction with a specific intervention.

Parent satisfaction was assessed using 32 different methods: 29 different questionnaires, 2 different single questions, and by structured interview in 1 study; in total 334 different questions were used to assess parent satisfaction. Only 6/29 (21%) of questionnaires were reported to be fully validated (both content validation and reliability testing); 23/29 (79%) questionnaires were partially or completely unvalidated. The most commonly used questionnaire was the validated *Neonatal Index of Parent Satisfaction (NIPS)* (17) questionnaire (3 studies).

Parent input into design of interventions

Five studies (5/32, 16%) reported involving parents in intervention design, of which 2 reported improvement of parent satisfaction. The number of included studies was too small to estimate any effect of parent co-design on the success of interventions at study level.

Interventions

We grouped included studies into 5 intervention themes: parent involvement (14 studies); information provision/communication (8 studies); clinical care (7 studies); parent emotional support (2 studies); other (1 study). Parent involvement interventions were more commonly assessed in RCT compared to non-RCT .

Confidential: For Review Only

We categorised interventions *as effective* or *not effective* based upon whether a statistically significant difference between intervention and control groups was reported for parent satisfaction (Table 1). None of the studies reported significantly lower parent satisfaction in the intervention group compared to the control group. We classified studies as *unclear if effective* if they included small sample numbers or if statistical analysis was not performed. Finally, we highlighted studies where *only the intervention group was assessed and only post-intervention,* where comparison to a control group was not possible.

Overall, 18/32 studies (56%) reported higher parent satisfaction in the intervention group; 4/10 RCT and 14/22 non-RCT. The intervention theme where higher satisfaction was most consistently reported was parent involvement (10/14 studies). Due to the large heterogeneity of outcome measure scales a quantitative synthesis and meta-analysis was not possible.

Confidential: For P

**1. Parent involvement**	**Outcome**
More NICU access, parents on WRs, Education (De Bernardo et al, Italy, 2017)	Effective
More NICU access, care involvement, education (Bastani et al, Iran, 2015) **RCT**	Effective
Newborn Individualised Developmental Care and Assessment Program (NIDCAP) (Wielenga et al, Netherlands, 2006)	Effective
Kangaroo care (Legault and Goulet, Canada, 1995)	Effective
Rooming-in care (Kazemian et al, Iran, 2016)	Effective
Single-family NICU rooms (Stevens et al, USA, 2011)	Effective
Parental Presence at Clinical Bedside Rounds (Abdel-Latif et al, Australia, 2015) **RCT**	Effective
Family-centered rounds (Voos et al, USA, 2011)	Effective
Infant Progress Charts filled by parents and 3 Care Planning Meetings (Penticuff and Arheart. USA, 2005)	Effective
Education re: pain management (Franck et al, UK, 2011) **RCT**	Effective

Open Unit policy: 24/7 NICU access (Voos and Park, USA, 2014)	Unclear if effective
Touch and massage for 7 days (Livingston et al, USA, 2009) **RCT**	Unclear if effective
a. Massage with auditory, tactile, visual, and vestibular stimulation<br>b. Kangaroo care (Holditch-Davis et al, USA, 2013) **RCT**	Not effective
Individualised, developmentally supportive family-centered care interventions (Byers et al, USA, 2006)	Not effective

**2. Information provision / communication**	**Outcome**
Internet-based education (Kadivar et al, Iran, 2017)	Effective
Daily SMS from Electronic Patient Record (Globus et al, Israel, 2016)	Effective
Staff education, staff contact card given to parents, staff poster at NICU reception (Weiss et al, USA, 2010)	Effective
Provision of taped conversations with neonatologists to mothers (Koh et al, Australia, 2007) **RCT**	Effective
Clinical staff enter updates in baby diary (Van de Vijver and Evans, UK, 2015)	Unclear if effective
Detailed information provided during consenting (Broyles et al, USA, 1992) **RCT**	Unclear if effective
Sharing information obtained from parent interviews with the primary NICU provider (Clarke-Pounder et al, USA, 2015) **RCT**	Not effective
Daily parent update letter from Electronic Patient Record (Palma et al, USA, 2012)	Only the intervention group was assessed and only post-intervention

**3. Clinical care**	**Outcome**
a. Headbox oxygen for respiratory distress<br>b. CPAP for respiratory distress (Foster et al, Australia, 2008)	Effective
Co-bedding infants in incubators (prospective) (Byers et al, USA, 2003)	Effective
Co-bedding infants in incubators (retrospective) (Polizzi et al, USA, 2003)	Effective
Palliative care (Petteys et al, USA, 2015)	Unclear if effective
Five potentially better practices in the area of discharge planning (Mills et al, USA, 2006)	Unclear if effective
Clinical Nurse Specialist/ neonatal practitioner team care (Mitchell-DiCenso et al, Canada, 1996) **RCT**	Not effective

**4. Parent emotional support**	**Outcome**
Narrative writing (Kadivar et al, Iran, 2017)	Effective
Listening visits (Segre et al, USA, 2013)	Only the intervention group was assessed and only post-intervention
Tele-rounding robot, off-site neonatologist (Garingo et al, USA, 2016)	Only the intervention group was assessed and only post-intervention

**5. Other**	**Outcome**
Free Parking (Northrup et al, USA, 2016) **RCT**	Not effective

Table 1. Interventions in themes

Legend: *The colours illustrate each intervention's reported effect on parent satisfaction.* *Green (intervention effective): Parent satisfaction was reported to be statistically significantly higher in the intervention group;* *Red (intervention not effective): Parent satisfaction was not reported to be statistically significantly different in the intervention group; Yellow (unclear if effective): Small study numbers and/or no statistical analysis performed); Grey (Only the intervention group was assessed and only post-intervention).* ***RCT:*** *Randomised Controlled Trial*

Methodological quality

For the majority of RCT, key study characteristics, such as randomisation, allocation concealment and blinding of outcome assessment, were either not stated or unclear (Figure 2). Only one RCT had an available study protocol (retrospectively registered)

For peer review only

and none described blinding of study participants and/or personnel. All RCT scored a high/unclear risk of bias in at least 4/6 Cochrane tool categories, except for one, which scored a high/unclear risk in 3/6 categories.

We assessed 21/22 non-RCT studies using the ROBINS-I tool (14), excluding the implementation project. All 21 studies were assessed as having an overall *serious* risk of bias and 7/21 of studies (33%) were further categorised as having *critical* risk of bias (Figure 3). Blinding of participants, personnel and outcome assessment was poorly reported across all non-RCT and no study reported a published study protocol. None of the included non-RCT measured or corrected for important parent/infant confounding variables, or other relevant neonatal unit co-interventions taking place at the same time as the intervention.

We were unable to use the *Standards for Reporting Implementation Studies (StaRI) Statement Tool* (18) for assessing the implementation project, as the reporting was incomplete.

There was no association between methodological quality assessments and the studies' reported effect on parent satisfaction. All 4/10 RCT that reported a higher level of parent satisfaction associated with their intervention, scored a high/unclear risk of bias in at least 4/6 Cochrane tool categories, one of which scored high/unclear *risk* in all categories. Out of the 14/22 non-RCT reporting an improved parent satisfaction, two were deemed to be at *critical risk* of bias on the ROBINS- I tool, whilst the rest we assessed to be at *serious risk* of bias.

https://mc.manuscriptcentral.com/bmjpo

DISCUSSION

Parent satisfaction with neonatal care is increasingly recognised as an important measure of parent experience and is being used to evaluate hospitals and healthcare providers; use of interventions to improve parent satisfaction in neonatal units is increasing. This is the largest review of interventions where an outcome was parent satisfaction with neonatal care and includes 32 studies. We find low quality evidence that interventions targeting parent involvement may improve parent satisfaction with neonatal care, but this result must be interpreted cautiously in view of the high risk of bias in included studies.

A further reason for only selecting parent satisfaction as the outcome of interest was to focus on a single component of parent experience, in order to reduce outcome heterogeneity and allow direct comparison. Despite this approach, the key methodological limitation identified in this review was inconsistency in how parent satisfaction is defined and measured; it is notable that the majority of questionnaires (23/29) lack validation. In keeping with neonatal studies more widely (19), this study confirms inconsistent outcome selection as a major source of research waste in neonatal studies examining parent experience, and further finds that there is limited involvement of parents in study design.

Strengths of our review include identifying studies with both mothers and fathers as participants, inclusion of the full range of infant gestations and a wide range of interventions. We followed a pre-registered protocol and report this review in line with PRISMA guidelines (12). To aid direct comparison of interventions, we only

Confidential: For Review Only

included studies that evaluated parent experience using at least the outcome of parent satisfaction. One limitation of this approach is that by excluding studies which evaluated parent experience using other measures (e.g. stress, anxiety and depressions scales) we are unable to comment on interventions that targeted these other components of parent experience.

Brett et al (10) systematically reviewed interventions aimed at improving the parent experience more widely, but only included parents of preterm infants. The large number of outcome domains and heterogeneity of outcome measures included in this study meant that the authors we unable to draw firm conclusions about the efficacy of interventions and that meta-analysis was not possible. The majority of our review's studies have been published in the 7 years since the Brett review, highlighting the increasing interest in this area. However, despite including all gestations and focusing on a specific aspect of parent experience, heterogeneity in measurement of parent satisfaction meant we were also unable to conduct a quantitative synthesis. Inconsistency and lack of validation of instruments measuring parent satisfaction in neonatal care (specifically with family-centred care) has previously been highlighted by Dall'Oglio et al (20).

Although 31% of included studies were RCT, all were assessed as having a high risk of bias. Randomised controlled trials are traditionally considered the highest-ranking form of evidence, however it is worth considering whether such a design is feasible or desirable to evaluate interventions targeting parent satisfaction. Parents in neonatal care talk to each other, compare notes and invariably create parent-support communities; hence it is inherently difficult to avoid contamination between parents

Confidential: For Review Only

receiving an intervention and those who are not, meaning that blinding of parents or health professionals is near impossible. Furthermore, parent satisfaction is likely to be particularly susceptible to the Hawthorne effect (21), requiring longer-term follow up. These factors may explain the low number of RCT identified in our review and the high risk of bias seen in those that were included. In non-RCT studies, the main methodological concern is the degree to which unmeasured and uncontrolled confounders may explain any differences seen between groups. The non-RCT studies included in this review were classed as having either a serious or critical risk of bias. The overwhelming majority of studies did not adequately report baseline variables or report other interventions during the study period, making it impossible to assess studies for selection bias or treatment bias. Furthermore, limitations such as contamination bias and the Hawthorne effect affect non-RCT as well. Only two non-RCT studies evaluated the outcome of interest (parent satisfaction) both before and after the intervention, in the same group of parents *(group level effect),* with most studies evaluating different parent groups pre and post intervention *(unit level effect).* An inherent weakness of this latter approach is that it assumes parent satisfaction is a static measure at the unit level, which is unlikely to be true. As a result of these numerous important limitations identified across all included studies, we find only low-quality evidence in support of interventions to improve parent satisfaction with neonatal care, despite a majority of studies reporting a beneficial effect of interventions. These limitations may explain the limited uptake of these interventions by the wider neonatal community.

Changing neonatal unit practices to incorporate any new intervention requires robust evidence. We demonstrate here that such evidence is not currently available for

https://mc.manuscriptcentral.com/bmjpo

Confidential: For Review Only

improving parent satisfaction. We highlight the use of non-randomised study designs, inconsistency in definition and measurement of parent satisfaction, the use of unvalidated questionnaires, methodological limitations and a lack of parent involvement as contributors. Given the importance of parent satisfaction for both parent and offspring wellbeing, higher quality trials that involve parents, use standardised definitions and validated parent satisfaction measures are needed. Given the nature and challenges of the neonatal care environment and the limitations we have identified in existing research, a cluster trial may be the most appropriate study design to rigorously evaluate interventions to improve parent satisfaction with neonatal care.

CONCLUSIONS

Many interventions, commonly relating to parent involvement, are reported to improve parent satisfaction with neonatal care but inconsistency in definition and measurement of parent satisfaction and high risk of bias in all studies makes this low quality evidence. Standardised definitions and validated parent satisfaction measures are needed, as well as higher quality trials of parent experience, involving parents in intervention design.

What is already known on this topic

- Neonatal care significantly affects parents' mental health; parent satisfaction is increasingly being used as a parent experience measure
- Parent satisfaction is inversely related to parent stress; interventions improving parent satisfaction have the potential to reduce parent stress, improve parent-infant bonding and infant outcomes

https://mc.manuscriptcentral.com/bmjpo

Confidential: For Review Only

- Use of interventions to improve parent satisfaction in neonatal units is increasing, though few are formally evaluated and wider uptake is limited; it is not known the degree to which parents are involved in intervention design

What this study adds

- There is inconsistency in how parent satisfaction in neonatal care is defined and measured, and the majority of studies do not include parents in intervention design
- There is low quality evidence that interventions relating to parent involvement may improve parent satisfaction with neonatal care
- Standardised, validated measures of parent satisfaction and higher quality trials, involving parents in intervention design, are needed

DECLARATIONS

Conflict of interest disclosure

SS has received research grants from the National Institute of Health Research (NIHR), the NIHR CLAHRC NWL, Rosetrees Trust and CW+ charity. NM is Director of the Neonatal Data Analysis Unit at Imperial College London. In the last five years NM has served on the Board of Trustees of the Royal College of Paediatrics and Child Health, David Harvey Trust, Medical Women's Federation and Medact; and is a member of the Nestle Scientific Advisory Board. NM has received research grants from the British Heart Foundation, Medical Research Council, National Institute of Health Research, Westminster Research Fund, Collaboration for Leadership in Applied Health and Care Northwest London,

Confidential: For Review Only

Healthcare Quality Improvement Partnership, Bliss, Prolacta Life Sciences, Chiesi, Shire and HCA International; travel and accommodation expenses from, Nutricia, Prolacta, Nestle and Chiesi; honoraria from Ferring Pharmaceuticals and Alexion Pharmaceuticals for contributions to expert advisory boards, and Chiesi for contributing to a lecture programme. CG is funded by the United Kingdom Medical Research Council (MRC) through a Clinician Scientist Fellowship award. He has received support from Chiesi Pharmaceuticals to attend an educational conference; in the past 5 years he has been investigator on received research grants from Medical Research Council, National Institute of Health Research, Canadian Institute of Health Research, Department of Health in England, Mason Medical Research Foundation, Westminster Medical School Research Trust and Chiesi Pharmaceuticals. IA, JW, DB: None to declare.

Authors' contributions

SS and CG conceived this systematic review. The protocol was created by SS and CG. Searches were performed by SS and IA. All search results were reviewed by SS, and JW. Coding was completed by SS and JW. Data analysis was completed by SS. The first draft of the manuscript was written by SS; SS, CG and JW edited and reviewed the manuscript. All authors approved the manuscript. This article presents independent research supported by the National Institute for Health Research (NIHR) The views expressed in this publication are those of the authors and not necessarily those of the NHS, the NIHR or the Department of Health and Social Care.

Funding

This work is sponsored by Imperial College London and supported by a peer-

https://mc.manuscriptcentral.com/bmjpo

reviewed National Institute of Health Research Doctoral Research Fellowship, awarded to SS (DRF-2017-10-172).

References

1. Neonatal Data Analysis Unit. Neonatal Data Analysis Unit Annual Report 2015. Online. 2016: https://www1.imperial.ac.uk/resources/69CED33F-CF8D-4727-BC05-94A885B8699/ndau2015reportv1.4.pdf

2. Lefkowitz DS, Baxt C, Evans JR. Prevalence and Correlates of Posttraumatic Stress and Postpartum Depression in Parents of Infants in the Neonatal Intensive Care Unit (NICU). *J Clin Psychol Med S.* 2010;17(3):230-7.

3. Shaw RJ, Bernard RS, DeBlois T, Ikuta LM, Ginzburg K, Koopman C. The Relationship Between Acute Stress Disorder and Posttraumatic Stress Disorder in the Neonatal Intensive Care Unit. *Psychosomatics.* 2009;50(2):131-7.

4. Beck CT, Woynar J. Posttraumatic Stress in Mothers While Their Preterm Infants Are in the Newborn Intensive Care Unit: A Mixed Research Synthesis. *ANS Adv Nurs Sci.* 2017;40(4):337-55.

5. Lee SK, O'Brien K. INNOVATIONS Parents as primary caregivers in the neonatal intensive care unit. *Can Med Assoc J.* 2014;186(11):845-7.

6. Grace SL, Evindar A, Stewart DE. The effect of postpartum depression on child cognitive development and behavior: a review and critical analysis of the literature. *Arch Womens Ment Health.* 2003;6(4):263-74.

7. Rocha G, Candeias L, Ramos M, Maia T, Guimaraes H, Viana V. [Stress and satisfaction of mothers in neonatal intensive care]. *Acta Med Port.* 2011;24 Suppl 2:157-66.

Confidential: For Review Only

8. Lopez-Maestro et al. Quality of attachment in infants less than 1500g or less than 32 weeks. Related factors. *Early Hum Dev.* 2016;104:1-6.

9. Charpak N, Tessier R, Ruiz JG, Hernandez JT, Uriza F, Villegas J, et al. Twenty-year Follow-up of Kangaroo Mother Care Versus Traditional Care. *Pediatrics.* 2017;139(1).

10. Brett J, Staniszewska S, Newburn M, Jones N, Taylor L. A systematic mapping review of effective interventions for communicating with, supporting and providing information to parents of preterm infants. *BMJ Open.* 2011;1(1):e000023.

11. PROSPERO database. Available from: http://www.crd.york.ac.uk/prospero/display_record.asp?ID=CRD42016042110

12. Moher D, Liberati A, Tetzlaff J, Altman DG. Preferred reporting items for systematic reviews and meta-analyses: the PRISMA statement. *Annals of internal medicine.* 2009;151(4):264-9, W64. Epub 2009/07/23.

13. Higgins Julian PT, Altman Douglas G, Gotzsche Peter C, Juni Peter, Moher David, Oxman Andrew D et al. The Cochrane collaboration's tool for assessing risk of bias in randomised trials. *BMJ.* 2011;343:d5928.

14. Sterne Jonathan AC, Hernan Miguel A, Reeves Barnaby C, Savovic Jelena, Berkman Nancy D, Viswanathan Meera et al. ROBINS-I: a tool for assessing risk of bias in non-randomised studies of interventions *BMJ.* 2016; 355:i4919.

15. Green J TN. Qualitative Methods for Health Research. London, SAGE. 2014(3rd edition):SAGE.

16. Duley L Uhm S, Oliver S et al. Top 15 UK Research Priorities for Preterm Birth. The Lancet. 2014;383(9934):2041-2042.

17. Mitchell-DiCenso A, Guyatt G, Paes B, Blatz S, Kirpalani H, Fryers M et al.

Confidential: For Review Only

A new measure of parent satisfaction with medical care provided in the neonatal intensive care unit. *Journal of Clinical Epidemiology.* 1996;49(3):313-318.

18. Pinnock H, Barwick M, Carpenter C, Eldridge S, Grandes G, Griffiths CJ et al for the StaRI Group. Standards for Reporting Implementation Studies (StaRI) statement. *BMJ*. 2017;356:i6795.

19. Webbe JWH, Ali S, Sakonidou S, Webbe T, Duffy JMN, Brunton G et al, Inconsistent outcome reporting in large neonatal trials: a systematic review, *Archives of Disease in Childhood - Fetal and Neonatal Edition.* Published Online First: 13 May 2019; doi: 10.1136/archdischild-2019-316823

20. Dall'Oglio, I, Mascolo, R, Gawronski, O, Tiozzo, E, Portanova, A, Ragni, A, et al. A systematic review of instruments for assessing parent satisfaction with family-centred care in neonatal intensive care units. *Acta Paediatr.* 2018;107:391-402.

21. McCambridge J, Witton J, Elbourne DR. Systematic review of the Hawthorne effect: new concepts are needed to study research participation effects. *J Clin Epidemiol*. 2014;67(3):267–277.

22. Abdel-Latif ME, Boswell D, Broom M, Smith J, Davis D. Parental presence on neonatal intensive care unit clinical bedside rounds: randomised trial and focus group discussion. *Arch Dis Child Fetal Neonatal Ed*. 2015;100:F203–9.

23. K.F. Pridham, A.S. Chang, What being the parent of a new baby is like: revision of an instrument, *Res. Nurs. Health* 12;1989:323–329.

24. York Hospital NICU Discharge Survey http://www.nann.org/pdf/09discharge

25. Conner JM, Nelson EC. Neonatal intensive care: satisfaction measured from a parent's perspective. *Pediatrics.* 1999;103:336.

Confidential: For Review Only

26. Department of Health, Toolkit for High-Quality Neonatal Services 2009, Principle 3: Care of the baby and family experience.

27. NICE, Quality standard for specialist neonatal care 2010, Quality Statement No 5.

28. Burger SA, King J, Tallett A. "Parents' experiences of neonatal care in England". *Patient Experience Journal.* 2015;2(2):7.

29. Press Ganey Associates, I. (2004). NICU survey: Product description and psychometrics. South Bend, IN: Author.

30. McPherson ML, Sachdeva RC, Jefferson LS. Development of a survey to measure parent satisfaction in a pediatric intensive care unit. *Crit Care Med.* 2000;28: 3009–3013.

31. Jenkinson C, Coulter A, Bruster S. The Picker Patient Experience Questionnaire: development and validation using data from in-patient surveys in five countries. *Int J Qual Health Care.* 2002;14:353–358.

32. Crofton C, Darby C, Farquhar M, Clancy CM. The CAHPS hospital survey: development, testing, and use. *Jt Comm J Qual Patient Saf.* 2005;31:655–659.

33. Miceli PJ. Validating a patient satisfaction survey translated into Spanish. *J Healthc Qua.l* 2004;26:4–13.

34. Conner JM, Nelson EC. Neonatal intensive care: satisfaction measured from a parent's perspective. *Pediatrics.* 1999;103(1,suppl):336–349.

35. Baggs JG. Collaborative interdisciplinary bioethical decision making in intensive care units. *Nurs Out- look.* 1993;41(3):108–112.

36. Little, GA and Edwards WH. Advancing family-centered care as a quality improvement initiative. 2000. *Proceedings of the Physical and Developmental Environment of the High-Risk Infant Conference.*

Confidential: For Review Only

37. Affonso DD, Wahlberg V., Persson B. Exploration of mothers' reactions to the kangaroo method of prematurity care. *Neonatal Network. 1989;*7(6), 43-51.

Figure / Table Legends

Figure 1: PRISMA Flow diagram of selected studies

Figure 2. Cochrane Collaboration Risk of Bias tool assessment (RCT)

Legend: Green- low risk of bias; Yellow- unclear risk of bias; Red- high risk of bias

Figure 3. ROBINS-I risk of bias assessment (Non-RCT)

Table 1. Interventions in themes

Legend: *The colours illustrate each intervention's reported effect on parent satisfaction.* *Green (intervention effective): Parent satisfaction was reported to be statistically significantly higher in the intervention group;* *Red (intervention not effective): Parent satisfaction was not reported to be statistically significantly different in the intervention group; Yellow (unclear if effective): Small study numbers and/or no statistical analysis performed); Grey (Only the intervention group was assessed and only post-intervention).* ***RCT:*** *Randomised Controlled Trial*

Online supplementary files

eTable 1. Included studies by study design: Randomised controlled trials (RCT) and non-RCT

Legend: *Number in last column illustrates each intervention's reported effect on parent satisfaction: 1. Parent satisfaction was statistically significantly higher in the intervention group; 2. Parent satisfaction was not reported to be statistically*

Confidential: For Review Only

significantly different in the intervention group; 3. Unclear if parent satisfaction improved (small study numbers and/or no statistical analysis performed); 4. Only the intervention group was assessed and only post-intervention

File 1. OVID MEDLINE search strategy

Research checklist

PRISMA checklist

Confidential: For Review Only

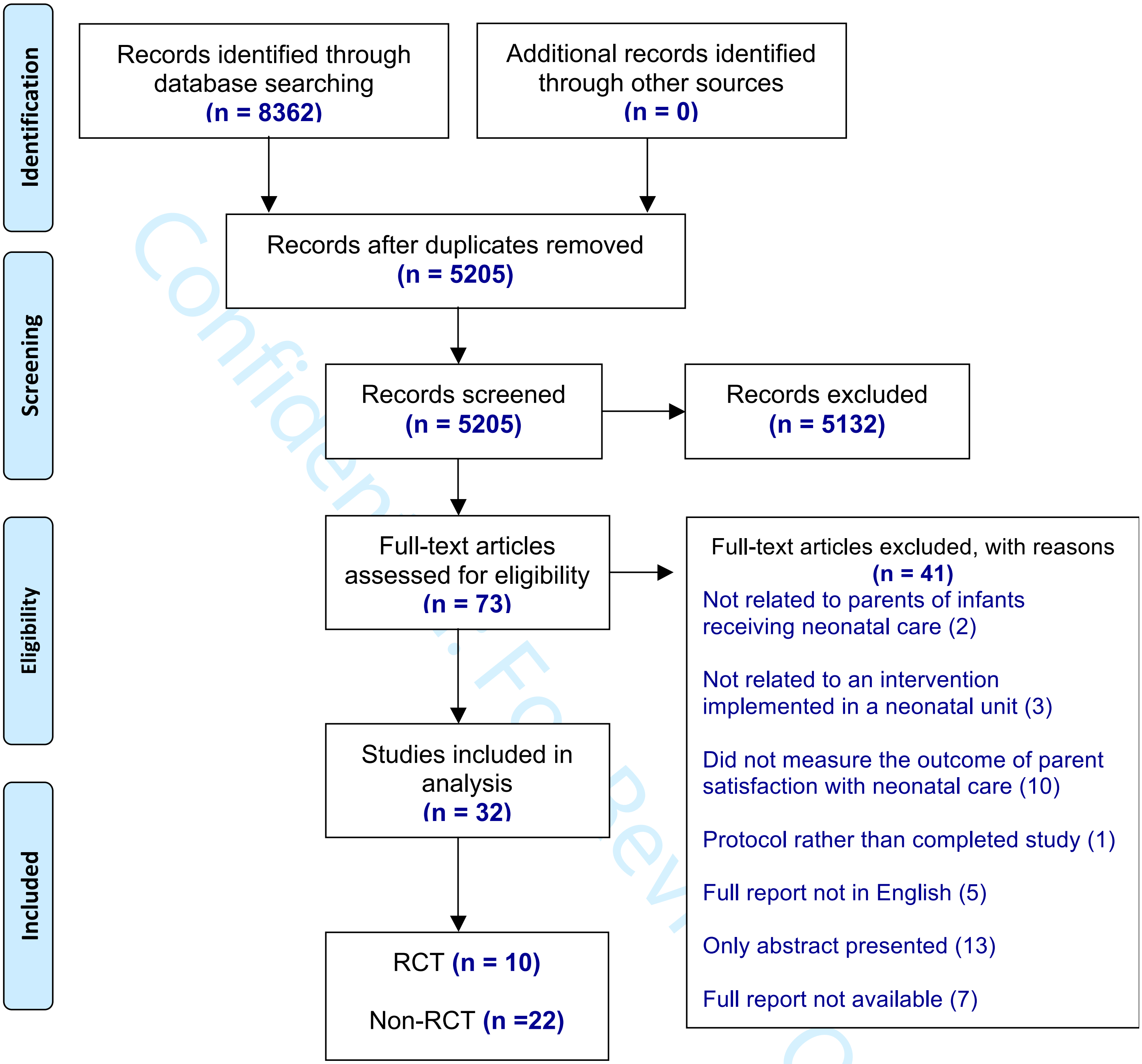

Confidential: For Review Only

Risk of Bias (Cochrane)

Author by publication year	Random sequence generation	Allocation concealment	Blinding of participants and personnel	Blinding of outcome assessment	Incomplete outcome data	Selective reporting
1. Northrup (2016)	?	+	-	?	+	?
2. Abdel-Latif (2015)	+	+	-	-	-	?
3. Bastani (2015)	?	?	-	?	+	?
4. Clarke-Pounder (2015)	?	?	-	?	+	?
5. Holditch-Davis (2013)	+	+	-	+	?	?
6. Franck (2011)	-	?	-	?	-	+
7. Livingston (2009)	?	?	-	?	+	?
8. Koh (2007)	?	?	-	?	?	?
9. Mitchell-DiCenso (1996)	+	?	?	?	?	?
10. Broyles (1992)	?	?	-	+	+	?

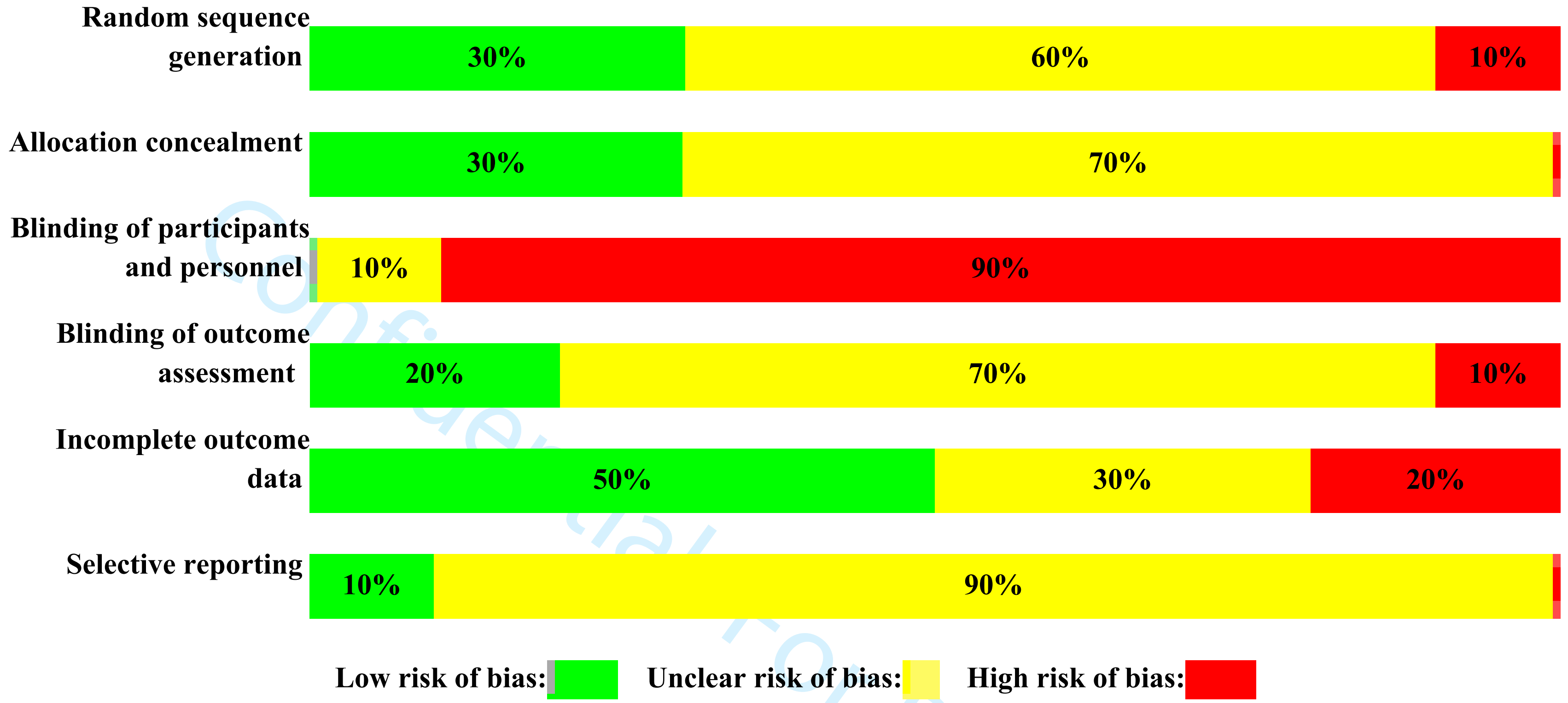

Confidential: For Review Only

Risk of Bias (ROBINS-I)

Author by publication year	Bias due to confounding	Bias in selection of participants into the study	Bias in classification of interventions	Bias due to deviations from intended interventions	Bias due to missing data	Bias in measurement of outcomes	Bias in selection of the reported result	OVERALL risk of bias
1. De Bernardo (2017)	SERIOUS	LOW	LOW	SERIOUS	LOW	SERIOUS	SERIOUS	SERIOUS
2. Kadivar (2017) *Internet-based education*	SERIOUS	LOW	LOW	SERIOUS	LOW	SERIOUS	MODERATE	SERIOUS
3. Kadivar (2017) *Narrative writing*	SERIOUS	SERIOUS	LOW	SERIOUS	LOW	SERIOUS	MODERATE	SERIOUS
4. Garingo (2016)	CRITICAL	LOW	LOW	SERIOUS	SERIOUS	SERIOUS	SERIOUS	CRITICAL
5. Globus (2016)	SERIOUS	LOW	LOW	NO INFO	SERIOUS	SERIOUS	SERIOUS	SERIOUS
6. Kazemian (2016)	SERIOUS	NO INFO	LOW	SERIOUS	NO INFO	SERIOUS	SERIOUS	SERIOUS
7. Petteys (2015)	SERIOUS	LOW	LOW	SERIOUS	MODERATE	SERIOUS	MODERATE	SERIOUS
8. Van de Vijver (2015)	CRITICAL	LOW	LOW	SERIOUS	MODERATE	SERIOUS	MODERATE	CRITICAL
9. Voos (2013)	CRITICAL	LOW	LOW	SERIOUS	NO INFO	SERIOUS	SERIOUS	CRITICAL
10. Segre (2013)	CRITICAL	NO INFO	LOW	SERIOUS	SERIOUS	SERIOUS	MODERATE	CRITICAL
11. Palma (2012)	CRITICAL	NO INFO	LOW	SERIOUS	SERIOUS	SERIOUS	CRITICAL	CRITICAL
12. Stevens (2011)	SERIOUS	LOW	LOW	SERIOUS	SERIOUS	SERIOUS	MODERATE	SERIOUS
13.Voos (2011)	SERIOUS	LOW	LOW	SERIOUS	SERIOUS	SERIOUS	MODERATE	SERIOUS
14. Weiss (2010)	SERIOUS	LOW	LOW	SERIOUS	LOW	SERIOUS	MODERATE	SERIOUS
15. Foster (2008)	SERIOUS	CRITICAL	LOW	SERIOUS	SERIOUS	SERIOUS	MODERATE	CRITICAL
16. Byers (2006)	SERIOUS	LOW	LOW	SERIOUS	SERIOUS	SERIOUS	MODERATE	SERIOUS
18. Wielenga (2006)	SERIOUS	LOW	LOW	SERIOUS	LOW	SERIOUS	MODERATE	SERIOUS
19. Penticuff (2005)	SERIOUS	LOW	LOW	SERIOUS	LOW	SERIOUS	MODERATE	SERIOUS
20. Byers (2003)	SERIOUS	LOW	LOW	SERIOUS	LOW	SERIOUS	SERIOUS	SERIOUS
21. Polizzi (2003)	SERIOUS	MODERATE	LOW	SERIOUS	SERIOUS	SERIOUS	MODERATE	SERIOUS
22. Legault (1995)	SERIOUS	CRITICAL	LOW	CRITICAL	LOW	SERIOUS	MODERATE	CRITICAL

Randomised controlled trials (RCT) by publication year

Author (Date), Country	Parents' gender/ Total sample Size	Infants' Gestational age (GA) in weeks	Study design	Intervention	Outcome measures	Timing of measurement	Method of measurement	Results	Was the intervention co-designed with parents	Improved parent satisfaction?
1. Northrup et al. (2016), USA	Mothers and fathers /116	<28	Randomised controlled trial	Intervention: **Free Parking (FP).**<br><br>Parents received seven parking vouchers at a time (value: $10/each) from the hospital's research office and continued to receive vouchers until infant discharge. Each voucher allowed free entry and exit for a 24-h period (including re-entry).<br><br>Control: Parents received the standard care and did not receive vouchers.	*Parent satisfaction with NICU care*	After babies were discharged (once)<br><br>- During the first high-risk-infant clinic visit after discharge<br><br>No pre-intervention parent satisfaction data available for comparison.	*Satisfaction questionnaire*<br><br>Validation: No content validity or reliability testing reported.<br><br>11 questions<br><br>- Seven items were summed (score 7-35) to measure "Support" (e.g., information sharing).<br><br>- Three items measured "Emotional Connection" to the infant (score 3-15)<br><br>- One item assessed "family involvement in infant care" (responses: not enough-just right-too much).<br><br>Greater scores indicated higher perceived support, connection and satisfaction.	**The groups did not differ significantly with respect to satisfaction.**<br><br>**Mean (SD)** — **Intervention / Control / p-value**<br><br>NICU support: 30 (2.7) / 28.7 (3.7) / 0.07<br><br>Emotional connection: 12.3 (1.7) / 12.3 (1.7) / 0.96<br><br>Family involvement "Just right": 81.4% / 85% / 0.07	**No**	**2**
2. Abdel-Latif et al. (2015), Australia	Mothers and fathers /63	25-42	Cross-over Randomised Controlled Trial	Intervention: **Parental Presence at Clinical Bedside Rounds (PPCBR).**<br><br>Parents attended bedside clinical rounds. Parents had opportunity to ask questions about their baby's condition and management.<br><br>Control: Parents received the standard care with no parental presence at bedside clinical rounds.	*Parent satisfaction as assessed by questions of 3 domains:*<br>*1. Knowledge and understanding*<br>*2. Communication and collaboration*<br>*3. Privacy and confidentiality*	During babies' admission (once)<br><br>- At the end of each study arm, separated by a washout period<br><br>- No pre-intervention parent satisfaction data available for comparison	*Satisfaction questionnaire*<br><br>The authors stated "the research team designed the questionnaire".<br><br>Validation: No content validity or reliability testing reported.<br><br>Number and format of questions: not stated	**PPCBR had significantly higher adjusted mean (95% CI) scores for some questions from domains 1 and 2.**<br><br>Domain 3 was comparable between the two study groups.<br>**Intervention / Control / p-value**<br>Domain 1 question:<br>*"I have received adequate information about my baby's condition and management"*<br>**Mean satisfaction** 4.321 / 3.947 / 0.03<br><br>Domain 2 questions:<br>*"In the last week I have been able to communicate effectively with my baby's healthcare team"*<br>**Mean satisfaction** 4.407 / 4.250 / 0.05<br><br>*"In the last week I have collaborated with my baby's healthcare team in the planning of care for my baby"*<br>**Mean satisfaction** 3.843 / 3.426 / 0.02<br><br>*"In the last week I have been able to ask the healthcare team questions about my baby's care"*<br>**Mean satisfaction** 4.642 / 4.259 / 0.004	**No**	**1**
3. Bastani et al, (2015), Iran	Mothers /100	30-37 Mean (SD)<br><br>Control 33.90 (2.33)	Randomised Controlled Trial (block randomization)	Intervention: **Family-centered Care (FCC).**<br><br>Mothers were allowed access to their baby at any time, participated in the	*Maternal satisfaction* relating to three themes:<br>1. Parental presence<br>2. Participation in	During babies' admission (twice)<br><br>- 24 hours after admission	*Satisfaction questionnaire* (Validated)<br><br>A modified satisfaction questionnaire was used,	**In the FCC group, pre and post intervention difference in maternal satisfaction was statistically significant p<0.001**<br><br>**Satisfaction / Intervention / Control / p-value**	**Unclear**<br><br>Mothers determined the reliability of	**1**

Confidential: For Review Only

		Intervention<br>34 (1.9)		care process and were provided with information about neonatal care.<br><br><u>Control:</u> Mothers received the standard care where they were only allowed to be present at the time of the infant's entry to the neonatal care unit, and were only routinely informed.	neonatal care<br>3. Information about neonatal care	- At the time of discharge	based on a parental satisfaction instrument developed for measuring satisfaction in Paediatric intensive care Units (PICU).<br><br>18 questions<br><br>Graded 0 (very dissatisfied) to 4 (very satisfied).<br><br>The overall satisfaction rate was classified based on the mean scores (score<50%, between 75-50% and > 75%).	**Mean (SD)**<br>**At 24 hours** 22.36 (8.90) 22.06 (9.77) 0.87<br>**At discharge** 59.28 (6.86) 30.18 (14.09) <0.01	the satisfaction tool and approved the educational pamphlet. Authors did not report if mothers had direct input in the intervention's design.	
4. Clarke-Pounder et al. (2015), USA	Mothers and fathers /19 families	23-39	Randomised Controlled Trial	<u>Intervention:</u> **Sharing information obtained from parent interviews with the primary NICU provider.**<br><br>Parents were interviewed using the *NICU- adapted Decision Making Tool (N-DMT).* Information obtained was placed in the electronic medical record (EMR) and communicated to the primary neonatal provider via email. Daily rounds on all infants were audio-recorded for 3 consecutive days after enrollment to see if information from the N-DMT was incorporated into daily care planning.<br><br><u>Control:</u> The content of a recent social work note was communicated with the primary provider via e-mail, creating an attentional control group.	*Parent satisfaction with care*	During babies' admission (once)<br><br>- 2 weeks after study entry<br><br>No pre-intervention parent satisfaction data available for comparison.	*Satisfaction questionnaire*<br><br>A *NICU- adapted Decision Making Tool (N-DMT)* – specific questionnaire was used.<br><br><u>Validation:</u> Partially reported. Authors stated reliability testing took place; no information on content validity provided.<br><br>8 questions: e.g.''My baby's doctors considered my goals and hopes for my baby during decision-making''.<br><br>Likert scale (1 strongly agree-4 strongly disagree). Total N-DMT score range 8–32.	**There was no significant difference in satisfaction with care as measured by the N-DMT scale between the control group and intervention groups in a univariable model or multiple variable model controlling for gestational age.**<br><br>**Satisfaction Intervention Control**<br>**Median (range)** 26 (15–28) 28.8 (19–32)<br><br>**No p-value reported**<br><br>There was, however, a pattern of decreased satisfaction with care among the intervention group compared to the control group across the N-DMT-specific survey questions, although the differences were not statistically significant.	**Yes**<br><br>Information obtained from parents using the N-DMT was placed in the electronic medical record (EMR) and communicated to the primary NICU provider via email (forming the intervention)	**2**
5.Holditch-Davis et al. (2013), USA	Mothers /208	Preterm infants<br><br>Mean (SD)<br><br>Overall group 27.2 (3.0)	Randomised controlled trial<br><br>3 groups (2 intervention and 1 control)<br><br>Post-intervention testing only.	<u>Interventions:</u> **1. Mothers were taught how to massage infants with auditory, tactile, visual, and vestibular stimulation (ATVV intervention)**<br>**2. Kangaroo care**<br><br><u>Control:</u> Attention control group. Mothers spent a similar amount of time with the study nurse discussing the equipment needed for preterm infant care at home. Study nurses provided education and support for all three groups. Mothers were not prevented from engaging in interventions of the other groups but did not receive formal education from the study nurse on the other interventions.	*1. Parent (mother) satisfaction with the intervention*<br><br>*2. Satisfaction with the helpfulness of the study nurse*<br><br>*3. Whether the mother would recommend the study to others and the degree of change in the mother as a person and as a mother as a result of being in the study.*	During admission period and post discharge<br><br>- At the time of discharge<br><br>- At 2 months corrected age<br><br>No pre-intervention parent satisfaction data available for comparison.	*Satisfaction questionnaire*<br><br>The questionnaire was designed by the study team.<br><br><u>Validation:</u> Partially reported. Authors stated reliability testing took place; no information on content validity provided.<br><br><u>26 questions:</u> relating to three dimensions of satisfaction: efficacy, caring, and technical quality.<br><br>Likert (1 least satisfied-5, 5 most satisfied)	**No significant differences occurred between the groups.**<br><br>Mothers in all three groups were satisfied with the intervention (mean scores of 3.3 or higher on a 5-point scale) and the helpfulness of the nurse (mean scores of 4.6 or higher on a 5-point scale).	**No**	**2**
6. Franck et al. (2011), UK	Mothers and fathers /169	Mean (SD)<br><br>Control:<br>31.94 (5.17)<br><br>Intervention:<br>29.40 (3.17)	Randomised Controlled Trial	<u>Intervention:</u> **Increasing parental involvement in infant pain management in the NICU.**<br><br>Parents received a booklet providing evidence-based information about pain and comforting infants in the NICU setting. Parents received 2 visits from a research nurse showing them	At baseline:<br><br>*1. Parent satisfaction with NICU care*<br><br>One week after the intervention:<br><br>*1. Satisfaction with*	During babies' admission (twice)<br><br>-At baseline (within 3 to 7 days of admission)<br><br>- 1 week after the	*Individual questions*<br><br><u>Validation:</u> No content validity or reliability testing reported.<br><br>1. At baseline:<br><br>Parent satisfaction was	**At baseline: there was no significant difference in satisfaction between intervention and control group**<br><br>**Satisfaction Intervention Control p-value**<br>**Mean (SD):** 1.45 (0.71) 1.51 (0.76) missing<br><br>**1 week after the intervention: Intervention**	**Yes**<br><br>The booklet was reviewed by 12 parents of infants who had been cared for in NICUs in the United	**1**

Confidential: For Review Only

				how to apply the comforting techniques described in the booklet.<br><br><u>Control:</u> As part of usual care, parents in both the intervention and control groups received a detailed booklet with generic information about NICU care. Parents in the control group also received 2 visits from a research nurse listening to what parents had to say about their NICU experience (attention placebo).	*information about pain control*<br><br>*2. Satisfied nurses make infant comfortable*<br><br>*3. Satisfied pain medicines help infant*	intervention	measured by 1 question: *"Satisfaction with NICU care"* (1 very satisfied-6 very unsatisfied) as part of the baseline parent characteristics questionnaire.<br><br>2. One week after the intervention:<br><br>Three questions using the word "satisfied' were selected from the validated *Parent Attitudes About Infant Nociception (PAIN)* survey (Likert scale 1 very satisfied-6 very unsatisfied)	**parents were more satisfied with the information about pain control received than control parents.**<br><br>**Satisfaction Mean (SD):** Intervention 2.10 (0.97), Control 3.28 (1.27), p-value < 0.001	Kingdom.	
7.Livingston et al. (2009), USA	Mothers /12	Mean (SD)<br><br>Control: 33.4 (6.4)<br><br>Intervention: 38.5 (3.1)	Randomised Controlled Trial	<u>Intervention:</u> **Touch and massage.**<br><br>Mothers attended a 1-hour massage class taught by a nurse CIMI (certified infant massage instructor) and were subsequently asked to participate in at least three bedside massage instruction sessions taught within the next week. Infants received massage for seven consecutive days, from the mother or a CIMI. The touch procedure lasted 20 minutes.<br><br><u>Control:</u> Infants received all usual hospital services including medical care, physical and occupational therapy services and developmentally supportive nursing care.	*1. Caregiver (mother) satisfaction with their infant's care*<br><br>2. *Caregiver satisfaction with the neonatal unit and the massage therapist*	During babies' admission (three times)<br><br>- At baseline<br><br>- Upon completing the 7-day massage program<br><br>- 1 month following intervention	*Satisfaction questionnaire*<br><br>Two questionnaires were developed by the research team.<br><br><u>Validation:</u> No content validity or reliability testing reported.<br><br>-1st questionnaire (at baseline): a brief self-report questionnaire about caregiver satisfaction with their infant's care until that moment. No further details reported.<br><br>-2nd questionnaire (upon completing the 7-day massage program and 1 month following intervention): a 10-minute satisfaction questionnaire relating to infant's response and caregiver satisfaction with the neonatal unit and the massage therapist.<br><br><u>Number of questions:</u> not stated.<br><br>Likert scale (1 very dissatisfied-4 very satisfied).<br><br>Sample statements:<br>*'How satisfied do you feel giving massage to your infant?'*<br>*'I feel that massage improved my infant's hospital stay.'*	**It is unclear in the report if specific between-group comparisons and statistical analysis were conducted.**<br><br><u>At baseline and day 7:</u><br>All caregivers were highly satisfied with the medical treatment their infant received.<br><br><u>At day 7 and 1 month follow-up:</u><br>All caregivers participating in the massage group reported high levels of satisfaction regarding their relationship with their infant and the massage program's impact on that relationship.<br><br>Slight improvements in satisfaction regarding time the caregiver spent with the infant and involvement in the infant's care were observed between day 7 and the 1-month follow-up (no further information reported).	**No**	**3**
8. Koh et al. (2007), Australia	Mothers /200	Not stated	Randomised, Controlled Trial	<u>Intervention:</u> **Provision of taped conversations with neonatologists to mothers.**<br><br>The initial conversation and	*Satisfaction with conversations held with the neonatologist*<br><br>*Satisfaction with the*	During admission period and post discharge<br><br>- At 10 days	*Individual questions and a satisfaction scale*<br><br><u>Validation:</u> No content validity or reliability	No differences were found between the two groups in satisfaction with conversations.<br><br>**Mothers of babies with a poor outcome in the tape group were, however, significantly**	**No**	**1**

Confidential: For Review Only

				subsequent conversations of significance with a neonatologist were taped and analysed (for both groups). Mothers received a tape of each of the conversations and a tape recorder.<br><br>Control: Usual care. Mothers were not given the tape or a recorder.	*tape*	- At 4 months<br><br>- At 12 months<br><br>No pre-intervention parent satisfaction data available for comparison.	testing reported.<br><br>Number of questions: not stated.<br><br>Likert scale (1-5 most satisfied)<br><br>Questions related to: Satisfaction with amount and quality of information presented, doctors' communication skills, patient's participation in the conversation.<br><br>A satisfaction scale was used to assess: Satisfaction with the tape	**more satisfied with the conversations:**<br><br>**Satisfaction** **Intervention** **Control**<br>**Mean (95%CI)** 115 (104-123.2) 100.5 (94.1-109.4)<br><br>**p-value** 0.0051<br><br>Most (71-92%) of the mothers given the tapes stated that they helped their understanding, reminded them of what had been said, and helped their family to understand and recall information.		
9. Mitchell-DiCenso et al. (1996), Canada	Mothers and fathers/ 482	Mean (SD)<br><br>Intervention: 35.1 (4.5)<br><br>Control: 35 (4.3)	Randomised, Controlled Trial	Intervention: **Clinical Nurse Specialist/ neonatal practitioner team (CNS/NP) care.**<br><br>Infants of intervention parents were assigned to be cared for by the Clinical nurse specialist/neonatal practitioner CNS/NP team during the day and by paediatric residents during the night.<br><br>Control: Paediatric residents cared for infants of control parents around the clock. Neonatologists supervised both teams.	*Parent satisfaction with care*	During admission period and post discharge (twice)<br><br>- On 5th day after admission (full questionnaire administered)<br><br>- After discharge over the phone (administered only the questions related to satisfaction with discharge process)<br><br>No pre-intervention parent satisfaction data available for comparison.	*Satisfaction questionnaire (Validated)*<br><br>The study team developed and used the validated *Neonatal Index of Parent Satisfaction (NIPS)* questionnaire[16].<br><br>Number of questions: not stated.<br><br>NIPS score range (27-189); higher scores indicating greater satisfaction with care.	**No statistically significant difference between groups.**<br><br>**Intervention** **Control** **p-value**<br>**NIPS Mean** 140 139 0.67<br>**Difference in means** 1.0, CI (-3.6-5.6)	**No**	**2**
10. Broyles et al. (1992), USA	Mothers /25	Mean (SD)<br><br>Intervention: 33.4 (4)<br><br>Flexible: 34 (4)	Randomised Controlled Trial	Intervention: **Detailed consent.**<br><br>Mothers were given information about mechanical ventilation. Detailed risk/benefit disclosure was provided both verbally and in writing.<br><br>Control:<br>Mothers were given a brief verbal description about mechanical ventilation supplemented with detailed verbal and written disclosure if desired by them (flexible consent).	*Maternal satisfaction with the information provided about mechanical ventilation*	During babies' admission (once)<br><br>- 24-48 hours after the intervention<br><br>No pre-intervention parent satisfaction data available for comparison.	*An interview* evaluating maternal satisfaction with the information provided about mechanical ventilation.<br><br>Validation: A psychiatrist with a special interest in interviewing techniques was consulted in designing and standardising this assessment.<br><br>A research nurse conducted the interview, "checking" each mother against one option regarding:<br><br>- Amount of information: Right amount-Too much-Too little<br><br>- Information made coping: More Difficult-Easier-No effect-Uncertain.	This study is measuring and comparing satisfaction with two different interventions (detailed vs flexible consent process), neither of which formally represent the usual routine care for all babies (no control).<br><br>**Small numbers. No data indicating statistical analysis conducted or evidence of statistically significant results.**<br><br>**Detailed consent** **Flexible consent**<br>**Right amount of information** 75% mothers 100% mothers<br><br>**Too little** 25% mothers<br><br>**Information made coping easier** 67% mothers 69% mothers	**No**	**3**

Confidential: For Review Only

Non-Randomised controlled trials (Non-RCT) by publication year

Author (Date), Country	Parents' gender/ Total sample Size	Infants' Gestational age (GA) in weeks	Study design	Intervention	Outcome measures	Timing of measurement	Method of measurement	Outcome	Was the intervention co-designed with parents	Improved parent satisfaction?
1. De Bernardo et al (2017), Italy	Mothers and Fathers /96	Mean (SD)<br><br>Control: 34.2 (5.25)<br><br>Intervention: 32.7 (5.25)	Non-randomized, prospective cohort pilot study<br><br>*Unit level effect*: Two different time periods	Intervention: **FCC (Family-Centered Care).**<br><br>Parents had access to NICU for 8 hours a day. The NICU was widened and paediatric nurses taught parents procedures and practices for 10 days. Parents could observe clinical bedside rounds, hold meetings with the physicians and use the rooms and kitchen.<br><br>Control: Parents were permitted to visit their baby in NICU for 1 hour a day.	*Parent satisfaction relating to 3 specific domains:*<br><br>1. Knowledge and Understanding<br><br>2. Communication and Collaboration<br><br>3. Privacy and confidentiality	During babies' admission (once)<br><br>- At discharge (pre-FCC cohort and post-FCC cohort)<br><br>No pre-intervention parent satisfaction data available for comparison (different parent groups pre and post intervention).	*Satisfaction questionnaire.*<br><br>Validation: The authors state the survey *"was designed and validated by Abdel-Latif et al[22]".* No content validity or reliability testing reported in the original paper.<br><br>9 questions<br><br>3 questions: Related to adequate and timely information about the baby's condition.<br><br>3 questions: Related to communication and collaboration with the healthcare team.<br><br>3 questions: Related to respect of patient privacy.<br><br>Likert (1 strongly disagree-5 strongly agree)	**7/9 individual statements in the parent satisfaction questionnaire scored higher in the FCC compared to the NFCC (statistically significant difference).**<br><br>Example statement:<br>*"I have received adequate information about my baby's condition and management."*<br><br>| | **Intervention** | **Control** | **p-value** | | **Median score** | 5 (3.45-5) | 4 (3-5) | <0.05 |	**No**	**1**
2. Kadivar et al. (2017), Iran	Mothers /68	<=30 - 36	Non-randomised, Convenience sampling.<br><br>*Group level effect:* Intervention/control groups.<br><br>Pre and post-intervention testing.	Intervention: **Internet-based education.**<br><br>Mothers were given a unique ID and password to use an educational website set up by the research team (files and clips). Mothers could visit the website from 5:00-6:00 pm for 10 days. They were also allowed to use the website outside of the above hours and to report the duration of using the website to the researcher. The mothers had to use the website at least 3 times during 10 days, each time for at least 30 min.<br><br>Control: Mothers in the control group received the routine education provided in the NICU.	*Maternal satisfaction*	During babies' admission (twice)<br><br>- Day 1 of intervention<br><br>- Day 10 of intervention	*Satisfaction questionnaire (Validated)*<br><br>The *"What Being The Parent of a Baby is Like-Revised" Questionnaire (WBPL- Revised)* was used. The original English version by Pridham and Chang[23] was translated to Persian.<br><br>11 questions<br><br>Total satisfaction score range (11–99)	There was a significant difference in the mean score of satisfaction between cases and controls while the mean score of satisfaction increased in both groups.<br><br>**Comparison of the mean score between the two groups showed that the level of satisfaction was significantly higher in the case group versus the control group.**<br><br>| | **Intervention** | **Control** | **p-value** | | **Mean(SD) Satisfaction before intervention** | 81.62(13.50) | 85.71(9.46) | 0.993 | | **Satisfaction after intervention** | 93.88 (5.38) | 90.12 (7.78) | 0.024 |	**No**	**1**

Confidential: For Review Only

3. Kadivar et al. (20), Iran	Mothers /70	Mean (SD)<br><br>Control 31.6 (2.4)<br><br>Intervention 32.9 (3.1)	Non-randomised, Convenience sampling.<br><br>*Unit level effect*: Two different time periods	Intervention: **Narrative writing.**<br><br>Mothers did narrative writing at least 3 times until the 10th day of admission.<br><br>Control: Mothers in the control group received the routine NICU treatment and care.	*Mothers' satisfaction with medical care provided by physicians, medical students, and nurses during neonatal admission to the NICU*	During babies' admission (twice)<br><br>- Day 3 of intervention<br><br>- Day 10 of intervention	*Satisfaction questionnaire (Validated)*<br><br>The *NIPS questionnaire* by Mitchell et al[16] was used and translated to Persian.<br><br>24 questions (Likert scale)<br><br>Likert (1 always or not satisfied-7 never or completely satisfied). A higher score indicates more satisfaction.	**The satisfaction level of the mothers in the intervention group increased significantly during the study.**<br><br>The results of independent t test showed a significant difference in the satisfaction changes of the mothers on the 3rd and 10th day of NICU admission between intervention and control groups, indicating the effectiveness of narrative writing.<br><br>The results of paired t-test also showed a significant difference in the mean satisfaction level of the mothers between the 3rd and the 10th day in the intervention group.<br><br>**Mean(SD)** — **Intervention** / **Control** / **p-value**<br>**Satisfaction after intervention** — 137 (15.2) / 102.3 (25.6) / 0.001	**No**	**1**
4. Garingo et al. (2016), USA	Not stated /9	23-39	Non-randomised, Convenience sampling.<br><br>*Group level effect:* Intervention/control groups<br><br>Post-intervention group testing only	Intervention: **Tele-rounding.**<br><br>Infants of intervention parents were cared for by an OFFSN (off site neonatologist) who was present in the NICU only via A remote-controlled robot. The OFFSN clinically assessed infants via the robot's integrated high-sensitivity, electronic stethoscope, with assistance from the nursing staff. During routine working hours the OFFSN was called to discuss new information or changes in the patients' status. Emergencies and out of hours work were covered by an ONSN (on site neonatologist).<br><br>Control: Infants of control parents received ONSN care. The attending neonatologist made daily patient rounds with the NICU team. After patient rounds, the NICU staff, under the supervision of the attending neonatologist implemented the care plan.	*Satisfaction with telemedicine*	During babies' admission (once)<br><br>- At the time of discharge<br><br>No pre-intervention parent satisfaction data available for comparison.	*Satisfaction questionnaire*<br><br>Validation: No content validity or reliability testing reported.<br><br>Number of questions: not stated.<br><br>Likert (1 excellent-5 very poor).	**Only the intervention group was assessed and only post-intervention.**<br><br>The authors reported that the parents surveyed were *"satisfied with their experience. 100% responded that they felt comfortable talking to the OFFSN on the mobile robot and would allow their infant or themselves to be cared for by a physician via telemedicine in the future."*	**No**	**4**
5. Globus et al. (2016), Israel	Mothers and fathers /Number of total surveys returned: 178	~40% in each group <32	Non-randomised, Convenience sampling.<br><br>*Unit level effect:* Two different time periods	Intervention: **SMSi- Short Message Services Implementation.**<br><br>Parents were updated daily regarding the health status of their infant via SMS (short-message-services) from the Electronic Patient Record. All SMS messages were sent at 09:00am, including one-sentence prefaces and conclusions with updated information(e.g. location of the infant's crib and current weight). Information regarding acute events or deterioration of the infant's medical condition was not included in the SMS, but was delivered personally to the parents in real time.<br><br>Control: Routine care pre-SMS implementation.	*1. Parent satisfaction related to parent communication with the medical staff*<br><br>*2. Overall parent satisfaction with treatment and staff attitudes throughout hospitalisation.*	During babies' admission (once)<br><br>- pre-SMS cohort and post-SMS cohort<br><br>No pre-intervention parent satisfaction data available for comparison (different parent groups pre and post intervention).	*Satisfaction questionnaire*<br><br>The *"Parents' attitudes regarding their experience during their infants' hospitalisation in the NICU"* questionnaire was used, as well as selected items from a literature review of similar questionnaires, including that by York Hospital[24] and by Conner and Nelson [25].<br><br>Validation: No content validity or reliability testing reported.<br><br>Selected items related to four aspects of the NICU experience. 2 out of 4 directly assessed parent satisfaction:	Overall, in both periods, parents expressed a high degree of satisfaction regarding the medical treatment, the information given and the communication with the medical staff. Overall satisfaction with treatment and with staff attitudes throughout hospitalisation was slightly greater in the post-SMS cohort but did not reach statistical significance.<br><br>**In the post-SMS cohort, a statistically significant improvement was noted regarding physician availability and patience, parental feelings of comfort in approaching the physicians and nurses, and regularly receiving information regarding the infants' medical status from the physicians.**<br><br>**Mean (SD)** — **Post SMS** 4.1 (1.0) / **Pre SMS** 3.7 (1.3) / **p-value** 0.03<br><br>*Specific question: "I was pleased with the frequency with which I received information regarding my infant".*	**No**	**1**

Confidential: For Review Only

							1. Parental assessment of their communication with the medical staff.<br><br>Likert scale (1 do not agree at all-5 strongly agree)<br><br>2. Overall satisfaction with treatment and staff attitudes throughout hospitalisation.<br><br>Visual analog scale (scores range 0-10). Higher scores reflect greater satisfaction.	Although improvement in all other categories was documented, it did not reach statistical significance.		
6.Kazemian et al. (2016), Iran	Mothers /220 newborns (assumed 220 mothers)	>37	Non-randomised, Convenience sampling.<br><br>*Group level effect:* Intervention/control groups<br><br>Post-intervention testing only	Intervention: **Rooming-in care.**<br><br>Mothers and babies were admitted to a different atmosphere to the routine care. This facilitated the mothers and neonates with separate beds along with phototherapy devices and nursing clinical supervision.<br><br>Control: The routine care practiced in this neonatal unit supported partial stay of mothers beside their neonates, while sitting on chairs; however, most of the time the mother-infant dyad was separated.	*Maternal satisfaction with the neonatal care services and hospital stay comfort*	During babies' admission (once)<br><br>-Not stated exactly when<br><br>No pre-intervention parent satisfaction data available for comparison.	*Satisfaction questionnaire*<br><br>Validation: No content validity or reliability testing reported.<br><br>The authors state, *"a validated self-made questionnaire was employed, which was filled in by some trained midwives."* No further information on validation processes, number of questions or name of the questionnaire was provided.<br><br>Likert (5 very satisfied-1 dissatisfied).	**The level of satisfaction was significantly higher in the intervention group, compared to that in the control group.**<br><br>**Intervention / Control / p-value**<br>**Satisfaction %** 26.6 / 18.8 / 0.027	**No**	**1**
7. Petteys et al. (2015), USA	Not stated/ 10 parents included in sample analysis for parent satisfaction assessment	24-36+	A prospective cohort design.<br><br>A feasibility study.<br><br>*Group level effect:* Intervention/control groups<br><br>Post-intervention testing only	Intervention: **PC (Palliative care).**<br><br>PC nurses provided important continuity of care for NICU infants clinically requiring PC and at least weekly verbal support of parents. The PC service also coordinated family conferences, provided or requested orders to improve infant symptom management and comfort, and addressed parental coping and self-care.<br><br>Control: Usual clinical care for infants not requiring PC.	*Overall satisfaction with care received*	During babies' admission (once)<br><br>- At discharge (or study closure for infants who remained hospitalised)<br><br>No pre-intervention parent satisfaction data available for comparison.	*Satisfaction questionnaire*<br><br>A researcher-created questionnaire based on extensive current literature review.<br><br>Validation: Partially reported. Authors stated content validity testing took place; no information on reliability testing provided.<br><br>1 question<br><br>Likert (1 extremely dissatisfied-4 to extremely satisfied).<br><br>Optional free text (description of specific experiences impacting satisfaction with care)	**Parent satisfaction response numbers were small (n= 10), thus statistical comparison of parental satisfaction between cohorts was not possible.**<br><br>However, 100% of responding PC parents (n= 2) reported being "extremely satisfied" with care, whereas only 50% of responding usual care parents (n= 4) reported extreme satisfaction.	**No**	**3**

Confidential: For Review Only

8. Van de Vijver and Evans (2015), UK	Not stated /105	Not stated	Non-randomised, Convenience sampling.<br><br>*Unit level effect:*<br>Three different time periods	Intervention: **Baby diary.**<br><br>Each parent received a communication diary on their infant's admission to the unit. Doctors and nurses wrote in infant status updates and kept an infant interaction log with parents and staff. Parents wrote in memories and questions for staff to address during face-to-face communication.<br><br>Control: Routine care, before implementation of the diaries.	*Satisfaction with communication from neonatal staff*	During babies' admission (three times)<br><br>- On the day of babies' discharge at study baseline<br><br>- On the day of babies' discharge at 1 month<br><br>On the day of babies' discharge at 15 months	*Satisfaction questionnaire*<br><br>The study team designed a questionnaire, based on the Department of Health[26] and the National Institute for Health and Care Excellence (NICE)[27] quality standards for specialist neonatal care.<br><br>Validation: No content validity or reliability testing reported.<br><br>5 questions ("yes or no")	**Small numbers. No data indicating statistical analysis conducted or evidence of statistically significant results.**<br><br>*"I was receiving regular communication from staff"*<br>94% - 1 month post diary cohort<br>93% - 15 months post diary cohort<br>77% - pre diary cohort<br><br>*"My questions and concerns were being addressed"*<br>100% - 1 month post diary cohort<br>93% - 15 months post diary cohort<br>91% - pre diary cohort<br><br>"I feel more involved in my baby's care"<br>92% - 1 month post diary cohort<br>100% - 15 months post diary cohort<br>88% - pre diary cohort	**Yes.**<br><br>The intervention's concept was created by the project leaders following analysis of baseline questionnaire results and implemented after multi-disciplinary input and discussion with staff and parents.	3
9. Voos and Park. (2014), USA	Not stated / 62	Not stated	Non-randomised, Convenience sampling.<br><br>*Unit level effect:*<br>Two different time periods	Intervention: **OU (Open Unit) policy.**<br><br>Parents were allowed access to their baby 24 hours a day, 7 days a week.<br><br>Control: Parents pre-OU implementation received routine care. The unit was closed to parents during nurse change of shift in mornings and evenings.	*Parent satisfaction with how much time parents get to spend with their baby*	After babies were discharged (once)<br><br>- After pre-OU parents were discharged<br><br>- After post-OU parents were discharged	*Single question (From a validated questionnaire)*<br><br>The question *"Did you get to spend as much time as you wanted with your baby?"* was used from the NRC (National Research Corporation) Picker parent survey[28].<br><br>1 question ("yes or no")	**Small numbers. No data indicating statistical analysis conducted or evidence of statistically significant results.**<br><br>*"Did you get to spend as much time as you wanted with your baby?" Yes.*<br><br>Pre OU 78% (18/23)<br>Post OU 92% (36/39)	**Yes.**<br><br>The NICU has a multidisciplinary FCC (Family-centered care) committee that also includes parents. The FCC committee conducted this project.	3
10. Segre et al. (2013), USA	Mothers /23	Mean (SD) 31.57 (5.30)	For the outcome of parent satisfaction:<br><br>Non-Randomised, Convenience sampling.<br><br>*Group level effect:*<br>Intervention/control groups<br><br>Post-intervention group testing only	Intervention: **(LV) Listening visits.**<br><br>Mothers met with the LV provider for up to six 50-min LV sessions, conducted in a private hospital location, every 2–3 days, within a 1-month frame. The general structure of a visit entailed greeting, debriefing, updating on current issues, working an agenda through listening and problem solving, and providing closure through summary.<br><br>Control: Women who did not meet the specific criteria (e.g. minimum score on depression scale) were not invited to join the treatment trial and received routine NICU care/support instead.	*Satisfaction with the treatment and the outcome.*	During babies' admission (once)<br><br>- Not stated exactly when<br><br>No pre-intervention parent satisfaction data available for comparison.	*Satisfaction questionnaire*<br><br>The *Client Satisfaction Questionnaire* was used.<br><br>Validation: Partially reported. Authors stated reliability testing took place; no information on content validity provided.<br><br>8 questions.<br><br>Format of questions: not stated	**Only the intervention group was assessed and only post-intervention.**<br><br>The authors reported:<br><br>*"The majority of women who received LVs were highly satisfied with the intervention".*<br><br>*"The average score for the Client Satisfaction Questionnaire was 29.91, comparable to levels of satisfaction reported by clients receiving depression treatment from a mental health professional."*<br><br>*"91.3% of our participants rated the quality of help they received as excellent."*	**No**	4
11. Palma et al. (2012), USA	Not stated / 26 families returned the survey containing the satisfaction measure)	Not stated	Non-randomised, Convenience sampling.<br><br>*Unit level effect:*<br>Two different time periods	Intervention: **YBDU (Your Baby's Daily Update). A daily parent update letter generated from the Electronic Medical Record (EMR).**<br><br>Parents were given daily YBDU reports, printed automatically from the EMR. The YBDU included information about an infant's status during the past 24 hours and a hand-written update by the infant's care provider.<br><br>Control: Parents received routine care and usual verbal updates (6 months pre- adoption of YBDU).	*Satisfaction with YBDU*	During babies' admission (once)<br><br>- Not stated exactly when<br><br>No pre-intervention parent satisfaction data available for comparison (different parent groups pre and post intervention).	*Satisfaction questionnaire*<br><br>A questionnaire including items regarding adoption of and satisfaction with YBDU was used.<br><br>Validation: No content validity or reliability testing reported.<br><br>Number and format of questions: not stated.	**Only the intervention group was assessed and only post-intervention.**<br><br>The authors reported:<br>*"When asked to rate the statement "I like receiving Your Baby's Daily Update", 96% of families who used YBDU as an information source responded with the highest rating, "always"."*	**No**	4

Confidential: For Review Only

12. Stevens et al. (2011), USA	Mothers /147. For the OPBY NICU, 58 surveys were returned. For the SFR NICU, 89 were returned	Mean (SD)<br><br>Control 35 (4)<br><br>Intervention 34 (3)	Cohort trial. This research was part of a large prospective evaluation.<br><br>*Unit level effect:* Two different time periods	Intervention: **SFR (Single-family room) NICU for neonatal care.**<br><br>Parents could visit their baby, room-in, do kangaroo care, and breastfeed at any time, in individual rooms containing a bed, desk, closet, telephone, chair and a refrigerator for breast-milk storage.<br><br>Control: OPBY (Open-bay) NICU. The traditional open-bay NICU was typical of facilities built before 1980. All neonates, family members, staff, monitors, and equipment were visible for all neonates in each room. Portable partitions were placed around the incubator for breastfeeding and kangaroo care.	*Parent satisfaction with different elements of NICU:*<br>*- Delivery*<br>*- Environment*<br>*- Nurses*<br>*- Physicians*<br>*- Discharge*<br>*- Personal*<br>*- Overall Assessment*	After babies were discharged (once)<br><br>- Mailed within 60 days of discharge of parents' infants from the NICU<br><br>No pre-intervention parent satisfaction data available for comparison (different parent groups pre and post intervention).	*Satisfaction questionnaire*<br><br>A questionnaire from Press Ganey Associates[29] was used. Also included were three questions added by the investigators.<br><br>Validation: Partially reported. The original questionnaire was validated questionnaire but no content validity or reliability testing was reported regarding the 3 questions added by the study team.<br><br>42 questions in total (7 categories):<br>Delivery, Environment, Nurses,<br>Physicians, Discharge, Personal,<br>Overall Assessment.<br><br>Likert (1 very poor-5 very good).	**Statistically significant improvement was found for the survey categories of Environment, Overall and the Total survey.**<br><br>Estimated numbers from report's figures as numbers not provided):<br><br>**Median** **SFR** **OPBY** **p-value**<br>Environment 4.7 3.7 <0.001<br>Overall 5 4.8 0.018<br>Total 4.7 4.5 0.045<br><br>16 items composite score for family-centered care: 4.4 4.0 0.017	**Yes.**<br><br>Former NICU parents were involved in all phases of planning for the new SFR NICU.	**1**
13. Voos et al. (2011), USA	Not stated /28	Not stated	Non-randomised, Convenience sampling.<br><br>*Unit level effect:* Two different time periods	Intervention: **Family-centered rounds (FCRs).**<br><br>Parents were invited to attend rounds and choose their level of involvement (attend every day, not at all, periodically). Parents received a handout explaining that the team would still be communicating with the parents if the parents were unable to attend FCRs. For confidentiality concerns, Parents were asked to step out of the room while rounds of others' infants took place. The staff augmented FCRs by meeting with parents again after rounds if needed.<br><br>Control: Parents received routine care. Prior to implementation of FCR parents were asked to leave the unit during rounds.	*Global satisfaction with the NICU experience*	During babies' admission (twice)<br><br>- Prior to FCR<br><br>- 6 months after starting FCR	*Satisfaction questionnaire (Validated)*<br><br>*The NIPS* questionnaire[16].<br><br>24 questions: looking at satisfaction in different areas of the NICU (medical caregivers, communication, tests, and procedures).<br><br>Likert scale (1-7 points).	**A subset of NIPS items related to communication (i.e. being kept informed as to changes in the infant's condition, meeting with physicians, and information about long-term expectations) yielded a significant increase from pre to post FCR scores.**<br><br>**post FCR** **pre FCR** **p-value**<br>**NIPS score** 5.5 4.4 <0.01<br><br>The average score on the NIPS did not change significantly.	**No**	**1**
14. Weiss et al. (2010), USA	Mothers /84	Mean (SD)<br><br>Pre-intervention group: 32 (4.4)<br><br>Post-intervention group: 32 (9)	Non-randomised, Convenience sampling<br><br>*Unit level effect:* Two different time periods	Intervention:<br>**An intervention to increase PMP (Principal Medical Providers) availability and communication frequency.**<br><br>(1) A brief education module for PMPs was introduced, (2) parents received a contact card with PMP names, job descriptions and contact information and (3) a large poster of the faces, names and titles of the PMPs was placed at the parent entrance of the NICU.<br><br>Control: Parents received routine care in the pre-intervention cohort, without the above.	*Parent satisfaction with physician and nurse practitioner communication*	During babies' admission (twice)<br><br>- Pre-intervention<br><br>- Post-intervention	*Satisfaction Questionnaire (Validated)*<br><br>A pilot survey written by Press Ganey and the Picker Institute was used and revised based on parent responses[30-33].<br><br>6 open-ended questions (Quantity of communication)<br><br>6 Likert scale questions (range questions (Availability, understanding, reciprocity, empathy, overall satisfaction)	**Overall satisfaction, based on the ordinal analysis of the five-point Likert scale, was significantly higher after the intervention (P<0.01).**<br><br>**Overall satisfaction, dichotomised into a satisfied subgroup and a dissatisfied subgroup for each cohort, was also significantly increased after the intervention.**<br><br>**post -ntervention** **pre-intervention**<br>**Very satisfied /somewhat satisfied** 97%(32/33) 74%(37/50)<br><br>**p-value** <0.01	**No**<br><br>Authors stated that only after implementation of the intervention many parents (both satisfied and unsatisfied) gave suggestions for improvement.	**1**

Confidential: For Review Only

15. Foster et al. (2008), Australia	Mothers and fathers /93<br><br>5 Special Care Nurseries	Mean (SD)<br><br>Headbox: 36.5 (2.6)<br><br>CPAP: 36 (3)	Non-randomised, Convenience sampling<br><br>*Group level effect:* Intervention 1/ intervention 2 groups<br><br>Post intervention testing only	Intervention 1: **Infants received headbox oxygen treatment for respiratory distress.**<br><br>Intervention 2: **Infants received continuous oxygen positive airway pressure (CPAP) treatment for respiratory distress.**	*Satisfaction with treatment (i.e. headbox oxygen or CPAP)*	During babies' admission (once)<br><br>- Within 5 days of the babies' admission<br><br>No pre-intervention parent satisfaction data available for comparison.	*Single question*<br><br>Validation: No content validity or reliability testing reported.<br><br>1 likert scale question (1 not at all satisfied-5 extremely satisfied).	**Parents with babies receiving CPAP rated their satisfaction with the baby's treatment statistically significantly higher than the headbox group mean rating.**<br><br>**Headbox CPAP p-value**<br>**Mean (SD)** 3.71 (1.31) 4.51 (0.79) 0.001<br><br>The CPAP group averaged between *very and extremely satisfied* compared with parents of babies receiving headbox, who averaged between *satisfied and very satisfied ratings.*	**No**	**1**
16. Byers et al. (2006), USA	Only mothers reported /35	Preterm infants<br><br>Mean (SD)<br><br>Control: 28.9 (3.44)<br><br>Intervention: 28.6 (3.37)	For the outcome of parent satisfaction:<br><br>Non-randomised, Convenience sampling<br><br>*Group level effect:* Intervention/control groups<br><br>Post-intervention testing only	Intervention: **Infants received individualised, developmentally supportive family-centered care.**<br><br>Infants received the standard of care within the framework and philosophy of individualised, developmentally supportive family-centered interventions.<br><br>Control: Infants received the traditional NICU standard of care.	*Parent satisfaction relating to:*<br>*- parental perceptions of staff caring*<br>*- education received*<br>*- preparation for the parental role*<br>*- overall satisfaction with the NICU experience*	During babies' admission (once)<br><br>- On the day before discharge<br><br>No pre-intervention parent satisfaction data available for comparison.	*Satisfaction questionnaire*<br><br>The *NICU's parental satisfaction tool* was used.<br><br>Validation: Partially reported. Authors stated content validity testing took place, but "because of the disparate nature of the items, survey reliability was not assessed".<br><br>11 questions<br><br>Likert scale (1-5 strongly agree)	**Independent t-test analysis of parent satisfaction/perception scores showed no significant difference between groups.**<br><br>Example statement: "*I was satisfied with the car my baby and I received in the NICU"*<br><br>**Intervention Control p-value**<br>**Mean (SD)** 4.94(0.23) 4.71(0.47) 0.064<br><br>Both groups reported very high satisfaction with their NICU experience (4.4-5.0)	**No**	**2**
17. Mills et al. (2006), USA	Not stated/ not stated<br><br>Parents of infants from 6 hospitals	Not stated	Implementation project<br><br>Plan Do Study Act (PDSA) quality improvement testing	Intervention: **5 potentially better practices (PBPs) in the area of discharge planning.**<br><br>The project team iteratively implemented 5 PBPs:<br>1. Created an easy-to-use, easy-to-access discharge planning tool kit.<br>2. Restructured communication tools and processes to reflect a "plan for the day, the stay, and the way" to discharge.<br>3. Maximised the impact and use of caregiver educational tools, and updated materials and delivery systems for caregiver education.<br>4. Used various continuous quality improvement tools and processes to ensure parent/caregiver and staff satisfaction.<br>5. Analysed and enhanced interactions with and transfers into the community.<br><br>Control: N/A. No discrete control group. PDSA quality improvement methodology was applied to parent participants.	*General satisfaction*<br>*- with care*<br>*- parents' feelings about preparedness for discharge*<br>*- ability and confidence in feeding*<br>*- familiarity with their infant*<br>*- feeling like a parent*<br>*- participation in care*<br>*- adequacy of information from staff about medical and care issues*	During babies' admission (4 times)<br><br>- Not reported exactly when	*Satisfaction questionnaire*<br><br>The Internet-based parent satisfaction survey *"howsyourbaby.com"* that was developed especially for this NICU population was used.<br>Validation: No content validity or reliability testing reported.<br><br>Number and format of questions: not stated.	Through multiple rapid-cycle projects, the project's collaborative group made changes within the 5 PBP plans.<br><br>**Parent satisfaction measures were used to longitudinally monitor the changes made, rather than make direct group comparison. No data indicating statistical analysis conducted or evidence of statistically significant results.**<br><br>Parent satisfaction survey results (all centers combined) were high across 4 measurement quartiles. No specific interquartile analysis was reported.<br><br>Parent readiness for discharge was high at the beginning and throughout the collaborative. Parents' receiving *"just the right amount of information"* regarding car seat trials and safe sleep demonstrated some variability throughout the collaborative.	**No**	**3**

Confidential: For Review Only

18. Wielenga et al. (2006), The Netherlands	Mothers and fathers / 46	Mean (SD)<br><br>Control: 28.5 (26.0–29.9)<br><br>Intervention: 28.3 (25.6-29.9)	Non-randomised, Convenience sampling<br><br>*Unit level effect:* Two different time periods	<u>Intervention:</u> **The Newborn Individualised Developmental Care and Assessment Program (NIDCAP).**<br><br>Infants received care according to general NIDCAP principles and parents were taught how to provide it. Caregiving plans were designed on the basis of the infant's current developmental stage and medical condition as well as on the needs of the family. Caregivers learnt to watch sensitively and note the reactions of the infant to different types of handling and care, and thus make continuously appropriate adjustments.<br><br><u>Control:</u> Infants received traditional neonatal care practiced at that time.	*Parent satisfaction relating to:*<br>*-Overall rating*<br>*-Care of the baby*<br>*-Communication with staff*<br>*-Involvement in care -Being prepared*<br>*-Support*<br>*-Being a parent*<br>*-Being near your baby*<br>*-Total score*	After babies were discharged (on day of discharge/ transfer)<br><br>- Pre NIDCAP cohort<br><br>- Post NIDCAP cohort	*Satisfaction questionnaire (Validated)*<br><br>The *NICU-PSF* was used and translated from English to Dutch[34].<br><br>62 questions<br><br>Closed and open-ended questions.<br><br>Different rating scales used (5-point rating scale from "extremely satisfied" to "not at all satisfied" or "excellent" to "poor").<br><br>Total score range (50-243 points)	**The intervention group's mean total score was significantly higher than the control.**<br><br>**Intervention Control**<br>**Mean (SD)** 185.67(17.74) 174.04(20.98)<br>**p-value** 0.041<br><br>Almost all separate concepts showed an increase in their mean scores. The concept of "*being a parent*" had a slightly lower mean score (9.39, SD = 1.73) in the intervention group than in the control group (9.78, SD = 2.09).<br><br>**The concept of *"preparedness"* showed statistically significant difference:**<br>**Intervention Control p-value**<br>**Mean** 16.38 13.83 0.038	**No**	**1**
19. Penticuff and Arheart. (2005), USA	Dyads (both parents or mother with her designated support person)/ 122 mothers<br><br>Results based only on mothers' data.	Not stated	A repeated measures design<br><br>- First 2 years (control group data collection)<br><br>- Year 3 (staff training)<br><br>- Year 4 (implementing the intervention)<br><br>- Year 5 (collecting data from the intervention group)<br><br>*Unit level effect:* Two different time periods	<u>Intervention:</u> **The Newborn Individualised IPC- CPM intervention (Infant Progress Chart) - (Care Planning Meetings).**<br><br>Both the mother and father (or the mother and her designated support person) were shown how to use the Infant Progress Chart and attended 3 Care Planning Meetings (with neonatologists/Neonatal Nurse Practitioners).<br><br><u>Control:</u> During the control phase, professionals carried out usual communication and interaction with control group parents.	*Satisfaction with participation in decision making* was measured by 5 collaboration indices:<br><br>Satisfaction with<br>(1) Care<br>(2) Relationships with professionals<br>(3) Decision input<br>(4) The process of decision making<br>(5) Decisions made	During babies' admission (three times)<br><br>- Within 0–3 days<br><br>- 9– 12 days<br><br>- 25–28 days of an infant's admission to the NICU	*Three satisfaction questionnaires*<br><br>1. Two subscales of the investigator-designed *"Parents' Understanding of Infant Care and Outcomes Questionnaire"* were used to measure Satisfaction with Care (1).<br><br><u>Validation:</u> Partially reported. Authors stated content validity testing took place; no information on reliability testing provided.<br><br>30 questions.<br><br>Five-point Likert scale.<br><br>2. A subscale of the investigator-designed *"Relationships with Professional and Decision Input Questionnaire"* was used to measure Satisfaction with relationships (2).<br><br><u>Validation:</u> Partially reported. Authors stated content validity testing took place; no information on reliability testing provided.<br>12 questions.<br><br>Five-point Likert scale<br><br>3. <u>Validated.</u><br>The *"Collaboration and Satisfaction About Care Questionnaire"* developed by Baggs[35], was used to measure Satisfaction with decision input (3), with decision process (4) and	**The intervention group was more satisfied with the amount of decision input they had (3) and with the process by which medical decisions were made (4).**<br><br>**Intervention Control p-value**<br>Decision input amount (3)<br>**Mean** 33.44 30.05 0.058<br><br>Process of decision making (4)<br>**Mean** 120.20 104.95 0.012<br><br>There were no statistically significant differences between control and intervention groups in satisfaction with their infants' care (1), with relationships with NICU professionals (2) and with the decisions made for infant treatment (5).	**No**	1

Confidential: For Review Only

							with decisions made (5).<br><br>9 questions.<br><br>7-point scale, (1 strongly disagree -7 strongly agree)			
20. Byers et al. (2003), USA	Mothers/ 19	Mean (SD)<br><br>Intervention: 28.9 (2.42)<br><br>Control: 29 (2.00)	For the outcome of parent satisfaction:<br><br>Non-randomised, Convenience sampling<br><br>*Group level effect:* Intervention/control groups<br><br>Pre and post-intervention testing	Intervention: **Co-bedding premature multiple-gestation infants in incubators.**<br><br>Infants were nursed in the same incubator using a co-bedding protocol (e.g. recording all of the care provided to one infant before providing care to the second infant)<br><br>Control: Single-bedding premature multiple-gestation infants in incubators.	*Parent satisfaction related to:*<br>*- staff concern*<br>*- support of family*<br>*- staff explanations*<br>*- infant environment,*<br>*- comfort with feeding*<br>*- kangaroo care encouragement*<br>*- staff explanation of signs of infant stress*<br>*- visiting schedule*<br>*- overall satisfaction with the NICU experience*	During babies' admission (twice)<br><br>- At baseline<br><br>- 5 days later	*Satisfaction questionnaire*<br><br>The NICU's standard parental satisfaction tool was used.<br><br>Validation: Partially reported. Authors stated content validity testing took place, but because of the disparate nature of the items, survey reliability could not be assessed.<br><br>11 questions.<br><br>5-point Likert-type scale.	**The only significant difference for a post-intervention item was a higher score for the item "Attempts were made to create a quiet environment for my baby."**<br><br>**Intervention Control p-value**<br>**Mean** 4.80 3.89 0.033<br><br>Independent t-tests comparing the co-bedded and control group parental scores found no significant differences in their parental satisfaction scores, except for higher baseline parental satisfaction scores (p=0.029) in the co-bedded group.	**No**	**1**
21. Polizzi et al. (2003), USA	Mothers and fathers/ 33	Mean (SD)<br><br>Intervention: 33.08 (1.31)<br><br>Control: 32.97 (1.9)	A retrospective, comparative, descriptive design.<br><br>*Unit level effect*	Intervention: **Co-bedding multiple-gestation infants in the NICU.**<br><br>Multiple-gestation infants were nursed in the same incubator or crib. The intervention was evaluated retrospectively after implementation of a co-bedding practice protocol.<br><br>Control: Traditionally-bedded group (babies were routinely placed in separate incubators or cribs)	*Parental satisfaction as measured by 9 questions relating to parent perceptions and their baby's care*	After babies were discharged (once)<br><br>- All parents were mailed the survey. A second survey was sent to those who did not respond after 2 months<br><br>No pre-intervention parent satisfaction data available for comparison.	*Satisfaction questionnaire*<br><br>The *parental perception/ satisfaction tool* was used.<br><br>Validation: Partially reported. Authors stated content validity testing took place; no information on reliability testing provided. 6/9 questions were from a similar tool that was validated by the Vermont Oxford NICU Quality Improvement Initiative[36].<br><br>9 questions (such as *"I was satisfied with the care my babies received in the hospital")*.<br><br>Likert (1 strongly disagree- 5 strongly agree)	Mothers reported overall satisfaction with the NICU care and staff, as well as adequacy of their ability to care for their infants after discharge, with scores ranging from 4.19 to 4.71.<br><br>**The only survey item score that was significantly different between groups was for the item *"I was encouraged by the hospital staff to bond with my babies."***<br><br>**Intervention Control p-value**<br>**Mean** 4.71 4.36 0.049	**No**	**1**
22. Legault and Goulet. (1995), Canada	Mothers/ 61 completed both tests	Mean (range)<br><br>30 (24-35)	Time-series design<br><br>*Group level effect:* Same group exposed to both methods with post-method testing only.	Intervention: **Kangaroo method of removing an infant from an incubator.**<br><br>Mothers were taught the "kangaroo method" (skin-to-skin contact): the infant wears a diaper and a head cap and is placed in a vertical position on the parent's bared chest. A flannel blanket covers the infant's back, and the parent's clothing is fastened around the infant. The parent sits in a rocking chair, inclined so that the infant's head is at an angle of approximately 60'.<br><br>Control: Traditional method. Newborns wearing a diaper and a head cap, are wrapped in a blanket and placed in their parent's arms. The parent keeps the infant's head at an	*Mothers' satisfaction with:*<br>*- Each method of removing an infant from incubator*<br>*- Her feelings after each method*	During babies' admission (twice)<br><br>- After the intervention<br><br>- After the control method<br><br>No pre-intervention parent satisfaction data available for comparison.	*Satisfaction questionnaire*<br><br>The *"Maternal Satisfaction Questionnaire"* was used. It was developed by integrating components described by Affonso et al[37] and the clinical experience of the investigators.<br><br>Validation: Partially reported. Authors stated content validity testing took place; no information on reliability testing provided.<br><br>15 questions<br><br>Likert (1 very much-5	**Regardless of the method tested, mothers expressed high levels of satisfaction (it was the first time since giving birth that they could hold their infants).**<br><br>Three statements proved more powerful in discriminating between the methods:<br><br>**Rated higher after the kangaroo method test:**<br>*- "I like the contact with my baby's skin"* (p=0.0001)<br><br>**Rated higher after the traditional method test:**<br>*- "I like to talk to and whisper to my baby" (p = 0.015)*<br>*- "I looked into my baby's eyes and stared at his/her face" (p=0.0001)*	**No**	**1**

Confidential: For Review Only

				angle of approximately 60' to allow for better pulmonary functional residual capacity.			don't know)<br><br>An open-ended question invited the mother to list and explain anything else related to her experience.			

Confidential: For Review Only

1. intervention$.ti,ab.
2. tool$.ti,ab.
3. way$.ti,ab.
4. updat$.ti,ab.
5. method$.ti,ab.
6. information.ti,ab.
7. sms.ti,ab.
8. implement$.ti,ab.
9. 1 or 2 or 3 or 4 or 5 or 6 or 7 or 8
10. bab$3.mp.
11. preterm$.ti,ab.
12. pre term.ti,ab.
13. premature.ti,ab.
14. postterm.ti,ab.
15. post term.ti,ab.
16. infant$.ti,ab.
17. newborn$.ti,ab.
18. exp Infant, Newborn/
19. 10 or 11 or 12 or 13 or 14 or 15 or 16 or 17 or 18
20. neonatal intensive care.ti,ab.
21. neonatal unit$.ti,ab.
22. NICU.ti,ab.
23. SCBU.ti,ab.
24. neonatal itu.ti,ab.
25. special care baby unit$.ti,ab.
26. neonat$.ti,ab.
27. Intensive Care Units, Neonatal/
28. Intensive Care Units/
29. Critical Care/
30. Neonatal Nursing/
31. 20 or 21 or 22 or 23 or 24 or 25 or 26 or 27 or 28 or 29 or 30
32. parent$.ti,ab.
33. mother$.ti,ab.
34. father$.ti,ab.
35. exp Parents/

36. 32 or 33 or 34 or 35
37. satisfaction.ti,ab.
38. experience$.ti,ab.
39. Patient Satisfaction/
40. personal satisfaction/
41. communicat$.ti,ab.
42. exp Communication/
43. Health Communication/
44. Information Dissemination/
45. 37 or 38 or 39 or 40 or 41 or 42 or 43 or 44
46. 9 and 19 and 31 and 36 and 45

Confidential: For Review Only

BMJ Paediatrics Open

Interventions to improve quantitative measures of parent satisfaction in neonatal care: a systematic review

Journal:	*BMJ Paediatrics Open*
Manuscript ID	bmjpo-2019-000613.R1
Article Type:	Original research
Date Submitted by the Author:	17-Dec-2019
Complete List of Authors:	Sakonidou, Susanna; Imperial College London, Neonatal Medicine, School of Public Health, Faculty of Medicine<br>Andrzejewska, Izabela; Imperial College London, Neonatal Medicine, School of Public Health, Faculty of Medicine<br>Webbe, James; Imperial College London, Neonatal Medicine, School of Public Health, Faculty of Medicine<br>Modi, Neena; Imperial College London, Neonatal Medicine, School of Public Health, Faculty of Medicine<br>Bell, Derek; NIHR CLAHRC for Northwest London<br>Gale, Chris; Imperial College London, Neonatal Medicine, School of Public Health, Faculty of Medicine
Keywords:	Neonatology, Outcomes research, Patient perspective

SCHOLARONE™
Manuscripts

I, the Submitting Author has the right to grant and does grant on behalf of all authors of the Work (as defined in the below author licence), an exclusive licence and/or a non-exclusive licence for contributions from authors who are: i) UK Crown employees; ii) where BMJ has agreed a CC-BY licence shall apply, and/or iii) in accordance with the terms applicable for US Federal Government officers or employees acting as part of their official duties; on a worldwide, perpetual, irrevocable, royalty-free basis to BMJ Publishing Group Ltd ("BMJ") its licensees and where the relevant Journal is co-owned by BMJ to the co-owners of the Journal, to publish the Work in this journal and any other BMJ products and to exploit all rights, as set out in our licence.

The Submitting Author accepts and understands that any supply made under these terms is made by BMJ to the Submitting Author unless you are acting as an employee on behalf of your employer or a postgraduate student of an affiliated institution which is paying any applicable article publishing charge ("APC") for Open Access articles. Where the Submitting Author wishes to make the Work available on an Open Access basis (and intends to pay the relevant APC), the terms of reuse of such Open Access shall be governed by a Creative Commons licence – details of these licences and which Creative Commons licence will apply to this Work are set out in our licence referred to above.

Other than as permitted in any relevant BMJ Author's Self Archiving Policies, I confirm this Work has not been accepted for publication elsewhere, is not being considered for publication elsewhere and does not duplicate material already published. I confirm all authors consent to publication of this Work and authorise the granting of this licence.

Confidential: For Review Only

Interventions to improve quantitative measures of parent satisfaction in neonatal care: a systematic review

Susanna Sakonidou[1], Izabela Andrzejewska[2], James Webbe[3], Neena Modi[4], Derek Bell[5], Chris Gale[6]

1 Susanna Sakonidou, Clinical Research Fellow
Highest academic degree: MBBS
Neonatal Medicine, School of Public Health, Faculty of Medicine, Imperial College London, United Kingdom.
s.sakonidou@imperial.ac.uk

2 Izabela Andrzejewska, Neonatal Research Nurse
Highest academic degree: RN MSc
Neonatal Medicine, School of Public Health, Faculty of Medicine, Imperial College London, United Kingdom.
Izabela.ukpl@gmail.com

3 James Webbe, Clinical Research Fellow
Highest academic degree: MB BChir
Neonatal Medicine, School of Public Health, Faculty of Medicine, Imperial College. London, United Kingdom.
j.webbe@imperial.ac.uk

4 Neena Modi, Professor of Neonatal Medicine

Confidential: For Review Only

Highest academic degree: MD

Neonatal Medicine, School of Public Health, Faculty of Medicine, Imperial College London, United Kingdom.

n.modi@imperial.ac.uk

5 Derek Bell, Professor of Acute Medicine and NIHR CLAHRC Programme Director

Highest academic degree: MD

National Institute for Health Research Collaboration for Leadership in Applied Health Research and Care, Northwest London, United Kingdom.

d.bell@imperial.ac.uk

6 Chris Gale, Reader in Neonatal Medicine

Highest academic degree: PhD

Neonatal Medicine, School of Public Health, Faculty of Medicine, Imperial College London.

London, United Kingdom.

christopher.gale@imperial.ac.uk

Corresponding Author

Susanna Sakonidou, Clinical Research Fellow

Neonatal Medicine, School of Public Health, Faculty of Medicine, Imperial College London

369 Fulham Road, London, SW10 9NH, United Kingdom.

+ 44 (0) 203 315 5418, s.sakonidou@imperial.ac.uk

Confidential: For Review Only

Manuscript word count: 3245

ABSTRACT

Objective

Interventions improving parent satisfaction can reduce parent stress, may improve parent-infant bonding and infant outcomes. Our objective was to systematically review neonatal interventions relating to parents of infants of all gestations where an outcome was parent satisfaction.

Methods

We searched the databases MEDLINE, EMBASE, PsychINFO, Cochrane Central, CINAHL, HMIC, Maternity and Infant Care between 1/1/1946-1/10/2017. Inclusion criteria were randomised controlled trials (RCT), cohort studies and other non-randomised studies if participants were parents of infants receiving neonatal care, interventions were implemented in neonatal units (of any care level) and ≥1 quantitative outcome of parent satisfaction was measured. Included studies were limited to the English language only. We extracted study characteristics, interventions, outcomes and parent involvement in intervention design. Included studies were not sufficiently homogenous to enable quantitative synthesis. We assessed quality with the Cochrane Collaboration risk of bias tool (randomised) and the ROBINS-I tool (non-randomised studies).

Results

Confidential: For Review Only

We identified 32 studies with satisfaction measures from over 2800 parents and grouped interventions into 5 themes. Most studies were non-randomised involving preterm infants. Parent satisfaction was measured by 334 different questions in 29 questionnaires (only 6/29 fully validated). 18/32 studies reported higher parent satisfaction in the intervention group. The theme with most studies reporting higher satisfaction was parent involvement (10/14). Five (5/32) studies reported involving parents in intervention design. All studies had high risk of bias.

Conclusions

Many interventions, commonly relating to parent involvement, are reported to improve parent satisfaction. Inconsistency in satisfaction measurements and high risk of bias makes this low-quality evidence. Standardised, validated parent satisfaction measures are needed, as well as higher quality trials of parent experience involving parents in intervention design.

PROSPERO registration: CRD42017072388

Keywords: neonatology, parents, satisfaction

INTRODUCTION

One in 10 newborn babies in high-income countries require neonatal care[1]. This is stressful for parents, who often develop anxiety, depression and Post Traumatic Stress Disorder symptoms[2-4]. Parental stress interferes with parent-child bonding[5] and there is a well-established link between maternal mental health and infant development[6]. Parent satisfaction, defined as *"the perception of parents'*

Confidential: For Review Only

needs and expectations being met" is inversely related to parental stress[7]. As such, it is increasingly being used as a parent experience measure and neonatal service quality indicator. Interventions aimed at improving parent satisfaction have the potential to reduce parent stress, improve parent-infant bonding[8] and infant outcomes[9].

A range of parent-centred interventions, such as including parents on ward rounds, have recently become widespread in neonatal practice. Many are implemented on a small scale, without evaluating their impact on parent experience, making long-term integration into neonatal services challenging, while many others are using parent questionnaires. 'Parent satisfaction' as an outcome is gaining momentum, as neonatal trusts attempt to match more 'business-like models' where effectiveness of interventions (and evidence for change) is measured by quantitative outcomes. Moreover, where parent experience is measured as 'parent satisfaction', some studies include it as a primary outcome, whereas others use it as a secondary indicator to explore the parent point of view.

Furthermore, there are multiple experience measures available in addition to parent satisfaction, including parent stress, anxiety and depressions scales; both quantitative and qualitative. Finally, it is not known the degree to which parents are involved in the design of such interventions. There have been no previous systematic evaluations focused on interventions measuring parent satisfaction with neonatal care as an outcome.

The aim of this review is to identify and describe neonatal interventions relating to

parents of infants of all gestations where an outcome was parent satisfaction. For the reasons outlined above, we have only included studies that reported ≥1 quantitative measure of parent satisfaction. We aim to report each intervention's effect on parent satisfaction, as well as parent input in intervention design.

METHODS

We prospectively registered this study on PROSPERO[10] (prospective register of systematic reviews-CRD42017072388) and reported it using PRISMA guidelines[11]. We searched MEDLINE (Medical Literature Analysis and Retrieval System Online), EMBASE (Excerpta Medica database), PsychINFO (Psychological Information), Cochrane Central Register of Controlled Trials, CINAHL (CUMULATIVE Index to NURSING and Allied HEALTH LITERATURE), HMIC (Health Management Information Consortium), Maternity and Infant Care (online_ supplementaryFile1) for English papers published between 1946-October 2017, with update searches on 1st September 2018.

Inclusion criteria were: randomised controlled trials (RCT) and non-randomised studies (non-RCT) if participants were parents of infants receiving neonatal care, interventions were implemented in neonatal units and ≥1 quantitative outcome of parent satisfaction was measured. We have restricted our review to studies where ≥1 quantitative outcome of parent satisfaction was measured, in order to enable comparison of interventions, which has previously not been possible in any published review. Including studies with all available measures of parent experience (in addition to parent satisfaction), as well as those only qualitatively evaluated, would make any comparison very difficult. By using these pre-registered search criteria, we

Confidential: For Review

also ensured we would capture studies measuring parent satisfaction both as primary and as secondary outcomes. We included studies from all neonatal care level units and all healthcare settings, without excluding studies in low or middle-income settings. This was because definitions of neonatal care levels differ between different countries and healthcare settings, making them not easily comparable. Moreover, different levels of care are found within the same hospital settings. We excluded systematic reviews, entirely qualitative studies, grey literature (e.g. conference abstracts), studies only reporting protocols or abstracts and full reports not in English.

Two authors (SS, IA) independently double-screened titles and abstracts, reviewed full texts for eligibility and resolved any discrepancies with a third reviewer (JW). We extracted data using a pilot-tested, standardised data extraction form including study characteristics, interventions, outcomes and parent input into interventions' design. We assessed methodological quality with the Cochrane Collaboration risk of bias tool[12] for RCT and the ROBINS-I tool[13] for non-RCT.

We presented individual study aggregate data in a narrative synthesis, grouped studies into themes using a Grounded Theory Approach[14] and planned meta-analysis where data were appropriate for quantitative synthesis.

Patient involvement

This review was conceived in response to the clinical need identified by parents with neonatal care experience; a partnership including families with experience of preterm birth identified "what emotional and practical support improves attachment and bonding, and does the provision of such support improve outcomes for premature

babies and their families?" as a top 10 research priority[15]. Additionally, this review was conceived as part of planning a wider project to pilot a neonatal intervention, with parents' full input. Patients were not directly involved in the design, conduct, reporting or dissemination plans of our research.

RESULTS

We identified 8362 studies for screening and assessed 73 full text articles for eligibility (Figure 1). A total of 32 studies describing interventions that measured parent satisfaction in neonatal care as an outcome met the inclusion criteria, reporting data from over 2866 parents, 1 study did not report number of parents. Our analysis included 10 RCT and 22 non-RCT: 3 cohort trials, 18 unspecified designs and 1 implementation project. We classified the unspecified non-RCT into 2 types, depending on how they defined their control groups and how they evaluated parent satisfaction (eTable 1).

1. *"Unit- level effect":* Studies that assessed parent satisfaction during a period of routine care (control group) and introduced the intervention at a later time, with a different group of parents. In these studies improvement in parent satisfaction was evaluated between different parent groups, on a *unit level.*
2. *"Group level effect":* Studies that formed intervention and control groups using convenience sampling during the same time period. Both groups (or sometimes only the intervention group) had satisfaction measured after the intervention period (post intervention testing). Baseline parent satisfaction was also measured in both groups (pre intervention testing) in some studies. Improvement in parent satisfaction was demonstrated either by comparing

Confidential: For Review Only

outcomes between intervention/control groups following the intervention, or in comparison with the pre-intervention data.

Parent participants included mothers (14 studies), mothers and fathers (10 studies) or were not specified (7 studies). One study defined parent participants as a dyad of the mother with her designated support person. Median parent sample size was 63, ranging 7-482. This was higher for RCT (108 studies) compared to non-RCT (61 studies).

Study participants included parents of babies across the full range of gestations (23-42 weeks). Overall, 24/32 (75%) of studies involved preterm infants, 5/32 (16%) term infants and 7 studies did not state the gestational age of infants involved. Most studies (19, 59%) involved only preterm infants (up to 37 weeks); only 1 study (3%) involved only term infants and 5 studies (16%) involved both preterm and term infants. Preterm infants were included in 44% of RCT, versus 63% of non-RCT.

Most studies were reported as conducted in level III neonatal units (17 studies), followed by level not stated (9 studies), level II-III (3 studies), level II (2 studies) and level I (1 study). Definitions of neonatal levels of care are not standardised but vary across different countries; none of the included studies have explicitly stated which definition applies to them.

eTable 1 shows the key characteristics of included studies. eTable 1 includes a description of each study's parent and infant sample, study design and intervention,

https://mc.manuscriptcentral.com/bmjpo

Confidential: For Review Only

outcome measures (timing and methods), results, parent input into intervention design and study impact on parent satisfaction.

Parent satisfaction

Outcome measures: All 32 studies reported they measured parent satisfaction as an *a priori* outcome. Only one study confirmed this through a protocol. Overall 18/32 (56%) of studies (4/10, 40% RCT and 14/22, 64% non-RCT) reported a higher level of parent satisfaction associated with the intervention studied. Multiple different outcome measures within the domain of parent satisfaction were used; we grouped these into 4 categories: i) Parent satisfaction (no additional description); ii) Parent satisfaction with NICU care; iii) Parent satisfaction related to specific components such as communication, staff or information; iv) Parent satisfaction with a specific intervention.

Timing of measurement: Parent satisfaction was mostly measured *'during infant admission only'* (24 studies; between 1-4 times), followed by *'after infant discharge only'* (5 studies; 1 time) and '*both during admission and after discharge'* (3 studies; between 1-3 times). In the majority of studies (19/32, 59%) no pre-intervention parent satisfaction measurements were conducted in the same parent groups with available post-intervention data (ie paired parent data for satisfaction levels did not exist). Instead, impact of interventions was determined comparing intervention/control group measurements in different time periods (eTable 1).

Confidential: For Review Only

Method of measurement: Parent satisfaction was assessed using 32 different methods: 29 different questionnaires, 2 different single questions, and by structured interview in 1 study; in total 334 different questions were used to assess parent satisfaction. Only 6/29 (21%) of questionnaires were reported to be fully validated (both content validation and reliability testing); 23/29 (79%) questionnaires were partially or completely unvalidated. The most commonly used questionnaire was the validated *Neonatal Index of Parent Satisfaction (NIPS)*[16] questionnaire (3 studies).

Interventions and impact on parent satisfaction

We grouped included studies into 5 intervention themes: parent involvement (14 studies); information provision/communication (8 studies); clinical care (7 studies); parent emotional support (2 studies); other (1 study). Parent involvement interventions were more commonly assessed in RCT compared to non-RCT . We categorised interventions *as effective* or *not effective* based upon whether a statistically significant difference between intervention and control groups was reported for parent satisfaction (Table 1). None of the studies reported significantly lower parent satisfaction in the intervention group compared to the control group. We classified studies as *unclear if effective* if they included small sample numbers or if statistical analysis was not performed. Finally, we highlighted studies where *only the intervention group was assessed and only post-intervention,* where comparison to a control group was not possible.

Overall, 18/32 studies (56%) reported higher parent satisfaction in the intervention group; 4/10 RCT and 14/22 non-RCT. The intervention theme where higher

Confidential: For Review Only

271 satisfaction was most consistently reported was parent involvement (10/14 studies).

272 Due to the large heterogeneity of outcome measure scales a quantitative synthesis and

273 meta-analysis was not possible.

**1. Parent involvement**	**Outcome**
More NICU access, parents on WRs, Education (De Bernardo et al, Italy, 2017)	Effective
More NICU access, care involvement, education (Bastani et al, Iran, 2015) **RCT**	Effective
Newborn Individualised Developmental Care and Assessment Program (NIDCAP) (Wielenga et al, Netherlands, 2006)	Effective
Kangaroo care (Legault and Goulet, Canada, 1995)	Effective
Rooming-in care (Kazemian et al, Iran, 2016)	Effective
Single-family NICU rooms (Stevens et al, USA, 2011)	Effective
Parental Presence at Clinical Bedside Rounds (Abdel-Latif et al, Australia, 2015) **RCT**	Effective
Family-centered rounds (Voos et al, USA, 2011)	Effective
Infant Progress Charts filled by parents and 3 Care Planning Meetings (Penticuff and Arheart. USA, 2005)	Effective
Education re: pain management (Franck et al, UK, 2011) **RCT**	Effective
Open Unit policy: 24/7 NICU access (Voos and Park, USA, 2014)	Unclear if effective
Touch and massage for 7 days (Livingston et al, USA, 2009) **RCT**	Unclear if effective
a. Massage with auditory, tactile, visual, and vestibular stimulation<br>b. Kangaroo care (Holditch-Davis et al, USA, 2013) **RCT**	Not effective
Individualised, developmentally supportive family-centered care interventions (Byers et al, USA, 2006)	Not effective

274

**2. Information provision / communication**	**Outcome**
Internet-based education (Kadivar et al, Iran, 2017)	Effective
Daily SMS from Electronic Patient Record (Globus et al, Israel, 2016)	Effective
Staff education, staff contact card given to parents, staff poster at NICU reception (Weiss et al, USA, 2010)	Effective
Provision of taped conversations with neonatologists to mothers (Koh et al, Australia, 2007) **RCT**	Effective

Clinical staff enter updates in baby diary (Van de Vijver and Evans, UK, 2015)	Unclear if effective
Detailed information provided during consenting (Broyles et al, USA, 1992) **RCT**	Unclear if effective
Sharing information obtained from parent interviews with the primary NICU provider (Clarke-Pounder et al, USA, 2015) **RCT**	Not effective
Daily parent update letter from Electronic Patient Record (Palma et al, USA, 2012)	Only the intervention group was assessed and only post-intervention

**3. Clinical care**	**Outcome**
a. Headbox oxygen for respiratory distress<br>b. CPAP for respiratory distress (Foster et al, Australia, 2008)	Effective
Co-bedding infants in incubators (prospective) (Byers et al, USA, 2003)	Effective
Co-bedding infants in incubators (retrospective) (Polizzi et al, USA, 2003)	Effective
Palliative care (Petteys et al, USA, 2015)	Unclear if effective
Five potentially better practices in the area of discharge planning (Mills et al, USA, 2006)	Unclear if effective
Clinical Nurse Specialist/ neonatal practitioner team care (Mitchell-DiCenso et al, Canada, 1996) **RCT**	Not effective

**4. Parent emotional support**	**Outcome**
Narrative writing (Kadivar et al, Iran, 2017)	Effective
Listening visits (Segre et al, USA, 2013)	Only the intervention group was assessed and only post-intervention
Tele-rounding robot, off-site neonatologist (Garingo et al, USA, 2016)	Only the intervention group was assessed and only post-intervention

**5. Other**	**Outcome**
Free Parking (Northrup et al, USA, 2016) **RCT**	Not effective

Table 1. Interventions in themes

Legend: *The colours illustrate each intervention's reported effect on parent satisfaction.* *Green (intervention effective):* *Parent satisfaction was reported to be statistically significantly higher in the intervention group;* *Red (intervention not effective):* *Parent satisfaction was not reported to be statistically significantly different in the intervention group;* *Yellow (unclear if effective):* *Small study numbers and/or no statistical analysis performed);* *Grey (Only the intervention group was assessed and only post-intervention).* ***RCT:*** *Randomised Controlled Trial*

Parent input into design of interventions

Five studies (5/32, 16%) reported involving parents in intervention design, of which 2 reported improvement of parent satisfaction. The number of included studies was too small to estimate any effect of parent co-design on the success of interventions at study level.

Methodological quality

For the majority of RCT, key study characteristics, such as randomisation, allocation concealment and blinding of outcome assessment, were either not stated or unclear (Figure 2). Only one RCT had an available study protocol (retrospectively registered) and none described blinding of study participants and/or personnel. All RCT scored a high/unclear risk of bias in at least 4/6 Cochrane tool categories, except for one, which scored a high/unclear risk in 3/6 categories.

Confidential: For Review Only

We assessed 21/22 non-RCT studies using the ROBINS-I tool (13), excluding the implementation project. All 21 studies were assessed as having an overall *serious* risk of bias and 7/21 of studies (33%) were further categorised as having *critical* risk of bias (Figure 3). Blinding of participants, personnel and outcome assessment was poorly reported across all non-RCT and no study reported a published study protocol. None of the included non-RCT measured or corrected for important parent/infant confounding variables, or other relevant neonatal unit co-interventions taking place at the same time as the intervention.

We were unable to use the *Standards for Reporting Implementation Studies (StaRI) Statement Tool*[17] for assessing the implementation project, as the reporting was incomplete.

There was no association between methodological quality assessments and the studies' reported effect on parent satisfaction. All 4/10 RCT that reported a higher level of parent satisfaction associated with their intervention, scored a high/unclear risk of bias in at least 4/6 Cochrane tool categories, one of which scored high/unclear *risk* in all categories. Out of the 14/22 non-RCT reporting an improved parent satisfaction, two were deemed to be at *critical risk* of bias on the ROBINS- I tool, whilst the rest we assessed to be at *serious risk* of bias.

DISCUSSION

Parent satisfaction with neonatal care is increasingly recognised as an important measure of parent experience and is being used to evaluate hospitals and healthcare

https://mc.manuscriptcentral.com/bmjpo

Confidential: For Review Only

providers; use of interventions to improve parent satisfaction in neonatal units is increasing. This is the largest review of interventions where an outcome was parent satisfaction with neonatal care and includes 32 studies. We find low quality evidence that interventions targeting ‘parent involvement’ may improve parent satisfaction with neonatal care, but this result must be interpreted cautiously in view of the high risk of bias in included studies.

Overall, our review highlights the complexity of evaluating parent satisfaction. As a multidimensional construct, parent satisfaction can be affected just as much by interventions directly relating to infant care (eg. Kangaroo care) as well as interventions relating to neonatal care facilities (eg. Free parking). By grouping included interventions into themes (Table 1) we have highlighted the variety of interventions available, as well as the majority of interventions being those relating to ‘parent involvement’.

A key reason for only selecting parent satisfaction as the outcome of interest was to focus on a single component of parent experience, in order to reduce outcome heterogeneity and allow direct comparison. Despite this approach, the key methodological limitation identified in this review was inconsistency in how parent satisfaction is defined and measured; it is notable that the majority of questionnaires (23/29) lack validation. In keeping with neonatal studies more widely[18], this study confirms inconsistent outcome selection as a major source of research waste in neonatal studies examining parent experience, and further finds that there is limited involvement of parents in study design.

Confidential: For Review Only

Strengths of our review include identifying studies with both mother and father participants, inclusion of the full range of infant gestations and a wide range of interventions. We followed a pre-registered protocol and report this review in line with PRISMA guidelines[11]. To further aid direct comparison of interventions, we only included studies that evaluated parent experience using ≥1 quantitative outcome of parent satisfaction. One limitation of this approach is that by excluding studies which evaluated parent experience using other measures (e.g. stress, anxiety and depressions scales) we are unable to comment on interventions that targeted these other components of parent experience.

Another limitation is that we have only included studies in the English language, due to resource and time constraints. By not including studies in other languages, it is possible our results are more focused on work conducted in specific countries. Furthermore, we acknowledge that much of the research in parent experience is qualitatively evaluated. By restricting our review to studies where ≥1 quantitative outcome of parent satisfaction is measured, we have not included any interventions with solely qualitative outcomes. This was in an attempt to enable direct comparison of interventions, which has previously not been possible in any published review. By not including studies evaluated by qualitative measures only, it is possible our results are more focused on a particular type of interventions where quantitative evaluation would be preferable and/or easier. It also means we may not have included all studies ever conducted on a particular intervention, where some were only evaluated qualitatively, making some interventions appear more 'widespread' than others.

Confidential: For Review Only

Brett et al[19] systematically reviewed interventions aimed at improving the parent experience more widely, but only included parents of preterm infants. Their large number of outcome domains and heterogeneity of outcome measures (including studies that reported only qualitative outcomes) meant the authors we unable to draw firm conclusions about the efficacy of interventions and that comparison and meta-analysis was not possible. The majority of our review's studies have been published in the 7 years since the Brett review, highlighting the increasing interest in this area. However, despite including all gestations and focusing on a specific aspect of parent experience, heterogeneity in measurement of parent satisfaction meant we were also unable to conduct a quantitative synthesis. Inconsistency and lack of validation of instruments measuring parent satisfaction in neonatal care (specifically with family-centred care) has previously been highlighted by Dall'Oglio et al[20].

Although 31% of included studies were RCT, all were assessed as having a high risk of bias. Randomised controlled trials are traditionally considered the highest-ranking form of evidence, however it is worth considering whether such a design is feasible or desirable to evaluate interventions targeting parent satisfaction. Parents in neonatal care talk to each other, compare notes and invariably create parent-support communities; hence it is inherently difficult to avoid contamination between parents receiving an intervention and those who are not, meaning that blinding of parents or health professionals is near impossible. Furthermore, parent satisfaction is likely to be particularly susceptible to the Hawthorne effect[21], requiring longer-term follow up. These factors may explain the low number of RCT identified in our review and the high risk of bias seen in those that were included. In non-RCT studies, the main methodological concern is the degree to which unmeasured and uncontrolled

https://mc.manuscriptcentral.com/bmjpo

401 confounders may explain any differences seen between groups. The non-RCT studies
402 included in this review were classed as having either a serious or critical risk of bias.
403 The overwhelming majority of studies did not adequately report baseline variables or
404 report other interventions during the study period, making it impossible to assess
405 studies for selection bias or treatment bias. Furthermore, limitations such as
406 contamination bias and the Hawthorne effect affect non-RCT as well. Only two non-
407 RCT studies evaluated the outcome of interest (parent satisfaction) both before and
408 after the intervention, in the same group of parents *(group level effect),* with most
409 studies evaluating different parent groups pre and post intervention *(unit level effect).*
410 An inherent weakness of this latter approach is that it assumes parent satisfaction is a
411 static measure at the unit level, which is unlikely to be true. As a result of these
412 numerous important limitations identified across all included studies, we find only
413 low-quality evidence in support of interventions to improve parent satisfaction with
414 neonatal care, despite a majority of studies reporting a beneficial effect of
415 interventions. These limitations may explain the limited uptake of these interventions
416 by the wider neonatal community.
417
418 Changing neonatal unit practices to incorporate any new intervention requires robust
419 evidence. We demonstrate here that such evidence is not currently available for
420 improving parent satisfaction. We highlight the use of non-randomised study designs,
421 inconsistency in definition and measurement of parent satisfaction, the use of
422 unvalidated questionnaires, methodological limitations and a lack of parent
423 involvement as contributors. Our review empirically documents the extent of these
424 issues in studies that use quantitative parent satisfaction surveys, and their
425 contribution to research waste in neonatology.

https://mc.manuscriptcentral.com/bmjpo

Confidential: For Review Only

Given the importance of parent satisfaction for both parent and offspring wellbeing, higher quality trials that involve parents, use standardised definitions and validated parent satisfaction measures are needed. Given the nature and challenges of the neonatal care environment and the limitations we have identified in existing research, a cluster trial may be the most appropriate study design to rigorously evaluate interventions to improve parent satisfaction with neonatal care.

CONCLUSIONS

Many interventions, commonly relating to parent involvement, are reported to improve parent satisfaction with neonatal care but inconsistency in definition and measurement of parent satisfaction and high risk of bias in all studies makes this low quality evidence. Standardised definitions and validated parent satisfaction measures are needed, as well as higher quality trials of parent experience, involving parents in intervention design.

What is already known on this topic

- Neonatal care significantly affects parents' mental health; parent satisfaction is increasingly being used as a parent experience measure
- Parent satisfaction is inversely related to parent stress; interventions improving parent satisfaction have the potential to reduce parent stress, improve parent-infant bonding and infant outcomes
- Use of interventions measuring parent satisfaction as an outcome in neonatal units is increasing, though few are formally evaluated and wider

Confidential: For Review Only

uptake is limited; it is not known the degree to which parents are involved in intervention design

What this study adds

- There is inconsistency in how parent satisfaction in neonatal care is defined and measured, and the majority of studies do not include parents in intervention design
- There is low quality evidence that interventions relating to parent involvement may improve parent satisfaction with neonatal care
- Standardised, validated measures of parent satisfaction and higher quality trials, involving parents in intervention design, are needed

DECLARATIONS

Conflict of interest disclosure

SS has received research grants from the National Institute of Health Research (NIHR), the NIHR CLAHRC NWL, Rosetrees Trust and CW+ charity. NM is Director of the Neonatal Data Analysis Unit at Imperial College London. In the last five years NM has served on the Board of Trustees of the Royal College of Paediatrics and Child Health, David Harvey Trust, Medical Women's Federation and Medact; and is a member of the Nestle Scientific Advisory Board. NM has received research grants from the British Heart Foundation, Medical Research Council, National Institute of Health Research, Westminster Research Fund, Collaboration for Leadership in Applied Health and Care Northwest London, Healthcare Quality Improvement Partnership, Bliss, Prolacta Life Sciences, Chiesi,

Confidential: For Review Only

Shire and HCA International; travel and accommodation expenses from, Nutricia, Prolacta, Nestle and Chiesi; honoraria from Ferring Pharmaceuticals and Alexion Pharmaceuticals for contributions to expert advisory boards, and Chiesi for contributing to a lecture programme. CG is funded by the United Kingdom Medical Research Council (MRC) through a Clinician Scientist Fellowship award. He has received support from Chiesi Pharmaceuticals to attend an educational conference; in the past 5 years he has been investigator on received research grants from Medical Research Council, National Institute of Health Research, Canadian Institute of Health Research, Department of Health in England, Mason Medical Research Foundation, Westminster Medical School Research Trust and Chiesi Pharmaceuticals. IA, JW, DB: None to declare.

Authors' contributions

SS and CG conceived this systematic review. The protocol was created by SS and CG. Searches were performed by SS and IA. All search results were reviewed by SS, and JW. Coding was completed by SS and JW. Data analysis was completed by SS. The first draft of the manuscript was written by SS; SS, CG and JW edited and reviewed the manuscript. All authors approved the manuscript. This article presents independent research supported by the National Institute for Health Research (NIHR) The views expressed in this publication are those of the authors and not necessarily those of the NHS, the NIHR or the Department of Health and Social Care.

Funding

This work is sponsored by Imperial College London and supported by a peer-reviewed National Institute of Health Research Doctoral Research Fellowship,

Confidential: For Review Only

awarded to SS (DRF-2017-10-172).

References

1. Neonatal Data Analysis Unit. Neonatal Data Analysis Unit Annual Report 2017, 2018. Available: https://www.rcpch.ac.uk/sites/default/files/2018-10/2018_nnap_report_on_2017_data_final_v8.pdf

2. Lefkowitz DS, Baxt C, Evans JR. Prevalence and Correlates of Posttraumatic Stress and Postpartum Depression in Parents of Infants in the Neonatal Intensive Care Unit (NICU). *J Clin Psychol Med Settings* 2010;17(3):230-7.

3. Shaw RJ, Bernard RS, DeBlois T et al. The Relationship Between Acute Stress Disorder and Posttraumatic Stress Disorder in the Neonatal Intensive Care Unit. *Psychosomatics* 2009;50(2):131-7.

4. Beck CT, Woynar J. Posttraumatic Stress in Mothers While Their Preterm Infants Are in the Newborn Intensive Care Unit: A Mixed Research Synthesis. *ANS Adv Nurs Sci* 2017;40(4):337-55.

5. Lee SK, O'Brien K. Parents as primary caregivers in the neonatal intensive care unit. *CMAJ* 2014;186(11):845-7.

6. Grace SL, Evindar A, Stewart DE. The effect of postpartum depression on child cognitive development and behavior: a review and critical analysis of the literature. *Arch Womens Ment Health* 2003;6(4):263-74.

7. Rocha G, Candeias L, Ramos M et al. Stress and satisfaction of mothers in neonatal intensive care. *Acta Med Port* 2011;24(2):157-66.

8. Lopez-Maestro M, Sierra-Garcia P, Diaz-Gonzalez C et al. Quality of attachment in infants less than 1500g or less than 32 weeks. Related factors. *Early Hum Dev* 2016;104:1-6.

Confidential: For Review Only

9. Charpak N, Tessier R, Ruiz JG et al. Twenty-year Follow-up of Kangaroo Mother Care Versus Traditional Care. *Pediatrics* 2017;139(1):e20162063.

10. PROSPERO database. Available: http://www.crd.york.ac.uk/prospero/display_record.asp?ID=CRD42016042110

11. Moher D, Liberati A, Tetzlaff J et al. Preferred reporting items for systematic reviews and meta-analyses: the PRISMA statement. *J Clin Epidemiol* 2009;62(10):1006-12.

12. Higgins JP, Altman DG, Gotzsche PC et al. The Cochrane collaboration's tool for assessing risk of bias in randomised trials. *BMJ* 2011;343:d5928.

13. Sterne JA, Hernan MA, Reeves BC et al. ROBINS-I: a tool for assessing risk of bias in non-randomised studies of interventions *BMJ* 2016;355:i4919.

14. Green JTN. Qualitative Methods for Health Research. SAGE, 2014.

15. Duley L, Uhm S, Oliver S et al. Top 15 UK Research Priorities for Preterm Birth. *The Lancet* 2014;383(9934):2041-2042.

16. Mitchell-DiCenso A, Guyatt G, Paes B et al. A new measure of parent satisfaction with medical care provided in the neonatal intensive care unit. *J Clin Epidemiol* 1996;49(3):313-318.

17. Pinnock H, Barwick M, Carpenter C et al. Standards for Reporting Implementation Studies (StaRI) statement. *BMJ* 2017;356:i6795.

18. Webbe JWH, Ali S, Sakonidou S et al, Inconsistent outcome reporting in large neonatal trials: a systematic review, *Arch Dis Child Fetal Neonatal Ed* 2019 doi: 10.1136/archdischild-2019-316823. [Epub ahead of print]

19. Brett J, Staniszewska S, Newburn M et al. A systematic mapping review of effective interventions for communicating with, supporting and providing information to parents of preterm infants. *BMJ Open* 2011;1(1):e000023.

Confidential: For Review Only

20. Dall'Oglio I, Mascolo R, Gawronski O et al. A systematic review of instruments for assessing parent satisfaction with family-centred care in neonatal intensive care units. *Acta Paediatr* 2018;107:391-402.

21. McCambridge J, Witton J, Elbourne DR. Systematic review of the Hawthorne effect: new concepts are needed to study research participation effects. *J Clin Epidemiol* 2014;67(3):267–277.

Figure / Table Legends

Figure 1: PRISMA Flow diagram of selected studies

Figure 2. Cochrane Collaboration Risk of Bias tool assessment (RCT)

Legend: Green- low risk of bias; Yellow- unclear risk of bias; Red- high risk of bias

Figure 3. ROBINS-I risk of bias assessment (Non-RCT)

Table 1. Interventions in themes

Legend: *The colours illustrate each intervention's reported effect on parent satisfaction*. *Green (intervention effective): Parent satisfaction was reported to be statistically significantly higher in the intervention group*; *Red (intervention not effective): Parent satisfaction was not reported to be statistically significantly different in the intervention group; Yellow (unclear if effective): Small study numbers and/or no statistical analysis performed); Grey (Only the intervention group was assessed and only post-intervention).* ***RCT:*** *Randomised Controlled Trial*

Online supplementary files

File 1. OVID MEDLINE search strategy

Confidential: For Review Only

eTable 1. Included studies by study design- Randomised controlled trials (RCT) and non-RCT

Legend: *Number in last column illustrates each intervention's reported effect on parent satisfaction:* *1. Parent satisfaction was statistically significantly higher in the intervention group*; *2. Parent satisfaction was not reported to be statistically significantly different in the intervention group; 3. Unclear if parent satisfaction improved (small study numbers and/or no statistical analysis performed); 4. Only the intervention group was assessed and only post-intervention*

Research checklist

PRISMA checklist

Confidential: For Review Only

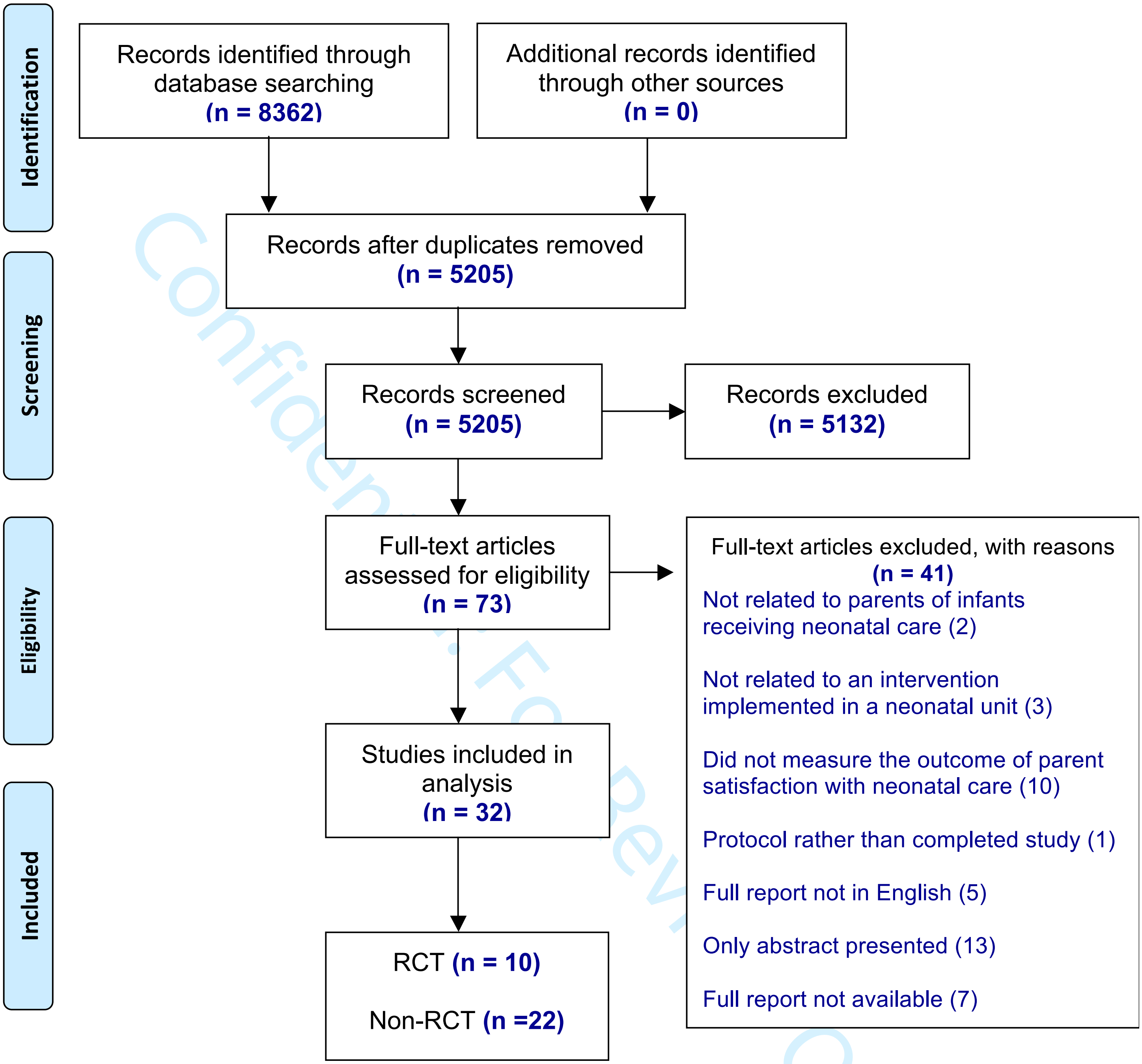

Confidential: For Review Only

Risk of Bias (Cochrane)

Author by publication year	Random sequence generation	Allocation concealment	Blinding of participants and personnel	Blinding of outcome assessment	Incomplete outcome data	Selective reporting
1. Northrup (2016)	?	+	-	?	+	?
2. Abdel-Latif (2015)	+	+	-	-	-	?
3. Bastani (2015)	?	?	-	?	+	?
4. Clarke-Pounder (2015)	?	?	-	?	+	?
5. Holditch-Davis (2013)	+	+	-	+	?	?
6. Franck (2011)	-	?	-	?	-	+
7. Livingston (2009)	?	?	-	?	+	?
8. Koh (2007)	?	?	-	?	?	?
9. Mitchell-DiCenso (1996)	+	?	?	?	?	?
10. Broyles (1992)	?	?	-	+	+	?

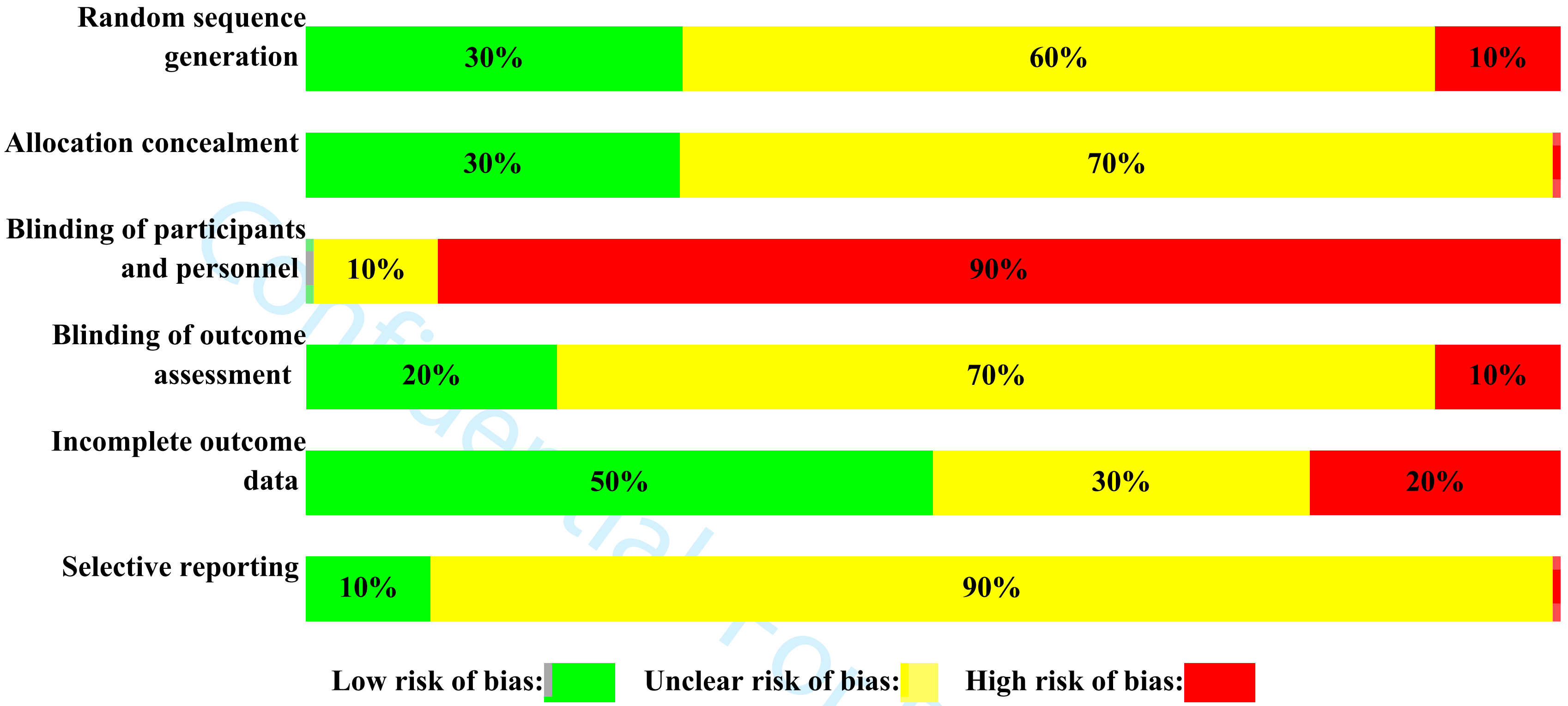

Confidential: For Review Only

Risk of Bias (ROBINS-I)

Author by publication year	Bias due to confounding	Bias in selection of participants into the study	Bias in classification of interventions	Bias due to deviations from intended interventions	Bias due to missing data	Bias in measurement of outcomes	Bias in selection of the reported result	OVERALL risk of bias
1. De Bernardo (2017)	SERIOUS	LOW	LOW	SERIOUS	LOW	SERIOUS	SERIOUS	SERIOUS
2. Kadivar (2017) *Internet-based education*	SERIOUS	LOW	LOW	SERIOUS	LOW	SERIOUS	MODERATE	SERIOUS
3. Kadivar (2017) *Narrative writing*	SERIOUS	SERIOUS	LOW	SERIOUS	LOW	SERIOUS	MODERATE	SERIOUS
4. Garingo (2016)	CRITICAL	LOW	LOW	SERIOUS	SERIOUS	SERIOUS	SERIOUS	CRITICAL
5. Globus (2016)	SERIOUS	LOW	LOW	NO INFO	SERIOUS	SERIOUS	SERIOUS	SERIOUS
6. Kazemian (2016)	SERIOUS	NO INFO	LOW	SERIOUS	NO INFO	SERIOUS	SERIOUS	SERIOUS
7. Petteys (2015)	SERIOUS	LOW	LOW	SERIOUS	MODERATE	SERIOUS	MODERATE	SERIOUS
8. Van de Vijver (2015)	CRITICAL	LOW	LOW	SERIOUS	MODERATE	SERIOUS	MODERATE	CRITICAL
9. Voos (2013)	CRITICAL	LOW	LOW	SERIOUS	NO INFO	SERIOUS	SERIOUS	CRITICAL
10. Segre (2013)	CRITICAL	NO INFO	LOW	SERIOUS	SERIOUS	SERIOUS	MODERATE	CRITICAL
11. Palma (2012)	CRITICAL	NO INFO	LOW	SERIOUS	SERIOUS	SERIOUS	CRITICAL	CRITICAL
12. Stevens (2011)	SERIOUS	LOW	LOW	SERIOUS	SERIOUS	SERIOUS	MODERATE	SERIOUS
13.Voos (2011)	SERIOUS	LOW	LOW	SERIOUS	SERIOUS	SERIOUS	MODERATE	SERIOUS
14. Weiss (2010)	SERIOUS	LOW	LOW	SERIOUS	LOW	SERIOUS	MODERATE	SERIOUS
15. Foster (2008)	SERIOUS	CRITICAL	LOW	SERIOUS	SERIOUS	SERIOUS	MODERATE	CRITICAL
16. Byers (2006)	SERIOUS	LOW	LOW	SERIOUS	SERIOUS	SERIOUS	MODERATE	SERIOUS
18. Wielenga (2006)	SERIOUS	LOW	LOW	SERIOUS	LOW	SERIOUS	MODERATE	SERIOUS
19. Penticuff (2005)	SERIOUS	LOW	LOW	SERIOUS	LOW	SERIOUS	MODERATE	SERIOUS
20. Byers (2003)	SERIOUS	LOW	LOW	SERIOUS	LOW	SERIOUS	SERIOUS	SERIOUS
21. Polizzi (2003)	SERIOUS	MODERATE	LOW	SERIOUS	SERIOUS	SERIOUS	MODERATE	SERIOUS
22. Legault (1995)	SERIOUS	CRITICAL	LOW	CRITICAL	LOW	SERIOUS	MODERATE	CRITICAL

1. intervention$.ti,ab.
2. tool$.ti,ab.
3. way$.ti,ab.
4. updat$.ti,ab.
5. method$.ti,ab.
6. information.ti,ab.
7. sms.ti,ab.
8. implement$.ti,ab.
9. 1 or 2 or 3 or 4 or 5 or 6 or 7 or 8
10. bab$3.mp.
11. preterm$.ti,ab.
12. pre term.ti,ab.
13. premature.ti,ab.
14. postterm.ti,ab.
15. post term.ti,ab.
16. infant$.ti,ab.
17. newborn$.ti,ab.
18. exp Infant, Newborn/
19. 10 or 11 or 12 or 13 or 14 or 15 or 16 or 17 or 18
20. neonatal intensive care.ti,ab.
21. neonatal unit$.ti,ab.
22. NICU.ti,ab.
23. SCBU.ti,ab.
24. neonatal itu.ti,ab.
25. special care baby unit$.ti,ab.
26. neonat$.ti,ab.
27. Intensive Care Units, Neonatal/
28. Intensive Care Units/
29. Critical Care/
30. Neonatal Nursing/
31. 20 or 21 or 22 or 23 or 24 or 25 or 26 or 27 or 28 or 29 or 30
32. parent$.ti,ab.
33. mother$.ti,ab.
34. father$.ti,ab.
35. exp Parents/

36. 32 or 33 or 34 or 35
37. satisfaction.ti,ab.
38. experience$.ti,ab.
39. Patient Satisfaction/
40. personal satisfaction/
41. communicat$.ti,ab.
42. exp Communication/
43. Health Communication/
44. Information Dissemination/
45. 37 or 38 or 39 or 40 or 41 or 42 or 43 or 44
46. 9 and 19 and 31 and 36 and 45

Confidential: For Review Only

Randomised controlled trials (RCT) by publication year

Author (Date), Country	Parent Gender/ sample size	Infants Gestation age (GA) in weeks /NICU level	Study design	Intervention	Outcome measures	Timing of measurement	Method of measurement	Results	Parent co-design?	Improved parent satisfaction?
1. Northrup et al. (2016), USA	Mothers and fathers /116	<28 / level III	Randomised controlled trial	Intervention: **Free Parking (FP).**<br><br>Parents received 7 parking vouchers at a time (value: $10/each) and continued to receive vouchers until infant discharge. Each voucher allowed free entry and exit for 24hr.<br><br>Control: Parents received the standard care and did not receive vouchers.	***Parent satisfaction with NICU care***	After babies were discharged (once)<br><br>- During the first high-risk-infant clinic visit after discharge<br><br>No pre-intervention parent satisfaction data available for comparison.	*Satisfaction questionnaire*<br><br>Validation: No content validity or reliability testing reported.<br><br>11 questions<br><br>- Seven items were summed (score 7-35) to measure "Support" (e.g., information sharing).<br><br>- Three items measured "Emotional Connection" to the infant (score 3-15)<br><br>- One item assessed "family involvement in infant care" (responses: not enough-just right-too much).<br><br>Greater scores indicated higher perceived support, connection and satisfaction.	**The groups did not differ significantly with respect to satisfaction.**<br><br>**Interv Control p-value**<br>NICU support<br>**Mean (SD)** 30(2.7) 28.7(3.7) 0.07<br><br>Emotional connection<br>12.3(1.7) 12.3(1.7) 0.96<br><br>Family involvement<br>"Just right"<br>81.4% 85% 0.07	**No**	**2**
2. Abdel-Latif et al. (2015), Australia	Mothers and fathers /63	25-42 / level III	Cross-over Randomised Controlled Trial	Intervention: **Parental Presence at Clinical Bedside Rounds (PPCBR).**<br><br>Parents attended bedside clinical rounds. Parents had opportunity to ask questions about their baby's condition and management.<br><br>Control: Parents received the standard care with no parental presence at bedside clinical rounds.	***Parent satisfaction assessed by questions of 3 domains:***<br>*1. Knowledge and understanding*<br>*2. Communication and collaboration*<br>*3. Privacy and confidentiality*	During babies' admission (once)<br><br>- At the end of each study arm, separated by a washout period<br><br>- No pre-intervention parent satisfaction data available for comparison	*Satisfaction questionnaire*<br><br>The authors stated "the research team designed the questionnaire".<br><br>Validation: No content validity or reliability testing reported.<br><br>Number and format of questions: not stated	**PPCBR had significantly higher adjusted mean (95% CI) scores for some questions from domains 1 and 2.**<br><br>Domain 3 was comparable between the two study groups.<br><br>**Interv Control p-value**<br>Domain 1 question:<br>*"I have received adequate information about my baby's condition and management"*<br>**Mean** 4.321 3.947 0.03<br><br>Domain 2 questions:<br>*"In the last week I have been able to communicate effectively with my baby's healthcare team"*<br>**Mean** 4.407 4.250 0.05<br><br>*"In the last week I have collaborated with my baby's healthcare team in the*	**No**	**1**

Confidential: For Review Only

								planning of care for my baby"<br>**Mean** 3.843 3.426 0.02<br><br>*"In the last week I have been able to ask the healthcare team questions about my baby's care"*<br>**Mean** 4.642 4.259 0.004		
3. Bastani et al, (2015), Iran	Mothers /100	30-37 Mean (SD)<br><br>Control: 33.90 (2.33)<br><br>Interv: 34 (1.9)<br><br>/ level not stated	Randomised Controlled Trial (block randomisation)	<u>Intervention:</u> **Family-centered Care (FCC).**<br><br>Mothers allowed access to their baby at any time, participated in the care process and were provided with information about neonatal care.<br><br><u>Control:</u> Mothers received the standard care where they were only allowed to be present at the time of the infant's entry to the neonatal care unit, and were only routinely informed.	***Maternal satisfaction*** **relating to three themes:**<br>1. Parental presence<br>2. Participation in neonatal care<br>3. Information about neonatal care	During babies' admission (twice)<br><br>- 24 hours after admission<br>- At the time of discharge	*Satisfaction questionnaire* (Validated)<br><br>A modified satisfaction questionnaire was used, based on a parental satisfaction instrument developed for measuring satisfaction in Paediatric intensive care Units (PICU).<br><br>18 questions<br><br>Graded 0 (very dissatisfied) to 4 (very satisfied).<br><br>The overall satisfaction rate was classified based on the mean scores (score<50%, between 75-50% and > 75%).	**In the FCC group, pre and post intervention difference in maternal satisfaction was statistically significant p<0.001**<br><br>**Interv Control p-value**<br>**Mean (SD)**<br><br>**At 24 hr**<br>22.36(8.90) 22.06(9.77) 0.87<br><br>**At discharge**<br>59.28(6.86) 30.18(14.09) <0.01	**Unclear**<br><br>Mothers determined the reliability of the satisfaction tool and approved the educational pamphlet. Authors did not report if mothers had direct input in the intervention design.	1
4. Clarke-Pounder et al. (2015), USA	Mothers and fathers /19 families	23-39 / level III	Randomised Controlled Trial	<u>Intervention:</u> **Sharing information obtained from parent interviews with the primary NICU provider.**<br><br>Parents interviewed using the *NICU- adapted Decision Making Tool (N-DMT).* Information obtained was placed in the electronic medical record (EMR) and shared with the primary neonatal provider via email. Daily rounds on all infants were audio-recorded for 3 days after enrollment to see if information from the N-DMT was incorporated into daily care planning.<br><br><u>Control:</u> The content of a recent social work note was communicated with the primary provider via e-mail, creating an attentional control group.	***Parent satisfaction with care***	During babies' admission (once)<br><br>- 2 weeks after study entry<br><br>No pre-intervention parent satisfaction data available for comparison.	*Satisfaction questionnaire*<br><br>A *NICU- adapted Decision Making Tool (N-DMT)* – specific questionnaire was used.<br><br><u>Validation:</u> Partially reported. Authors stated reliability testing took place; no information on content validity provided.<br><br>8 questions: e.g."My baby's doctors considered my goals and hopes for my baby during decision-making".<br><br>Likert scale (1 strongly agree-4 strongly disagree). Total N-DMT score range 8–32.	**There was no significant difference in satisfaction with care as measured by the N-DMT scale between the control group and intervention groups in a univariable model or multiple variable model controlling for gestational age.**<br><br>**Interv Control**<br>**Median** (range)<br>26(15–28) 28.8(19–32)<br><br>**No p-value reported**<br><br>There was, however, a pattern of decreased satisfaction with care among the intervention group compared to the control group across the N-DMT-specific survey questions, although the differences were not statistically significant.	**Yes**<br><br>Information obtained from parents using the N-DMT was placed in the electronic medical record (EMR) and shared with the primary NICU provider via email (forming the intervention)	2
5.Holditch-Davis et al. (2013), USA	Mothers /208	Preterm infants	Randomised controlled trial	<u>Interventions:</u> **1. Mothers were taught how to massage infants with auditory, tactile, visual,**	**1.** ***Parent (mother) satisfaction with the***	During admission period and post discharge	*Satisfaction questionnaire*<br><br>The questionnaire was designed by the study team.	**No significant differences occurred between the groups.**	**No**	2

Confidential: For Review Only

https://mc.manuscriptcentral.com/bmjpo

		Mean (SD)<br><br>Overall group 27.2 (3.0)<br><br>/ 4 centres, levels II-III	3 groups (2 intervention and 1 control)<br><br>Post-intervention testing only.	**and vestibular stimulation (ATVV intervention)**<br>**2. Kangaroo care**<br><br><u>Control:</u> Attention control group. Mothers spent a similar amount of time with the study nurse discussing the equipment needed for preterm infant care at home. Study nurses provided education and support for all three groups. Mothers were not prevented from engaging in interventions of the other groups but did not receive formal education from the study nurse on the other interventions.	***intervention***<br><br>***2. Satisfaction with the helpfulness of the study nurse***<br><br>***3. Whether the mother would recommend the study to others and the degree of change in the mother as a person and as a mother as a result of being in the study.***	- At the time of discharge<br><br>- At 2 months corrected age<br><br>No pre-intervention parent satisfaction data available for comparison.	<u>Validation:</u> Partially reported. Authors stated reliability testing took place; no information on content validity provided.<br><br><u>26 questions:</u> relating to three dimensions of satisfaction: efficacy, caring, and technical quality.<br><br>Likert (1 least satisfied-5, 5 most satisfied)	Mothers in all three groups were satisfied with the intervention (mean scores of 3.3 or higher on a 5-point scale) and the helpfulness of the nurse (mean scores of 4.6 or higher on a 5-point scale).		
6. Franck et al. (2011), UK	Mothers and fathers /169	Mean (SD)<br><br>Control: 31.94 (5.17)<br><br>Interv: 29.40 (3.17)<br><br>/4 centres, level III	Cluster Randomised Controlled Trial	<u>Intervention:</u> **Increasing parental involvement in infant pain management in the NICU.**<br><br>Parents received a booklet providing evidence-based information about pain and comforting infants in the NICU setting. Parents received 2 visits from a research nurse showing them how to apply the comforting techniques described in the booklet.<br><br><u>Control:</u> As part of usual care, parents in both the intervention and control groups received a detailed booklet with generic information about NICU care. Parents in the control group also received 2 visits from a research nurse listening to what parents had to say about their NICU experience (attention placebo).	At baseline:<br><br>***1. Parent satisfaction with NICU care***<br><br>One week after the intervention:<br><br>***1. Satisfaction with information about pain control***<br><br>***2. Satisfied nurses make infant comfortable***<br><br>***3. Satisfied pain medicines help infant***	During babies' admission (twice)<br><br>-At baseline (within 3 to 7 days of admission)<br><br>- 1 week after the intervention	*Individual questions*<br><br><u>Validation:</u> No content validity or reliability testing reported.<br><br>1. At baseline:<br><br>Parent satisfaction was measured by 1 question: *"Satisfaction with NICU care"* (1 very satisfied-6 very unsatisfied) as part of the baseline parent characteristics questionnaire.<br><br>2. One week after the intervention:<br><br>Three questions using the word "satisfied' were selected from the validated *Parent Attitudes About Infant Nociception (PAIN)* survey (Likert scale 1 very satisfied-6 very unsatisfied)	**At baseline: there was no significant difference in satisfaction between intervention and control group**<br><br>**Interv** **Control**<br>**Mean (SD)** 1.45(0.71) 1.51(0.76)<br><br>**p-value** missing<br><br>**1 week after the intervention: Intervention parents were more satisfied with the information about pain control received than control parents.**<br><br>**Interv** **Control**<br>**Mean (SD)** 2.10(0.97) 3.28(1.27)<br>**p-value** < 0.001	**Yes**<br><br>The booklet was reviewed by 12 parents of infants who had been cared for in NICUs in the United Kingdom.	**1**
7.Livingston et al. (2009), USA	Mothers /12	Mean (SD)<br><br>Control:	Randomised Controlled Trial	<u>Intervention:</u> **Touch and massage.**<br><br>Mothers attended a 1hr massage class taught by a	***1. Caregiver (mother) satisfaction with their infant's care***	During babies' admission (three times)<br><br>- At baseline	*Satisfaction questionnaire*<br><br>Two questionnaires were developed by the research team.	**It is unclear in the report if specific between-group comparisons and statistical analysis were conducted.**	**No**	**3**

Confidential: For Review Only

		33.4 (6.4)<br><br>Interv:<br>38.5 (3.1)<br><br>/ level III		nurse CIMI (certified infant massage instructor) and were asked to participate in at least 3 bedside massage instruction sessions taught within the next week. Infants received massage for 7 consecutive days, from the mother or a CIMI. The touch procedure lasted 20 minutes.<br><br><u>Control:</u> Infants received all usual hospital services including medical care, physical and occupational therapy services and developmentally supportive nursing care.	**2. *Caregiver satisfaction with the neonatal unit and the massage therapist***	- Upon completing the 7-day massage program<br><br>- 1 month following intervention	<u>Validation:</u> No content validity or reliability testing reported.<br><br>-1st questionnaire (at baseline): a brief self-report questionnaire about caregiver satisfaction with their infant's care until that moment. No further details reported.<br><br>-2nd questionnaire (upon completing the 7-day massage program and 1 month following intervention): a 10-minute satisfaction questionnaire relating to infant's response and caregiver satisfaction with the neonatal unit and the massage therapist.<br><br><u>Number of questions:</u> not stated.<br><br>Likert scale (1 very dissatisfied-4 very satisfied).<br><br>Sample statements:<br>*'How satisfied do you feel giving massage to your infant?'; 'I feel that massage improved my infant's hospital stay.'*	<u>At baseline and day 7:</u><br>All caregivers were highly satisfied with the medical treatment their infant received.<br><br><u>At day 7 and 1 month follow-up:</u><br>All caregivers participating in the massage group reported high levels of satisfaction regarding their relationship with their infant and the massage program's impact on that relationship.<br><br>Slight improvements in satisfaction regarding time the caregiver spent with the infant and involvement in the infant's care were observed between day 7 and the 1-month follow-up (no further information reported).		
8. Koh et al. (2007), Australia	Mothers /200	Not stated / not stated	Randomised, Controlled Trial	<u>Intervention:</u> **Provision of taped conversations with neonatologists to mothers.**<br><br>The initial conversation and subsequent conversations of significance with a neonatologist were taped and analysed (for both groups). Mothers received a tape of each conversation and a tape recorder.<br><br><u>Control:</u> Usual care. Mothers were not given the tape or recorder.	***Satisfaction with conversations held with the neonatologist***<br><br>***Satisfaction with the tape***	During admission period and post discharge<br><br>- At 10 days<br><br>- At 4 months<br><br>- At 12 months<br><br>No pre-intervention parent satisfaction data available for comparison.	*Individual questions and a satisfaction scale*<br><br><u>Validation:</u> No content validity or reliability testing reported.<br><br><u>Number of questions:</u> not stated.<br><br>Likert scale (1-5 most satisfied)<br><br>Questions related to:<br>Satisfaction with amount and quality of information presented, doctors' communication skills, patient's participation in the conversation.<br><br>A satisfaction scale was used to assess:<br>Satisfaction with the tape	No differences were found between the two groups in satisfaction with conversations.<br><br>**Mothers of babies with a poor outcome in the tape group were, however, significantly more satisfied with the conversations:**<br><br>**Interv** **Control**<br>**Mean (95%CI)**<br>115(104-123.2) 100.5(94.1-109.4)<br><br>**p-value** 0.0051<br><br>Most (71-92%) of the mothers given the tapes stated that they helped their understanding, reminded them of what had been said, and helped their family to understand and recall information.	**No**	**1**

Confidential: For Review Only

9. Mitchell-DiCenso et al. (1996), Canada	Mothers and fathers/ 482	Mean (SD)<br><br>Interv: 35.1 (4.5)<br><br>Control: 35 (4.3)<br><br>/ level III	Randomised, Controlled Trial	Intervention: **Clinical Nurse Specialist/ neonatal practitioner team (CNS/NP) care.**<br><br>Infants of intervention parents were assigned to be cared for by the Clinical nurse special/neonatal practitioner CNS/NP team during the day and by paediatric residents during the night.<br><br>Control: Paediatric residents cared for infants of control parents around the clock. Neonatologists supervised both teams.	***Parent satisfaction with care***	During admission period and post discharge (twice)<br><br>- On 5th day after admission (full survey)<br><br>- After discharge over the phone (only questions related to satisfaction with discharge process)<br><br>No pre-intervention parent satisfaction data available for comparison.	*Satisfaction questionnaire (Validated)*<br><br>The study team developed and used the validated *Neonatal Index of Parent Satisfaction (NIPS)* questionnaire.<br><br>Number of questions: not stated.<br><br>NIPS score range (27-189); higher scores indicating greater satisfaction with care.	**No statistically significant difference between groups.**<br><br>**Interv Control p-value**<br>**NIPS Mean** 140 139 0.67<br><br>**Difference in means** 1.0, CI (-3.6-5.6)	**No**	**2**
10. Broyles et al. (1992), USA	Mothers /25	Mean (SD)<br><br>Control: 34 (4)<br><br>Interv: 33.4 (4)<br><br>/ level III	Randomised Controlled Trial	Intervention: **Detailed consent.**<br><br>Mothers were given information about mechanical ventilation. Detailed risk/benefit disclosure was provided both verbally and in writing.<br><br>Control:<br>Mothers were given a brief verbal description about mechanical ventilation supplemented with detailed verbal and written disclosure if desired by them (flexible consent).	***Maternal satisfaction with the information provided about mechanical ventilation***	During babies' admission (once)<br><br>- 24-48 hours after the intervention<br><br>No pre-intervention parent satisfaction data available for comparison.	*An interview* evaluating maternal satisfaction with the information provided about mechanical ventilation.<br><br>Validation: A psychiatrist with a special interest in interviewing techniques was consulted in designing and standardising this assessment.<br><br>A research nurse conducted the interview, "checking" each mother against one option regarding:<br>- Amount of information: Right amount-Too much-Too little<br><br>- Information made coping: More Difficult-Easier-No effect-Uncertain.	This study is measuring and comparing satisfaction with two different interventions (detailed vs flexible consent process), neither of which formally represent the usual routine care for all babies (no control).<br><br>**Small numbers. No data indicating statistical analysis conducted or evidence of statistically significant results.**<br><br>**Detailed Flexible**<br>**Right amount of information** 75% mothers 100%<br><br>**Too little information** 25% mothers<br><br>**Made coping easier** 67% mothers 69%	**No**	**3**

Confidential: For Review Only

Non-Randomised controlled trials (Non-RCT) by publication year

Author (Date), Country	Parents' gender/ sample Size	Infant Gestation age (GA) in weeks /NICU level	Study design	Intervention	Outcome measures	Timing of measurement	Method of measurement	Results	Parent co-design?	Improved parent satisfaction?
1. De Bernardo et al (2017), Italy	Mothers and Fathers /96	Mean (SD)<br><br>Control: 34.2 (5.25)<br><br>Interv: 32.7 (5.25)<br><br>/ level III	Non-randomized, prospective cohort pilot study<br><br>*Unit level effect*:<br>Two different time periods	Intervention: **FCC (Family-Centered Care).**<br><br>Parents had access to NICU for 8 hours/day. The NICU was widened and paediatric nurses taught parents procedures/practices for 10 days. Parents could observe clinical bedside rounds, hold meetings with the physicians, use the rooms and kitchen.<br><br>Control: Parents were permitted to visit their baby in NICU for 1 hour a day.	***Parent satisfaction relating to 3 specific domains:***<br><br>1. Knowledge and Understanding<br><br>2. Communication and Collaboration<br><br>3. Privacy and confidentiality	During babies' admission (once)<br><br>- At discharge (pre-FCC cohort and post-FCC cohort)<br><br>No pre-intervention parent satisfaction data available for comparison (different parent groups pre and post intervention).	*Satisfaction questionnaire.*<br><br>Validation: The authors state the survey *"was designed and validated by Abdel-Latif et al"*. No content validity or reliability testing reported in the original paper.<br><br>9 questions<br><br>3 questions: Related to adequate and timely information about the baby's condition.<br><br>3 questions: Related to communication and collaboration with the healthcare team.<br><br>3 questions: Related to respect of patient privacy.<br><br>Likert (1 strongly disagree-5 strongly agree)	**7/9 individual statements in the parent satisfaction questionnaire scored higher in the FCC compared to the NFCC (statistically significant difference).**<br><br>Example statement:<br>*"I have received adequate information about my baby's condition and management."*<br><br>**Interv** **Control**<br>**Median** 5 (3.45-5) 4 (3-5)<br><br>**p-value** <0.05	**No**	**1**
2. Kadivar et al. (2017), Iran	Mothers /68	<=30 – 36<br><br>/ level not stated	Non-randomised, Convenience sampling.<br><br>*Group level effect:*<br>Intervention/ control groups.<br><br>Pre and post-intervention testing.	Intervention: **Internet-based education.**<br><br>Mothers used an educational website set up by the research team (files and clips). Mothers could visit the website from 5:00-6:00 pm for 10 days. They were also allowed to use the website outside of the above hours and to report the duration of using the website to the researcher. Mothers had	***Maternal satisfaction***	During babies' admission (twice)<br><br>- Day 1 of intervention<br><br>- Day 10 of intervention	*Satisfaction questionnaire (Validated)*<br><br>The *"What Being The Parent of a Baby is Like-Revised" Questionnaire (WBPL- Revised)* was used. The original English version by Pridham and Chang was translated to Persian.<br><br>11 questions<br><br>Total satisfaction score range (11–99)	There was a significant difference in the mean score of satisfaction between cases and controls while the mean score of satisfaction increased in both groups.<br><br>**Comparison of the mean score between the two groups showed that the level of satisfaction was significantly higher in the case group versus the control group.**	**No**	**1**

https://mc.manuscriptcentral.com/bmjpo
Confidential: For Review Only

				to use the website at least 3 times during 10 days, each time for at least 30 min.<br><br><u>Control:</u> Mothers in the control group received the routine education provided in the NICU.				**Interv Control**<br>**before intervention**<br>**Mean** 81.62(13.50) 85.71(9.46)<br>**(SD)**<br>**p-value** 0.993<br><br>**after intervention**<br>**Mean** 93.88 (5.38) 90.12 (7.78)<br>**(SD)**<br><br>**p-value** 0.024		
3. Kadivar et al. (2017), Iran	Mothers /70	Mean (SD)<br><br>Control 31.6 (2.4)<br><br>Interv: 32.9 (3.1)<br><br>/ level not stated	Non-randomised, Convenience sampling.<br><br>*Unit level effect*:<br>Two different time periods	<u>Intervention:</u> **Narrative writing.**<br><br>Mothers did narrative writing at least 3 times until the 10th day of admission.<br><br><u>Control:</u> Mothers in the control group received the routine NICU treatment and care.	***Mothers' satisfaction with medical care provided by physicians, medical students, and nurses during neonatal admission to the NICU***	During babies' admission (twice)<br><br>- Day 3 of intervention<br><br>- Day 10 of intervention	*Satisfaction questionnaire (Validated)*<br><br>The *NIPS questionnaire* by Mitchell et al was used and translated to Persian.<br><br>24 questions (Likert scale)<br><br>Likert (1 always or not satisfied-7 never or completely satisfied). A higher score indicates more satisfaction.	**The satisfaction level of the mothers in the intervention group increased significantly during the study.**<br><br>The results of independent t test showed a significant difference in the satisfaction changes of the mothers on the 3rd and 10th day of NICU admission between intervention and control groups, indicating the effectiveness of narrative writing.<br><br>The results of paired t-test also showed a significant difference in the mean satisfaction level of the mothers between the 3rd and the 10th day in the intervention group.<br><br>**Interv Control**<br>**After intervention**<br><br>**Mean** 137 (15.2) 102.3 (25.6)<br>**(SD)**<br><br>**p-value** 0.001	**No**	**1**
4. Garingo et al. (2016), USA	Not stated /9	23-39 / level III	Non-randomised, Convenience sampling.<br><br>*Group level effect:*<br>Intervention/ control groups<br><br>Post-intervention group testing	<u>Intervention:</u> **Tele-rounding.**<br><br>Infants of intervention parents were cared for by an OFFSN (off site neonatologist) who was present via a remote-controlled robot. The OFFSN assessed infants via the robot's integrated stethoscope, with assistance from the nursing staff. During	***Satisfaction with telemedicine***	During babies' admission (once)<br><br>- At the time of discharge<br><br>No pre-intervention parent satisfaction data available for comparison.	*Satisfaction questionnaire*<br><br><u>Validation:</u> No content validity or reliability testing reported.<br><br><u>Number of questions:</u> not stated.<br><br>Likert (1 excellent-5 very poor).	**Only the intervention group was assessed and only post-intervention.**<br><br>The authors reported that the parents surveyed were *"satisfied with their experience. 100% responded that they felt comfortable talking to the OFFSN on the mobile robot and would allow their infant or themselves to be cared for by a physician via telemedicine in the future."*	**No**	**4**

Confidential: For Review Only

			only	routine hours the OFFSN was called to discuss any issues with the patient. Emergencies/out of hours were covered by an ONSN (on site neonatologist).<br><br>Control: Infants of control parents received ONSN care. The attending neonatologist made daily patient rounds with the NICU team. After patient rounds, the NICU staff, under the supervision of the attending neonatologist implemented the care plan.						
5. Globus et al. (2016), Israel	Mothers and fathers /Total surveys returned: 178	~40% in each group <32 / level III	Non-randomised, Convenience sampling.<br><br>*Unit level effect:*<br>Two different time periods	Intervention: **SMSi-Short Message Services Implementation.**<br><br>Parents were updated daily regarding the health status of their infant via SMS (short-message-services) from the Electronic Patient Record. All SMS messages were sent at 09:00am, including one-sentence sections with updated information (e.g. location of the infant's crib and current weight). Information regarding acute events/deterioration of the infant's medical condition was not included in the SMS, but was delivered personally to the parents in real time.<br><br>Control: Routine care pre-SMS implementation.	***1. Parent satisfaction related to parent communication with the medical staff***<br><br>***2. Overall parent satisfaction with treatment and staff attitudes throughout hospitalisation.***	During babies' admission (once)<br><br>- pre-SMS cohort and post-SMS cohort<br><br>No pre-intervention parent satisfaction data available for comparison (different parent groups pre and post intervention).	*Satisfaction questionnaire*<br><br>The *"Parents' attitudes regarding their experience during their infants' hospitalisation in the NICU"* questionnaire was used, as well as selected items from a literature review of similar questionnaires, including that by York Hospital and by Conner and Nelson.<br><br>Validation: No content validity or reliability testing reported.<br><br>Selected items related to four aspects of the NICU experience. 2 out of 4 directly assessed parent satisfaction:<br><br>1. Parental assessment of their communication with the medical staff.<br><br>Likert scale (1 do not agree at all-5 strongly agree)<br><br>2. Overall satisfaction with treatment and staff attitudes throughout hospitalisation.<br><br>Visual analog scale (scores range 0-10). Higher scores reflect greater satisfaction.	Overall, in both periods, parents expressed a high degree of satisfaction regarding the medical treatment, the information given and the communication with the medical staff. Overall satisfaction with treatment and with staff attitudes throughout hospitalisation was slightly greater in the post-SMS cohort but did not reach statistical significance.<br><br>**In the post-SMS cohort, a statistically significant improvement was noted regarding physician availability and patience, parental feelings of comfort in approaching the physicians and nurses, and regularly receiving information regarding the infants' medical status from the physicians.**<br><br>**Post SMS Pre SMS**<br>**Mean (SD)** 4.1 (1.0) 3.7 (1.3)<br>**p-value** 0.03<br>*Specific question: "I was pleased with the frequency with which I received information regarding my infant".*	**No**	**1**

Confidential: For Review Only

								Although improvement in all other categories was documented, it did not reach statistical significance.		
6.Kazemian et al. (2016), Iran	Mothers /220 newborns (assumed 220 mothers)	>37 / level not stated	Non-randomised, Convenience sampling.<br><br>*Group level effect:* Intervention/ control groups<br><br>Post-intervention testing only	Intervention: **Rooming-in care.**<br><br>Mothers and babies were admitted to a different atmosphere to the routine care. This facilitated the mothers and neonates with separate beds along with phototherapy devices and nursing clinical supervision.<br><br>Control: The routine care practiced in this neonatal unit supported partial stay of mothers beside their neonates, while sitting on chairs; however, most of the time the mother-infant dyad was separated.	***Maternal satisfaction with the neonatal care services and hospital stay comfort***	During babies' admission (once)<br><br>-Not stated exactly when<br><br>No pre-intervention parent satisfaction data available for comparison.	*Satisfaction questionnaire*<br><br>Validation: No content validity or reliability testing reported.<br><br>The authors state, *"a validated self-made questionnaire was employed, which was filled in by some trained midwives."* No further information on validation processes, number of questions or name of the questionnaire was provided.<br><br>Likert (5 very satisfied-1 dissatisfied).	**The level of satisfaction was significantly higher in the intervention group, compared to that in the control group.**<br><br>**Interv** **Control**<br>**Satisfaction %** 26.6 18.8<br><br>**p-value** 0.027	**No**	**1**
7. Petteys et al. (2015), USA	Not stated/ 10 parents included in sample analysis	24-36+ / level III	A prospective cohort design.<br><br>A feasibility study.<br><br>*Group level effect:* Intervention/ control groups<br><br>Post-intervention testing only	Intervention: **PC (Palliative care).**<br><br>PC nurses provided important continuity of care for NICU infants clinically requiring PC and at least weekly verbal support of parents. The PC service also coordinated family conferences, provided or requested orders to improve infant symptom management and comfort, and addressed parental coping and self-care.<br><br>Control: Usual clinical care for infants not requiring PC.	***Overall satisfaction with care received***	During babies' admission (once)<br><br>- At discharge (or study closure for infants who remained hospitalised)<br><br>No pre-intervention parent satisfaction data available for comparison.	*Satisfaction questionnaire*<br><br>A researcher-created questionnaire based on extensive current literature review.<br><br>Validation: Partially reported. Authors stated content validity testing took place; no information on reliability testing provided.<br><br>1 question<br><br>Likert (1 extremely dissatisfied-4 to extremely satisfied).<br><br>Optional free text (description of specific experiences impacting satisfaction with care)	**Parent satisfaction response numbers were small (n= 10), thus statistical comparison of parental satisfaction between cohorts was not possible.**<br><br>However, 100% of responding PC parents (n= 2) reported being "extremely satisfied" with care, whereas only 50% of responding usual care parents (n= 4) reported extreme satisfaction.	**No**	**3**

Confidential: For Review Only

8. Van de Vijver and Evans (2015), UK	Not stated /105	Not stated / not stated	Non-randomised, Convenience sampling.<br><br>*Unit level effect:*<br>Three different time periods	Intervention: **Baby diary.**<br><br>Each parent received a communication diary on their infant's admission to the unit. Staff wrote-in infant status updates and kept an infant interaction log with parents. Parents wrote in memories and questions for staff to address during face-to-face communication.<br><br>Control: Routine care, before implementation of the diaries.	***Satisfaction with communication from neonatal staff***	During babies' admission (three times)<br><br>- On the day of babies' discharge at study baseline<br><br>- On the day of babies' discharge at 1 month<br><br>On the day of babies' discharge at 15 months	*Satisfaction questionnaire*<br><br>The study team designed a questionnaire, based on the Department of Health and the National Institute for Health and Care Excellence (NICE) quality standards for specialist neonatal care.<br><br>Validation: No content validity or reliability testing reported.<br><br>5 questions ("yes or no")	**Small numbers. No data indicating statistical analysis conducted or evidence of statistically significant results.**<br><br>*"I was receiving regular communication from staff"*<br>94% - 1 month post diary cohort<br>93% - 15 months post diary cohort<br>77% - pre diary cohort<br><br>*"My questions and concerns were being addressed"*<br>100% - 1 month post diary cohort<br>93% - 15 months post diary cohort<br>91% - pre diary cohort<br><br>"I feel more involved in my baby's care"<br>92% - 1 month post diary cohort<br>100% - 15 months post diary cohort<br>88% - pre diary cohort	**Yes.**<br><br>The interventi on concept was created by the project leaders following analysis of baseline survey results and used after multi-disciplina ry input and discussion with staff and parents.	**3**
9. Voos and Park. (2014), USA	Not stated / 62	Not stated / level III	Non-randomised, Convenience sampling.<br><br>*Unit level effect:* Two different time periods	Intervention: **OU (Open Unit) policy.**<br><br>Parents were allowed access to their baby 24 hours a day, 7 days a week.<br><br>Control: Parents pre-OU implementation received routine care. The unit was closed to parents during nurse change of shift in mornings and evenings.	***Parent satisfaction with how much time parents get to spend with their baby***	After babies were discharged (once)<br><br>- After pre-OU parents were discharged<br><br>- After post-OU parents were discharged	*Single question (From a validated questionnaire)*<br><br>The question *"Did you get to spend as much time as you wanted with your baby?"* was used from the NRC (National Research Corporation) Picker parent survey.<br><br>1 question ("yes or no")	**Small numbers. No data indicating statistical analysis conducted or evidence of statistically significant results.**<br><br>*"Did you get to spend as much time as you wanted with your baby?" Yes.*<br><br>Pre OU 78% (18/23)<br>Post OU 92% (36/39)	**Yes.**<br><br>The NICU has a Family-centered care committe e including parents, which conducted this project.	**3**
10. Segre et al. (2013), USA	Mothers /23	Mean (SD) 31.57 (5.30) / level III	For the outcome of parent satisfaction:<br><br>Non-Randomised, Convenience sampling.<br><br>*Group level effect:*<br>Intervention/	Intervention: **(LV) Listening visits.**<br><br>Mothers met with the LV provider for up to six 50-min LV sessions, conducted in a private hospital, every 2–3 days, within 1-month. Visits entailed greeting, debriefing, updating on current issues, working an agenda through listening and problem solving, and providing closure through	***Satisfaction with the treatment and the outcome.***	During babies' admission (once)<br><br>- Not stated exactly when<br><br>No pre-intervention parent satisfaction data available for comparison.	*Satisfaction questionnaire*<br><br>The *Client Satisfaction Questionnaire* was used.<br><br>Validation: Partially reported. Authors stated reliability testing took place; no information on content validity provided.<br><br>8 questions.<br><br>Format of questions: not stated	**Only the intervention group was assessed and only post-intervention.**<br><br>The authors reported:<br><br>*"The majority of women who received LVs were highly satisfied with the intervention".*<br><br>*"The average score for the Client Satisfaction Questionnaire was 29.91, comparable to levels of satisfaction reported by clients receiving depression treatment from a mental health professional."*	**No**	**4**

Confidential: For Review Only

			control groups<br><br>Post-intervention group testing only	summary.<br><br>Control: Women who did not meet the specific criteria (e.g. minimum score on depression scale) were not invited to join the treatment trial and received routine NICU care/support instead.				*"91.3% of our participants rated the quality of help they received as excellent."*		
11. Palma et al. (2012), USA	Not stated / 26 families returned the survey containing the satisf. measure)	Not stated / level II	Non-randomised, Convenience sampling.<br><br>*Unit level effect:*<br>Two different time periods	Intervention: **YBDU (Your Baby's Daily Update). A daily parent update letter generated from the Electronic Medical Record (EMR).**<br><br>Parents were given daily YBDU reports, printed automatically from the EMR. The YBDU included information about an infant's status during the past 24 hours and a hand-written update by the infant's care provider.<br><br>Control: Parents received routine care and usual verbal updates (6 months pre- adoption of YBDU).	***Satisfaction with YBDU***	During babies' admission (once)<br><br>- Not stated exactly when<br><br>No pre-intervention parent satisfaction data available for comparison (different parent groups pre and post intervention).	*Satisfaction questionnaire*<br><br>A questionnaire including items regarding adoption of and satisfaction with YBDU was used.<br><br>Validation: No content validity or reliability testing reported.<br><br>Number and format of questions: not stated.	**Only the intervention group was assessed and only post-intervention.**<br><br>The authors reported:<br>*"When asked to rate the statement "I like receiving Your Baby's Daily Update", 96% of families who used YBDU as an information source responded with the highest rating, "always"."*	**No**	**4**
12. Stevens et al. (2011), USA	Mothers /147. For the OPBY NICU, 58 surveys were returned. For the SFR NICU, 89 were returned	Mean (SD)<br><br>Control: 35 (4)<br><br>Interv: 34 (3)<br><br>/ level not stated	Cohort trial. This research was part of a large prospective evaluation.<br><br>*Unit level effect:*<br>Two different time periods	Intervention: **SFR (Single-family room) NICU for neonatal care.**<br><br>Parents could visit their baby, room-in, do kangaroo care and breastfeed at any time, in individual rooms (containing bed, desk, closet, telephone, chair, refrigerator for breast-milk storage).<br><br>Control: OPBY (Open-bay) NICU. The traditional open-bay NICU was typical of facilities built before 1980. All neonates, family members, staff, monitors, and equipment were visible for all neonates in each room. Portable partitions were placed	***Parent satisfaction with different elements of NICU:***<br>*- Delivery*<br>*- Environment*<br>*- Nurses*<br>*- Physicians*<br>*- Discharge*<br>*- Personal*<br>*- Overall Assessment*	After babies were discharged (once)<br><br>- Mailed within 60 days of discharge of parents' infants from the NICU<br><br>No pre-intervention parent satisfaction data available for comparison (different parent groups pre and post intervention).	*Satisfaction questionnaire*<br><br>A questionnaire from Press Ganey Associates was used. Also included were three questions added by the investigators.<br><br>Validation: Partially reported. The original questionnaire was validated questionnaire but no content validity or reliability testing was reported regarding the 3 questions added by the study team.<br><br>42 questions in total (7 categories):<br>Delivery, Environment, Nurses, Physicians, Discharge, Personal, Overall Assessment.<br><br>Likert (1 very poor-5 very	**Statistically significant improvement was found for the survey categories of Environment, Overall and the Total survey.**<br><br>Estimated numbers from report's figures as numbers not provided):<br><br>**Median SFR OPBY p-value**<br>Environment 4.7 3.7 <0.001<br>Overall 5 4.8 0.018<br>Total 4.7 4.5 0.045<br><br>16 items composite score for family-centered care:<br>4.4 4.0 0.017	**Yes**<br><br>Former NICU parents were involved in all phases of planning for the new SFR NICU.	**1**

Confidential: For Review Only

				around the incubator for breastfeeding and kangaroo care.			good).			
13. Voos et al. (2011), USA	Not stated /28	Not stated / level not stated	Non-randomised, Convenience sampling.<br><br>*Unit level effect:*<br>Two different time periods	Intervention: **Family-centered rounds (FCRs).**<br><br>Parents were invited to attend rounds and choose their level of involvement (attend every day/not at all/periodically). For confidentiality concerns, parents were asked to step outside while rounds of others' infants took place. The staff augmented FCRs by meeting with parents again after rounds if needed.<br><br>Control: Parents received routine care. Prior to FCR implementation parents were asked to leave the unit during rounds.	***Global satisfaction with the NICU experience***	During babies' admission (twice)<br><br>- Prior to FCR<br><br>- 6 months after starting FCR	*Satisfaction questionnaire (Validated)*<br><br>*The NIPS* questionnaire.<br><br>24 questions: looking at satisfaction in different areas of the NICU (medical caregivers, communication, tests, and procedures).<br><br>Likert scale (1-7 points).	**A subset of NIPS items related to communication (i.e. being kept informed as to changes in the infant's condition, meeting with physicians, and information about long-term expectations) yielded a significant increase from pre to post FCR scores.**<br><br>**post-FCR pre-FCR p-value**<br>**NIPS score** 5.5 4.4 <0.01<br><br>The average score on the NIPS did not change significantly.	**No**	**1**
14. Weiss et al. (2010), USA	Mothers /84	Mean (SD)<br><br>Pre-interv group: 32 (4.4)<br><br>Post-interv group: 32 (9)<br><br>/ level III	Non-randomised, Convenience sampling<br><br>*Unit level effect:*<br>Two different time periods	Intervention:<br>**An intervention to increase PMP (Principal Medical Providers) availability and communication frequency.**<br><br>(1) A brief education module for PMPs was introduced (2) parents received a contact card with PMP names, job descriptions and contact information (3) a poster of the faces, names and titles of the PMPs was placed at NICU entrance.<br><br>Control: Parents received routine care in the pre-intervention cohort, without the above.	***Parent satisfaction with physician and nurse practitioner communication***	During babies' admission (twice)<br><br>- Pre-intervention<br><br>- Post-intervention	*Satisfaction Questionnaire (Validated)*<br><br>A pilot survey written by Press Ganey and the Picker Institute was used and revised based on parent responses.<br><br>6 open-ended questions (Quantity of communication)<br><br>6 Likert scale questions (range questions (Availability, understanding, reciprocity, empathy, overall satisfaction)	**Overall satisfaction, based on the ordinal analysis of the five-point Likert scale, was significantly higher after the intervention (P<0.01).**<br><br>**Overall satisfaction, dichotomised into a satisfied subgroup and a dissatisfied subgroup for each cohort, was also significantly increased after the intervention.**<br><br>**post –interv pre-interv**<br>**Very satisfied/ Somewhat satisfied** 97%(32/33) 74%(37/50)<br><br>**p-value** <0.01	**No**<br><br>Authors stated that only after trialing the intervention on many parents (both satisfied and unsatisfied) gave suggestions to improve it.	**1**
15. Foster et al. (2008), Australia	Mothers and fathers /93<br><br>5 Special Care	Mean (SD)<br><br>Headbox: 36.5 (2.6)	Non-randomised, Convenience sampling<br><br>*Group level*	Intervention 1: **Infants received headbox oxygen treatment for respiratory distress.**<br><br>Intervention 2: **Infants**	***Satisfaction with treatment (i.e. headbox oxygen or CPAP)***	During babies' admission (once)<br><br>- Within 5 days of the babies' admission.	*Single question*<br><br>Validation: No content validity or reliability testing reported.	**Parents with babies receiving CPAP rated their satisfaction with the baby's treatment statistically significantly higher than the headbox**	**No**	**1**

Confidential: For Review Only

	Nurseries	CPAP: 36 (3)<br><br>/level I	*effect:* Intervention 1/ intervention 2 groups<br><br>Post intervention testing only	**received continuous oxygen positive airway pressure (CPAP) treatment for respiratory distress.**		No pre-intervention parent satisfaction data available for comparison.	1 likert scale question (1 not at all satisfied-5 extremely satisfied).	**group mean rating.**<br>**Headbox** **CPAP**<br>**Mean (SD)** 3.71 (1.31) 4.51 (0.79)<br>**p-value** 0.001<br><br>The CPAP group averaged between *very and extremely satisfied* compared with parents of babies receiving headbox, who averaged between *satisfied and very satisfied ratings.*		
16. Byers et al. (2006), USA	Only mothers reported /35	Preterm infants<br><br>Mean (SD)<br><br>Control: 28.9 (3.44)<br><br>Interv: 28.6 (3.37)<br><br>/ level II/III	For the outcome of parent satisfaction:<br><br>Non-randomised, Convenience sampling<br><br>*Group level effect:* Intervention/ control groups<br><br>Post-intervention testing only	<u>Intervention:</u> **Infants received individualised, developmentally supportive family-centered care.**<br><br>Infants received care within the framework and philosophy of individualised, developmentally supportive family-centered interventions.<br><br><u>Control:</u> Infants received the traditional NICU standard of care.	***Parent satisfaction relating to:***<br>*- parental perceptions of staff caring*<br>*- education received*<br>*- preparation for the parental role*<br>*- overall satisfaction with the NICU experience*	During babies' admission (once)<br><br>- On the day before discharge<br><br>No pre-intervention parent satisfaction data available for comparison.	*Satisfaction questionnaire*<br><br>The *NICU's parental satisfaction tool* was used.<br><br><u>Validation:</u> Partially reported. Authors stated content validity testing took place, but "because of the disparate nature of the items, survey reliability was not assessed".<br><br>11 questions<br><br>Likert scale (1-5 strongly agree)	**Independent t-test analysis of parent satisfaction/perception scores showed no significant difference between groups.**<br><br>Example statement: "*I was satisfied with the car my baby and I received in the NICU"*<br><br>**Interv** **Control**<br>**Mean (SD)** 4.94(0.23) 4.71(0.47)<br>**p-value** 0.064<br><br>Both groups reported very high satisfaction with their NICU experience (4.4-5.0)	**No**	**2**
17. Mills et al. (2006), USA	Not stated/ not stated<br><br>Parents of infants from 6 hospitals	Not stated / level not stated	Implementation project<br><br>Plan Do Study Act (PDSA) quality improvement testing	<u>Intervention:</u> **5 potentially better practices (PBPs) in the area of discharge planning.**<br><br>The project team iteratively implemented 5 PBPs:<br>1. Created an easy-to-use, easy-to-access discharge planning tool kit.<br>2. Restructured communication tools and processes to reflect a "plan for the day, the stay, and the way" to discharge.<br>3. Maximised the impact and use of caregiver educational tools, and updated materials and delivery systems for caregiver education.<br>4. Used various continuous quality improvement tools and processes to ensure	***General satisfaction***<br>*- with care*<br>*- parents' feelings about preparedness for discharge*<br>*- ability and confidence in feeding*<br>*- familiarity with their infant*<br>*- feeling like a parent*<br>*- participation in care*<br>*- adequacy of information from staff about medical and care issues*	During babies' admission (4 times)<br><br>- Not reported exactly when	*Satisfaction questionnaire*<br><br>The Internet-based parent satisfaction survey *"howsyourbaby.com"* that was developed especially for this NICU population was used.<br><br><u>Validation:</u> No content validity or reliability testing reported.<br><br><u>Number and format of questions:</u> not stated.	Through multiple rapid-cycle projects, the project's collaborative group made changes within the 5 PBP plans.<br><br>**Parent satisfaction measures were used to longitudinally monitor the changes made, rather than make direct group comparison. No data indicating statistical analysis conducted or evidence of statistically significant results.**<br><br>Parent satisfaction survey results (all centers combined) were high across 4 measurement quartiles. No specific interquartile analysis was reported.<br><br>Parent readiness for discharge was high at the beginning and throughout the collaborative. Parents' receiving *"just the right amount of information"* regarding car seat trials and	**No**	**3**

Confidential: For Review Only

				parent/caregiver and staff satisfaction.<br>5. Analysed and enhanced interactions with and transfers into the community.<br><br><u>Control:</u> N/A. No discrete control group. PDSA quality improvement methodology was applied to parent participants.				safe sleep demonstrated some variability throughout the collaborative.		
18. Wielenga et al. (2006), The Netherlands	Mothers and fathers / 46	Mean (SD)<br><br>Control: 28.5 (26.0–29.9)<br><br>Interv: 28.3 (25.6-29.9)<br><br>/ level III	Non-randomised, Convenience sampling<br><br>*Unit level effect:*<br>Two different time periods	<u>Intervention:</u> **The Newborn Individualised Developmental Care and Assessment Program (NIDCAP).**<br><br>Infants received care according to NIDCAP principles and parents were taught how to provide it. Caregiving plans were designed based on the infant's current developmental stage, medical condition and family needs. Caregivers learnt to watch sensitively and note the infant's reactions to different types of handling and care, making continuous adjustments.<br><br><u>Control:</u> Infants received traditional neonatal care practiced at that time.	***Parent satisfaction relating to:***<br>*-Overall rating*<br>*-Care of the baby*<br>*-Communication with staff*<br>*-Involvement in care -Being prepared -Support*<br>*-Being a parent*<br>*-Being near your baby -Total score*	After babies were discharged (on day of discharge/ transfer)<br><br>- Pre NIDCAP cohort<br><br>- Post NIDCAP cohort	*Satisfaction questionnaire (Validated)*<br><br>The *NICU-PSF* was used and translated from English to Dutch.<br><br>62 questions<br><br>Closed and open-ended questions.<br><br>Different rating scales used (5-point rating scale from "extremely satisfied" to "not at all satisfied" or "excellent" to "poor").<br><br>Total score range (50-243 points)	**The intervention group's mean total score was significantly higher than the control.**<br><br>**Interv Control**<br>**Mean (SD)**<br>185.67(17.74) 174.04(20.98)<br><br>**p-value** 0.041<br><br>Almost all separate concepts showed an increase in their mean scores. The concept of "*being a parent*" had a slightly lower mean score (9.39, SD = 1.73) in the intervention group than in the control group (9.78, SD = 2.09).<br><br>**The concept of *"preparedness"* showed statistically significant difference:**<br><br>**Interv Control**<br>**Mean** 16.38 13.83<br>**p-value** 0.038	**No**	**1**
19. Penticuff and Arheart. (2005), USA	Dyads (both parents or mother with her support person)/ 122 mothers<br><br>Results based only on mothers' data.	Not stated /<br>Level III	A repeated measures design<br><br>- First 2 years (control group data collection)<br><br>- Year 3 (staff training)<br><br>- Year 4 (implementing the intervention)<br><br>- Year 5 (collecting data	<u>Intervention:</u> **The Newborn Individualised IPC- CPM intervention (Infant Progress Chart) - (Care Planning Meetings).**<br><br>Both the mother and father (or the mother and her designated support person) were shown how to use the Infant Progress Chart and attended 3 Care Planning Meetings (with neonatologists/Neonatal Nurse Practitioners).	***Satisfaction with participation in decision making* was measured by 5 collaboration indices:**<br><br>Satisfaction with<br>(1) Care<br>(2) Relationships with professionals<br>(3) Decision input<br>(4) The process of decision	During babies' admission (three times)<br><br>- Within 0–3 days<br><br>- 9– 12 days<br><br>- 25–28 days of an infant's admission to the NICU	*Three satisfaction questionnaires*<br><br>1. Two subscales of the investigator-designed *"Parents' Understanding of Infant Care and Outcomes Questionnaire"* were used to measure Satisfaction with Care (1).<br><br><u>Validation:</u> Partially reported. Authors stated content validity testing took place; no information on reliability testing provided.	**The intervention group was more satisfied with the amount of decision input they had (3) and with the process by which medical decisions were made (4).**<br><br>**Interv Control p-value**<br>Decision input amount (3)<br>**Mean** 33.44 30.05 0.058<br><br>Process of decision making (4)<br>**Mean** 120.20 104.95 0.012<br><br>There were no statistically significant differences between control and intervention groups in satisfaction with their infants' care (1), with relationships with	**No**	1

Confidential: For Review Only

			from the intervention group)<br><br>*Unit level effect:* Two different time periods	Control: During the control phase, professionals carried out usual communication and interaction with control group parents.	making<br>(5) Decisions made		30 questions.<br><br>Five-point Likert scale.<br><br>2. A subscale of the investigator-designed *"Relationships with Professional and Decision Input Questionnaire"* was used to measure Satisfaction with relationships (2).<br><br>Validation: Partially reported. Authors stated content validity testing took place; no information on reliability testing provided.<br>12 questions.<br><br>Five-point Likert scale<br><br>3. Validated.<br>The *"Collaboration and Satisfaction About Care Questionnaire"* developed by Baggs, was used to measure Satisfaction with decision input (3), with decision process (4) and with decisions made (5).<br><br>9 questions.<br><br>7-point scale, (1 strongly disagree -7 strongly agree)	NICU professionals (2) and with the decisions made for infant treatment (5).		
20. Byers et al. (2003), USA	Mothers/ 19	Mean (SD)<br><br>Control: 29 (2.00)<br><br>Interv: 28.9 (2.42)<br><br>/ level II-III	For the outcome of parent satisfaction:<br><br>Non-randomised, Convenience sampling<br><br>*Group level effect:* Intervention/ control groups<br><br>Pre and post-intervention testing	Intervention: **Co-bedding premature multiple-gestation infants in incubators.**<br><br>Infants were nursed in the same incubator using a co-bedding protocol (e.g. recording all of the care provided to one infant before providing care to the second infant)<br><br>Control: Single-bedding premature multiple-gestation infants in incubators.	***Parent satisfaction related to:***<br>*- staff concern*<br>*- support of family*<br>*- staff explanations*<br>*- infant environment,*<br>*- comfort with feeding*<br>*- kangaroo care encouragement*<br>*- staff explanation of signs of infant stress*<br>*- visiting schedule*<br>*- overall satisfaction with the NICU*	During babies' admission (twice)<br><br>- At baseline<br><br>- 5 days later	*Satisfaction questionnaire*<br><br>The NICU's standard parental satisfaction tool was used.<br><br>Validation: Partially reported. Authors stated content validity testing took place, but because of the disparate nature of the items, survey reliability could not be assessed.<br><br>11 questions.<br><br>5-point Likert-type scale.	**The only significant difference for a post-intervention item was a higher score for the item "Attempts were made to create a quiet environment for my baby."**<br><br>**Interv Control p-value**<br>**Mean** 4.80 3.89 0.033<br><br>Independent t-tests comparing the co-bedded and control group parental scores found no significant differences in their parental satisfaction scores, except for higher baseline parental satisfaction scores (p=0.029) in the co-bedded group.	**No**	**1**

Confidential: For Review Only

					experience					
21. Polizzi et al. (2003), USA	Mothers and fathers/ 33	Mean (SD)<br><br>Control: 32.97 (1.9)<br><br>Interv: 33.08 (1.31)<br><br>/ level III	A retrospective, comparative, descriptive design.<br><br>*Unit level effect*	Intervention: **Co-bedding multiple-gestation infants in the NICU.**<br><br>Multiple-gestation infants were nursed in the same incubator or crib. The intervention was evaluated retrospectively after implementation of a co-bedding practice protocol.<br><br>Control: Traditionally-bedded group (babies were routinely placed in separate incubators or cribs)	***Parental satisfaction as measured by 9 questions relating to parent perceptions and their baby's care***	After babies were discharged (once)<br><br>- All parents were mailed the survey. A second survey was sent to those who did not respond after 2 months<br><br>No pre-intervention parent satisfaction data available for comparison.	*Satisfaction questionnaire*<br><br>The *parental perception/ satisfaction tool* was used.<br><br>Validation: Partially reported. Authors stated content validity testing took place; no information on reliability testing provided. 6/9 questions were from a similar tool that was validated by the Vermont Oxford NICU Quality Improvement Initiative.<br><br>9 questions (such as *"I was satisfied with the care my babies received in the hospital").*<br><br>Likert (1 strongly disagree- 5 strongly agree)	Mothers reported overall satisfaction with the NICU care and staff, as well as adequacy of their ability to care for their infants after discharge, with scores ranging from 4.19 to 4.71.<br><br>**The only survey item score that was significantly different between groups was for the item *"I was encouraged by the hospital staff to bond with my babies."***<br><br>**Interv Control p-value**<br>**Mean** 4.71 4.36 0.049	**No**	**1**
22. Legault and Goulet. (1995), Canada	Mothers/ 61 completed both tests	Mean (range)<br><br>30 (24-35)<br><br>/ level II	Time-series design<br><br>*Group level effect:* Same group exposed to both methods with post-method testing only.	Intervention: **Kangaroo method of removing an infant from an incubator.**<br><br>Mothers were taught the "kangaroo method" (skin-to-skin contact): infant wears a diaper/head cap and is placed in a vertical position on the parent's bared chest. A blanket covers the infant and the parent's clothing is fastened around the infant. The parent sits in a rocking chair, inclined so that the infant's head is at 60'.<br><br>Control: Traditional method. Newborns wearing a diaper and a head cap, are wrapped in a blanket and placed in their parent's arms.	***Mothers' satisfaction with:***<br>*- Each method of removing an infant from incubator*<br>*- Her feelings after each method*	During babies' admission (twice)<br><br>- After the intervention<br><br>- After the control method<br><br>No pre-intervention parent satisfaction data available for comparison.	*Satisfaction questionnaire*<br><br>The *"Maternal Satisfaction Questionnaire"* was used. It was developed by integrating components described by Affonso et al and the clinical experience of the investigators.<br><br>Validation: Partially reported. Authors stated content validity testing took place; no information on reliability testing provided.<br><br>15 questions<br><br>Likert (1 very much-5 don't know)<br><br>An open-ended question invited the mother to list and explain anything else related to her experience.	**Regardless of the method tested, mothers expressed high levels of satisfaction (it was the first time since giving birth that they could hold their infants).**<br><br>Three statements proved more powerful in discriminating between the methods:<br><br>**Rated higher after the kangaroo method test:**<br>- *"I like the contact with my baby's skin"*<br>(p=0.0001)<br><br>**Rated higher after the traditional method test:**<br>- *"I like to talk to and whisper to my baby" (p = 0.015)*<br>- *"I looked into my baby's eyes and stared at his/her face" (p=0.0001)*	**No**	**1**

Confidential: For Review Only

Randomised controlled trials (RCT) by publication year

Author (Date), Country	Parent Gender/ sample size	Infants Gestation age (GA) in weeks /NICU level	Study design	Intervention	Outcome measures	Timing of measurement	Method of measurement	Results	Parent co-design?	Improved parent satisfaction?
1. Northrup et al. (2016), USA	Mothers and fathers /116	<28 / level III	Randomised controlled trial	Intervention: **Free Parking (FP).**<br><br>Parents received 7 parking vouchers at a time (value: $10/each) and continued to receive vouchers until infant discharge. Each voucher allowed free entry and exit for 24hr.<br><br>Control: Parents received the standard care and did not receive vouchers.	***Parent satisfaction with NICU care***	After babies were discharged (once)<br><br>- During the first high-risk-infant clinic visit after discharge<br><br>No pre-intervention parent satisfaction data available for comparison.	*Satisfaction questionnaire*<br><br>Validation: No content validity or reliability testing reported.<br><br>11 questions<br><br>- Seven items were summed (score 7-35) to measure "Support" (e.g., information sharing).<br><br>- Three items measured "Emotional Connection" to the infant (score 3-15)<br><br>- One item assessed "family involvement in infant care" (responses: not enough-just right-too much).<br><br>Greater scores indicated higher perceived support, connection and satisfaction.	**The groups did not differ significantly with respect to satisfaction.**<br><br>**Interv Control p-value**<br>NICU support<br>**Mean (SD)** 30(2.7) 28.7(3.7) 0.07<br><br>Emotional connection<br>12.3(1.7) 12.3(1.7) 0.96<br><br>Family involvement "Just right"<br>81.4% 85% 0.07	**No**	**2**
2. Abdel-Latif et al. (2015), Australia	Mothers and fathers /63	25-42 / level III	Cross-over Randomised Controlled Trial	Intervention: **Parental Presence at Clinical Bedside Rounds (PPCBR).**<br><br>Parents attended bedside clinical rounds. Parents had opportunity to ask questions about their baby's condition and management.<br><br>Control: Parents received the standard care with no parental presence at bedside clinical rounds.	***Parent satisfaction assessed by questions of 3 domains:***<br>*1. Knowledge and understanding*<br>*2. Communication and collaboration*<br>*3. Privacy and confidentiality*	During babies' admission (once)<br><br>- At the end of each study arm, separated by a washout period<br><br>- No pre-intervention parent satisfaction data available for comparison	*Satisfaction questionnaire*<br><br>The authors stated "the research team designed the questionnaire".<br><br>Validation: No content validity or reliability testing reported.<br><br>Number and format of questions: not stated	**PPCBR had significantly higher adjusted mean (95% CI) scores for some questions from domains 1 and 2.**<br><br>Domain 3 was comparable between the two study groups.<br><br>**Interv Control p-value**<br>Domain 1 question:<br>*"I have received adequate information about my baby's condition and management"*<br>**Mean** 4.321 3.947 0.03<br><br>Domain 2 questions:<br>*"In the last week I have been able to communicate effectively with my baby's healthcare team"*<br>**Mean** 4.407 4.250 0.05<br><br>*"In the last week I have collaborated with my baby's healthcare team in the*	**No**	**1**

Confidential: For Review Only

								planning of care for my baby"<br>**Mean** 3.843 3.426 0.02<br><br>*"In the last week I have been able to ask the healthcare team questions about my baby's care"*<br>**Mean** 4.642 4.259 0.004		
3. Bastani et al, (2015), Iran	Mothers /100	30-37<br>Mean (SD)<br><br>Control: 33.90 (2.33)<br><br>Interv: 34 (1.9)<br><br>/ level not stated	Randomised Controlled Trial (block randomisation)	Intervention: **Family-centered Care (FCC).**<br><br>Mothers allowed access to their baby at any time, participated in the care process and were provided with information about neonatal care.<br><br>Control: Mothers received the standard care where they were only allowed to be present at the time of the infant's entry to the neonatal care unit, and were only routinely informed.	***Maternal satisfaction*** **relating to three themes:**<br>1. Parental presence<br>2. Participation in neonatal care<br>3. Information about neonatal care	During babies' admission (twice)<br><br>- 24 hours after admission<br>- At the time of discharge	*Satisfaction questionnaire* (Validated)<br><br>A modified satisfaction questionnaire was used, based on a parental satisfaction instrument developed for measuring satisfaction in Paediatric intensive care Units (PICU).<br><br>18 questions<br><br>Graded 0 (very dissatisfied) to 4 (very satisfied).<br><br>The overall satisfaction rate was classified based on the mean scores (score<50%, between 75-50% and > 75%).	**In the FCC group, pre and post intervention difference in maternal satisfaction was statistically significant p<0.001**<br><br>**Interv Control p-value**<br>**Mean (SD)**<br><br>**At 24 hr**<br>22.36(8.90) 22.06(9.77) 0.87<br><br>**At discharge**<br>59.28(6.86) 30.18(14.09) <0.01	**Unclear**<br><br>Mothers determined the reliability of the satisfaction tool and approved the educational pamphlet. Authors did not report if mothers had direct input in the intervention design.	**1**
4. Clarke-Pounder et al. (2015), USA	Mothers and fathers /19 families	23-39 / level III	Randomised Controlled Trial	Intervention: **Sharing information obtained from parent interviews with the primary NICU provider.**<br><br>Parents interviewed using the *NICU- adapted Decision Making Tool (N-DMT).* Information obtained was placed in the electronic medical record (EMR) and shared with the primary neonatal provider via email. Daily rounds on all infants were audio-recorded for 3 days after enrollment to see if information from the N-DMT was incorporated into daily care planning.<br><br>Control: The content of a recent social work note was communicated with the primary provider via e-mail, creating an attentional control group.	***Parent satisfaction with care***	During babies' admission (once)<br><br>- 2 weeks after study entry<br><br>No pre-intervention parent satisfaction data available for comparison.	*Satisfaction questionnaire*<br><br>A *NICU- adapted Decision Making Tool (N-DMT)* – specific questionnaire was used.<br><br>Validation: Partially reported. Authors stated reliability testing took place; no information on content validity provided.<br><br>8 questions: e.g."My baby's doctors considered my goals and hopes for my baby during decision-making".<br><br>Likert scale (1 strongly agree-4 strongly disagree). Total N-DMT score range 8–32.	**There was no significant difference in satisfaction with care as measured by the N-DMT scale between the control group and intervention groups in a univariable model or multiple variable model controlling for gestational age.**<br><br>**Interv Control**<br>**Median** (range)<br>26(15–28) 28.8(19–32)<br><br>**No p-value reported**<br><br>There was, however, a pattern of decreased satisfaction with care among the intervention group compared to the control group across the N-DMT-specific survey questions, although the differences were not statistically significant.	**Yes**<br><br>Information obtained from parents using the N-DMT was placed in the electronic medical record (EMR) and shared with the primary NICU provider via email (forming the intervention)	**2**
5.Holditch-Davis et al. (2013), USA	Mothers /208	Preterm infants	Randomised controlled trial	Interventions: **1. Mothers were taught how to massage infants with auditory, tactile, visual,**	**1.** ***Parent (mother) satisfaction with the***	During admission period and post discharge	*Satisfaction questionnaire*<br><br>The questionnaire was designed by the study team.	**No significant differences occurred between the groups.**	**No**	**2**

Confidential: For Review Only

		Mean (SD)<br><br>Overall group 27.2 (3.0)<br><br>/ 4 centres, levels II-III	3 groups (2 intervention and 1 control)<br><br>Post-intervention testing only.	**and vestibular stimulation (ATVV intervention)**<br>**2. Kangaroo care**<br><br>Control: Attention control group. Mothers spent a similar amount of time with the study nurse discussing the equipment needed for preterm infant care at home. Study nurses provided education and support for all three groups. Mothers were not prevented from engaging in interventions of the other groups but did not receive formal education from the study nurse on the other interventions.	***intervention***<br><br>***2. Satisfaction with the helpfulness of the study nurse***<br><br>***3. Whether the mother would recommend the study to others and the degree of change in the mother as a person and as a mother as a result of being in the study.***	- At the time of discharge<br><br>- At 2 months corrected age<br><br>No pre-intervention parent satisfaction data available for comparison.	Validation: Partially reported. Authors stated reliability testing took place; no information on content validity provided.<br><br>26 questions: relating to three dimensions of satisfaction: efficacy, caring, and technical quality.<br><br>Likert (1 least satisfied-5, 5 most satisfied)	Mothers in all three groups were satisfied with the intervention (mean scores of 3.3 or higher on a 5-point scale) and the helpfulness of the nurse (mean scores of 4.6 or higher on a 5-point scale).		
6. Franck et al. (2011), UK	Mothers and fathers /169	Mean (SD)<br><br>Control: 31.94 (5.17)<br><br>Interv: 29.40 (3.17)<br><br>/4 centres, level III	Cluster Randomised Controlled Trial	Intervention: **Increasing parental involvement in infant pain management in the NICU.**<br><br>Parents received a booklet providing evidence-based information about pain and comforting infants in the NICU setting. Parents received 2 visits from a research nurse showing them how to apply the comforting techniques described in the booklet.<br><br>Control: As part of usual care, parents in both the intervention and control groups received a detailed booklet with generic information about NICU care. Parents in the control group also received 2 visits from a research nurse listening to what parents had to say about their NICU experience (attention placebo).	At baseline:<br><br>***1. Parent satisfaction with NICU care***<br><br>One week after the intervention:<br><br>***1. Satisfaction with information about pain control***<br><br>***2. Satisfied nurses make infant comfortable***<br><br>***3. Satisfied pain medicines help infant***	During babies' admission (twice)<br><br>-At baseline (within 3 to 7 days of admission)<br><br>- 1 week after the intervention	*Individual questions*<br><br>Validation: No content validity or reliability testing reported.<br><br>1. At baseline:<br><br>Parent satisfaction was measured by 1 question: *"Satisfaction with NICU care"* (1 very satisfied-6 very unsatisfied) as part of the baseline parent characteristics questionnaire.<br><br>2. One week after the intervention:<br><br>Three questions using the word "satisfied' were selected from the validated *Parent Attitudes About Infant Nociception (PAIN)* survey (Likert scale 1 very satisfied-6 very unsatisfied)	**At baseline: there was no significant difference in satisfaction between intervention and control group**<br><br>**Interv** **Control**<br>**Mean (SD)** 1.45(0.71) 1.51(0.76)<br><br>**p-value** missing<br><br>**1 week after the intervention: Intervention parents were more satisfied with the information about pain control received than control parents.**<br><br>**Interv** **Control**<br>**Mean (SD)** 2.10(0.97) 3.28(1.27)<br>**p-value** < 0.001	**Yes**<br><br>The booklet was reviewed by 12 parents of infants who had been cared for in NICUs in the United Kingdom.	**1**
7.Livingston et al. (2009), USA	Mothers /12	Mean (SD)<br><br>Control:	Randomised Controlled Trial	Intervention: **Touch and massage.**<br><br>Mothers attended a 1hr massage class taught by a	***1. Caregiver (mother) satisfaction with their infant's care***	During babies' admission (three times)<br><br>- At baseline	*Satisfaction questionnaire*<br><br>Two questionnaires were developed by the research team.	**It is unclear in the report if specific between-group comparisons and statistical analysis were conducted.**	**No**	**3**

Confidential: For Review Only

		33.4 (6.4)<br><br>Interv: 38.5 (3.1)<br><br>/ level III		nurse CIMI (certified infant massage instructor) and were asked to participate in at least 3 bedside massage instruction sessions taught within the next week. Infants received massage for 7 consecutive days, from the mother or a CIMI. The touch procedure lasted 20 minutes.<br><br>Control: Infants received all usual hospital services including medical care, physical and occupational therapy services and developmentally supportive nursing care.	***2. Caregiver satisfaction with the neonatal unit and the massage therapist***	- Upon completing the 7-day massage program<br><br>- 1 month following intervention	Validation: No content validity or reliability testing reported.<br><br>-1st questionnaire (at baseline): a brief self-report questionnaire about caregiver satisfaction with their infant's care until that moment. No further details reported.<br><br>-2nd questionnaire (upon completing the 7-day massage program and 1 month following intervention): a 10-minute satisfaction questionnaire relating to infant's response and caregiver satisfaction with the neonatal unit and the massage therapist.<br><br>Number of questions: not stated.<br><br>Likert scale (1 very dissatisfied-4 very satisfied).<br><br>Sample statements: *'How satisfied do you feel giving massage to your infant?'; 'I feel that massage improved my infant's hospital stay.'*	At baseline and day 7: All caregivers were highly satisfied with the medical treatment their infant received.<br><br>At day 7 and 1 month follow-up: All caregivers participating in the massage group reported high levels of satisfaction regarding their relationship with their infant and the massage program's impact on that relationship.<br><br>Slight improvements in satisfaction regarding time the caregiver spent with the infant and involvement in the infant's care were observed between day 7 and the 1-month follow-up (no further information reported).		
8. Koh et al. (2007), Australia	Mothers /200	Not stated / not stated	Randomised, Controlled Trial	Intervention: **Provision of taped conversations with neonatologists to mothers.**<br><br>The initial conversation and subsequent conversations of significance with a neonatologist were taped and analysed (for both groups). Mothers received a tape of each conversation and a tape recorder.<br><br>Control: Usual care. Mothers were not given the tape or recorder.	***Satisfaction with conversations held with the neonatologist***<br><br>***Satisfaction with the tape***	During admission period and post discharge<br><br>- At 10 days<br><br>- At 4 months<br><br>- At 12 months<br><br>No pre-intervention parent satisfaction data available for comparison.	*Individual questions and a satisfaction scale*<br><br>Validation: No content validity or reliability testing reported.<br><br>Number of questions: not stated.<br><br>Likert scale (1-5 most satisfied)<br><br>Questions related to: Satisfaction with amount and quality of information presented, doctors' communication skills, patient's participation in the conversation.<br><br>A satisfaction scale was used to assess: Satisfaction with the tape	No differences were found between the two groups in satisfaction with conversations.<br><br>**Mothers of babies with a poor outcome in the tape group were, however, significantly more satisfied with the conversations:**<br><br>**Interv** **Control**<br>**Mean (95%CI)** 115(104-123.2) 100.5(94.1-109.4)<br><br>**p-value** 0.0051<br><br>Most (71-92%) of the mothers given the tapes stated that they helped their understanding, reminded them of what had been said, and helped their family to understand and recall information.	**No**	**1**

Confidential: For Review Only

9. Mitchell-DiCenso et al. (1996), Canada	Mothers and fathers/ 482	Mean (SD)<br><br>Interv: 35.1 (4.5)<br><br>Control: 35 (4.3)<br><br>/ level III	Randomised, Controlled Trial	Intervention: **Clinical Nurse Specialist/ neonatal practitioner team (CNS/NP) care.**<br><br>Infants of intervention parents were assigned to be cared for by the Clinical nurse special/neonatal practitioner CNS/NP team during the day and by paediatric residents during the night.<br><br>Control: Paediatric residents cared for infants of control parents around the clock. Neonatologists supervised both teams.	***Parent satisfaction with care***	During admission period and post discharge (twice)<br><br>- On 5th day after admission (full survey)<br><br>- After discharge over the phone (only questions related to satisfaction with discharge process)<br><br>No pre-intervention parent satisfaction data available for comparison.	*Satisfaction questionnaire (Validated)*<br><br>The study team developed and used the validated *Neonatal Index of Parent Satisfaction (NIPS)* questionnaire.<br><br>Number of questions: not stated.<br><br>NIPS score range (27-189); higher scores indicating greater satisfaction with care.	**No statistically significant difference between groups.**<br><br>**Interv Control p-value**<br>**NIPS Mean** 140 139 0.67<br><br>**Difference in means** 1.0, CI (-3.6-5.6)	**No**	**2**
10. Broyles et al. (1992), USA	Mothers /25	Mean (SD)<br><br>Control: 34 (4)<br><br>Interv: 33.4 (4)<br><br>/ level III	Randomised Controlled Trial	Intervention: **Detailed consent.**<br><br>Mothers were given information about mechanical ventilation. Detailed risk/benefit disclosure was provided both verbally and in writing.<br><br>Control:<br>Mothers were given a brief verbal description about mechanical ventilation supplemented with detailed verbal and written disclosure if desired by them (flexible consent).	***Maternal satisfaction with the information provided about mechanical ventilation***	During babies' admission (once)<br><br>- 24-48 hours after the intervention<br><br>No pre-intervention parent satisfaction data available for comparison.	*An interview* evaluating maternal satisfaction with the information provided about mechanical ventilation.<br><br>Validation: A psychiatrist with a special interest in interviewing techniques was consulted in designing and standardising this assessment.<br><br>A research nurse conducted the interview, "checking" each mother against one option regarding:<br>- Amount of information: Right amount-Too much-Too little<br><br>- Information made coping: More Difficult-Easier-No effect-Uncertain.	This study is measuring and comparing satisfaction with two different interventions (detailed vs flexible consent process), neither of which formally represent the usual routine care for all babies (no control).<br><br>**Small numbers. No data indicating statistical analysis conducted or evidence of statistically significant results.**<br><br>**Detailed Flexible**<br>**Right amount of information** 75% mothers 100%<br><br>**Too little information** 25% mothers<br><br>**Made coping easier** 67% mothers 69%	**No**	**3**

Confidential: For Review Only

Non-Randomised controlled trials (Non-RCT) by publication year

Author (Date), Country	Parents' gender/ sample Size	Infant Gestation age (GA) in weeks /NICU level	Study design	Intervention	Outcome measures	Timing of measurement	Method of measurement	Results	Parent co-design?	Improved parent satisfaction?
1. De Bernardo et al (2017), Italy	Mothers and Fathers /96	Mean (SD)<br><br>Control: 34.2 (5.25)<br><br>Interv: 32.7 (5.25)<br><br>/ level III	Non-randomized, prospective cohort pilot study<br><br>*Unit level effect*:<br>Two different time periods	Intervention: **FCC (Family-Centered Care).**<br><br>Parents had access to NICU for 8 hours/day. The NICU was widened and paediatric nurses taught parents procedures/practices for 10 days. Parents could observe clinical bedside rounds, hold meetings with the physicians, use the rooms and kitchen.<br><br>Control: Parents were permitted to visit their baby in NICU for 1 hour a day.	***Parent satisfaction relating to 3 specific domains:***<br><br>1. Knowledge and Understanding<br><br>2. Communication and Collaboration<br><br>3. Privacy and confidentiality	During babies' admission (once)<br><br>- At discharge (pre-FCC cohort and post-FCC cohort)<br><br>No pre-intervention parent satisfaction data available for comparison (different parent groups pre and post intervention).	*Satisfaction questionnaire.*<br><br>Validation: The authors state the survey *"was designed and validated by Abdel-Latif et al"*. No content validity or reliability testing reported in the original paper.<br><br>9 questions<br><br>3 questions: Related to adequate and timely information about the baby's condition.<br><br>3 questions: Related to communication and collaboration with the healthcare team.<br><br>3 questions: Related to respect of patient privacy.<br><br>Likert (1 strongly disagree-5 strongly agree)	**7/9 individual statements in the parent satisfaction questionnaire scored higher in the FCC compared to the NFCC (statistically significant difference).**<br><br>Example statement:<br>*"I have received adequate information about my baby's condition and management."*<br><br>**Interv** **Control**<br>**Median** 5 (3.45-5) 4 (3-5)<br><br>**p-value** <0.05	**No**	**1**
2. Kadivar et al. (2017), Iran	Mothers /68	<=30 – 36<br><br>/ level not stated	Non-randomised, Convenience sampling.<br><br>*Group level effect:*<br>Intervention/ control groups.<br><br>Pre and post-intervention testing.	Intervention: **Internet-based education.**<br><br>Mothers used an educational website set up by the research team (files and clips). Mothers could visit the website from 5:00-6:00 pm for 10 days. They were also allowed to use the website outside of the above hours and to report the duration of using the website to the researcher. Mothers had	***Maternal satisfaction***	During babies' admission (twice)<br><br>- Day 1 of intervention<br><br>- Day 10 of intervention	*Satisfaction questionnaire (Validated)*<br><br>The *"What Being The Parent of a Baby is Like-Revised" Questionnaire (WBPL- Revised)* was used. The original English version by Pridham and Chang was translated to Persian.<br><br>11 questions<br><br>Total satisfaction score range (11–99)	There was a significant difference in the mean score of satisfaction between cases and controls while the mean score of satisfaction increased in both groups.<br><br>**Comparison of the mean score between the two groups showed that the level of satisfaction was significantly higher in the case group versus the control group.**	**No**	**1**

Confidential: For Review Only

				to use the website at least 3 times during 10 days, each time for at least 30 min.<br><br><u>Control:</u> Mothers in the control group received the routine education provided in the NICU.				**Interv Control**<br>**before intervention**<br>**Mean (SD)** 81.62(13.50) 85.71(9.46)<br>**p-value** 0.993<br><br>**after intervention**<br>**Mean (SD)** 93.88 (5.38) 90.12 (7.78)<br><br>**p-value** 0.024		
3. Kadivar et al. (2017), Iran	Mothers /70	Mean (SD)<br><br>Control 31.6 (2.4)<br><br>Interv: 32.9 (3.1)<br><br>/ level not stated	Non-randomised, Convenience sampling.<br><br>*Unit level effect*:<br>Two different time periods	<u>Intervention:</u> **Narrative writing.**<br><br>Mothers did narrative writing at least 3 times until the 10th day of admission.<br><br><u>Control:</u> Mothers in the control group received the routine NICU treatment and care.	***Mothers' satisfaction with medical care provided by physicians, medical students, and nurses during neonatal admission to the NICU***	During babies' admission (twice)<br><br>- Day 3 of intervention<br><br>- Day 10 of intervention	*Satisfaction questionnaire (Validated)*<br><br>The *NIPS questionnaire* by Mitchell et al was used and translated to Persian.<br><br>24 questions (Likert scale)<br><br>Likert (1 always or not satisfied-7 never or completely satisfied). A higher score indicates more satisfaction.	**The satisfaction level of the mothers in the intervention group increased significantly during the study.**<br><br>The results of independent t test showed a significant difference in the satisfaction changes of the mothers on the 3rd and 10th day of NICU admission between intervention and control groups, indicating the effectiveness of narrative writing.<br><br>The results of paired t-test also showed a significant difference in the mean satisfaction level of the mothers between the 3rd and the 10th day in the intervention group.<br><br>**Interv Control**<br>**After intervention**<br><br>**Mean (SD)** 137 (15.2) 102.3 (25.6)<br><br>**p-value** 0.001	**No**	**1**
4. Garingo et al. (2016), USA	Not stated /9	23-39 / level III	Non-randomised, Convenience sampling.<br><br>*Group level effect:*<br>Intervention/ control groups<br><br>Post-intervention group testing	<u>Intervention:</u> **Tele-rounding.**<br><br>Infants of intervention parents were cared for by an OFFSN (off site neonatologist) who was present via a remote-controlled robot. The OFFSN assessed infants via the robot's integrated stethoscope, with assistance from the nursing staff. During	***Satisfaction with telemedicine***	During babies' admission (once)<br><br>- At the time of discharge<br><br>No pre-intervention parent satisfaction data available for comparison.	*Satisfaction questionnaire*<br><br><u>Validation:</u> No content validity or reliability testing reported.<br><br><u>Number of questions:</u> not stated.<br><br>Likert (1 excellent-5 very poor).	**Only the intervention group was assessed and only post-intervention.**<br><br>The authors reported that the parents surveyed were *"satisfied with their experience. 100% responded that they felt comfortable talking to the OFFSN on the mobile robot and would allow their infant or themselves to be cared for by a physician via telemedicine in the future."*	**No**	**4**

Confidential: For Review Only

			only	routine hours the OFFSN was called to discuss any issues with the patient. Emergencies/out of hours were covered by an ONSN (on site neonatologist).<br><br>Control: Infants of control parents received ONSN care. The attending neonatologist made daily patient rounds with the NICU team. After patient rounds, the NICU staff, under the supervision of the attending neonatologist implemented the care plan.						
5. Globus et al. (2016), Israel	Mothers and fathers /Total surveys returned: 178	~40% in each group <32 / level III	Non-randomised, Convenience sampling.<br><br>*Unit level effect:*<br>Two different time periods	Intervention: **SMSi-Short Message Services Implementation.**<br><br>Parents were updated daily regarding the health status of their infant via SMS (short-message-services) from the Electronic Patient Record. All SMS messages were sent at 09:00am, including one-sentence sections with updated information (e.g. location of the infant's crib and current weight). Information regarding acute events/deterioration of the infant's medical condition was not included in the SMS, but was delivered personally to the parents in real time.<br><br>Control: Routine care pre-SMS implementation.	***1. Parent satisfaction related to parent communication with the medical staff***<br><br>***2. Overall parent satisfaction with treatment and staff attitudes throughout hospitalisation.***	During babies' admission (once)<br><br>- pre-SMS cohort and post-SMS cohort<br><br>No pre-intervention parent satisfaction data available for comparison (different parent groups pre and post intervention).	*Satisfaction questionnaire*<br><br>The *"Parents' attitudes regarding their experience during their infants' hospitalisation in the NICU"* questionnaire was used, as well as selected items from a literature review of similar questionnaires, including that by York Hospital and by Conner and Nelson.<br><br>Validation: No content validity or reliability testing reported.<br><br>Selected items related to four aspects of the NICU experience. 2 out of 4 directly assessed parent satisfaction:<br><br>1. Parental assessment of their communication with the medical staff.<br><br>Likert scale (1 do not agree at all-5 strongly agree)<br><br>2. Overall satisfaction with treatment and staff attitudes throughout hospitalisation.<br><br>Visual analog scale (scores range 0-10). Higher scores reflect greater satisfaction.	Overall, in both periods, parents expressed a high degree of satisfaction regarding the medical treatment, the information given and the communication with the medical staff. Overall satisfaction with treatment and with staff attitudes throughout hospitalisation was slightly greater in the post-SMS cohort but did not reach statistical significance.<br><br>**In the post-SMS cohort, a statistically significant improvement was noted regarding physician availability and patience, parental feelings of comfort in approaching the physicians and nurses, and regularly receiving information regarding the infants' medical status from the physicians.**<br><br>**Post SMS** **Pre SMS**<br>**Mean (SD)** 4.1 (1.0) 3.7 (1.3)<br>**p-value** 0.03<br>*Specific question: "I was pleased with the frequency with which I received information regarding my infant".*	**No**	**1**

Confidential: For Review Only

								Although improvement in all other categories was documented, it did not reach statistical significance.		
6.Kazemian et al. (2016), Iran	Mothers /220 newborns (assumed 220 mothers)	>37 / level not stated	Non-randomised, Convenience sampling.<br><br>*Group level effect:* Intervention/ control groups<br><br>Post-intervention testing only	Intervention: **Rooming-in care.**<br><br>Mothers and babies were admitted to a different atmosphere to the routine care. This facilitated the mothers and neonates with separate beds along with phototherapy devices and nursing clinical supervision.<br><br>Control: The routine care practiced in this neonatal unit supported partial stay of mothers beside their neonates, while sitting on chairs; however, most of the time the mother-infant dyad was separated.	***Maternal satisfaction with the neonatal care services and hospital stay comfort***	During babies' admission (once)<br><br>-Not stated exactly when<br><br>No pre-intervention parent satisfaction data available for comparison.	*Satisfaction questionnaire*<br><br>Validation: No content validity or reliability testing reported.<br><br>The authors state, *"a validated self-made questionnaire was employed, which was filled in by some trained midwives."* No further information on validation processes, number of questions or name of the questionnaire was provided.<br><br>Likert (5 very satisfied-1 dissatisfied).	**The level of satisfaction was significantly higher in the intervention group, compared to that in the control group.**<br><br>**Interv** **Control**<br>**Satisfaction %** 26.6 18.8<br><br>**p-value** 0.027	**No**	**1**
7. Petteys et al. (2015), USA	Not stated/ 10 parents included in sample analysis	24-36+ / level III	A prospective cohort design.<br><br>A feasibility study.<br><br>*Group level effect:* Intervention/ control groups<br><br>Post-intervention testing only	Intervention: **PC (Palliative care).**<br><br>PC nurses provided important continuity of care for NICU infants clinically requiring PC and at least weekly verbal support of parents. The PC service also coordinated family conferences, provided or requested orders to improve infant symptom management and comfort, and addressed parental coping and self-care.<br><br>Control: Usual clinical care for infants not requiring PC.	***Overall satisfaction with care received***	During babies' admission (once)<br><br>- At discharge (or study closure for infants who remained hospitalised)<br><br>No pre-intervention parent satisfaction data available for comparison.	*Satisfaction questionnaire*<br><br>A researcher-created questionnaire based on extensive current literature review.<br><br>Validation: Partially reported. Authors stated content validity testing took place; no information on reliability testing provided.<br><br>1 question<br><br>Likert (1 extremely dissatisfied-4 to extremely satisfied).<br><br>Optional free text (description of specific experiences impacting satisfaction with care)	**Parent satisfaction response numbers were small (n= 10), thus statistical comparison of parental satisfaction between cohorts was not possible.**<br><br>However, 100% of responding PC parents (n= 2) reported being "extremely satisfied" with care, whereas only 50% of responding usual care parents (n= 4) reported extreme satisfaction.	**No**	**3**

Confidential: For Review Only

8. Van de Vijver and Evans (2015), UK	Not stated /105	Not stated / not stated	Non-randomised, Convenience sampling.<br><br>*Unit level effect:* Three different time periods	Intervention: **Baby diary.**<br><br>Each parent received a communication diary on their infant's admission to the unit. Staff wrote-in infant status updates and kept an infant interaction log with parents. Parents wrote in memories and questions for staff to address during face-to-face communication.<br><br>Control: Routine care, before implementation of the diaries.	***Satisfaction with communication from neonatal staff***	During babies' admission (three times)<br><br>- On the day of babies' discharge at study baseline<br><br>- On the day of babies' discharge at 1 month<br><br>On the day of babies' discharge at 15 months	*Satisfaction questionnaire*<br><br>The study team designed a questionnaire, based on the Department of Health and the National Institute for Health and Care Excellence (NICE) quality standards for specialist neonatal care.<br><br>Validation: No content validity or reliability testing reported.<br><br>5 questions ("yes or no")	**Small numbers. No data indicating statistical analysis conducted or evidence of statistically significant results.**<br><br>*"I was receiving regular communication from staff"*<br>94% - 1 month post diary cohort<br>93% - 15 months post diary cohort<br>77% - pre diary cohort<br><br>*"My questions and concerns were being addressed"*<br>100% - 1 month post diary cohort<br>93% - 15 months post diary cohort<br>91% - pre diary cohort<br><br>"I feel more involved in my baby's care"<br>92% - 1 month post diary cohort<br>100% - 15 months post diary cohort<br>88% - pre diary cohort	**Yes.**<br><br>The interventi on concept was created by the project leaders following analysis of baseline survey results and used after multi-disciplina ry input and discussion with staff and parents.	3
9. Voos and Park. (2014), USA	Not stated / 62	Not stated / level III	Non-randomised, Convenience sampling.<br><br>*Unit level effect:* Two different time periods	Intervention: **OU (Open Unit) policy.**<br><br>Parents were allowed access to their baby 24 hours a day, 7 days a week.<br><br>Control: Parents pre-OU implementation received routine care. The unit was closed to parents during nurse change of shift in mornings and evenings.	***Parent satisfaction with how much time parents get to spend with their baby***	After babies were discharged (once)<br><br>- After pre-OU parents were discharged<br><br>- After post-OU parents were discharged	*Single question (From a validated questionnaire)*<br><br>The question *"Did you get to spend as much time as you wanted with your baby?"* was used from the NRC (National Research Corporation) Picker parent survey.<br><br>1 question ("yes or no")	**Small numbers. No data indicating statistical analysis conducted or evidence of statistically significant results.**<br><br>*"Did you get to spend as much time as you wanted with your baby?" Yes.*<br><br>Pre OU 78% (18/23)<br>Post OU 92% (36/39)	**Yes.**<br><br>The NICU has a Family-centered care committe e including parents, which conducted this project.	3
10. Segre et al. (2013), USA	Mothers /23	Mean (SD) 31.57 (5.30) / level III	For the outcome of parent satisfaction:<br><br>Non-Randomised, Convenience sampling.<br><br>*Group level effect:* Intervention/	Intervention: **(LV) Listening visits.**<br><br>Mothers met with the LV provider for up to six 50-min LV sessions, conducted in a private hospital, every 2–3 days, within 1-month. Visits entailed greeting, debriefing, updating on current issues, working an agenda through listening and problem solving, and providing closure through	***Satisfaction with the treatment and the outcome.***	During babies' admission (once)<br><br>- Not stated exactly when<br><br>No pre-intervention parent satisfaction data available for comparison.	*Satisfaction questionnaire*<br><br>The *Client Satisfaction Questionnaire* was used.<br><br>Validation: Partially reported. Authors stated reliability testing took place; no information on content validity provided.<br><br>8 questions.<br><br>Format of questions: not stated	**Only the intervention group was assessed and only post-intervention.**<br><br>The authors reported:<br><br>*"The majority of women who received LVs were highly satisfied with the intervention".*<br><br>*"The average score for the Client Satisfaction Questionnaire was 29.91, comparable to levels of satisfaction reported by clients receiving depression treatment from a mental health professional."*	**No**	4

Confidential: For Review Only

			control groups<br><br>Post-intervention group testing only	summary.<br><br><u>Control:</u> Women who did not meet the specific criteria (e.g. minimum score on depression scale) were not invited to join the treatment trial and received routine NICU care/support instead.				*"91.3% of our participants rated the quality of help they received as excellent."*		
11. Palma et al. (2012), USA	Not stated / 26 families returned the survey containing the satisf. measure)	Not stated / level II	Non-randomised, Convenience sampling.<br><br>*Unit level effect:* Two different time periods	<u>Intervention:</u> **YBDU (Your Baby's Daily Update). A daily parent update letter generated from the Electronic Medical Record (EMR).**<br><br>Parents were given daily YBDU reports, printed automatically from the EMR. The YBDU included information about an infant's status during the past 24 hours and a hand-written update by the infant's care provider.<br><br><u>Control:</u> Parents received routine care and usual verbal updates (6 months pre- adoption of YBDU).	***Satisfaction with YBDU***	During babies' admission (once)<br><br>- Not stated exactly when<br><br>No pre-intervention parent satisfaction data available for comparison (different parent groups pre and post intervention).	*Satisfaction questionnaire*<br><br>A questionnaire including items regarding adoption of and satisfaction with YBDU was used.<br><br><u>Validation:</u> No content validity or reliability testing reported.<br><br><u>Number and format of questions:</u> not stated.	**Only the intervention group was assessed and only post-intervention.**<br><br>The authors reported:<br>*"When asked to rate the statement "I like receiving Your Baby's Daily Update", 96% of families who used YBDU as an information source responded with the highest rating, "always"."*	**No**	**4**
12. Stevens et al. (2011), USA	Mothers /147. For the OPBY NICU, 58 surveys were returned. For the SFR NICU, 89 were returned	Mean (SD)<br><br>Control: 35 (4)<br><br>Interv: 34 (3)<br><br>/ level not stated	Cohort trial. This research was part of a large prospective evaluation.<br><br>*Unit level effect:* Two different time periods	<u>Intervention:</u> **SFR (Single-family room) NICU for neonatal care.**<br><br>Parents could visit their baby, room-in, do kangaroo care and breastfeed at any time, in individual rooms (containing bed, desk, closet, telephone, chair, refrigerator for breast-milk storage).<br><br><u>Control:</u> OPBY (Open-bay) NICU. The traditional open-bay NICU was typical of facilities built before 1980. All neonates, family members, staff, monitors, and equipment were visible for all neonates in each room. Portable partitions were placed	***Parent satisfaction with different elements of NICU:***<br>*- Delivery*<br>*- Environment*<br>*- Nurses*<br>*- Physicians*<br>*- Discharge*<br>*- Personal*<br>*- Overall Assessment*	After babies were discharged (once)<br><br>- Mailed within 60 days of discharge of parents' infants from the NICU<br><br>No pre-intervention parent satisfaction data available for comparison (different parent groups pre and post intervention).	*Satisfaction questionnaire*<br><br>A questionnaire from Press Ganey Associates was used. Also included were three questions added by the investigators.<br><br><u>Validation:</u> Partially reported. The original questionnaire was validated questionnaire but no content validity or reliability testing was reported regarding the 3 questions added by the study team.<br><br>42 questions in total (7 categories):<br>Delivery, Environment, Nurses, Physicians, Discharge, Personal,<br>Overall Assessment.<br><br>Likert (1 very poor-5 very	**Statistically significant improvement was found for the survey categories of Environment, Overall and the Total survey.**<br><br>Estimated numbers from report's figures as numbers not provided):<br><br>**Median SFR OPBY p-value**<br>Environment 4.7 3.7 <0.001<br>Overall 5 4.8 0.018<br>Total 4.7 4.5 0.045<br><br>16 items composite score for family-centered care:<br>4.4 4.0 0.017	**Yes**<br><br>Former NICU parents were involved in all phases of planning for the new SFR NICU.	**1**

Confidential: For Review Only

				around the incubator for breastfeeding and kangaroo care.			good).		
13. Voos et al. (2011), USA	Not stated /28	Not stated / level not stated	Non-randomised, Convenience sampling.<br><br>*Unit level effect:*<br>Two different time periods	<u>Intervention:</u> **Family-centered rounds (FCRs).**<br><br>Parents were invited to attend rounds and choose their level of involvement (attend every day/not at all/periodically). For confidentiality concerns, parents were asked to step outside while rounds of others' infants took place. The staff augmented FCRs by meeting with parents again after rounds if needed.<br><br><u>Control:</u> Parents received routine care. Prior to FCR implementation parents were asked to leave the unit during rounds.	***Global satisfaction with the NICU experience***	During babies' admission (twice)<br><br>- Prior to FCR<br><br>- 6 months after starting FCR	*Satisfaction questionnaire (Validated)*<br><br>*The NIPS* questionnaire.<br><br>24 questions: looking at satisfaction in different areas of the NICU (medical caregivers, communication, tests, and procedures).<br><br>Likert scale (1-7 points).	**A subset of NIPS items related to communication (i.e. being kept informed as to changes in the infant's condition, meeting with physicians, and information about long-term expectations) yielded a significant increase from pre to post FCR scores.**<br><br>**post-FCR pre-FCR p-value**<br>**NIPS score** 5.5 4.4 <0.01<br><br>The average score on the NIPS did not change significantly.	**No**	**1**
14. Weiss et al. (2010), USA	Mothers /84	Mean (SD)<br><br>Pre-interv group: 32 (4.4)<br><br>Post-interv group: 32 (9)<br><br>/ level III	Non-randomised, Convenience sampling<br><br>*Unit level effect:*<br>Two different time periods	<u>Intervention:</u><br>**An intervention to increase PMP (Principal Medical Providers) availability and communication frequency.**<br><br>(1) A brief education module for PMPs was introduced (2) parents received a contact card with PMP names, job descriptions and contact information (3) a poster of the faces, names and titles of the PMPs was placed at NICU entrance.<br><br><u>Control:</u> Parents received routine care in the pre-intervention cohort, without the above.	***Parent satisfaction with physician and nurse practitioner communication***	During babies' admission (twice)<br><br>- Pre-intervention<br><br>- Post-intervention	*Satisfaction Questionnaire (Validated)*<br><br>A pilot survey written by Press Ganey and the Picker Institute was used and revised based on parent responses.<br><br>6 open-ended questions (Quantity of communication)<br><br>6 Likert scale questions (range questions (Availability, understanding, reciprocity, empathy, overall satisfaction)	**Overall satisfaction, based on the ordinal analysis of the five-point Likert scale, was significantly higher after the intervention (P<0.01).**<br><br>**Overall satisfaction, dichotomised into a satisfied subgroup and a dissatisfied subgroup for each cohort, was also significantly increased after the intervention.**<br><br>**post –interv pre-interv**<br>**Very satisfied/ Somewhat satisfied** 97%(32/33) 74%(37/50)<br><br>**p-value** <0.01	**No**<br><br>Authors stated that only after trialing the intervention on many parents (both satisfied and unsatisfied) gave suggestions to improve it.	**1**
15. Foster et al. (2008), Australia	Mothers and fathers /93<br><br>5 Special Care	Mean (SD)<br><br>Headbox: 36.5 (2.6)	Non-randomised, Convenience sampling<br><br>*Group level*	<u>Intervention 1:</u> **Infants received headbox oxygen treatment for respiratory distress.**<br><br><u>Intervention 2:</u> **Infants**	***Satisfaction with treatment (i.e. headbox oxygen or CPAP)***	During babies' admission (once)<br><br>- Within 5 days of the babies' admission	*Single question*<br><br><u>Validation:</u> No content validity or reliability testing reported.	**Parents with babies receiving CPAP rated their satisfaction with the baby's treatment statistically significantly higher than the headbox**	**No**	**1**

Confidential: For Review Only

	Nurseries	CPAP:<br>36 (3)<br><br>/level I	*effect:*<br>Intervention 1/ intervention 2 groups<br><br>Post intervention testing only	**received continuous oxygen positive airway pressure (CPAP) treatment for respiratory distress.**		No pre-intervention parent satisfaction data available for comparison.	1 likert scale question (1 not at all satisfied-5 extremely satisfied).	**group mean rating.**<br>**Headbox** **CPAP**<br>**Mean (SD)** 3.71 (1.31) 4.51 (0.79)<br>**p-value** 0.001<br><br>The CPAP group averaged between *very and extremely satisfied* compared with parents of babies receiving headbox, who averaged between *satisfied and very satisfied ratings.*		
16. Byers et al. (2006), USA	Only mothers reported /35	Preterm infants<br><br>Mean (SD)<br><br>Control: 28.9 (3.44)<br><br>Interv: 28.6 (3.37)<br><br>/ level II/III	For the outcome of parent satisfaction:<br><br>Non-randomised, Convenience sampling<br><br>*Group level effect:*<br>Intervention/ control groups<br><br>Post-intervention testing only	<u>Intervention:</u> **Infants received individualised, developmentally supportive family-centered care.**<br><br>Infants received care within the framework and philosophy of individualised, developmentally supportive family-centered interventions.<br><br><u>Control:</u> Infants received the traditional NICU standard of care.	***Parent satisfaction relating to:***<br>*- parental perceptions of staff caring*<br>*- education received*<br>*- preparation for the parental role*<br>*- overall satisfaction with the NICU experience*	During babies' admission (once)<br><br>- On the day before discharge<br><br>No pre-intervention parent satisfaction data available for comparison.	*Satisfaction questionnaire*<br><br>The *NICU's parental satisfaction tool* was used.<br><br><u>Validation:</u> Partially reported. Authors stated content validity testing took place, but "because of the disparate nature of the items, survey reliability was not assessed".<br><br>11 questions<br><br>Likert scale (1-5 strongly agree)	**Independent t-test analysis of parent satisfaction/perception scores showed no significant difference between groups.**<br><br>Example statement: "*I was satisfied with the car my baby and I received in the NICU"*<br><br>**Interv** **Control**<br>**Mean (SD)** 4.94(0.23) 4.71(0.47)<br>**p-value** 0.064<br><br>Both groups reported very high satisfaction with their NICU experience (4.4-5.0)	**No**	**2**
17. Mills et al. (2006), USA	Not stated/ not stated<br><br>Parents of infants from 6 hospitals	Not stated / level not stated	Implementation project<br><br>Plan Do Study Act (PDSA) quality improvement testing	<u>Intervention:</u> **5 potentially better practices (PBPs) in the area of discharge planning.**<br><br>The project team iteratively implemented 5 PBPs:<br>1. Created an easy-to-use, easy-to-access discharge planning tool kit.<br>2. Restructured communication tools and processes to reflect a "plan for the day, the stay, and the way" to discharge.<br>3. Maximised the impact and use of caregiver educational tools, and updated materials and delivery systems for caregiver education.<br>4. Used various continuous quality improvement tools and processes to ensure	***General satisfaction***<br>*- with care*<br>*- parents' feelings about preparedness for discharge*<br>*- ability and confidence in feeding*<br>*- familiarity with their infant*<br>*- feeling like a parent*<br>*- participation in care*<br>*- adequacy of information from staff about medical and care issues*	During babies' admission (4 times)<br><br>- Not reported exactly when	*Satisfaction questionnaire*<br><br>The Internet-based parent satisfaction survey *"howsyourbaby.com"* that was developed especially for this NICU population was used.<br><br><u>Validation:</u> No content validity or reliability testing reported.<br><br><u>Number and format of questions:</u> not stated.	Through multiple rapid-cycle projects, the project's collaborative group made changes within the 5 PBP plans.<br><br>**Parent satisfaction measures were used to longitudinally monitor the changes made, rather than make direct group comparison. No data indicating statistical analysis conducted or evidence of statistically significant results.**<br><br>Parent satisfaction survey results (all centers combined) were high across 4 measurement quartiles. No specific interquartile analysis was reported.<br><br>Parent readiness for discharge was high at the beginning and throughout the collaborative. Parents' receiving *"just the right amount of information"* regarding car seat trials and	**No**	**3**

Confidential: For Review Only

				parent/caregiver and staff satisfaction.<br>5. Analysed and enhanced interactions with and transfers into the community.<br><br><u>Control:</u> N/A. No discrete control group. PDSA quality improvement methodology was applied to parent participants.				safe sleep demonstrated some variability throughout the collaborative.		
18. Wielenga et al. (2006), The Netherlands	Mothers and fathers / 46	Mean (SD)<br><br>Control: 28.5 (26.0–29.9)<br><br>Interv: 28.3 (25.6-29.9)<br><br>/ level III	Non-randomised, Convenience sampling<br><br>*Unit level effect:*<br>Two different time periods	<u>Intervention:</u> **The Newborn Individualised Developmental Care and Assessment Program (NIDCAP).**<br><br>Infants received care according to NIDCAP principles and parents were taught how to provide it. Caregiving plans were designed based on the infant's current developmental stage, medical condition and family needs. Caregivers learnt to watch sensitively and note the infant's reactions to different types of handling and care, making continuous adjustments.<br><br><u>Control:</u> Infants received traditional neonatal care practiced at that time.	***Parent satisfaction relating to:***<br>*-Overall rating*<br>*-Care of the baby*<br>*-Communication with staff*<br>*-Involvement in care -Being prepared -Support*<br>*-Being a parent*<br>*-Being near your baby -Total score*	After babies were discharged (on day of discharge/ transfer)<br><br>- Pre NIDCAP cohort<br><br>- Post NIDCAP cohort	*Satisfaction questionnaire (Validated)*<br><br>The *NICU-PSF* was used and translated from English to Dutch.<br><br>62 questions<br><br>Closed and open-ended questions.<br><br>Different rating scales used (5-point rating scale from "extremely satisfied" to "not at all satisfied" or "excellent" to "poor").<br><br>Total score range (50-243 points)	**The intervention group's mean total score was significantly higher than the control.**<br><br>**Interv Control**<br>**Mean (SD)**<br>185.67(17.74) 174.04(20.98)<br><br>**p-value** 0.041<br><br>Almost all separate concepts showed an increase in their mean scores. The concept of "*being a parent"* had a slightly lower mean score (9.39, SD = 1.73) in the intervention group than in the control group (9.78, SD = 2.09).<br><br>**The concept of *"preparedness"* showed statistically significant difference**:<br><br>**Interv Control**<br>**Mean** 16.38 13.83<br>**p-value** 0.038	**No**	**1**
19. Penticuff and Arheart. (2005), USA	Dyads (both parents or mother with her support person)/ 122 mothers<br><br>Results based only on mothers' data.	Not stated /<br>Level III	A repeated measures design<br><br>- First 2 years (control group data collection)<br><br>- Year 3 (staff training)<br><br>- Year 4 (implementing the intervention)<br><br>- Year 5 (collecting data	<u>Intervention:</u> **The Newborn Individualised IPC- CPM intervention (Infant Progress Chart) - (Care Planning Meetings).**<br><br>Both the mother and father (or the mother and her designated support person) were shown how to use the Infant Progress Chart and attended 3 Care Planning Meetings (with neonatologists/Neonatal Nurse Practitioners).	***Satisfaction with participation in decision making*** **was measured by 5 collaboration indices:**<br><br>Satisfaction with<br>(1) Care<br>(2) Relationships with professionals<br>(3) Decision input<br>(4) The process of decision	During babies' admission (three times)<br><br>- Within 0–3 days<br><br>- 9– 12 days<br><br>- 25–28 days of an infant's admission to the NICU	*Three satisfaction questionnaires*<br><br>1. Two subscales of the investigator-designed *"Parents' Understanding of Infant Care and Outcomes Questionnaire"* were used to measure Satisfaction with Care (1).<br><br><u>Validation:</u> Partially reported. Authors stated content validity testing took place; no information on reliability testing provided.	**The intervention group was more satisfied with the amount of decision input they had (3) and with the process by which medical decisions were made (4).**<br><br>**Interv Control p-value**<br>Decision input amount (3)<br>**Mean** 33.44 30.05 0.058<br><br>Process of decision making (4)<br>**Mean** 120.20 104.95 0.012<br><br>There were no statistically significant differences between control and intervention groups in satisfaction with their infants' care (1), with relationships with	**No**	1

			from the intervention group)<br><br>*Unit level effect:* Two different time periods	Control: During the control phase, professionals carried out usual communication and interaction with control group parents.	making<br>(5) Decisions made		30 questions.<br><br>Five-point Likert scale.<br><br>2. A subscale of the investigator-designed *"Relationships with Professional and Decision Input Questionnaire"* was used to measure Satisfaction with relationships (2).<br><br>Validation: Partially reported. Authors stated content validity testing took place; no information on reliability testing provided.<br>12 questions.<br><br>Five-point Likert scale<br><br>3. Validated.<br>The *"Collaboration and Satisfaction About Care Questionnaire"* developed by Baggs, was used to measure Satisfaction with decision input (3), with decision process (4) and with decisions made (5).<br><br>9 questions.<br><br>7-point scale, (1 strongly disagree -7 strongly agree)	NICU professionals (2) and with the decisions made for infant treatment (5).		
20. Byers et al. (2003), USA	Mothers/ 19	Mean (SD)<br><br>Control: 29 (2.00)<br><br>Interv: 28.9 (2.42)<br><br>/ level II-III	For the outcome of parent satisfaction:<br><br>Non-randomised, Convenience sampling<br><br>*Group level effect:* Intervention/ control groups<br><br>Pre and post-intervention testing	Intervention: **Co-bedding premature multiple-gestation infants in incubators.**<br><br>Infants were nursed in the same incubator using a co-bedding protocol (e.g. recording all of the care provided to one infant before providing care to the second infant)<br><br>Control: Single-bedding premature multiple-gestation infants in incubators.	***Parent satisfaction related to:***<br>*- staff concern*<br>*- support of family*<br>*- staff explanations*<br>*- infant environment,*<br>*- comfort with feeding*<br>*- kangaroo care encouragement*<br>*- staff explanation of signs of infant stress*<br>*- visiting schedule*<br>*- overall satisfaction with the NICU*	During babies' admission (twice)<br><br>- At baseline<br><br>- 5 days later	*Satisfaction questionnaire*<br><br>The NICU's standard parental satisfaction tool was used.<br><br>Validation: Partially reported. Authors stated content validity testing took place, but because of the disparate nature of the items, survey reliability could not be assessed.<br><br>11 questions.<br><br>5-point Likert-type scale.	**The only significant difference for a post-intervention item was a higher score for the item "Attempts were made to create a quiet environment for my baby."**<br><br>**Interv Control p-value**<br>**Mean** 4.80 3.89 0.033<br><br>Independent t-tests comparing the co-bedded and control group parental scores found no significant differences in their parental satisfaction scores, except for higher baseline parental satisfaction scores (p=0.029) in the co-bedded group.	**No**	**1**

Confidential: For Review Only

					experience					
21. Polizzi et al. (2003), USA	Mothers and fathers/ 33	Mean (SD)<br><br>Control: 32.97 (1.9)<br><br>Interv: 33.08 (1.31)<br><br>/ level III	A retrospective, comparative, descriptive design.<br><br>*Unit level effect*	Intervention: **Co-bedding multiple-gestation infants in the NICU.**<br><br>Multiple-gestation infants were nursed in the same incubator or crib. The intervention was evaluated retrospectively after implementation of a co-bedding practice protocol.<br><br>Control: Traditionally-bedded group (babies were routinely placed in separate incubators or cribs)	***Parental satisfaction as measured by 9 questions relating to parent perceptions and their baby's care***	After babies were discharged (once)<br><br>- All parents were mailed the survey. A second survey was sent to those who did not respond after 2 months<br><br>No pre-intervention parent satisfaction data available for comparison.	*Satisfaction questionnaire*<br><br>The *parental perception/ satisfaction tool* was used.<br><br>Validation: Partially reported. Authors stated content validity testing took place; no information on reliability testing provided. 6/9 questions were from a similar tool that was validated by the Vermont Oxford NICU Quality Improvement Initiative.<br><br>9 questions (such as *"I was satisfied with the care my babies received in the hospital")*.<br><br>Likert (1 strongly disagree- 5 strongly agree)	Mothers reported overall satisfaction with the NICU care and staff, as well as adequacy of their ability to care for their infants after discharge, with scores ranging from 4.19 to 4.71.<br><br>**The only survey item score that was significantly different between groups was for the item *"I was encouraged by the hospital staff to bond with my babies."***<br><br>**Interv Control p-value**<br>**Mean** 4.71 4.36 0.049	**No**	**1**
22. Legault and Goulet. (1995), Canada	Mothers/ 61 completed both tests	Mean (range)<br><br>30 (24-35)<br><br>/ level II	Time-series design<br><br>*Group level effect:* Same group exposed to both methods with post-method testing only.	Intervention: **Kangaroo method of removing an infant from an incubator.**<br><br>Mothers were taught the "kangaroo method" (skin-to-skin contact): infant wears a diaper/head cap and is placed in a vertical position on the parent's bared chest. A blanket covers the infant and the parent's clothing is fastened around the infant. The parent sits in a rocking chair, inclined so that the infant's head is at 60'.<br><br>Control: Traditional method. Newborns wearing a diaper and a head cap, are wrapped in a blanket and placed in their parent's arms.	***Mothers' satisfaction with:***<br>*- Each method of removing an infant from incubator*<br>*- Her feelings after each method*	During babies' admission (twice)<br><br>- After the intervention<br><br>- After the control method<br><br>No pre-intervention parent satisfaction data available for comparison.	*Satisfaction questionnaire*<br><br>The *"Maternal Satisfaction Questionnaire"* was used. It was developed by integrating components described by Affonso et al and the clinical experience of the investigators.<br><br>Validation: Partially reported. Authors stated content validity testing took place; no information on reliability testing provided.<br><br>15 questions<br><br>Likert (1 very much-5 don't know)<br><br>An open-ended question invited the mother to list and explain anything else related to her experience.	**Regardless of the method tested, mothers expressed high levels of satisfaction (it was the first time since giving birth that they could hold their infants).**<br><br>Three statements proved more powerful in discriminating between the methods:<br><br>**Rated higher after the kangaroo method test:**<br>- *"I like the contact with my baby's skin"*<br>(p=0.0001)<br><br>**Rated higher after the traditional method test:**<br>- *"I like to talk to and whisper to my baby" (p = 0.015)*<br>- *"I looked into my baby's eyes and stared at his/her face" (p=0.0001)*	**No**	**1**

Confidential: For Review Only

BMJ Paediatrics Open

Interventions to improve quantitative measures of parent satisfaction in neonatal care: a systematic review

Journal:	*BMJ Paediatrics Open*
Manuscript ID	bmjpo-2019-000613.R2
Article Type:	Original research
Date Submitted by the Author:	03-Jan-2020
Complete List of Authors:	Sakonidou, Susanna; Imperial College London, Neonatal Medicine, School of Public Health, Faculty of Medicine<br>Andrzejewska, Izabela; Imperial College London, Neonatal Medicine, School of Public Health, Faculty of Medicine<br>Webbe, James; Imperial College London, Neonatal Medicine, School of Public Health, Faculty of Medicine<br>Modi, Neena; Imperial College London, Neonatal Medicine, School of Public Health, Faculty of Medicine<br>Bell, Derek; NIHR CLAHRC for Northwest London<br>Gale, Chris; Imperial College London, Neonatal Medicine, School of Public Health, Faculty of Medicine
Keywords:	Neonatology, Outcomes research, Patient perspective

SCHOLARONE™
Manuscripts

I, the Submitting Author has the right to grant and does grant on behalf of all authors of the Work (as defined in the below author licence), an exclusive licence and/or a non-exclusive licence for contributions from authors who are: i) UK Crown employees; ii) where BMJ has agreed a CC-BY licence shall apply, and/or iii) in accordance with the terms applicable for US Federal Government officers or employees acting as part of their official duties; on a worldwide, perpetual, irrevocable, royalty-free basis to BMJ Publishing Group Ltd ("BMJ") its licensees and where the relevant Journal is co-owned by BMJ to the co-owners of the Journal, to publish the Work in this journal and any other BMJ products and to exploit all rights, as set out in our licence.

The Submitting Author accepts and understands that any supply made under these terms is made by BMJ to the Submitting Author unless you are acting as an employee on behalf of your employer or a postgraduate student of an affiliated institution which is paying any applicable article publishing charge ("APC") for Open Access articles. Where the Submitting Author wishes to make the Work available on an Open Access basis (and intends to pay the relevant APC), the terms of reuse of such Open Access shall be governed by a Creative Commons licence – details of these licences and which Creative Commons licence will apply to this Work are set out in our licence referred to above.

Other than as permitted in any relevant BMJ Author's Self Archiving Policies, I confirm this Work has not been accepted for publication elsewhere, is not being considered for publication elsewhere and does not duplicate material already published. I confirm all authors consent to publication of this Work and authorise the granting of this licence.

Confidential: For Review Only

Interventions to improve quantitative measures of parent satisfaction in neonatal care: a systematic review

Susanna Sakonidou[1], Izabela Andrzejewska[2], James Webbe[3], Neena Modi[4], Derek Bell[5], Chris Gale[6]

1 Susanna Sakonidou, Clinical Research Fellow
Highest academic degree: MBBS
Neonatal Medicine, School of Public Health, Faculty of Medicine, Imperial College London, United Kingdom.
s.sakonidou@imperial.ac.uk

2 Izabela Andrzejewska, Neonatal Research Nurse
Highest academic degree: RN MSc
Neonatal Medicine, School of Public Health, Faculty of Medicine, Imperial College London, United Kingdom.
Izabela.ukpl@gmail.com

3 James Webbe, Clinical Research Fellow
Highest academic degree: MB BChir
Neonatal Medicine, School of Public Health, Faculty of Medicine, Imperial College. London, United Kingdom.
j.webbe@imperial.ac.uk

4 Neena Modi, Professor of Neonatal Medicine

Confidential: For Review Only

Highest academic degree: MD

Neonatal Medicine, School of Public Health, Faculty of Medicine, Imperial College London, United Kingdom.

n.modi@imperial.ac.uk

5 Derek Bell, Professor of Acute Medicine and NIHR CLAHRC Programme Director

Highest academic degree: MD

National Institute for Health Research Collaboration for Leadership in Applied Health Research and Care, Northwest London, United Kingdom.

d.bell@imperial.ac.uk

6 Chris Gale, Reader in Neonatal Medicine

Highest academic degree: PhD

Neonatal Medicine, School of Public Health, Faculty of Medicine, Imperial College London.

London, United Kingdom.

christopher.gale@imperial.ac.uk

Corresponding Author

Susanna Sakonidou, Clinical Research Fellow

Neonatal Medicine, School of Public Health, Faculty of Medicine, Imperial College London

369 Fulham Road, London, SW10 9NH, United Kingdom.

+ 44 (0) 203 315 5418, s.sakonidou@imperial.ac.uk

Confidential: For Review Only

Manuscript word count: 3245

ABSTRACT

Objective

Interventions improving parent satisfaction can reduce parent stress, may improve parent-infant bonding and infant outcomes. Our objective was to systematically review neonatal interventions relating to parents of infants of all gestations where an outcome was parent satisfaction.

Methods

We searched the databases MEDLINE, EMBASE, PsychINFO, Cochrane Central, CINAHL, HMIC, Maternity and Infant Care between 1/1/1946-1/10/2017. Inclusion criteria were randomised controlled trials (RCT), cohort studies and other non-randomised studies if participants were parents of infants receiving neonatal care, interventions were implemented in neonatal units (of any care level) and ≥1 quantitative outcome of parent satisfaction was measured. Included studies were limited to the English language only. We extracted study characteristics, interventions, outcomes and parent involvement in intervention design. Included studies were not sufficiently homogenous to enable quantitative synthesis. We assessed quality with the Cochrane Collaboration risk of bias tool (randomised) and the ROBINS-I tool (non-randomised studies).

Results

Confidential: For Review Only

We identified 32 studies with satisfaction measures from over 2800 parents and grouped interventions into 5 themes. Most studies were non-randomised involving preterm infants. Parent satisfaction was measured by 334 different questions in 29 questionnaires (only 6/29 fully validated). 18/32 studies reported higher parent satisfaction in the intervention group. The theme with most studies reporting higher satisfaction was parent involvement (10/14). Five (5/32) studies reported involving parents in intervention design. All studies had high risk of bias.

Conclusions

Many interventions, commonly relating to parent involvement, are reported to improve parent satisfaction. Inconsistency in satisfaction measurements and high risk of bias makes this low-quality evidence. Standardised, validated parent satisfaction measures are needed, as well as higher quality trials of parent experience involving parents in intervention design.

PROSPERO registration: CRD42017072388

Keywords: neonatology, parents, satisfaction

INTRODUCTION

One in 10 newborn babies in high-income countries require neonatal care[1]. This is stressful for parents, who often develop anxiety, depression and Post Traumatic Stress Disorder symptoms[2-4]. Parental stress interferes with parent-child bonding[5] and there is a well-established link between maternal mental health and infant development[6]. Parent satisfaction, defined as *"the perception of parents'*

Confidential: For Review Only

needs and expectations being met" is inversely related to parental stress[7]. As such, it is increasingly being used as a parent experience measure and neonatal service quality indicator. Interventions aimed at improving parent satisfaction have the potential to reduce parent stress, improve parent-infant bonding[8] and infant outcomes[9].

A range of parent-centred interventions, such as including parents on ward rounds, have recently become widespread in neonatal practice. Many are implemented on a small scale, without evaluating their impact on parent experience, making long-term integration into neonatal services challenging, while many others are using parent questionnaires. 'Parent satisfaction' as an outcome is gaining momentum, as neonatal trusts attempt to match more 'business-like models' where effectiveness of interventions (and evidence for change) is measured by quantitative outcomes. Moreover, where parent experience is measured as 'parent satisfaction', some studies include it as a primary outcome, whereas others use it as a secondary indicator to explore the parent point of view.

Furthermore, there are multiple experience measures available in addition to parent satisfaction, including parent stress, anxiety and depressions scales; both quantitative and qualitative. Finally, it is not known the degree to which parents are involved in the design of such interventions. There have been no previous systematic evaluations focused on interventions measuring parent satisfaction with neonatal care as an outcome.

The aim of this review is to identify and describe neonatal interventions relating to

Confidential: For Review Only

parents of infants of all gestations where an outcome was parent satisfaction. For the reasons outlined above, we have only included studies that reported ≥1 quantitative measure of parent satisfaction. We aim to report each intervention's effect on parent satisfaction, as well as parent input in intervention design.

METHODS

We prospectively registered this study on PROSPERO[10] (prospective register of systematic reviews-CRD42017072388) and reported it using PRISMA guidelines[11]. We searched MEDLINE (Medical Literature Analysis and Retrieval System Online), EMBASE (Excerpta Medica database), PsychINFO (Psychological Information), Cochrane Central Register of Controlled Trials, CINAHL (CUMULATIVE Index to NURSING and Allied HEALTH LITERATURE), HMIC (Health Management Information Consortium), Maternity and Infant Care (online_ supplementaryFile1) for English papers published between 1946-October 2017, with update searches on 1st September 2018.

Inclusion criteria were: randomised controlled trials (RCT) and non-randomised studies (non-RCT) if participants were parents of infants receiving neonatal care, interventions were implemented in neonatal units and ≥1 quantitative outcome of parent satisfaction was measured. We have restricted our review to studies where ≥1 quantitative outcome of parent satisfaction was measured, in order to enable comparison of interventions, which has previously not been possible in any published review. Including studies with all available measures of parent experience (in addition to parent satisfaction), as well as those only qualitatively evaluated, would make any comparison very difficult. By using these pre-registered search criteria, we

Confidential: For Revi

also ensured we would capture studies measuring parent satisfaction both as primary and as secondary outcomes. We included studies from all neonatal care level units and all healthcare settings, without excluding studies in low or middle-income settings. This was because definitions of neonatal care levels differ between different countries and healthcare settings, making them not easily comparable. Moreover, different levels of care are found within the same hospital settings. We excluded systematic reviews, entirely qualitative studies, grey literature (e.g. conference abstracts), studies only reporting protocols or abstracts and full reports not in English.

Two authors (SS, IA) independently double-screened titles and abstracts, reviewed full texts for eligibility and resolved any discrepancies with a third reviewer (JW). We extracted data using a pilot-tested, standardised data extraction form including study characteristics, interventions, outcomes and parent input into interventions' design. We assessed methodological quality with the Cochrane Collaboration risk of bias tool[12] for RCT and the ROBINS-I tool[13] for non-RCT.

We presented individual study aggregate data in a narrative synthesis, grouped studies into themes using a Grounded Theory Approach[14] and planned meta-analysis where data were appropriate for quantitative synthesis.

Patient involvement

This review was conceived in response to the clinical need identified by parents with neonatal care experience; a partnership including families with experience of preterm birth identified "what emotional and practical support improves attachment and bonding, and does the provision of such support improve outcomes for premature

https://mc.manuscriptcentral.com/bmjpo

babies and their families?" as a top 10 research priority[15]. Additionally, this review was conceived as part of planning a wider project to pilot a neonatal intervention, with parents' full input. Patients were not directly involved in the design, conduct, reporting or dissemination plans of our research.

RESULTS

We identified 8362 studies for screening and assessed 73 full text articles for eligibility (Figure 1). A total of 32 studies describing interventions that measured parent satisfaction in neonatal care as an outcome met the inclusion criteria, reporting data from over 2866 parents, 1 study did not report number of parents. Our analysis included 10 RCT and 22 non-RCT: 3 cohort trials, 18 unspecified designs and 1 implementation project (Tables 1-3). We further classified the unspecified non-RCT into 2 types, depending on how they defined their control groups and how they evaluated parent satisfaction (Table 3).

1. *"Unit- level effect":* Studies that assessed parent satisfaction during a period of routine care (control group) and introduced the intervention at a later time, with a different group of parents. In these studies improvement in parent satisfaction was evaluated between different parent groups, on a *unit level.*
2. *"Group level effect":* Studies that formed intervention and control groups using convenience sampling during the same time period. Both groups (or sometimes only the intervention group) had satisfaction measured after the intervention period (post intervention testing). Baseline parent satisfaction was also measured in both groups (pre intervention testing) in some studies. Improvement in parent satisfaction was demonstrated either by comparing

Confidential: For Review Only

outcomes between intervention/control groups following the intervention, or in comparison with the pre-intervention data.

Parent participants included mothers (14 studies), mothers and fathers (10 studies) or were not specified (7 studies). One study defined parent participants as a dyad of the mother with her designated support person. Median parent sample size was 63, ranging 7-482. This was higher for RCT (108 studies) compared to non-RCT (61 studies).

Study participants included parents of babies across the full range of gestations (23-42 weeks). Overall, 24/32 (75%) of studies involved preterm infants, 5/32 (16%) term infants and 7 studies did not state the gestational age of infants involved. Most studies (19, 59%) involved only preterm infants (up to 37 weeks); only 1 study (3%) involved only term infants and 5 studies (16%) involved both preterm and term infants. Preterm infants were included in 44% of RCT, versus 63% of non-RCT.

Most studies were reported as conducted in level III neonatal units (17 studies), followed by level not stated (9 studies), level II-III (3 studies), level II (2 studies) and level I (1 study). Definitions of neonatal levels of care are not standardised but vary across different countries; none of the included studies have explicitly stated which definition applies to them.

Tables 1-3 show the key characteristics of included studies. They include a description of each study's parent and infant sample, study design and intervention,

Confidential: For Review Only

outcome measures (timing and methods), results, parent input into intervention design and study impact on parent satisfaction.

Table 1. Included Randomised Controlled Trials (RCT)

Randomised controlled trials (RCT) by publication year

Author (Date), Country	Parent Gender/ sample size	Infants Gestation age (GA) in weeks /NICU level	Study design	Intervention	Outcome measures	Timing of measurement	Method of measurement	Results	Parent co-design?	Improved parent satisfaction?
1. Northrup et al. (2016), USA	Mothers and fathers /116	<28 / level III	Randomised controlled trial	Intervention: **Free Parking (FP).**<br><br>Parents received 7 parking vouchers at a time (value: $10/each) and continued to receive vouchers until infant discharge. Each voucher allowed free entry and exit for 24hr.<br><br>Control: Parents received the standard care and did not receive vouchers.	***Parent satisfaction with NICU care***	After babies were discharged (once)<br><br>- During the first high-risk-infant clinic visit after discharge<br><br>No pre-intervention parent satisfaction data available for comparison.	*Satisfaction questionnaire*<br><br>Validation: No content validity or reliability testing reported.<br><br>11 questions<br><br>- Seven items were summed (score 7-35) to measure "Support" (e.g., information sharing).<br><br>- Three items measured "Emotional Connection" to the infant (score 3-15)<br><br>- One item assessed "family involvement in infant care" (responses: not enough-just right-too much).<br><br>Greater scores indicated higher perceived support, connection and satisfaction.	**The groups did not differ significantly with respect to satisfaction.**<br><br>**Interv Control p-value**<br>NICU support<br>**Mean (SD)** 30(2.7) 28.7(3.7) 0.07<br><br>Emotional connection<br>12.3(1.7) 12.3(1.7) 0.96<br><br>Family involvement<br>"Just right"<br>81.4% 85% 0.07	**No**	**2**
2. Abdel-Latif et al. (2015), Australia	Mothers and fathers /63	25-42 / level III	Cross-over Randomised Controlled Trial	Intervention: **Parental Presence at Clinical Bedside Rounds (PPCBR).**<br><br>Parents attended bedside clinical rounds. Parents had opportunity to ask questions about their baby's condition and management.<br><br>Control: Parents received the standard care with no parental presence at bedside clinical rounds.	***Parent satisfaction assessed by questions of 3 domains:***<br>*1. Knowledge and understanding*<br>*2. Communication and collaboration*<br>*3. Privacy and confidentiality*	During babies' admission (once)<br><br>- At the end of each study arm, separated by a washout period<br><br>- No pre-intervention parent satisfaction data available for comparison	*Satisfaction questionnaire*<br><br>The authors stated "the research team designed the questionnaire".<br><br>Validation: No content validity or reliability testing reported.<br><br>Number and format of questions: not stated	**PPCBR had significantly higher adjusted mean (95% CI) scores for some questions from domains 1 and 2.**<br><br>Domain 3 was comparable between the two study groups.<br><br>**Interv Control p-value**<br>Domain 1 question:<br>*"I have received adequate information about my baby's condition and management"*<br>**Mean** 4.321 3.947 0.03<br><br>Domain 2 questions:<br>*"In the last week I have been able to communicate effectively with my baby's healthcare team"*<br>**Mean** 4.407 4.250 0.05<br><br>*"In the last week I have collaborated with my baby's healthcare team in the planning of care for my baby"*<br>**Mean** 3.843 3.426 0.02<br><br>*"In the last week I have been able to ask the healthcare team questions about my baby's care"*<br>**Mean** 4.642 4.259 0.004	**No**	**1**
3. Bastani et al, (2015), Iran	Mothers /100	30-37 Mean (SD)<br><br>Control: 33.90 (2.33)<br><br>Interv: 34 (1.9)<br><br>/ level not stated	Randomised Controlled Trial (block randomisation)	Intervention: **Family-centered Care (FCC).**<br><br>Mothers allowed access to their baby at any time, participated in the care process and were provided with information about neonatal care.<br><br>Control: Mothers received the standard care where they were only allowed to be present at the time of the infant's entry to the neonatal care unit, and were only routinely informed.	***Maternal satisfaction* relating to three themes:**<br>1. Parental presence<br>2. Participation in neonatal care<br>3. Information about neonatal care	During babies' admission (twice)<br><br>- 24 hours after admission<br>- At the time of discharge	*Satisfaction questionnaire* (Validated)<br><br>A modified satisfaction questionnaire was used, based on a parental satisfaction instrument developed for measuring satisfaction in Paediatric intensive care Units (PICU).<br><br>18 questions<br><br>Graded 0 (very dissatisfied) to 4 (very satisfied).<br><br>The overall satisfaction rate was classified based on the mean scores (score<50%, between 75-50% and > 75%).	**In the FCC group, pre and post intervention difference in maternal satisfaction was statistically significant p<0.001**<br><br>**Interv Control p-value**<br>**Mean (SD)**<br><br>**At 24 hr**<br>22.36(8.90) 22.06(9.77) 0.87<br><br>**At discharge**<br>59.28(6.86) 30.18(14.09) <0.01	**Unclear**<br><br>Mothers determined the reliability of the satisfaction tool and approved the educational pamphlet. Authors did not report if mothers had direct input in the intervention design.	**1**
4. Clarke-Pounder et al. (2015), USA	Mothers and fathers /19 families	23-39 / level III	Randomised Controlled Trial	Intervention: **Sharing information obtained from parent interviews with the primary NICU provider.**<br><br>Parents interviewed using the *NICU- adapted Decision Making Tool (N-DMT).* Information obtained was placed in the electronic medical record (EMR) and shared with the primary neonatal provider via email. Daily rounds on all infants were audio-recorded for 3 days after enrollment to see if information from the N-DMT was incorporated into daily care planning.<br><br>Control: The content of a recent social work note was communicated with the primary provider via e-mail, creating an attentional control group.	***Parent satisfaction with care***	During babies' admission (once)<br><br>- 2 weeks after study entry<br><br>No pre-intervention parent satisfaction data available for comparison.	*Satisfaction questionnaire*<br><br>A *NICU- adapted Decision Making Tool (N-DMT)* – specific questionnaire was used.<br><br>Validation: Partially reported. Authors stated reliability testing took place; no information on content validity provided.<br><br>8 questions: e.g."My baby's doctors considered my goals and hopes for my baby during decision-making".<br><br>Likert scale (1 strongly agree-4 strongly disagree). Total N-DMT score range 8–32.	**There was no significant difference in satisfaction with care as measured by the N-DMT scale between the control group and intervention groups in a univariable model or multiple variable model controlling for gestational age.**<br><br>**Interv Control**<br>**Median** (range)<br>26(15–28) 28.8(19–32)<br><br>**No p-value reported**<br><br>There was, however, a pattern of decreased satisfaction with care among the intervention group compared to the control group across the N-DMT-specific survey questions, although the differences were not statistically significant.	**Yes**<br><br>Information obtained from parents using the N-DMT was placed in the electronic medical record (EMR) and shared with the primary NICU provider via email (forming the intervention)	**2**
5.Holditch-Davis et al. (2013), USA	Mothers /208	Preterm infants	Randomised controlled trial	Interventions: **1. Mothers were taught how to massage infants with auditory, tactile, visual,**	**1. *Parent (mother) satisfaction with the***	During admission period and post discharge	*Satisfaction questionnaire*<br><br>The questionnaire was designed by the study team.	**No significant differences occurred between the groups.**	**No**	**2**

Confidential: For Review Only

		Mean (SD)<br><br>Overall group 27.2 (3.0)<br><br>/ 4 centres, levels II-III	3 groups (2 intervention and 1 control)<br><br>Post-intervention testing only.	**and vestibular stimulation (ATVV intervention)**<br>**2. Kangaroo care**<br><br>Control: Attention control group. Mothers spent a similar amount of time with the study nurse discussing the equipment needed for preterm infant care at home. Study nurses provided education and support for all three groups. Mothers were not prevented from engaging in interventions of the other groups but did not receive formal education from the study nurse on the other interventions.	***intervention***<br><br>***2. Satisfaction with the helpfulness of the study nurse***<br><br>***3. Whether the mother would recommend the study to others and the degree of change in the mother as a person and as a mother as a result of being in the study.***	- At the time of discharge<br><br>- At 2 months corrected age<br><br>No pre-intervention parent satisfaction data available for comparison.	Validation: Partially reported. Authors stated reliability testing took place; no information on content validity provided.<br><br>26 questions: relating to three dimensions of satisfaction: efficacy, caring, and technical quality.<br><br>Likert (1 least satisfied-5, 5 most satisfied)	Mothers in all three groups were satisfied with the intervention (mean scores of 3.3 or higher on a 5-point scale) and the helpfulness of the nurse (mean scores of 4.6 or higher on a 5-point scale).		
6. Franck et al. (2011), UK	Mothers and fathers /169	Mean (SD)<br><br>Control: 31.94 (5.17)<br><br>Interv: 29.40 (3.17)<br><br>/4 centres, level III	Cluster Randomised Controlled Trial	Intervention: **Increasing parental involvement in infant pain management in the NICU.**<br><br>Parents received a booklet providing evidence-based information about pain and comforting infants in the NICU setting. Parents received 2 visits from a research nurse showing them how to apply the comforting techniques described in the booklet.<br><br>Control: As part of usual care, parents in both the intervention and control groups received a detailed booklet with generic information about NICU care. Parents in the control group also received 2 visits from a research nurse listening to what parents had to say about their NICU experience (attention placebo).	At baseline:<br><br>***1. Parent satisfaction with NICU care***<br><br>One week after the intervention:<br><br>***1. Satisfaction with information about pain control***<br><br>***2. Satisfied nurses make infant comfortable***<br><br>***3. Satisfied pain medicines help infant***	During babies' admission (twice)<br><br>-At baseline (within 3 to 7 days of admission)<br><br>- 1 week after the intervention	*Individual questions*<br><br>Validation: No content validity or reliability testing reported.<br><br>1. At baseline:<br><br>Parent satisfaction was measured by 1 question: *"Satisfaction with NICU care"* (1 very satisfied-6 very unsatisfied) as part of the baseline parent characteristics questionnaire.<br><br>2. One week after the intervention:<br><br>Three questions using the word "satisfied' were selected from the validated *Parent Attitudes About Infant Nociception (PAIN)* survey (Likert scale 1 very satisfied-6 very unsatisfied)	**At baseline: there was no significant difference in satisfaction between intervention and control group**<br><br>**Interv** **Control**<br>**Mean (SD)** 1.45(0.71) 1.51(0.76)<br><br>**p-value** missing<br><br>**1 week after the intervention: Intervention parents were more satisfied with the information about pain control received than control parents.**<br><br>**Interv** **Control**<br>**Mean (SD)** 2.10(0.97) 3.28(1.27)<br>**p-value** < 0.001	**Yes**<br><br>The booklet was reviewed by 12 parents of infants who had been cared for in NICUs in the United Kingdom.	**1**
7.Livingston et al. (2009), USA	Mothers /12	Mean (SD)<br><br>Control:	Randomised Controlled Trial	Intervention: **Touch and massage.**<br><br>Mothers attended a 1hr massage class taught by a	***1. Caregiver (mother) satisfaction with their infant's care***	During babies' admission (three times)<br><br>- At baseline	*Satisfaction questionnaire*<br><br>Two questionnaires were developed by the research team.	**It is unclear in the report if specific between-group comparisons and statistical analysis were conducted.**	**No**	**3**
		33.4 (6.4)<br><br>Interv: 38.5 (3.1)<br><br>/ level III		nurse CIMI (certified infant massage instructor) and were asked to participate in at least 3 bedside massage instruction sessions taught within the next week. Infants received massage for 7 consecutive days, from the mother or a CIMI. The touch procedure lasted 20 minutes.<br><br>Control: Infants received all usual hospital services including medical care, physical and occupational therapy services and developmentally supportive nursing care.	***2. Caregiver satisfaction with the neonatal unit and the massage therapist***	- Upon completing the 7-day massage program<br><br>- 1 month following intervention	Validation: No content validity or reliability testing reported.<br><br>-1st questionnaire (at baseline): a brief self-report questionnaire about caregiver satisfaction with their infant's care until that moment. No further details reported.<br><br>-2nd questionnaire (upon completing the 7-day massage program and 1 month following intervention): a 10-minute satisfaction questionnaire relating to infant's response and caregiver satisfaction with the neonatal unit and the massage therapist.<br><br>Number of questions: not stated.<br><br>Likert scale (1 very dissatisfied-4 very satisfied).<br><br>Sample statements: *'How satisfied do you feel giving massage to your infant?'; 'I feel that massage improved my infant's hospital stay.'*	At baseline and day 7:<br>All caregivers were highly satisfied with the medical treatment their infant received.<br><br>At day 7 and 1 month follow-up:<br>All caregivers participating in the massage group reported high levels of satisfaction regarding their relationship with their infant and the massage program's impact on that relationship.<br><br>Slight improvements in satisfaction regarding time the caregiver spent with the infant and involvement in the infant's care were observed between day 7 and the 1-month follow-up (no further information reported).		
8. Koh et al. (2007), Australia	Mothers /200	Not stated / not stated	Randomised, Controlled Trial	Intervention: **Provision of taped conversations with neonatologists to mothers.**<br><br>The initial conversation and subsequent conversations of significance with a neonatologist were taped and analysed (for both groups). Mothers received a tape of each conversation and a tape recorder.<br><br>Control: Usual care. Mothers were not given the tape or recorder.	***Satisfaction with conversations held with the neonatologist***<br><br>***Satisfaction with the tape***	During admission period and post discharge<br><br>- At 10 days<br><br>- At 4 months<br><br>- At 12 months<br><br>No pre-intervention parent satisfaction data available for comparison.	*Individual questions and a satisfaction scale*<br><br>Validation: No content validity or reliability testing reported.<br><br>Number of questions: not stated.<br><br>Likert scale (1-5 most satisfied)<br><br>Questions related to: Satisfaction with amount and quality of information presented, doctors' communication skills, patient's participation in the conversation.<br><br>A satisfaction scale was used to assess:<br>Satisfaction with the tape	No differences were found between the two groups in satisfaction with conversations.<br><br>**Mothers of babies with a poor outcome in the tape group were, however, significantly more satisfied with the conversations:**<br><br>**Interv** **Control**<br>**Mean (95%CI)** 115(104-123.2) 100.5(94.1-109.4)<br><br>**p-value** 0.0051<br><br>Most (71-92%) of the mothers given the tapes stated that they helped their understanding, reminded them of what had been said, and helped their family to understand and recall information.	**No**	**1**

Confidential: For Review Only

9. Mitchell-DiCenso et al. (1996), Canada	Mothers and fathers/ 482	Mean (SD)<br><br>Interv: 35.1 (4.5)<br><br>Control: 35 (4.3)<br><br>/ level III	Randomised, Controlled Trial	Intervention: **Clinical Nurse Specialist/ neonatal practitioner team (CNS/NP) care.**<br><br>Infants of intervention parents were assigned to be cared for by the Clinical nurse special/neonatal practitioner CNS/NP team during the day and by paediatric residents during the night.<br><br>Control: Paediatric residents cared for infants of control parents around the clock. Neonatologists supervised both teams.	***Parent satisfaction with care***	During admission period and post discharge (twice)<br><br>- On 5th day after admission (full survey)<br><br>- After discharge over the phone (only questions related to satisfaction with discharge process)<br><br>No pre-intervention parent satisfaction data available for comparison.	*Satisfaction questionnaire (Validated)*<br><br>The study team developed and used the validated *Neonatal Index of Parent Satisfaction (NIPS)* questionnaire.<br><br>Number of questions: not stated.<br><br>NIPS score range (27-189); higher scores indicating greater satisfaction with care.	**No statistically significant difference between groups.**<br><br>**Interv Control p-value**<br>**NIPS Mean** 140 139 0.67<br><br>**Difference in means** 1.0, CI (-3.6-5.6)	**No**	**2**
10. Broyles et al. (1992), USA	Mothers /25	Mean (SD)<br><br>Control: 34 (4)<br><br>Interv: 33.4 (4)<br><br>/ level III	Randomised Controlled Trial	Intervention: **Detailed consent.**<br><br>Mothers were given information about mechanical ventilation. Detailed risk/benefit disclosure was provided both verbally and in writing.<br><br>Control:<br>Mothers were given a brief verbal description about mechanical ventilation supplemented with detailed verbal and written disclosure if desired by them (flexible consent).	***Maternal satisfaction with the information provided about mechanical ventilation***	During babies' admission (once)<br><br>- 24-48 hours after the intervention<br><br>No pre-intervention parent satisfaction data available for comparison.	*An interview* evaluating maternal satisfaction with the information provided about mechanical ventilation.<br><br>Validation: A psychiatrist with a special interest in interviewing techniques was consulted in designing and standardising this assessment.<br><br>A research nurse conducted the interview, "checking" each mother against one option regarding:<br>- Amount of information: Right amount-Too much-Too little<br><br>- Information made coping: More Difficult-Easier-No effect-Uncertain.	This study is measuring and comparing satisfaction with two different interventions (detailed vs flexible consent process), neither of which formally represent the usual routine care for all babies (no control).<br><br>**Small numbers. No data indicating statistical analysis conducted or evidence of statistically significant results.**<br><br>**Detailed Flexible**<br>**Right amount of information** 75% mothers 100%<br><br>**Too little information** 25% mothers<br><br>**Made coping easier** 67% mothers 69%	**No**	**3**

Table 2. Included Prospective Cohort Studies

**Prospective cohort studies by publication year**										
Author (Date), Country	Parents' gender/ sample Size	Infant Gestation age (GA) in weeks /NICU level	Study design	Intervention	Outcome measures	Timing of measurement	Method of measurement	Results	Parent co-design?	Improved parent satisfaction?
1. De Bernardo et al (2017), Italy	Mothers and Fathers /96	Mean (SD)<br><br>Control: 34.2 (5.25)<br><br>Interv: 32.7 (5.25)<br><br>/ level III	Non-randomized, prospective cohort pilot study<br><br>*Unit level effect*:<br>Two different time periods	Intervention: **FCC (Family-Centered Care).**<br><br>Parents had access to NICU for 8 hours/day. The NICU was widened and paediatric nurses taught parents procedures/practices for 10 days. Parents could observe clinical bedside rounds, hold meetings with the physicians, use the rooms and kitchen.<br><br>Control: Parents were permitted to visit their baby in NICU for 1 hour a day.	***Parent satisfaction relating to 3 specific domains:***<br><br>1. Knowledge and Understanding<br><br>2. Communication and Collaboration<br><br>3. Privacy and confidentiality	During babies' admission (once)<br><br>- At discharge (pre-FCC cohort and post-FCC cohort)<br><br>No pre-intervention parent satisfaction data available for comparison (different parent groups pre and post intervention).	*Satisfaction questionnaire.*<br><br>Validation: The authors state the survey *"was designed and validated by Abdel-Latif et al"*. No content validity or reliability testing reported in the original paper.<br><br>9 questions<br><br>3 questions: Related to adequate and timely information about the baby's condition.<br><br>3 questions: Related to communication and collaboration with the healthcare team.<br><br>3 questions: Related to respect of patient privacy.<br><br>Likert (1 strongly disagree-5 strongly agree)	**7/9 individual statements in the parent satisfaction questionnaire scored higher in the FCC compared to the NFCC (statistically significant difference).**<br><br>Example statement:<br>*"I have received adequate information about my baby's condition and management."*<br><br>**Interv Control**<br>**Median** 5 (3.45-5) 4 (3-5)<br><br>**p-value** <0.05	**No**	**1**
2. Petteys et al. (2015), USA	Not stated/ 10 parents included in sample analysis	24-36+ / level III	A prospective cohort design.<br><br>A feasibility study.<br><br>*Group level effect:*<br>Intervention/ control groups<br><br>Post-intervention testing only	Intervention: **PC (Palliative care).**<br><br>PC nurses provided important continuity of care for NICU infants clinically requiring PC and at least weekly verbal support of parents. The PC service also coordinated family conferences, provided or requested orders to improve infant symptom management and comfort, and addressed parental coping and self-care.	***Overall satisfaction with care received***	During babies' admission (once)<br><br>- At discharge (or study closure for infants who remained hospitalised)<br><br>No pre-intervention parent satisfaction data available for comparison.	*Satisfaction questionnaire*<br><br>A researcher-created questionnaire based on extensive current literature review.<br><br>Validation: Partially reported. Authors stated content validity testing took place; no information on reliability testing provided.<br><br>1 question<br><br>Likert (1 extremely dissatisfied-4 to extremely satisfied).	**Parent satisfaction response numbers were small (n= 10), thus statistical comparison of parental satisfaction between cohorts was not possible.**<br><br>However, 100% of responding PC parents (n= 2) reported being "extremely satisfied" with care, whereas only 50% of responding usual care parents (n= 4) reported extreme satisfaction.	**No**	**3**

Confidential: For Review Only

				Control: Usual clinical care for infants not requiring PC.			Optional free text (description of specific experiences impacting satisfaction with care)			
3. Stevens et al. (2011), USA	Mothers /147. For the OPBY NICU, 58 surveys were returned. For the SFR NICU, 89 were returned	Mean (SD)<br><br>Control: 35 (4)<br><br>Interv: 34 (3)<br><br>/ level not stated	Cohort trial. This research was part of a large prospective evaluation.<br><br>*Unit level effect:*<br>Two different time periods	Intervention: **SFR (Single-family room) NICU for neonatal care.**<br><br>Parents could visit their baby, room-in, do kangaroo care and breastfeed at any time, in individual rooms (containing bed, desk, closet, telephone, chair, refrigerator for breast-milk storage).<br><br>Control: OPBY (Open-bay) NICU. The traditional open-bay NICU was typical of facilities built before 1980. All neonates, family members, staff, monitors, and equipment were visible for all neonates in each room. Portable partitions were placed around the incubator for breastfeeding and kangaroo care.	***Parent satisfaction with different elements of NICU:***<br>*- Delivery*<br>*- Environment*<br>*- Nurses*<br>*- Physicians*<br>*- Discharge*<br>*- Personal*<br>*- Overall Assessment*	After babies were discharged (once)<br><br>- Mailed within 60 days of discharge of parents' infants from the NICU<br><br>No pre-intervention parent satisfaction data available for comparison (different parent groups pre and post intervention).	*Satisfaction questionnaire*<br><br>A questionnaire from Press Ganey Associates was used. Also included were three questions added by the investigators.<br><br>Validation: Partially reported. The original questionnaire was validated questionnaire but no content validity or reliability testing was reported regarding the 3 questions added by the study team.<br><br>42 questions in total (7 categories):<br>Delivery, Environment, Nurses, Physicians, Discharge, Personal,<br>Overall Assessment.<br><br>Likert (1 very poor-5 very good).	**Statistically significant improvement was found for the survey categories of Environment, Overall and the Total survey.**<br><br>Estimated numbers from report's figures as numbers not provided):<br><br>**Median SFR OPBY p-value**<br>Environment 4.7 3.7 <0.001<br>Overall 5 4.8 0.018<br>Total 4.7 4.5 0.045<br><br>16 items composite score for family-centered care:<br>4.4 4.0 0.017	**Yes**<br><br>Former NICU parents were involved in all phases of planning for the new SFR NICU.	**1**

Table 3. Included "Other" non-Randomised Controlled Trials (non-RCT)

**"Other" Non-Randomised controlled trials (Non-RCT) by publication year**										
Author (Date), Country	Parents' gender/ sample Size	Infant Gestation age (GA) in weeks /NICU level	Study design	Intervention	Outcome measures	Timing of measurement	Method of measurement	Results	Parent co-design?	Improved parent satisfaction?
1. Kadivar et al. (2017), Iran	Mothers /68	<=30 – 36<br><br>/ level not stated	Non-randomised, Convenience sampling.<br><br>*Group level effect:*<br>Intervention/ control groups.<br><br>Pre and post-intervention testing.	Intervention: **Internet-based education.**<br><br>Mothers used an educational website set up by the research team (files and clips). Mothers could visit the website from 5:00-6:00 pm for 10 days. They were also allowed to use the website outside of the above hours and to report the duration of using the website to the researcher. Mothers had to use the website at least 3 times during 10 days, each time for at least 30 min.<br><br>Control: Mothers in the control group received the routine education provided in the NICU.	***Maternal satisfaction***	During babies' admission (twice)<br><br>- Day 1 of intervention<br><br>- Day 10 of intervention	*Satisfaction questionnaire (Validated)*<br><br>The *"What Being The Parent of a Baby is Like-Revised" Questionnaire (WBPL- Revised)* was used. The original English version by Pridham and Chang was translated to Persian.<br><br>11 questions<br><br>Total satisfaction score range (11–99)	There was a significant difference in the mean score of satisfaction between cases and controls while the mean score of satisfaction increased in both groups.<br><br>**Comparison of the mean score between the two groups showed that the level of satisfaction was significantly higher in the case group versus the control group.**<br><br>**Interv Control**<br>**before intervention**<br>**Mean (SD)** 81.62(13.50) 85.71(9.46)<br>**p-value** 0.993<br><br>**after intervention**<br>**Mean (SD)** 93.88 (5.38) 90.12 (7.78)<br><br>**p-value** 0.024	**No**	**1**

Confidential: For Review Only

2. Kadivar et al. (2017), Iran	Mothers /70	Mean (SD)<br><br>Control 31.6 (2.4)<br><br>Interv: 32.9 (3.1)<br><br>/ level not stated	Non-randomised, Convenience sampling.<br><br>*Unit level effect:* Two different time periods	Intervention: **Narrative writing.**<br><br>Mothers did narrative writing at least 3 times until the 10th day of admission.<br><br>Control: Mothers in the control group received the routine NICU treatment and care.	***Mothers' satisfaction with medical care provided by physicians, medical students, and nurses during neonatal admission to the NICU***	During babies' admission (twice)<br><br>- Day 3 of intervention<br><br>- Day 10 of intervention	*Satisfaction questionnaire (Validated)*<br><br>The *NIPS questionnaire* by Mitchell et al was used and translated to Persian.<br><br>24 questions (Likert scale)<br><br>Likert (1 always or not satisfied-7 never or completely satisfied). A higher score indicates more satisfaction.	**The satisfaction level of the mothers in the intervention group increased significantly during the study.**<br><br>The results of independent t test showed a significant difference in the satisfaction changes of the mothers on the 3rd and 10th day of NICU admission between intervention and control groups, indicating the effectiveness of narrative writing.<br><br>The results of paired t-test also showed a significant difference in the mean satisfaction level of the mothers between the 3rd and the 10th day in the intervention group.<br><br>**Interv** **Control**<br>**After intervention**<br><br>**Mean (SD)** 137 (15.2) 102.3 (25.6)<br><br>**p-value** 0.001	**No**	**1**
3. Garingo et al. (2016), USA	Not stated /9	23-39 / level III	Non-randomised, Convenience sampling.<br><br>*Group level effect:* Intervention/ control groups<br><br>Post-intervention group testing only	Intervention: **Tele-rounding.**<br><br>Infants of intervention parents were cared for by an OFFSN (off site neonatologist) who was present via a remote-controlled robot. The OFFSN assessed infants via the robot's integrated stethoscope, with assistance from the nursing staff. During routine hours the OFFSN was called to discuss any issues with the patient. Emergencies/out of hours were covered by an ONSN (on site neonatologist).<br><br>Control: Infants of control parents received ONSN care. The attending neonatologist made daily patient rounds with the NICU team. After patient rounds, the NICU staff, under the supervision of the attending neonatologist implemented the care plan.	***Satisfaction with telemedicine***	During babies' admission (once)<br><br>- At the time of discharge<br><br>No pre-intervention parent satisfaction data available for comparison.	*Satisfaction questionnaire*<br><br>Validation: No content validity or reliability testing reported.<br><br>Number of questions: not stated.<br><br>Likert (1 excellent-5 very poor).	**Only the intervention group was assessed and only post-intervention.**<br><br>The authors reported that the parents surveyed were *"satisfied with their experience. 100% responded that they felt comfortable talking to the OFFSN on the mobile robot and would allow their infant or themselves to be cared for by a physician via telemedicine in the future."*	**No**	**4**
4. Globus et al. (2016), Israel	Mothers and fathers /Total surveys returned: 178	~40% in each group <32 / level III	Non-randomised, Convenience sampling.<br><br>*Unit level effect:* Two different time periods	Intervention: **SMSi-Short Message Services Implementation.**<br><br>Parents were updated daily regarding the health status of their infant via SMS (short-message-services) from the Electronic Patient Record. All SMS messages were sent at 09:00am, including one-sentence sections with updated information (e.g. location of the infant's crib and current weight). Information regarding acute events/deterioration of the infant's medical condition was not included in the SMS, but was delivered personally to the parents in real time.<br><br>Control: Routine care pre-SMS implementation.	***1. Parent satisfaction related to parent communication with the medical staff***<br><br>***2. Overall parent satisfaction with treatment and staff attitudes throughout hospitalisation.***	During babies' admission (once)<br><br>- pre-SMS cohort and post-SMS cohort<br><br>No pre-intervention parent satisfaction data available for comparison (different parent groups pre and post intervention).	*Satisfaction questionnaire*<br><br>The *"Parents' attitudes regarding their experience during their infants' hospitalisation in the NICU"* questionnaire was used, as well as selected items from a literature review of similar questionnaires, including that by York Hospital and by Conner and Nelson.<br><br>Validation: No content validity or reliability testing reported.<br><br>Selected items related to four aspects of the NICU experience. 2 out of 4 directly assessed parent satisfaction:<br><br>1. Parental assessment of their communication with the medical staff.<br><br>Likert scale (1 do not agree at all-5 strongly agree)<br><br>2. Overall satisfaction with treatment and staff attitudes throughout hospitalisation.<br><br>Visual analog scale (scores range 0-10). Higher scores reflect greater satisfaction.	Overall, in both periods, parents expressed a high degree of satisfaction regarding the medical treatment, the information given and the communication with the medical staff. Overall satisfaction with treatment and with staff attitudes throughout hospitalisation was slightly greater in the post-SMS cohort but did not reach statistical significance.<br><br>**In the post-SMS cohort, a statistically significant improvement was noted regarding physician availability and patience, parental feelings of comfort in approaching the physicians and nurses, and regularly receiving information regarding the infants' medical status from the physicians.**<br><br>**Post SMS** **Pre SMS**<br>**Mean (SD)** 4.1 (1.0) 3.7 (1.3)<br>**p-value** 0.03<br>*Specific question: "I was pleased with the frequency with which I received information regarding my infant".*<br><br>Although improvement in all other categories was documented, it did not reach statistical significance.	**No**	**1**

Confidential: For Review Only

5.Kazemian et al. (2016), Iran	Mothers /220 newborns (assumed 220 mothers)	>37 / level not stated	Non-randomised, Convenience sampling.<br><br>*Group level effect:* Intervention/ control groups<br><br>Post-intervention testing only	Intervention: **Rooming-in care.**<br><br>Mothers and babies were admitted to a different atmosphere to the routine care. This facilitated the mothers and neonates with separate beds along with phototherapy devices and nursing clinical supervision.<br><br>Control: The routine care practiced in this neonatal unit supported partial stay of mothers beside their neonates, while sitting on chairs; however, most of the time the mother-infant dyad was separated.	***Maternal satisfaction with the neonatal care services and hospital stay comfort***	During babies' admission (once)<br><br>-Not stated exactly when<br><br>No pre-intervention parent satisfaction data available for comparison.	*Satisfaction questionnaire*<br><br>Validation: No content validity or reliability testing reported.<br><br>The authors state, *"a validated self-made questionnaire was employed, which was filled in by some trained midwives."* No further information on validation processes, number of questions or name of the questionnaire was provided.<br><br>Likert (5 very satisfied-1 dissatisfied).	**The level of satisfaction was significantly higher in the intervention group, compared to that in the control group.**<br><br>**Interv** **Control**<br>**Satisfaction %** 26.6 18.8<br><br>**p-value** 0.027	**No**	**1**
6. Van de Vijver and Evans (2015), UK	Not stated /105	Not stated / not stated	Non-randomised, Convenience sampling.<br><br>*Unit level effect:* Three different time periods	Intervention: **Baby diary.**<br><br>Each parent received a communication diary on their infant's admission to the unit. Staff wrote-in infant status updates and kept an infant interaction log with parents. Parents wrote in memories and questions for staff to address during face-to-face communication.<br><br>Control: Routine care, before implementation of the diaries.	***Satisfaction with communication from neonatal staff***	During babies' admission (three times)<br><br>- On the day of babies' discharge at study baseline<br><br>- On the day of babies' discharge at 1 month<br><br>On the day of babies' discharge at 15 months	*Satisfaction questionnaire*<br><br>The study team designed a questionnaire, based on the Department of Health and the National Institute for Health and Care Excellence (NICE) quality standards for specialist neonatal care.<br><br>Validation: No content validity or reliability testing reported.<br><br>5 questions ("yes or no")	**Small numbers. No data indicating statistical analysis conducted or evidence of statistically significant results.**<br><br>*"I was receiving regular communication from staff"*<br>94% - 1 month post diary cohort<br>93% - 15 months post diary cohort<br>77% - pre diary cohort<br><br>*"My questions and concerns were being addressed"*<br>100% - 1 month post diary cohort<br>93% - 15 months post diary cohort<br>91% - pre diary cohort<br><br>"I feel more involved in my baby's care"<br>92% - 1 month post diary cohort<br>100% - 15 months post diary cohort<br>88% - pre diary cohort	**Yes.**<br><br>The intervention concept was created by the project leaders following analysis of baseline survey results and used after multi-disciplinary input and discussion with staff and parents.	**3**
7. Voos and Park. (2014), USA	Not stated / 62	Not stated / level III	Non-randomised, Convenience sampling.<br><br>*Unit level effect:* Two different time periods	Intervention: **OU (Open Unit) policy.**<br><br>Parents were allowed access to their baby 24 hours a day, 7 days a week.<br><br>Control: Parents pre-OU implementation received routine care. The unit was closed to parents during nurse change of shift in mornings and evenings.	***Parent satisfaction with how much time parents get to spend with their baby***	After babies were discharged (once)<br><br>- After pre-OU parents were discharged<br><br>- After post-OU parents were discharged	*Single question (From a validated questionnaire)*<br><br>The question *"Did you get to spend as much time as you wanted with your baby?"* was used from the NRC (National Research Corporation) Picker parent survey.<br><br>1 question ("yes or no")	**Small numbers. No data indicating statistical analysis conducted or evidence of statistically significant results.**<br><br>*"Did you get to spend as much time as you wanted with your baby?" Yes.*<br><br>Pre OU 78% (18/23)<br>Post OU 92% (36/39)	**Yes.**<br><br>The NICU has a Family-centered care committee including parents, which conducted this project.	**3**
8. Segre et al. (2013), USA	Mothers /23	Mean (SD) 31.57 (5.30) / level III	For the outcome of parent satisfaction:<br><br>Non-Randomised, Convenience sampling.<br><br>*Group level effect:* Intervention/ control groups<br><br>Post-intervention group testing only	Intervention: **(LV) Listening visits.**<br><br>Mothers met with the LV provider for up to six 50-min LV sessions, conducted in a private hospital, every 2–3 days, within 1-month. Visits entailed greeting, debriefing, updating on current issues, working an agenda through listening and problem solving, and providing closure through summary.<br><br>Control: Women who did not meet the specific criteria (e.g. minimum score on depression scale) were not invited to join the treatment trial and received routine NICU care/support instead.	***Satisfaction with the treatment and the outcome.***	During babies' admission (once)<br><br>- Not stated exactly when<br><br>No pre-intervention parent satisfaction data available for comparison.	*Satisfaction questionnaire*<br><br>The *Client Satisfaction Questionnaire* was used.<br><br>Validation: Partially reported. Authors stated reliability testing took place; no information on content validity provided.<br><br>8 questions.<br><br>Format of questions: not stated	**Only the intervention group was assessed and only post-intervention.**<br><br>The authors reported:<br><br>*"The majority of women who received LVs were highly satisfied with the intervention".*<br><br>*"The average score for the Client Satisfaction Questionnaire was 29.91, comparable to levels of satisfaction reported by clients receiving depression treatment from a mental health professional."*<br><br>*"91.3% of our participants rated the quality of help they received as excellent."*	**No**	**4**
9. Palma et al. (2012), USA	Not stated / 26 families returned the survey containing the satisf. measure)	Not stated / level II	Non-randomised, Convenience sampling.<br><br>*Unit level effect:* Two different time periods	Intervention: **YBDU (Your Baby's Daily Update). A daily parent update letter generated from the Electronic Medical Record (EMR).**<br><br>Parents were given daily YBDU reports, printed automatically from the EMR. The YBDU included information about an infant's status during the past 24 hours and a hand-written update by the infant's care provider.<br><br>Control: Parents received routine care and usual verbal updates (6 months pre- adoption of YBDU).	***Satisfaction with YBDU***	During babies' admission (once)<br><br>- Not stated exactly when<br><br>No pre-intervention parent satisfaction data available for comparison (different parent groups pre and post intervention).	*Satisfaction questionnaire*<br><br>A questionnaire including items regarding adoption of and satisfaction with YBDU was used.<br><br>Validation: No content validity or reliability testing reported.<br><br>Number and format of questions: not stated.	**Only the intervention group was assessed and only post-intervention.**<br><br>The authors reported:<br>*"When asked to rate the statement "I like receiving Your Baby's Daily Update", 96% of families who used YBDU as an information source responded with the highest rating, "always"."*	**No**	**4**
10. Voos et al.	Not stated /28	Not stated / level not	Non-randomised,	Intervention: **Family-centered rounds**	***Global satisfaction with***	During babies' admission (twice)	*Satisfaction questionnaire (Validated)*	**A subset of NIPS items related to communication**	**No**	**1**

Confidential: For Review Only

(2011), USA		stated	Convenience sampling.<br><br>*Unit level effect:*<br>Two different time periods	**(FCRs).**<br><br>Parents were invited to attend rounds and choose their level of involvement (attend every day/not at all/periodically). For confidentiality concerns, parents were asked to step outside while rounds of others' infants took place. The staff augmented FCRs by meeting with parents again after rounds if needed.<br><br>Control: Parents received routine care. Prior to FCR implementation parents were asked to leave the unit during rounds.	***the NICU experience***	- Prior to FCR<br><br>- 6 months after starting FCR	*The NIPS* questionnaire.<br><br>24 questions: looking at satisfaction in different areas of the NICU (medical caregivers, communication, tests, and procedures).<br><br>Likert scale (1-7 points).	**(i.e. being kept informed as to changes in the infant's condition, meeting with physicians, and information about long-term expectations) yielded a significant increase from pre to post FCR scores.**<br><br>**post-FCR pre-FCR p-value**<br>**NIPS score** 5.5 4.4 <0.01<br><br>The average score on the NIPS did not change significantly.		
11. Weiss et al. (2010), USA	Mothers /84	Mean (SD)<br><br>Pre-interv group: 32 (4.4)<br><br>Post-interv group: 32 (9)<br><br>/ level III	Non-randomised, Convenience sampling<br><br>*Unit level effect:*<br>Two different time periods	Intervention:<br>**An intervention to increase PMP (Principal Medical Providers) availability and communication frequency.**<br><br>(1) A brief education module for PMPs was introduced (2) parents received a contact card with PMP names, job descriptions and contact information (3) a poster of the faces, names and titles of the PMPs was placed at NICU entrance.<br><br>Control: Parents received routine care in the pre-intervention cohort, without the above.	***Parent satisfaction with physician and nurse practitioner communication***	During babies' admission (twice)<br><br>- Pre-intervention<br><br>- Post-intervention	*Satisfaction Questionnaire (Validated)*<br><br>A pilot survey written by Press Ganey and the Picker Institute was used and revised based on parent responses.<br><br>6 open-ended questions (Quantity of communication)<br><br>6 Likert scale questions (range questions (Availability, understanding, reciprocity, empathy, overall satisfaction)	**Overall satisfaction, based on the ordinal analysis of the five-point Likert scale, was significantly higher after the intervention (P<0.01).**<br><br>**Overall satisfaction, dichotomised into a satisfied subgroup and a dissatisfied subgroup for each cohort, was also significantly increased after the intervention.**<br><br>**post –interv pre-interv**<br>**Very satisfied/ Somewhat satisfied** 97%(32/33) 74%(37/50)<br><br>**p-value** <0.01	**No**<br><br>Authors stated that only after trialing the intervention on many parents (both satisfied and unsatisfied) gave suggestions to improve it.	**1**
12. Foster et al. (2008), Australia	Mothers and fathers /93<br><br>5 Special Care Nurseries	Mean (SD)<br><br>Headbox: 36.5 (2.6)<br><br>CPAP: 36 (3)<br><br>/level I	Non-randomised, Convenience sampling<br><br>*Group level effect:*<br>Intervention 1/ intervention 2 groups	Intervention 1: **Infants received headbox oxygen treatment for respiratory distress.**<br><br>Intervention 2: **Infants received continuous oxygen positive airway pressure (CPAP) treatment for respiratory distress.**	***Satisfaction with treatment (i.e. headbox oxygen or CPAP)***	During babies' admission (once)<br><br>- Within 5 days of the babies' admission<br><br>No pre-intervention parent satisfaction data available for comparison.	*Single question*<br><br>Validation: No content validity or reliability testing reported.<br><br>1 likert scale question (1 not at all satisfied-5 extremely satisfied).	**Parents with babies receiving CPAP rated their satisfaction with the baby's treatment statistically significantly higher than the headbox group mean rating.**<br>**Headbox CPAP**<br>**Mean (SD)** 3.71 (1.31) 4.51 (0.79)<br>**p-value** 0.001	**No**	**1**
			Post intervention testing only					The CPAP group averaged between *very and extremely satisfied* compared with parents of babies receiving headbox, who averaged between *satisfied and very satisfied ratings.*		
13. Byers et al. (2006), USA	Only mothers reported /35	Preterm infants<br><br>Mean (SD)<br><br>Control: 28.9 (3.44)<br><br>Interv: 28.6 (3.37)<br><br>/ level II/III	For the outcome of parent satisfaction:<br><br>Non-randomised, Convenience sampling<br><br>*Group level effect:*<br>Intervention/ control groups<br><br>Post-intervention testing only	Intervention: **Infants received individualised, developmentally supportive family-centered care.**<br><br>Infants received care within the framework and philosophy of individualised, developmentally supportive family-centered interventions.<br><br>Control: Infants received the traditional NICU standard of care.	***Parent satisfaction relating to:***<br>*- parental perceptions of staff caring*<br>*- education received*<br>*- preparation for the parental role*<br>*- overall satisfaction with the NICU experience*	During babies' admission (once)<br><br>- On the day before discharge<br><br>No pre-intervention parent satisfaction data available for comparison.	*Satisfaction questionnaire*<br><br>The *NICU's parental satisfaction tool* was used.<br><br>Validation: Partially reported. Authors stated content validity testing took place, but "because of the disparate nature of the items, survey reliability was not assessed".<br><br>11 questions<br><br>Likert scale (1-5 strongly agree)	**Independent t-test analysis of parent satisfaction/perception scores showed no significant difference between groups.**<br><br>Example statement: "*I was satisfied with the car my baby and I received in the NICU*"<br><br>**Interv Control**<br>**Mean (SD)** 4.94(0.23) 4.71(0.47)<br>**p-value** 0.064<br><br>Both groups reported very high satisfaction with their NICU experience (4.4-5.0)	**No**	**2**
14. Mills et al. (2006), USA	Not stated/ not stated<br><br>Parents of infants from 6 hospitals	Not stated / level not stated	Implementation project<br><br>Plan Do Study Act (PDSA) quality improvement testing	Intervention: **5 potentially better practices (PBPs) in the area of discharge planning.**<br><br>The project team iteratively implemented 5 PBPs:<br>1. Created an easy-to-use, easy-to-access discharge planning tool kit.<br>2. Restructured communication tools and processes to reflect a "plan for the day, the stay, and the way" to discharge.<br>3. Maximised the impact and use of caregiver educational tools, and updated materials and delivery systems for caregiver education.<br>4. Used various continuous quality improvement tools and processes to ensure parent/caregiver and staff satisfaction.<br>5. Analysed and enhanced interactions with and transfers into	***General satisfaction***<br>*- with care*<br>*- parents' feelings about preparedness for discharge*<br>*- ability and confidence in feeding*<br>*- familiarity with their infant*<br>*- feeling like a parent*<br>*- participation in care*<br>*- adequacy of information from staff about medical and care issues*	During babies' admission (4 times)<br><br>- Not reported exactly when	*Satisfaction questionnaire*<br><br>The Internet-based parent satisfaction survey *"howsyourbaby.com"* that was developed especially for this NICU population was used.<br><br>Validation: No content validity or reliability testing reported.<br><br>Number and format of questions: not stated.	Through multiple rapid-cycle projects, the project's collaborative group made changes within the 5 PBP plans.<br><br>**Parent satisfaction measures were used to longitudinally monitor the changes made, rather than make direct group comparison. No data indicating statistical analysis conducted or evidence of statistically significant results.**<br><br>Parent satisfaction survey results (all centers combined) were high across 4 measurement quartiles. No specific interquartile analysis was reported.<br><br>Parent readiness for discharge was high at the beginning and throughout the collaborative. Parents' receiving *"just the right amount of information"* regarding car seat trials and safe sleep demonstrated some variability throughout the collaborative.	**No**	**3**

Confidential: For Review Only

				the community.<br><br><u>Control:</u> N/A. No discrete control group. PDSA quality improvement methodology was applied to parent participants.					
15. Wielenga et al. (2006), The Netherlands	Mothers and fathers / 46	Mean (SD)<br><br>Control: 28.5 (26.0–29.9)<br><br>Interv: 28.3 (25.6-29.9)<br><br>/ level III	Non-randomised, Convenience sampling<br><br>*Unit level effect:* Two different time periods	<u>Intervention:</u> **The Newborn Individualised Developmental Care and Assessment Program (NIDCAP).**<br><br>Infants received care according to NIDCAP principles and parents were taught how to provide it. Caregiving plans were designed based on the infant's current developmental stage, medical condition and family needs. Caregivers learnt to watch sensitively and note the infant's reactions to different types of handling and care, making continuous adjustments.<br><br><u>Control:</u> Infants received traditional neonatal care practiced at that time.	***Parent satisfaction relating to:***<br>*-Overall rating*<br>*-Care of the baby*<br>*-Communication with staff*<br>*-Involvement in care -Being prepared - Support*<br>*-Being a parent*<br>*-Being near your baby -Total score*	After babies were discharged (on day of discharge/ transfer)<br><br>- Pre NIDCAP cohort<br><br>- Post NIDCAP cohort	*Satisfaction questionnaire (Validated)*<br><br>The *NICU-PSF* was used and translated from English to Dutch.<br><br>62 questions<br><br>Closed and open-ended questions.<br><br>Different rating scales used (5-point rating scale from "extremely satisfied" to "not at all satisfied" or "excellent" to "poor").<br><br>Total score range (50-243 points)	**The intervention group's mean total score was significantly higher than the control.**<br><br>**Interv Control**<br>**Mean (SD)** 185.67(17.74) 174.04(20.98)<br><br>**p-value** 0.041<br><br>Almost all separate concepts showed an increase in their mean scores. The concept of "*being a parent"* had a slightly lower mean score (9.39, SD = 1.73) in the intervention group than in the control group (9.78, SD = 2.09).<br><br>**The concept of *"preparedness"* showed statistically significant difference:**<br><br>**Interv Control**<br>**Mean** 16.38 13.83<br>**p-value** 0.038	**No**	**1**
16. Penticuff and Arheart. (2005), USA	Dyads (both parents or mother with her support person)/ 122 mothers<br><br>Results based only on mothers' data.	Not stated / Level III	A repeated measures design<br><br>- First 2 years (control group data collection)<br><br>- Year 3 (staff training)<br><br>- Year 4 (implementing the intervention)<br><br>- Year 5 (collecting data from the intervention group)<br><br>*Unit level*	<u>Intervention:</u> **The Newborn Individualised IPC- CPM intervention (Infant Progress Chart) - (Care Planning Meetings).**<br><br>Both the mother and father (or the mother and her designated support person) were shown how to use the Infant Progress Chart and attended 3 Care Planning Meetings (with neonatologists/Neonatal Nurse Practitioners).<br><br><u>Control:</u> During the control phase, professionals carried out usual communication and interaction with control	***Satisfaction with participation in decision making* was measured by 5 collaboration indices:**<br><br>Satisfaction with<br>(1) Care<br>(2) Relationships with professionals<br>(3) Decision input<br>(4) The process of decision making<br>(5) Decisions made	During babies' admission (three times)<br><br>- Within 0–3 days<br><br>- 9– 12 days<br><br>- 25–28 days of an infant's admission to the NICU	*Three satisfaction questionnaires*<br><br>1. Two subscales of the investigator-designed *"Parents' Understanding of Infant Care and Outcomes Questionnaire"* were used to measure Satisfaction with Care (1).<br><br><u>Validation:</u> Partially reported. Authors stated content validity testing took place; no information on reliability testing provided.<br><br>30 questions.<br><br>Five-point Likert scale.<br><br>2. A subscale of the investigator-designed	**The intervention group was more satisfied with the amount of decision input they had (3) and with the process by which medical decisions were made (4).**<br><br>**Interv Control p-value**<br>Decision input amount (3)<br>**Mean** 33.44 30.05 0.058<br><br>Process of decision making (4)<br>**Mean** 120.20 104.95 0.012<br><br>There were no statistically significant differences between control and intervention groups in satisfaction with their infants' care (1), with relationships with NICU professionals (2) and with the decisions made for infant treatment (5).	**No**	1
			effect. Two different time periods	group parents.			*"Relationships with Professional and Decision Input Questionnaire"* was used to measure Satisfaction with relationships (2).<br><br><u>Validation:</u> Partially reported. Authors stated content validity testing took place; no information on reliability testing provided.<br>12 questions.<br><br>Five-point Likert scale<br><br>3<u>. Validated.</u><br>The *"Collaboration and Satisfaction About Care Questionnaire"* developed by Baggs, was used to measure Satisfaction with decision input (3), with decision process (4) and with decisions made (5).<br><br>9 questions.<br><br>7-point scale, (1 strongly disagree -7 strongly agree)			
17. Byers et al. (2003), USA	Mothers/ 19	Mean (SD)<br><br>Control: 29 (2.00)<br><br>Interv: 28.9 (2.42)<br><br>/ level II-III	For the outcome of parent satisfaction:<br><br>Non-randomised, Convenience sampling<br><br>*Group level effect:* Intervention/ control groups<br><br>Pre and post-intervention testing	<u>Intervention:</u> **Co-bedding premature multiple-gestation infants in incubators.**<br><br>Infants were nursed in the same incubator using a co-bedding protocol (e.g. recording all of the care provided to one infant before providing care to the second infant)<br><br><u>Control:</u> Single-bedding premature multiple-gestation infants in incubators.	***Parent satisfaction related to:***<br>*- staff concern*<br>*- support of family*<br>*- staff explanations*<br>*- infant environment,*<br>*- comfort with feeding*<br>*- kangaroo care encouragement*<br>*- staff explanation of signs of infant stress*<br>*- visiting schedule*<br>*- overall satisfaction with the NICU experience*	During babies' admission (twice)<br><br>- At baseline<br><br>- 5 days later	*Satisfaction questionnaire*<br><br>The NICU's standard parental satisfaction tool was used.<br><br><u>Validation:</u> Partially reported. Authors stated content validity testing took place, but because of the disparate nature of the items, survey reliability could not be assessed.<br><br>11 questions.<br><br>5-point Likert-type scale.	**The only significant difference for a post-intervention item was a higher score for the item "Attempts were made to create a quiet environment for my baby."**<br><br>**Interv Control p-value**<br>**Mean** 4.80 3.89 0.033<br><br>Independent t-tests comparing the co-bedded and control group parental scores found no significant differences in their parental satisfaction scores, except for higher baseline parental satisfaction scores (p=0.029) in the co-bedded group.	**No**	**1**

Confidential: For Review Only

18. Polizzi et al. (2003), USA	Mothers and fathers/ 33	Mean (SD)<br><br>Control: 32.97 (1.9)<br><br>Interv: 33.08 (1.31)<br><br>/ level III	A retrospective, comparative, descriptive design.<br><br>*Unit level effect*	Intervention: **Co-bedding multiple-gestation infants in the NICU.**<br><br>Multiple-gestation infants were nursed in the same incubator or crib. The intervention was evaluated retrospectively after implementation of a co-bedding practice protocol.<br><br>Control: Traditionally-bedded group (babies were routinely placed in separate incubators or cribs)	***Parental satisfaction as measured by 9 questions relating to parent perceptions and their baby's care***	After babies were discharged (once)<br><br>- All parents were mailed the survey. A second survey was sent to those who did not respond after 2 months<br><br>No pre-intervention parent satisfaction data available for comparison.	*Satisfaction questionnaire*<br><br>The *parental perception/ satisfaction tool* was used.<br><br>Validation: Partially reported. Authors stated content validity testing took place; no information on reliability testing provided. 6/9 questions were from a similar tool that was validated by the Vermont Oxford NICU Quality Improvement Initiative.<br><br>9 questions (such as *"I was satisfied with the care my babies received in the hospital").*<br><br>Likert (1 strongly disagree- 5 strongly agree)	Mothers reported overall satisfaction with the NICU care and staff, as well as adequacy of their ability to care for their infants after discharge, with scores ranging from 4.19 to 4.71.<br><br>**The only survey item score that was significantly different between groups was for the item *"I was encouraged by the hospital staff to bond with my babies."***<br><br>**Interv Control p-value**<br>**Mean** 4.71 4.36 0.049	**No**	**1**
19. Legault and Goulet. (1995), Canada	Mothers/ 61 completed both tests	Mean (range)<br><br>30 (24-35)<br><br>/ level II	Time-series design<br><br>*Group level effect:* Same group exposed to both methods with post-method testing only.	Intervention: **Kangaroo method of removing an infant from an incubator.**<br><br>Mothers were taught the "kangaroo method" (skin-to-skin contact): infant wears a diaper/head cap and is placed in a vertical position on the parent's bared chest. A blanket covers the infant and the parent's clothing is fastened around the infant. The parent sits in a rocking chair, inclined so that the infant's head is at 60'.<br><br>Control: Traditional method. Newborns wearing a diaper and a head cap, are wrapped in a blanket and placed in their parent's arms.	***Mothers' satisfaction with:***<br>*- Each method of removing an infant from incubator*<br>*- Her feelings after each method*	During babies' admission (twice)<br><br>- After the intervention<br><br>- After the control method<br><br>No pre-intervention parent satisfaction data available for comparison.	*Satisfaction questionnaire*<br><br>The *"Maternal Satisfaction Questionnaire"* was used. It was developed by integrating components described by Affonso et al and the clinical experience of the investigators.<br><br>Validation: Partially reported. Authors stated content validity testing took place; no information on reliability testing provided.<br><br>15 questions<br><br>Likert (1 very much-5 don't know)<br><br>An open-ended question invited the mother to list and explain anything else related to her experience.	**Regardless of the method tested, mothers expressed high levels of satisfaction (it was the first time since giving birth that they could hold their infants).**<br><br>Three statements proved more powerful in discriminating between the methods:<br><br>**Rated higher after the kangaroo method test:**<br>- *"I like the contact with my baby's skin"*<br>(p=0.0001)<br><br>**Rated higher after the traditional method test:**<br>- *"I like to talk to and whisper to my baby" (p = 0.015)*<br>- *"I looked into my baby's eyes and stared at his/her face" (p=0.0001)*	**No**	**1**

Legend for Tables 1-3: *Number in last column illustrates each intervention's reported effect on parent satisfaction: 1. Parent satisfaction was statistically significantly higher in the intervention group; 2. Parent satisfaction was not reported to be statistically significantly different in the intervention group; 3. Unclear if parent satisfaction improved (small study numbers and/or no statistical analysis performed); 4. Only the intervention group was assessed*

Parent satisfaction

Outcome measures: All 32 studies reported they measured parent satisfaction as an *a priori* outcome. Only one study confirmed this through a protocol. Overall 18/32 (56%) of studies (4/10, 40% RCT and 14/22, 64% non-RCT) reported a higher level of parent satisfaction associated with the intervention studied. Multiple different outcome measures within the domain of parent satisfaction were used; we grouped these into 4 categories: i) Parent satisfaction (no additional description); ii) Parent satisfaction with NICU care; iii) Parent satisfaction related to specific components such as communication, staff or information; iv) Parent satisfaction with a specific intervention.

Confidential: For Review Only

Timing of measurement: Parent satisfaction was mostly measured *'during infant admission only'* (24 studies; between 1-4 times), followed by *'after infant discharge only'* (5 studies; 1 time) and '*both during admission and after discharge'* (3 studies; between 1-3 times). In the majority of studies (19/32, 59%) no pre-intervention parent satisfaction measurements were conducted in the same parent groups with available post-intervention data (ie paired parent data for satisfaction levels did not exist). Instead, impact of interventions was determined comparing intervention/control group measurements in different time periods (Tables 1-3).

Method of measurement: Parent satisfaction was assessed using 32 different methods: 29 different questionnaires, 2 different single questions, and by structured interview in 1 study; in total 334 different questions were used to assess parent satisfaction. Only 6/29 (21%) of questionnaires were reported to be fully validated (both content validation and reliability testing); 23/29 (79%) questionnaires were partially or completely unvalidated. The most commonly used questionnaire was the validated *Neonatal Index of Parent Satisfaction (NIPS)*[16] questionnaire (3 studies).

Interventions and impact on parent satisfaction

We grouped included studies into 5 intervention themes: parent involvement (14 studies); information provision/communication (8 studies); clinical care (7 studies); parent emotional support (2 studies); other (1 study). Parent involvement interventions were more commonly assessed in RCT compared to non-RCT . We categorised interventions *as effective* or *not effective* based upon whether a statistically significant difference between intervention and control groups was

Confidential: For Review Only

reported for parent satisfaction (Tables 4,5). None of the studies reported statistically significantly lower parent satisfaction in the intervention group compared to the control group. We classified studies as *unclear if effective* if they included small sample numbers or if statistical analysis was not performed (Table 6). Finally, we highlighted studies where *only the intervention group was assessed and only post-intervention,* where comparison to a control group was not possible (Table 7).

Overall, 18/32 studies (56%) reported higher parent satisfaction in the intervention group; 4/10 RCT and 14/22 non-RCT. The intervention theme where higher satisfaction was most consistently reported was parent involvement (10/14 studies). Due to the large heterogeneity of outcome measure scales a quantitative synthesis and meta-analysis was not possible.

Table 4. "*Effective*" interventions in themes

**Theme: Parent involvement**
More NICU access, parents on WRs, Education (De Bernardo et al, Italy, 2017)
Rooming-in care (Kazemian et al, Iran, 2016)
Parental Presence at Clinical Bedside Rounds (Abdel-Latif et al, Australia, 2015) **RCT**
More NICU access, care involvement, education (Bastani et al, Iran, 2015) **RCT**
Education re: pain management (Franck et al, UK, 2011) **RCT**
Single-family NICU rooms (Stevens et al, USA, 2011)
Family-centered rounds (Voos et al, USA, 2011)
Newborn Individualised Developmental Care and Assessment Program (NIDCAP) [illegible]herlands, 2006)
Infant Progress Charts filled by parents and 3 Care Planning Meetings (Penticuff and Arheart. USA, 2005)
Kangaroo care (Legault and Goulet, Canada, 1995)
**Theme: Information provision / communication**

Confidential: For Review Only

Internet-based education (Kadivar et al, Iran, 2017)
Daily SMS from Electronic Patient Record (Globus et al, Israel, 2016)
Staff education, staff contact card given to parents, staff poster at NICU reception [illegible]010)
Provision of taped conversations with neonatologists to mothers (Koh et al, Australia, 2007) **RCT**
**Theme: Clinical care**
a. Headbox oxygen for respiratory distress<br>b. CPAP for respiratory distress (Foster et al, Australia, 2008)
Co-bedding infants in incubators (prospective) (Byers et al, USA, 2003)
Co-bedding infants in incubators (retrospective) (Polizzi et al, USA, 2003)
**Theme: Parent emotional support**
Narrative writing (Kadivar et al, Iran, 2017)

Legend: *Interventions where parent satisfaction was reported to be statistically significantly higher in the intervention group.* ***RCT:*** *Randomised Controlled Trial*

Table 5. "*Ineffective*" interventions in themes

**Theme: Parent involvement**
a. Massage with auditory, tactile, visual, and vestibular stimulation<br>b. Kangaroo care (Holditch-Davis et al, USA, 2013) **RCT**
Individualised, developmentally supportive family-centered care interventions (Byers et al, USA, 2006)
**Theme: Information provision / communication**
Sharing information obtained from parent interviews with the primary NICU provider (Clarke-Pounder et al, USA, 2015) **RCT**
**Theme: Clinical care**
Clinical Nurse Specialist/ neonatal practitioner team care (Mitchell-DiCenso et al, Canada, 1996) **RCT**
**Theme: Other**
Free Parking (Northrup et al, USA, 2016) **RCT**

Legend: *Interventions where parent satisfaction was not reported to be statistically significantly different in the intervention group;* ***RCT:*** *Randomised Controlled Trial*

https://mc.manuscriptcentral.com/bmjpo

Table 6. *"Unclear if effective"* interventions in themes

**Theme: Parent involvement**
Open Unit policy: 24/7 NICU access (Voos and Park, USA, 2014)
Touch and massage for 7 days (Livingston et al, USA, 2009) **RCT**
**Theme: Information provision / communication**
Clinical staff enter updates in baby diary (Van de Vijver and Evans, UK, 2015)
Detailed information provided during consenting (Broyles et al, USA, 1992) **RCT**
**Theme: Clinical care**
Palliative care (Petteys et al, USA, 2015)
Five potentially better practices in the area of discharge planning (Mills et al, USA, 2006)

Legend: *Interventions where small study numbers and/or no statistical analysis performed); **RCT:** Randomised Controlled Trial*

Table 7. Interventions in themes where *"only the intervention group was assessed and only post-intervention"*

**Theme: Information provision / communication**
Daily parent update letter from Electronic Patient Record (Palma et al, USA, 2012)
**Theme: Clinical care**
Tele-rounding robot, off-site neonatologist (Garingo et al, USA, 2016)
**Theme: Parent emotional support**
Listening visits (Segre et al, USA, 2013)

<u>Parent input into design of interventions</u>

Five studies (5/32, 16%) reported involving parents in intervention design, of which 2 reported improvement of parent satisfaction. The number of included studies was too

Confidential: For Review Only

small to estimate any effect of parent co-design on the success of interventions at study level.

Methodological quality

For the majority of RCT, key study characteristics, such as randomisation, allocation concealment and blinding of outcome assessment, were either not stated or unclear (Figure 2). Only one RCT had an available study protocol (retrospectively registered) and none described blinding of study participants and/or personnel. All RCT scored a high/unclear risk of bias in at least 4/6 Cochrane tool categories, except for one, which scored a high/unclear risk in 3/6 categories.

We assessed 21/22 non-RCT studies using the ROBINS-I tool (13), excluding the implementation project. All 21 studies were assessed as having an overall *serious* risk of bias and 7/21 of studies (33%) were further categorised as having *critical* risk of bias (Figure 3). Blinding of participants, personnel and outcome assessment was poorly reported across all non-RCT and no study reported a published study protocol. None of the included non-RCT measured or corrected for important parent/infant confounding variables, or other relevant neonatal unit co-interventions taking place at the same time as the intervention.

We were unable to use the *Standards for Reporting Implementation Studies (StaRI) Statement Tool*[17] for assessing the implementation project, as the reporting was incomplete.

Confidential: For Review Only

There was no association between methodological quality assessments and the studies' reported effect on parent satisfaction. All 4/10 RCT that reported a higher level of parent satisfaction associated with their intervention, scored a high/unclear risk of bias in at least 4/6 Cochrane tool categories, one of which scored high/unclear *risk* in all categories. Out of the 14/22 non-RCT reporting an improved parent satisfaction, two were deemed to be at *critical risk* of bias on the ROBINS- I tool, whilst the rest we assessed to be at *serious risk* of bias.

DISCUSSION

Parent satisfaction with neonatal care is increasingly recognised as an important measure of parent experience and is being used to evaluate hospitals and healthcare providers; use of interventions to improve parent satisfaction in neonatal units is increasing. This is the largest review of interventions where an outcome was parent satisfaction with neonatal care and includes 32 studies. We find low quality evidence that interventions targeting 'parent involvement' may improve parent satisfaction with neonatal care, but this result must be interpreted cautiously in view of the high risk of bias in included studies.

Overall, our review highlights the complexity of evaluating parent satisfaction. As a multidimensional construct, parent satisfaction can be affected just as much by interventions directly relating to infant care (eg. Kangaroo care) as well as interventions relating to neonatal care facilities (eg. Free parking). By grouping included interventions into themes (Tables 4-7) we have highlighted the variety of

Confidential: For Review Only

interventions available, as well as the majority of interventions being those relating to 'parent involvement'.

A key reason for only selecting parent satisfaction as the outcome of interest was to focus on a single component of parent experience, in order to reduce outcome heterogeneity and allow direct comparison. Despite this approach, the key methodological limitation identified in this review was inconsistency in how parent satisfaction is defined and measured; it is notable that the majority of questionnaires (23/29) lack validation. In keeping with neonatal studies more widely[18], this study confirms inconsistent outcome selection as a major source of research waste in neonatal studies examining parent experience, and further finds that there is limited involvement of parents in study design.

Strengths of our review include identifying studies with both mother and father participants, inclusion of the full range of infant gestations and a wide range of interventions. We followed a pre-registered protocol and report this review in line with PRISMA guidelines[11]. To further aid direct comparison of interventions, we only included studies that evaluated parent experience using ≥1 quantitative outcome of parent satisfaction. One limitation of this approach is that by excluding studies which evaluated parent experience using other measures (e.g. stress, anxiety and depressions scales) we are unable to comment on interventions that targeted these other components of parent experience.

Another limitation is that we have only included studies in the English language, due to resource and time constraints. By not including studies in other languages, it is

Confidential: For Review Only

possible our results are more focused on work conducted in specific countries. Furthermore, we acknowledge that much of the research in parent experience is qualitatively evaluated. By restricting our review to studies where ≥1 quantitative outcome of parent satisfaction is measured, we have not included any interventions with solely qualitative outcomes. This was in an attempt to enable direct comparison of interventions, which has previously not been possible in any published review. By not including studies evaluated by qualitative measures only, it is possible our results are more focused on a particular type of interventions where quantitative evaluation would be preferable and/or easier. It also means we may not have included all studies ever conducted on a particular intervention, where some were only evaluated qualitatively, making some interventions appear more 'widespread' than others.

Brett et al[19] systematically reviewed interventions aimed at improving the parent experience more widely, but only included parents of preterm infants. Their large number of outcome domains and heterogeneity of outcome measures (including studies that reported only qualitative outcomes) meant the authors we unable to draw firm conclusions about the efficacy of interventions and that comparison and meta-analysis was not possible. The majority of our review's studies have been published in the 7 years since the Brett review, highlighting the increasing interest in this area. However, despite including all gestations and focusing on a specific aspect of parent experience, heterogeneity in measurement of parent satisfaction meant we were also unable to conduct a quantitative synthesis. Inconsistency and lack of validation of instruments measuring parent satisfaction in neonatal care (specifically with family-centred care) has previously been highlighted by Dall'Oglio et al[20].

Confidential: For Review Only

Although 31% of included studies were RCT, all were assessed as having a high risk of bias. Randomised controlled trials are traditionally considered the highest-ranking form of evidence, however it is worth considering whether such a design is feasible or desirable to evaluate interventions targeting parent satisfaction. Parents in neonatal care talk to each other, compare notes and invariably create parent-support communities; hence it is inherently difficult to avoid contamination between parents receiving an intervention and those who are not, meaning that blinding of parents or health professionals is near impossible. Furthermore, parent satisfaction is likely to be particularly susceptible to the Hawthorne effect[21], requiring longer-term follow up. These factors may explain the low number of RCT identified in our review and the high risk of bias seen in those that were included. In non-RCT studies, the main methodological concern is the degree to which unmeasured and uncontrolled confounders may explain any differences seen between groups. The non-RCT studies included in this review were classed as having either a serious or critical risk of bias. The overwhelming majority of studies did not adequately report baseline variables or report other interventions during the study period, making it impossible to assess studies for selection bias or treatment bias. Furthermore, limitations such as contamination bias and the Hawthorne effect affect non-RCT as well. Only two non-RCT studies evaluated the outcome of interest (parent satisfaction) both before and after the intervention, in the same group of parents *(group level effect),* with most studies evaluating different parent groups pre and post intervention *(unit level effect).* An inherent weakness of this latter approach is that it assumes parent satisfaction is a static measure at the unit level, which is unlikely to be true. As a result of these numerous important limitations identified across all included studies, we find only low-quality evidence in support of interventions to improve parent satisfaction with

Confidential: For Review Only

neonatal care, despite a majority of studies reporting a beneficial effect of interventions. These limitations may explain the limited uptake of these interventions by the wider neonatal community.

Changing neonatal unit practices to incorporate any new intervention requires robust evidence. We demonstrate here that such evidence is not currently available for improving parent satisfaction. We highlight the use of non-randomised study designs, inconsistency in definition and measurement of parent satisfaction, the use of unvalidated questionnaires, methodological limitations and a lack of parent involvement as contributors. Our review empirically documents the extent of these issues in studies that use quantitative parent satisfaction surveys, and their contribution to research waste in neonatology.

Given the importance of parent satisfaction for both parent and offspring wellbeing, higher quality trials that involve parents, use standardised definitions and validated parent satisfaction measures are needed. Given the nature and challenges of the neonatal care environment and the limitations we have identified in existing research, a cluster trial may be the most appropriate study design to rigorously evaluate interventions to improve parent satisfaction with neonatal care.

CONCLUSIONS

Many interventions, commonly relating to parent involvement, are reported to improve parent satisfaction with neonatal care but inconsistency in definition and measurement of parent satisfaction and high risk of bias in all studies makes this low quality evidence. Standardised definitions and validated parent satisfaction measures

https://mc.manuscriptcentral.com/bmjpo

Confidential: For Review Only

are needed, as well as higher quality trials of parent experience, involving parents in intervention design.

What is already known on this topic

- Neonatal care significantly affects parents' mental health; parent satisfaction is increasingly being used as a parent experience measure
- Parent satisfaction is inversely related to parent stress; interventions improving parent satisfaction have the potential to reduce parent stress, improve parent-infant bonding and infant outcomes
- Use of interventions measuring parent satisfaction as an outcome in neonatal units is increasing, though few are formally evaluated and wider uptake is limited; it is not known the degree to which parents are involved in intervention design

What this study adds

- There is inconsistency in how parent satisfaction in neonatal care is defined and measured, and the majority of studies do not include parents in intervention design
- There is low quality evidence that interventions relating to parent involvement may improve parent satisfaction with neonatal care
- Standardised, validated measures of parent satisfaction and higher quality trials, involving parents in intervention design, are needed

DECLARATIONS

Confidential: For Review Only

Conflict of interest disclosure

SS has received research grants from the National Institute of Health Research (NIHR), the NIHR CLAHRC NWL, Rosetrees Trust and CW+ charity. NM is Director of the Neonatal Data Analysis Unit at Imperial College London. In the last five years NM has served on the Board of Trustees of the Royal College of Paediatrics and Child Health, David Harvey Trust, Medical Women's Federation and Medact; and is a member of the Nestle Scientific Advisory Board. NM has received research grants from the British Heart Foundation, Medical Research Council, National Institute of Health Research, Westminster Research Fund, Collaboration for Leadership in Applied Health and Care Northwest London, Healthcare Quality Improvement Partnership, Bliss, Prolacta Life Sciences, Chiesi, Shire and HCA International; travel and accommodation expenses from, Nutricia, Prolacta, Nestle and Chiesi; honoraria from Ferring Pharmaceuticals and Alexion Pharmaceuticals for contributions to expert advisory boards, and Chiesi for contributing to a lecture programme. CG is funded by the United Kingdom Medical Research Council (MRC) through a Clinician Scientist Fellowship award. He has received support from Chiesi Pharmaceuticals to attend an educational conference; in the past 5 years he has been investigator on received research grants from Medical Research Council, National Institute of Health Research, Canadian Institute of Health Research, Department of Health in England, Mason Medical Research Foundation, Westminster Medical School Research Trust and Chiesi Pharmaceuticals. IA, JW, DB: None to declare.

Authors' contributions

Confidential: For Review Only

SS and CG conceived this systematic review. The protocol was created by SS and CG. Searches were performed by SS and IA. All search results were reviewed by SS, and JW. Coding was completed by SS and JW. Data analysis was completed by SS. The first draft of the manuscript was written by SS; SS, CG and JW edited and reviewed the manuscript. All authors approved the manuscript. This article presents independent research supported by the National Institute for Health Research (NIHR) The views expressed in this publication are those of the authors and not necessarily those of the NHS, the NIHR or the Department of Health and Social Care.

Funding

This work is sponsored by Imperial College London and supported by a peer-reviewed National Institute of Health Research Doctoral Research Fellowship, awarded to SS (DRF-2017-10-172).

References

1. Neonatal Data Analysis Unit. Neonatal Data Analysis Unit Annual Report 2017, 2018. Available: https://www.rcpch.ac.uk/sites/default/files/2018-10/2018_nnap_report_on_2017_data_final_v8.pdf

2. Lefkowitz DS, Baxt C, Evans JR. Prevalence and Correlates of Posttraumatic Stress and Postpartum Depression in Parents of Infants in the Neonatal Intensive Care Unit (NICU). *J Clin Psychol Med Settings* 2010;17(3):230-7.

3. Shaw RJ, Bernard RS, DeBlois T et al. The Relationship Between Acute Stress Disorder and Posttraumatic Stress Disorder in the Neonatal Intensive Care Unit. *Psychosomatics* 2009;50(2):131-7.

Confidential: For Review Only

4. Beck CT, Woynar J. Posttraumatic Stress in Mothers While Their Preterm Infants Are in the Newborn Intensive Care Unit: A Mixed Research Synthesis. *ANS Adv Nurs Sci* 2017;40(4):337-55.

5. Lee SK, O'Brien K. Parents as primary caregivers in the neonatal intensive care unit. *CMAJ* 2014;186(11):845-7.

6. Grace SL, Evindar A, Stewart DE. The effect of postpartum depression on child cognitive development and behavior: a review and critical analysis of the literature. *Arch Womens Ment Health* 2003;6(4):263-74.

7. Rocha G, Candeias L, Ramos M et al. Stress and satisfaction of mothers in neonatal intensive care. *Acta Med Port* 2011;24(2):157-66.

8. Lopez-Maestro M, Sierra-Garcia P, Diaz-Gonzalez C et al. Quality of attachment in infants less than 1500g or less than 32 weeks. Related factors. *Early Hum Dev* 2016;104:1-6.

9. Charpak N, Tessier R, Ruiz JG et al. Twenty-year Follow-up of Kangaroo Mother Care Versus Traditional Care. *Pediatrics* 2017;139(1):e20162063.

10. PROSPERO database. Available: http://www.crd.york.ac.uk/prospero/display_record.asp?ID=CRD42016042110

11. Moher D, Liberati A, Tetzlaff J et al. Preferred reporting items for systematic reviews and meta-analyses: the PRISMA statement. *J Clin Epidemiol* 2009;62(10):1006-12.

12. Higgins JP, Altman DG, Gotzsche PC et al. The Cochrane collaboration's tool for assessing risk of bias in randomised trials. *BMJ* 2011;343:d5928.

13. Sterne JA, Hernan MA, Reeves BC et al. ROBINS-I: a tool for assessing risk of bias in non-randomised studies of interventions *BMJ* 2016;355:i4919.

14. Green JTN. Qualitative Methods for Health Research. SAGE, 2014.

Confidential: For Review Only

15. Duley L, Uhm S, Oliver S et al. Top 15 UK Research Priorities for Preterm Birth. *The Lancet* 2014;383(9934):2041-2042.

16. Mitchell-DiCenso A, Guyatt G, Paes B et al. A new measure of parent satisfaction with medical care provided in the neonatal intensive care unit. *J Clin Epidemiol* 1996;49(3):313-318.

17. Pinnock H, Barwick M, Carpenter C et al. Standards for Reporting Implementation Studies (StaRI) statement. *BMJ* 2017;356:i6795.

18. Webbe JWH, Ali S, Sakonidou S et al, Inconsistent outcome reporting in large neonatal trials: a systematic review, *Arch Dis Child Fetal Neonatal Ed* 2019 doi: 10.1136/archdischild-2019-316823. [Epub ahead of print]

19. Brett J, Staniszewska S, Newburn M et al. A systematic mapping review of effective interventions for communicating with, supporting and providing information to parents of preterm infants. *BMJ Open* 2011;1(1):e000023.

20. Dall'Oglio I, Mascolo R, Gawronski O et al. A systematic review of instruments for assessing parent satisfaction with family-centred care in neonatal intensive care units. *Acta Paediatr* 2018;107:391-402.

21. McCambridge J, Witton J, Elbourne DR. Systematic review of the Hawthorne effect: new concepts are needed to study research participation effects. *J Clin Epidemiol* 2014;67(3):267–277.

Figure / Table Legends

Figure 1: PRISMA Flow diagram of selected studies

Figure 2. Cochrane Collaboration Risk of Bias tool assessment (RCT)

Legend: Green- low risk of bias; Yellow- unclear risk of bias; Red- high risk of bias

Confidential: For Review Only

Figure 3. ROBINS-I risk of bias assessment (Non-RCT)

Table 1. Included Randomised Controlled Trials (RCT)

Table 2. Included Prospective Cohort Studies

Table 3. Included "Other" non-Randomised Controlled Trials (non-RCT)

Legend for Tables 1-3: *Number in last column illustrates each intervention's reported effect on parent satisfaction:* *1. Parent satisfaction was statistically significantly higher in the intervention group;* *2. Parent satisfaction was not reported to be statistically significantly different in the intervention group; 3. Unclear if parent satisfaction improved (small study numbers and/or no statistical analysis performed); 4. Only the intervention group was assessed and only post-intervention*

Table 4. "*Effective"* interventions in themes

Legend: *Interventions where parent satisfaction was reported to be statistically significantly higher in the intervention group*. ***RCT:*** *Randomised Controlled Trial*

Table 5. "*Ineffective"* interventions in themes

Legend: *Interventions where parent satisfaction was not reported to be statistically significantly different in the intervention group;* ***RCT:*** *Randomised Controlled Trial*

Table 6. *"Unclear if effective"* interventions in themes

Legend: *Interventions where small study numbers and/or no statistical analysis performed);* ***RCT:*** *Randomised Controlled Trial*

Confidential: For Review Only

Table 7. Interventions in themes where *"only the intervention group was assessed and only post-intervention"*

Online supplementary files

File 1. OVID MEDLINE search strategy

Research checklist

PRISMA checklist

Confidential: For Review Only

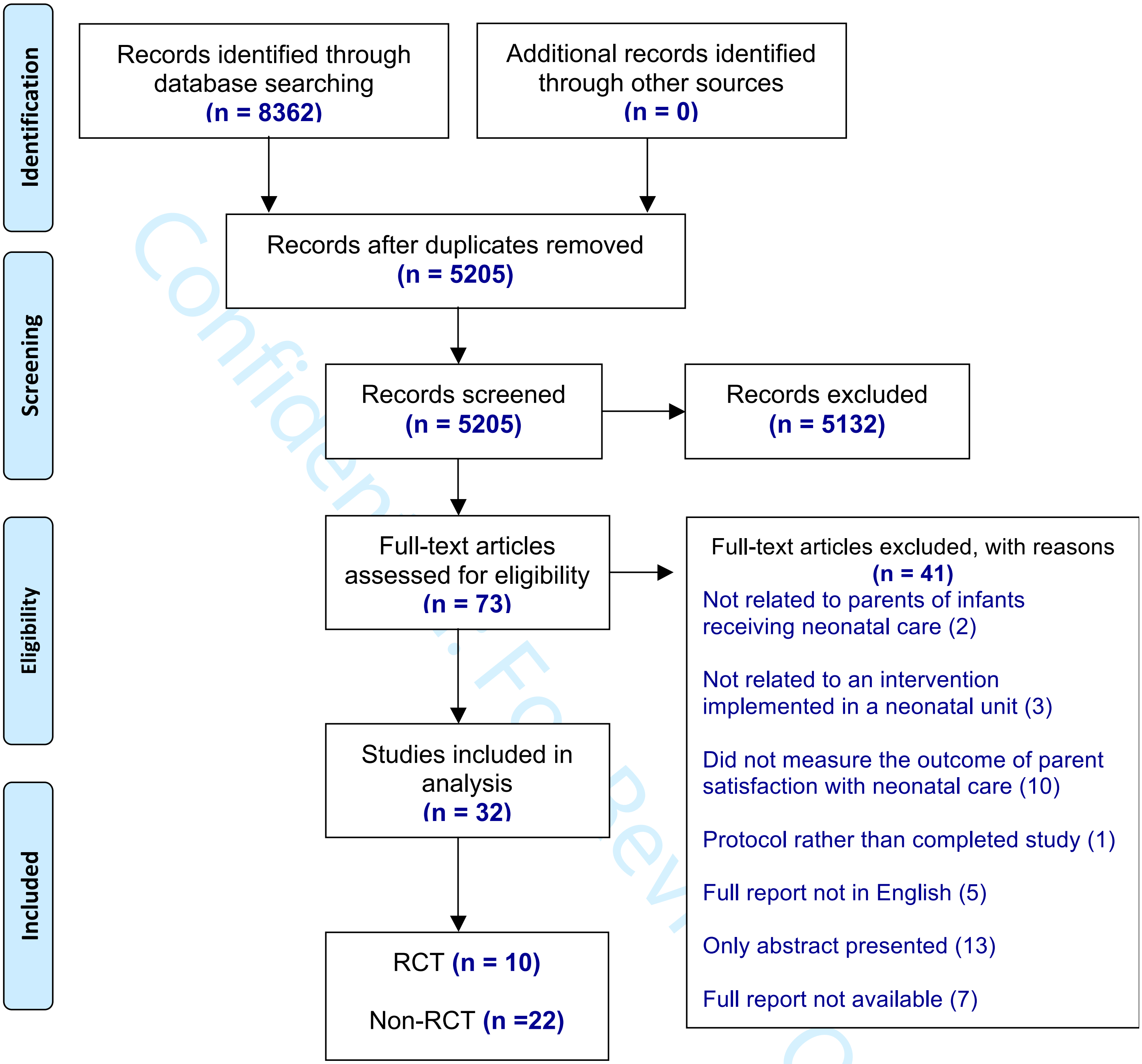

Confidential: For Review Only

Risk of Bias (Cochrane)

**Author by publication year**	**Random sequence generation**	**Allocation concealment**	**Blinding of participants and personnel**	**Blinding of outcome assessment**	**Incomplete outcome data**	**Selective reporting**
1. Northrup (2016)	?	+	-	?	+	?
2. Abdel-Latif (2015)	+	+	-	-	-	?
3. Bastani (2015)	?	?	-	?	+	?
4. Clarke-Pounder (2015)	?	?	-	?	+	?
5. Holditch-Davis (2013)	+	+	-	+	?	?
6. Franck (2011)	-	?	-	?	-	+
7. Livingston (2009)	?	?	-	?	+	?
8. Koh (2007)	?	?	-	?	?	?
9. Mitchell-DiCenso (1996)	+	?	?	?	?	?
10. Broyles (1992)	?	?	-	+	+	?

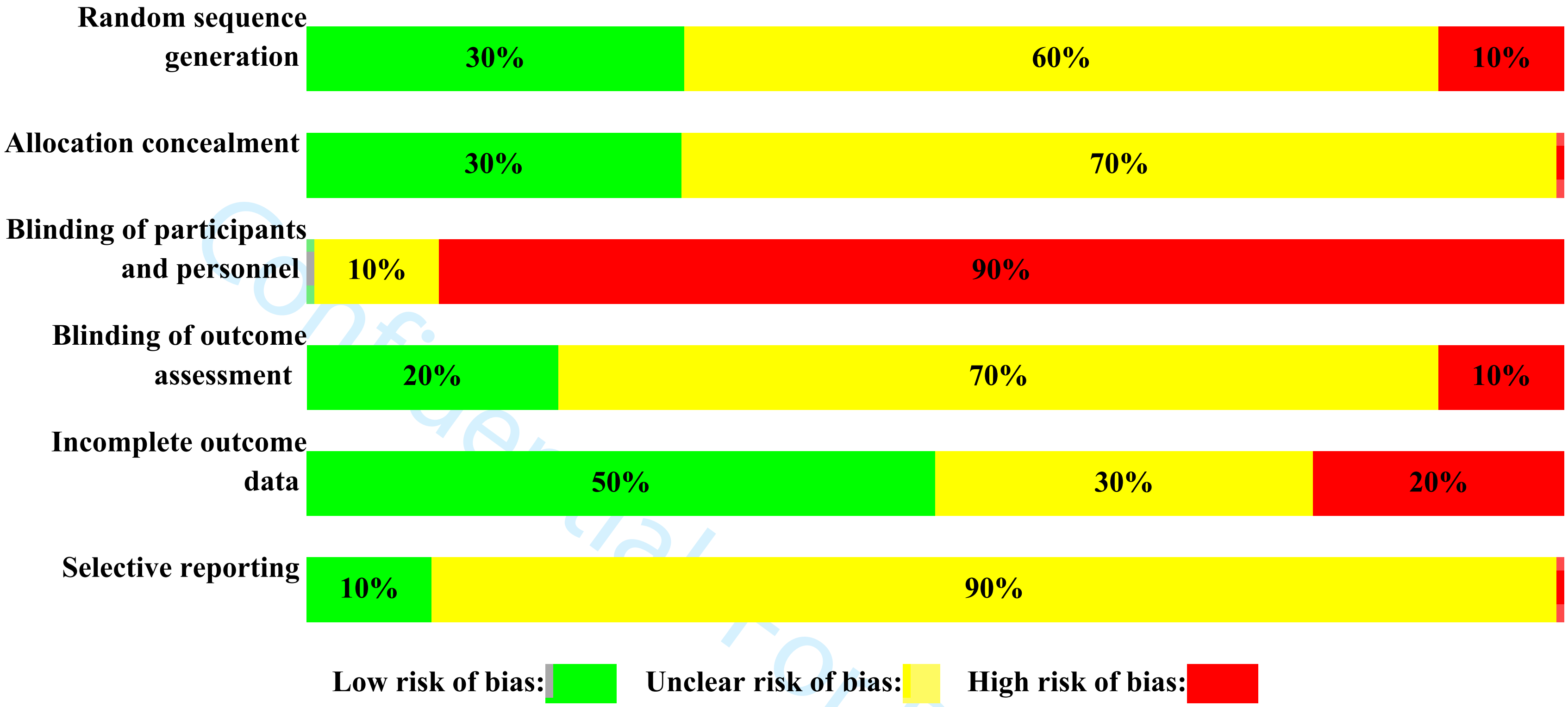

Confidential: For Review Only

Risk of Bias (ROBINS-I)

Author by publication year	Bias due to confounding	Bias in selection of participants into the study	Bias in classification of interventions	Bias due to deviations from intended interventions	Bias due to missing data	Bias in measurement of outcomes	Bias in selection of the reported result	OVERALL risk of bias
1. De Bernardo (2017)	SERIOUS	LOW	LOW	SERIOUS	LOW	SERIOUS	SERIOUS	SERIOUS
2. Kadivar (2017) *Internet-based education*	SERIOUS	LOW	LOW	SERIOUS	LOW	SERIOUS	MODERATE	SERIOUS
3. Kadivar (2017) *Narrative writing*	SERIOUS	SERIOUS	LOW	SERIOUS	LOW	SERIOUS	MODERATE	SERIOUS
4. Garingo (2016)	CRITICAL	LOW	LOW	SERIOUS	SERIOUS	SERIOUS	SERIOUS	CRITICAL
5. Globus (2016)	SERIOUS	LOW	LOW	NO INFO	SERIOUS	SERIOUS	SERIOUS	SERIOUS
6. Kazemian (2016)	SERIOUS	NO INFO	LOW	SERIOUS	NO INFO	SERIOUS	SERIOUS	SERIOUS
7. Petteys (2015)	SERIOUS	LOW	LOW	SERIOUS	MODERATE	SERIOUS	MODERATE	SERIOUS
8. Van de Vijver (2015)	CRITICAL	LOW	LOW	SERIOUS	MODERATE	SERIOUS	MODERATE	CRITICAL
9. Voos (2013)	CRITICAL	LOW	LOW	SERIOUS	NO INFO	SERIOUS	SERIOUS	CRITICAL
10. Segre (2013)	CRITICAL	NO INFO	LOW	SERIOUS	SERIOUS	SERIOUS	MODERATE	CRITICAL
11. Palma (2012)	CRITICAL	NO INFO	LOW	SERIOUS	SERIOUS	SERIOUS	CRITICAL	CRITICAL
12. Stevens (2011)	SERIOUS	LOW	LOW	SERIOUS	SERIOUS	SERIOUS	MODERATE	SERIOUS
13.Voos (2011)	SERIOUS	LOW	LOW	SERIOUS	SERIOUS	SERIOUS	MODERATE	SERIOUS
14. Weiss (2010)	SERIOUS	LOW	LOW	SERIOUS	LOW	SERIOUS	MODERATE	SERIOUS
15. Foster (2008)	SERIOUS	CRITICAL	LOW	SERIOUS	SERIOUS	SERIOUS	MODERATE	CRITICAL
16. Byers (2006)	SERIOUS	LOW	LOW	SERIOUS	SERIOUS	SERIOUS	MODERATE	SERIOUS
18. Wielenga (2006)	SERIOUS	LOW	LOW	SERIOUS	LOW	SERIOUS	MODERATE	SERIOUS
19. Penticuff (2005)	SERIOUS	LOW	LOW	SERIOUS	LOW	SERIOUS	MODERATE	SERIOUS
20. Byers (2003)	SERIOUS	LOW	LOW	SERIOUS	LOW	SERIOUS	SERIOUS	SERIOUS
21. Polizzi (2003)	SERIOUS	MODERATE	LOW	SERIOUS	SERIOUS	SERIOUS	MODERATE	SERIOUS
22. Legault (1995)	SERIOUS	CRITICAL	LOW	CRITICAL	LOW	SERIOUS	MODERATE	CRITICAL

1. intervention$.ti,ab.
2. tool$.ti,ab.
3. way$.ti,ab.
4. updat$.ti,ab.
5. method$.ti,ab.
6. information.ti,ab.
7. sms.ti,ab.
8. implement$.ti,ab.
9. 1 or 2 or 3 or 4 or 5 or 6 or 7 or 8
10. bab$3.mp.
11. preterm$.ti,ab.
12. pre term.ti,ab.
13. premature.ti,ab.
14. postterm.ti,ab.
15. post term.ti,ab.
16. infant$.ti,ab.
17. newborn$.ti,ab.
18. exp Infant, Newborn/
19. 10 or 11 or 12 or 13 or 14 or 15 or 16 or 17 or 18
20. neonatal intensive care.ti,ab.
21. neonatal unit$.ti,ab.
22. NICU.ti,ab.
23. SCBU.ti,ab.
24. neonatal itu.ti,ab.
25. special care baby unit$.ti,ab.
26. neonat$.ti,ab.
27. Intensive Care Units, Neonatal/
28. Intensive Care Units/
29. Critical Care/
30. Neonatal Nursing/
31. 20 or 21 or 22 or 23 or 24 or 25 or 26 or 27 or 28 or 29 or 30
32. parent$.ti,ab.
33. mother$.ti,ab.
34. father$.ti,ab.
35. exp Parents/

36. 32 or 33 or 34 or 35
37. satisfaction.ti,ab.
38. experience$.ti,ab.
39. Patient Satisfaction/
40. personal satisfaction/
41. communicat$.ti,ab.
42. exp Communication/
43. Health Communication/
44. Information Dissemination/
45. 37 or 38 or 39 or 40 or 41 or 42 or 43 or 44
46. 9 and 19 and 31 and 36 and 45

Confidential: For Review Only

Randomised controlled trials (RCT) by publication year

Author (Date), Country	Parent Gender/ sample size	Infants Gestation age (GA) in weeks /NICU level	Study design	Intervention	Outcome measures	Timing of measurement	Method of measurement	Results	Parent co-design?	Improved parent satisfaction?
1. Northrup et al. (2016), USA	Mothers and fathers /116	<28 / level III	Randomised controlled trial	Intervention: **Free Parking (FP).**<br><br>Parents received 7 parking vouchers at a time (value: $10/each) and continued to receive vouchers until infant discharge. Each voucher allowed free entry and exit for 24hr.<br><br>Control: Parents received the standard care and did not receive vouchers.	***Parent satisfaction with NICU care***	After babies were discharged (once)<br><br>- During the first high-risk-infant clinic visit after discharge<br><br>No pre-intervention parent satisfaction data available for comparison.	*Satisfaction questionnaire*<br><br>Validation: No content validity or reliability testing reported.<br><br>11 questions<br><br>- Seven items were summed (score 7-35) to measure "Support" (e.g., information sharing).<br><br>- Three items measured "Emotional Connection" to the infant (score 3-15)<br><br>- One item assessed “family involvement in infant care” (responses: not enough-just right-too much).<br><br>Greater scores indicated higher perceived support, connection and satisfaction.	**The groups did not differ significantly with respect to satisfaction.**<br><br>**Interv Control p-value**<br>NICU support<br>**Mean (SD)** 30(2.7) 28.7(3.7) 0.07<br><br>Emotional connection<br>12.3(1.7) 12.3(1.7) 0.96<br><br>Family involvement "Just right"<br>81.4% 85% 0.07	**No**	**2**
2. Abdel-Latif et al. (2015), Australia	Mothers and fathers /63	25-42 / level III	Cross-over Randomised Controlled Trial	Intervention: **Parental Presence at Clinical Bedside Rounds (PPCBR).**<br><br>Parents attended bedside clinical rounds. Parents had opportunity to ask questions about their baby’s condition and management.<br><br>Control: Parents received the standard care with no parental presence at bedside clinical rounds.	***Parent satisfaction assessed by questions of 3 domains:***<br>*1. Knowledge and understanding*<br>*2. Communication and collaboration*<br>*3. Privacy and confidentiality*	During babies’ admission (once)<br><br>- At the end of each study arm, separated by a washout period<br><br>- No pre-intervention parent satisfaction data available for comparison	*Satisfaction questionnaire*<br><br>The authors stated “the research team designed the questionnaire”.<br><br>Validation: No content validity or reliability testing reported.<br><br>Number and format of questions: not stated	**PPCBR had significantly higher adjusted mean (95% CI) scores for some questions from domains 1 and 2.**<br><br>Domain 3 was comparable between the two study groups.<br><br>**Interv Control p-value**<br>Domain 1 question:<br>*“I have received adequate information about my baby’s condition and management”*<br>**Mean** 4.321 3.947 0.03<br><br>Domain 2 questions:<br>*“In the last week I have been able to communicate effectively with my baby’s healthcare team”*<br>**Mean** 4.407 4.250 0.05<br><br>*“In the last week I have collaborated with my baby’s healthcare team in the*	**No**	**1**

Confidential: For Review Only

								planning of care for my baby"<br>**Mean** 3.843 3.426 0.02<br><br>*"In the last week I have been able to ask the healthcare team questions about my baby's care"*<br>**Mean** 4.642 4.259 0.004		
3. Bastani et al, (2015), Iran	Mothers /100	30-37<br>Mean (SD)<br><br>Control: 33.90 (2.33)<br><br>Interv: 34 (1.9)<br><br>/ level not stated	Randomised Controlled Trial (block randomisation)	Intervention: **Family-centered Care (FCC).**<br><br>Mothers allowed access to their baby at any time, participated in the care process and were provided with information about neonatal care.<br><br>Control: Mothers received the standard care where they were only allowed to be present at the time of the infant's entry to the neonatal care unit, and were only routinely informed.	***Maternal satisfaction* relating to three themes:**<br>1. Parental presence<br>2. Participation in neonatal care<br>3. Information about neonatal care	During babies' admission (twice)<br><br>- 24 hours after admission<br>- At the time of discharge	*Satisfaction questionnaire* (Validated)<br><br>A modified satisfaction questionnaire was used, based on a parental satisfaction instrument developed for measuring satisfaction in Paediatric intensive care Units (PICU).<br><br>18 questions<br><br>Graded 0 (very dissatisfied) to 4 (very satisfied).<br><br>The overall satisfaction rate was classified based on the mean scores (score<50%, between 75-50% and > 75%).	**In the FCC group, pre and post intervention difference in maternal satisfaction was statistically significant p<0.001**<br><br>**Interv Control p-value**<br>**Mean (SD)**<br><br>**At 24 hr**<br>22.36(8.90) 22.06(9.77) 0.87<br><br>**At discharge**<br>59.28(6.86) 30.18(14.09) <0.01	**Unclear**<br><br>Mothers determined the reliability of the satisfaction tool and approved the educational pamphlet. Authors did not report if mothers had direct input in the intervention design.	1
4. Clarke-Pounder et al. (2015), USA	Mothers and fathers /19 families	23-39 / level III	Randomised Controlled Trial	Intervention: **Sharing information obtained from parent interviews with the primary NICU provider.**<br><br>Parents interviewed using the *NICU- adapted Decision Making Tool (N-DMT).* Information obtained was placed in the electronic medical record (EMR) and shared with the primary neonatal provider via email. Daily rounds on all infants were audio-recorded for 3 days after enrollment to see if information from the N-DMT was incorporated into daily care planning.<br><br>Control: The content of a recent social work note was communicated with the primary provider via e-mail, creating an attentional control group.	***Parent satisfaction with care***	During babies' admission (once)<br><br>- 2 weeks after study entry<br><br>No pre-intervention parent satisfaction data available for comparison.	*Satisfaction questionnaire*<br><br>A *NICU- adapted Decision Making Tool (N-DMT)* – specific questionnaire was used.<br><br>Validation: Partially reported. Authors stated reliability testing took place; no information on content validity provided.<br><br>8 questions: e.g."My baby's doctors considered my goals and hopes for my baby during decision-making".<br><br>Likert scale (1 strongly agree-4 strongly disagree). Total N-DMT score range 8–32.	**There was no significant difference in satisfaction with care as measured by the N-DMT scale between the control group and intervention groups in a univariable model or multiple variable model controlling for gestational age.**<br><br>**Interv Control**<br>**Median** (range)<br>26(15–28) 28.8(19–32)<br><br>**No p-value reported**<br><br>There was, however, a pattern of decreased satisfaction with care among the intervention group compared to the control group across the N-DMT-specific survey questions, although the differences were not statistically significant.	**Yes**<br><br>Information obtained from parents using the N-DMT was placed in the electronic medical record (EMR) and shared with the primary NICU provider via email (forming the intervention)	2
5.Holditch-Davis et al. (2013), USA	Mothers /208	Preterm infants	Randomised controlled trial	Interventions: **1. Mothers were taught how to massage infants with auditory, tactile, visual,**	**1. *Parent (mother) satisfaction with the***	During admission period and post discharge	*Satisfaction questionnaire*<br><br>The questionnaire was designed by the study team.	**No significant differences occurred between the groups.**	**No**	2

Confidential: For Review Only

		Mean (SD)<br><br>Overall group 27.2 (3.0)<br><br>/ 4 centres, levels II-III	3 groups (2 intervention and 1 control)<br><br>Post-intervention testing only.	**and vestibular stimulation (ATVV intervention)**<br>**2. Kangaroo care**<br><br><u>Control:</u> Attention control group. Mothers spent a similar amount of time with the study nurse discussing the equipment needed for preterm infant care at home. Study nurses provided education and support for all three groups. Mothers were not prevented from engaging in interventions of the other groups but did not receive formal education from the study nurse on the other interventions.	***intervention***<br><br>***2. Satisfaction with the helpfulness of the study nurse***<br><br>***3. Whether the mother would recommend the study to others and the degree of change in the mother as a person and as a mother as a result of being in the study.***	- At the time of discharge<br><br>- At 2 months corrected age<br><br>No pre-intervention parent satisfaction data available for comparison.	<u>Validation:</u> Partially reported. Authors stated reliability testing took place; no information on content validity provided.<br><br><u>26 questions:</u> relating to three dimensions of satisfaction: efficacy, caring, and technical quality.<br><br>Likert (1 least satisfied-5, 5 most satisfied)	Mothers in all three groups were satisfied with the intervention (mean scores of 3.3 or higher on a 5-point scale) and the helpfulness of the nurse (mean scores of 4.6 or higher on a 5-point scale).		
6. Franck et al. (2011), UK	Mothers and fathers /169	Mean (SD)<br><br>Control: 31.94 (5.17)<br><br>Interv: 29.40 (3.17)<br><br>/4 centres, level III	Cluster Randomised Controlled Trial	<u>Intervention:</u> **Increasing parental involvement in infant pain management in the NICU.**<br><br>Parents received a booklet providing evidence-based information about pain and comforting infants in the NICU setting. Parents received 2 visits from a research nurse showing them how to apply the comforting techniques described in the booklet.<br><br><u>Control:</u> As part of usual care, parents in both the intervention and control groups received a detailed booklet with generic information about NICU care. Parents in the control group also received 2 visits from a research nurse listening to what parents had to say about their NICU experience (attention placebo).	At baseline:<br><br>***1. Parent satisfaction with NICU care***<br><br>One week after the intervention:<br><br>***1. Satisfaction with information about pain control***<br><br>***2. Satisfied nurses make infant comfortable***<br><br>***3. Satisfied pain medicines help infant***	During babies' admission (twice)<br><br>-At baseline (within 3 to 7 days of admission)<br><br>- 1 week after the intervention	*Individual questions*<br><br><u>Validation:</u> No content validity or reliability testing reported.<br><br>1. At baseline:<br><br>Parent satisfaction was measured by 1 question: *"Satisfaction with NICU care"* (1 very satisfied-6 very unsatisfied) as part of the baseline parent characteristics questionnaire.<br><br>2. One week after the intervention:<br><br>Three questions using the word "satisfied' were selected from the validated *Parent Attitudes About Infant Nociception (PAIN)* survey (Likert scale 1 very satisfied-6 very unsatisfied)	**At baseline: there was no significant difference in satisfaction between intervention and control group**<br><br>**Interv** **Control**<br>**Mean (SD)** 1.45(0.71) 1.51(0.76)<br><br>**p-value** missing<br><br>**1 week after the intervention: Intervention parents were more satisfied with the information about pain control received than control parents.**<br><br>**Interv** **Control**<br>**Mean (SD)** 2.10(0.97) 3.28(1.27)<br>**p-value** < 0.001	**Yes**<br><br>The booklet was reviewed by 12 parents of infants who had been cared for in NICUs in the United Kingdom.	**1**
7.Livingston et al. (2009), USA	Mothers /12	Mean (SD)<br><br>Control:	Randomised Controlled Trial	<u>Intervention:</u> **Touch and massage.**<br><br>Mothers attended a 1hr massage class taught by a	***1. Caregiver (mother) satisfaction with their infant's care***	During babies' admission (three times)<br><br>- At baseline	*Satisfaction questionnaire*<br><br>Two questionnaires were developed by the research team.	**It is unclear in the report if specific between-group comparisons and statistical analysis were conducted.**	**No**	**3**

Confidential: For Review Only

		33.4 (6.4)<br><br>Interv:<br>38.5 (3.1)<br><br>/ level III		nurse CIMI (certified infant massage instructor) and were asked to participate in at least 3 bedside massage instruction sessions taught within the next week. Infants received massage for 7 consecutive days, from the mother or a CIMI. The touch procedure lasted 20 minutes.<br><br><u>Control:</u> Infants received all usual hospital services including medical care, physical and occupational therapy services and developmentally supportive nursing care.	**2. *Caregiver satisfaction with the neonatal unit and the massage therapist***	- Upon completing the 7-day massage program<br><br>- 1 month following intervention	<u>Validation:</u> No content validity or reliability testing reported.<br><br>-1st questionnaire (at baseline): a brief self-report questionnaire about caregiver satisfaction with their infant's care until that moment. No further details reported.<br><br>-2nd questionnaire (upon completing the 7-day massage program and 1 month following intervention): a 10-minute satisfaction questionnaire relating to infant's response and caregiver satisfaction with the neonatal unit and the massage therapist.<br><br><u>Number of questions:</u> not stated.<br><br>Likert scale (1 very dissatisfied-4 very satisfied).<br><br>Sample statements: *'How satisfied do you feel giving massage to your infant?'; 'I feel that massage improved my infant's hospital stay.'*	<u>At baseline and day 7:</u><br>All caregivers were highly satisfied with the medical treatment their infant received.<br><br><u>At day 7 and 1 month follow-up:</u><br>All caregivers participating in the massage group reported high levels of satisfaction regarding their relationship with their infant and the massage program's impact on that relationship.<br><br>Slight improvements in satisfaction regarding time the caregiver spent with the infant and involvement in the infant's care were observed between day 7 and the 1-month follow-up (no further information reported).		
8. Koh et al. (2007), Australia	Mothers /200	Not stated / not stated	Randomised, Controlled Trial	<u>Intervention:</u> **Provision of taped conversations with neonatologists to mothers.**<br><br>The initial conversation and subsequent conversations of significance with a neonatologist were taped and analysed (for both groups). Mothers received a tape of each conversation and a tape recorder.<br><br><u>Control:</u> Usual care. Mothers were not given the tape or recorder.	***Satisfaction with conversations held with the neonatologist***<br><br>***Satisfaction with the tape***	During admission period and post discharge<br><br>- At 10 days<br><br>- At 4 months<br><br>- At 12 months<br><br>No pre-intervention parent satisfaction data available for comparison.	*Individual questions and a satisfaction scale*<br><br><u>Validation:</u> No content validity or reliability testing reported.<br><br><u>Number of questions:</u> not stated.<br><br>Likert scale (1-5 most satisfied)<br><br>Questions related to: Satisfaction with amount and quality of information presented, doctors' communication skills, patient's participation in the conversation.<br><br>A satisfaction scale was used to assess: Satisfaction with the tape	No differences were found between the two groups in satisfaction with conversations.<br><br>**Mothers of babies with a poor outcome in the tape group were, however, significantly more satisfied with the conversations:**<br><br>**Interv Control**<br>**Mean (95%CI)** 115(104-123.2) 100.5(94.1-109.4)<br><br>**p-value** 0.0051<br><br>Most (71-92%) of the mothers given the tapes stated that they helped their understanding, reminded them of what had been said, and helped their family to understand and recall information.	**No**	**1**

Confidential: For Review Only

9. Mitchell-DiCenso et al. (1996), Canada	Mothers and fathers/ 482	Mean (SD)<br><br>Interv: 35.1 (4.5)<br><br>Control: 35 (4.3)<br><br>/ level III	Randomised, Controlled Trial	<u>Intervention:</u> **Clinical Nurse Specialist/ neonatal practitioner team (CNS/NP) care.**<br><br>Infants of intervention parents were assigned to be cared for by the Clinical nurse special/neonatal practitioner CNS/NP team during the day and by paediatric residents during the night.<br><br><u>Control:</u> Paediatric residents cared for infants of control parents around the clock. Neonatologists supervised both teams.	***Parent satisfaction with care***	During admission period and post discharge (twice)<br><br>- On 5th day after admission (full survey)<br><br>- After discharge over the phone (only questions related to satisfaction with discharge process)<br><br>No pre-intervention parent satisfaction data available for comparison.	*Satisfaction questionnaire (Validated)*<br><br>The study team developed and used the validated *Neonatal Index of Parent Satisfaction (NIPS)* questionnaire.<br><br><u>Number of questions:</u> not stated.<br><br>NIPS score range (27-189); higher scores indicating greater satisfaction with care.	**No statistically significant difference between groups.**<br><br>**Interv Control p-value**<br>**NIPS Mean** 140 139 0.67<br><br>**Difference in means** 1.0, CI (-3.6-5.6)	**No**	**2**
10. Broyles et al. (1992), USA	Mothers /25	Mean (SD)<br><br>Control: 34 (4)<br><br>Interv: 33.4 (4)<br><br>/ level III	Randomised Controlled Trial	<u>Intervention:</u> **Detailed consent.**<br><br>Mothers were given information about mechanical ventilation. Detailed risk/benefit disclosure was provided both verbally and in writing.<br><br><u>Control:</u><br>Mothers were given a brief verbal description about mechanical ventilation supplemented with detailed verbal and written disclosure if desired by them (flexible consent).	***Maternal satisfaction with the information provided about mechanical ventilation***	During babies' admission (once)<br><br>- 24-48 hours after the intervention<br><br>No pre-intervention parent satisfaction data available for comparison.	*An interview* evaluating maternal satisfaction with the information provided about mechanical ventilation.<br><br><u>Validation:</u> A psychiatrist with a special interest in interviewing techniques was consulted in designing and standardising this assessment.<br><br>A research nurse conducted the interview, "checking" each mother against one option regarding:<br>- Amount of information: Right amount-Too much-Too little<br><br>- Information made coping: More Difficult-Easier-No effect-Uncertain.	This study is measuring and comparing satisfaction with two different interventions (detailed vs flexible consent process), neither of which formally represent the usual routine care for all babies (no control).<br><br>**Small numbers. No data indicating statistical analysis conducted or evidence of statistically significant results.**<br><br>**Detailed Flexible**<br>**Right amount of information** 75% mothers 100%<br><br>**Too little information** 25% mothers<br><br>**Made coping easier** 67% mothers 69%	**No**	**3**

Confidential: For Review Only

Prospective cohort studies by publication year

Author (Date), Country	Parents' gender/ sample Size	Infant Gestation age (GA) in weeks /NICU level	Study design	Intervention	Outcome measures	Timing of measurement	Method of measurement	Results	Parent co-design?	Improved parent satisfaction?
1. De Bernardo et al (2017), Italy	Mothers and Fathers /96	Mean (SD)<br><br>Control: 34.2 (5.25)<br><br>Interv: 32.7 (5.25)<br><br>/ level III	Non-randomized, prospective cohort pilot study<br><br>*Unit level effect*:<br>Two different time periods	Intervention: **FCC (Family-Centered Care).**<br><br>Parents had access to NICU for 8 hours/day. The NICU was widened and paediatric nurses taught parents procedures/practices for 10 days. Parents could observe clinical bedside rounds, hold meetings with the physicians, use the rooms and kitchen.<br><br>Control: Parents were permitted to visit their baby in NICU for 1 hour a day.	***Parent satisfaction relating to 3 specific domains:***<br><br>1. Knowledge and Understanding<br><br>2. Communication and Collaboration<br><br>3. Privacy and confidentiality	During babies' admission (once)<br><br>- At discharge (pre-FCC cohort and post-FCC cohort)<br><br>No pre-intervention parent satisfaction data available for comparison (different parent groups pre and post intervention).	*Satisfaction questionnaire.*<br><br>Validation: The authors state the survey *"was designed and validated by Abdel-Latif et al"*. No content validity or reliability testing reported in the original paper.<br><br>9 questions<br><br>3 questions: Related to adequate and timely information about the baby's condition.<br><br>3 questions: Related to communication and collaboration with the healthcare team.<br><br>3 questions: Related to respect of patient privacy.<br><br>Likert (1 strongly disagree-5 strongly agree)	**7/9 individual statements in the parent satisfaction questionnaire scored higher in the FCC compared to the NFCC (statistically significant difference).**<br><br>Example statement:<br>*"I have received adequate information about my baby's condition and management."*<br><br>**Median** Interv 5 (3.45-5) Control 4 (3-5)<br><br>**p-value** <0.05	**No**	**1**
2. Petteys et al. (2015), USA	Not stated/ 10 parents included in sample analysis	24-36+ / level III	A prospective cohort design.<br><br>A feasibility study.<br><br>*Group level effect:*<br>Intervention/ control groups<br><br>Post-intervention testing only	Intervention: **PC (Palliative care).**<br><br>PC nurses provided important continuity of care for NICU infants clinically requiring PC and at least weekly verbal support of parents. The PC service also coordinated family conferences, provided or requested orders to improve infant symptom management and comfort, and addressed parental coping and self-care.	***Overall satisfaction with care received***	During babies' admission (once)<br><br>- At discharge (or study closure for infants who remained hospitalised)<br><br>No pre-intervention parent satisfaction data available for comparison.	*Satisfaction questionnaire*<br><br>A researcher-created questionnaire based on extensive current literature review.<br><br>Validation: Partially reported. Authors stated content validity testing took place; no information on reliability testing provided.<br><br>1 question<br><br>Likert (1 extremely dissatisfied-4 to extremely satisfied).	**Parent satisfaction response numbers were small (n= 10), thus statistical comparison of parental satisfaction between cohorts was not possible.**<br><br>However, 100% of responding PC parents (n= 2) reported being "extremely satisfied'' with care, whereas only 50% of responding usual care parents (n= 4) reported extreme satisfaction.	**No**	**3**

Confidential: For Review Only

				Control: Usual clinical care for infants not requiring PC.			Optional free text (description of specific experiences impacting satisfaction with care)			
3. Stevens et al. (2011), USA	Mothers /147. For the OPBY NICU, 58 surveys were returned. For the SFR NICU, 89 were returned	Mean (SD)<br><br>Control: 35 (4)<br><br>Interv: 34 (3)<br><br>/ level not stated	Cohort trial. This research was part of a large prospective evaluation.<br><br>*Unit level effect:*<br>Two different time periods	Intervention: **SFR (Single-family room) NICU for neonatal care.**<br><br>Parents could visit their baby, room-in, do kangaroo care and breastfeed at any time, in individual rooms (containing bed, desk, closet, telephone, chair, refrigerator for breast-milk storage).<br><br>Control: OPBY (Open-bay) NICU. The traditional open-bay NICU was typical of facilities built before 1980. All neonates, family members, staff, monitors, and equipment were visible for all neonates in each room. Portable partitions were placed around the incubator for breastfeeding and kangaroo care.	***Parent satisfaction with different elements of NICU:***<br>*- Delivery*<br>*- Environment*<br>*- Nurses*<br>*- Physicians*<br>*- Discharge*<br>*- Personal*<br>*- Overall Assessment*	After babies were discharged (once)<br><br>- Mailed within 60 days of discharge of parents' infants from the NICU<br><br>No pre-intervention parent satisfaction data available for comparison (different parent groups pre and post intervention).	*Satisfaction questionnaire*<br><br>A questionnaire from Press Ganey Associates was used. Also included were three questions added by the investigators.<br><br>Validation: Partially reported. The original questionnaire was validated questionnaire but no content validity or reliability testing was reported regarding the 3 questions added by the study team.<br><br>42 questions in total (7 categories):<br>Delivery, Environment, Nurses, Physicians, Discharge, Personal,<br>Overall Assessment.<br><br>Likert (1 very poor-5 very good).	**Statistically significant improvement was found for the survey categories of Environment, Overall and the Total survey.**<br><br>Estimated numbers from report's figures as numbers not provided):<br><br>**Median SFR OPBY p-value**<br>Environment 4.7 3.7 <0.001<br>Overall 5 4.8 0.018<br>Total 4.7 4.5 0.045<br><br>16 items composite score for family-centered care:<br>4.4 4.0 0.017	**Yes**<br><br>Former NICU parents were involved in all phases of planning for the new SFR NICU.	**1**

Confidential: For Review Only

"Other" Non-Randomised controlled trials (Non-RCT) by publication year

Author (Date), Country	Parents' gender/ sample Size	Infant Gestation age (GA) in weeks /NICU level	Study design	Intervention	Outcome measures	Timing of measurement	Method of measurement	Results	Parent co-design?	Improved parent satisfaction?
1. Kadivar et al. (2017), Iran	Mothers /68	<=30 – 36<br><br>/ level not stated	Non-randomised, Convenience sampling.<br><br>*Group level effect:* Intervention/ control groups.<br><br>Pre and post-intervention testing.	Intervention: **Internet-based education.**<br><br>Mothers used an educational website set up by the research team (files and clips). Mothers could visit the website from 5:00-6:00 pm for 10 days. They were also allowed to use the website outside of the above hours and to report the duration of using the website to the researcher. Mothers had to use the website at least 3 times during 10 days, each time for at least 30 min.<br><br>Control: Mothers in the control group received the routine education provided in the NICU.	***Maternal satisfaction***	During babies' admission (twice)<br><br>- Day 1 of intervention<br><br>- Day 10 of intervention	*Satisfaction questionnaire (Validated)*<br><br>The *"What Being The Parent of a Baby is Like-Revised" Questionnaire (WBPL- Revised)* was used. The original English version by Pridham and Chang was translated to Persian.<br><br>11 questions<br><br>Total satisfaction score range (11–99)	There was a significant difference in the mean score of satisfaction between cases and controls while the mean score of satisfaction increased in both groups.<br><br>**Comparison of the mean score between the two groups showed that the level of satisfaction was significantly higher in the case group versus the control group.**<br><br>**Interv Control**<br>**before intervention**<br>**Mean (SD)** 81.62(13.50) 85.71(9.46)<br>**p-value** 0.993<br><br>**after intervention**<br>**Mean (SD)** 93.88 (5.38) 90.12 (7.78)<br><br>**p-value** 0.024	**No**	**1**

2. Kadivar et al. (2017), Iran	Mothers /70	Mean (SD)<br><br>Control 31.6 (2.4)<br><br>Interv: 32.9 (3.1)<br><br>/ level not stated	Non-randomised, Convenience sampling.<br><br>*Unit level effect*:<br>Two different time periods	Intervention: **Narrative writing.**<br><br>Mothers did narrative writing at least 3 times until the 10th day of admission.<br><br>Control: Mothers in the control group received the routine NICU treatment and care.	***Mothers' satisfaction with medical care provided by physicians, medical students, and nurses during neonatal admission to the NICU***	During babies' admission (twice)<br><br>- Day 3 of intervention<br><br>- Day 10 of intervention	*Satisfaction questionnaire (Validated)*<br><br>The *NIPS questionnaire* by Mitchell et al was used and translated to Persian.<br><br>24 questions (Likert scale)<br><br>Likert (1 always or not satisfied-7 never or completely satisfied). A higher score indicates more satisfaction.	**The satisfaction level of the mothers in the intervention group increased significantly during the study.**<br><br>The results of independent t test showed a significant difference in the satisfaction changes of the mothers on the 3rd and 10th day of NICU admission between intervention and control groups, indicating the effectiveness of narrative writing.<br><br>The results of paired t-test also showed a significant difference in the mean satisfaction level of the mothers between the 3rd and the 10th day in the intervention group.<br><br>**Interv** **Control**<br>**After intervention**<br><br>**Mean (SD)** 137 (15.2) 102.3 (25.6)<br><br>**p-value** 0.001	**No**	**1**
3. Garingo et al. (2016), USA	Not stated /9	23-39 / level III	Non-randomised, Convenience sampling.<br><br>*Group level effect*:<br>Intervention/ control groups<br><br>Post-intervention group testing only	Intervention: **Tele-rounding.**<br><br>Infants of intervention parents were cared for by an OFFSN (off site neonatologist) who was present via a remote-controlled robot. The OFFSN assessed infants via the robot's integrated stethoscope, with assistance from the nursing staff. During routine hours the OFFSN was called to discuss any issues with the patient. Emergencies/out of hours were covered by an ONSN (on site neonatologist).<br><br>Control: Infants of control parents received ONSN care. The attending neonatologist made daily patient rounds with the NICU team. After patient rounds, the NICU staff, under the supervision of	***Satisfaction with telemedicine***	During babies' admission (once)<br><br>- At the time of discharge<br><br>No pre-intervention parent satisfaction data available for comparison.	*Satisfaction questionnaire*<br><br>Validation: No content validity or reliability testing reported.<br><br>Number of questions: not stated.<br><br>Likert (1 excellent-5 very poor).	**Only the intervention group was assessed and only post-intervention.**<br><br>The authors reported that the parents surveyed were *"satisfied with their experience. 100% responded that they felt comfortable talking to the OFFSN on the mobile robot and would allow their infant or themselves to be cared for by a physician via telemedicine in the future."*	**No**	**4**

Confidential: For Review Only

				the attending neonatologist implemented the care plan.						
4. Globus et al. (2016), Israel	Mothers and fathers /Total surveys returned: 178	~40% in each group <32 / level III	Non-randomised, Convenience sampling.<br><br>*Unit level effect:*<br>Two different time periods	Intervention: **SMSi- Short Message Services Implementation.**<br><br>Parents were updated daily regarding the health status of their infant via SMS (short-message-services) from the Electronic Patient Record. All SMS messages were sent at 09:00am, including one-sentence sections with updated information (e.g. location of the infant's crib and current weight). Information regarding acute events/deterioration of the infant's medical condition was not included in the SMS, but was delivered personally to the parents in real time.<br><br>Control: Routine care pre-SMS implementation.	***1. Parent satisfaction related to parent communication with the medical staff***<br><br>***2. Overall parent satisfaction with treatment and staff attitudes throughout hospitalisation.***	During babies' admission (once)<br><br>- pre-SMS cohort and post-SMS cohort<br><br>No pre-intervention parent satisfaction data available for comparison (different parent groups pre and post intervention).	*Satisfaction questionnaire*<br><br>The *"Parents' attitudes regarding their experience during their infants' hospitalisation in the NICU"* questionnaire was used, as well as selected items from a literature review of similar questionnaires, including that by York Hospital and by Conner and Nelson.<br><br>Validation: No content validity or reliability testing reported.<br><br>Selected items related to four aspects of the NICU experience. 2 out of 4 directly assessed parent satisfaction:<br><br>1. Parental assessment of their communication with the medical staff.<br><br>Likert scale (1 do not agree at all-5 strongly agree)<br><br>2. Overall satisfaction with treatment and staff attitudes throughout hospitalisation.<br><br>Visual analog scale (scores range 0-10). Higher scores reflect greater satisfaction.	Overall, in both periods, parents expressed a high degree of satisfaction regarding the medical treatment, the information given and the communication with the medical staff. Overall satisfaction with treatment and with staff attitudes throughout hospitalisation was slightly greater in the post-SMS cohort but did not reach statistical significance.<br><br>**In the post-SMS cohort, a statistically significant improvement was noted regarding physician availability and patience, parental feelings of comfort in approaching the physicians and nurses, and regularly receiving information regarding the infants' medical status from the physicians.**<br><br>**Post SMS** **Pre SMS**<br>**Mean (SD)** 4.1 (1.0) 3.7 (1.3)<br>**p-value** 0.03<br>*Specific question: "I was pleased with the frequency with which I received information regarding my infant".*<br><br>Although improvement in all other categories was documented, it did not reach statistical significance.	**No**	**1**

Confidential: For Review Only

5.Kazemian et al. (2016), Iran	Mothers /220 newborns (assumed 220 mothers)	>37 / level not stated	Non-randomised, Convenience sampling.<br><br>*Group level effect:* Intervention/ control groups<br><br>Post-intervention testing only	Intervention: **Rooming-in care.**<br><br>Mothers and babies were admitted to a different atmosphere to the routine care. This facilitated the mothers and neonates with separate beds along with phototherapy devices and nursing clinical supervision.<br><br>Control: The routine care practiced in this neonatal unit supported partial stay of mothers beside their neonates, while sitting on chairs; however, most of the time the mother-infant dyad was separated.	***Maternal satisfaction with the neonatal care services and hospital stay comfort***	During babies' admission (once)<br><br>-Not stated exactly when<br><br>No pre-intervention parent satisfaction data available for comparison.	*Satisfaction questionnaire*<br><br>Validation: No content validity or reliability testing reported.<br><br>The authors state, *"a validated self-made questionnaire was employed, which was filled in by some trained midwives."* No further information on validation processes, number of questions or name of the questionnaire was provided.<br><br>Likert (5 very satisfied-1 dissatisfied).	**The level of satisfaction was significantly higher in the intervention group, compared to that in the control group.**<br><br>**Interv** **Control**<br>**Satisfaction %** 26.6 18.8<br><br>**p-value** 0.027	**No**	**1**
6. Van de Vijver and Evans (2015), UK	Not stated /105	Not stated / not stated	Non-randomised, Convenience sampling.<br><br>*Unit level effect:* Three different time periods	Intervention: **Baby diary.**<br><br>Each parent received a communication diary on their infant's admission to the unit. Staff wrote-in infant status updates and kept an infant interaction log with parents. Parents wrote in memories and questions for staff to address during face-to-face communication.<br><br>Control: Routine care, before implementation of the diaries.	***Satisfaction with communication from neonatal staff***	During babies' admission (three times)<br><br>- On the day of babies' discharge at study baseline<br><br>- On the day of babies' discharge at 1 month<br><br>On the day of babies' discharge at 15 months	*Satisfaction questionnaire*<br><br>The study team designed a questionnaire, based on the Department of Health and the National Institute for Health and Care Excellence (NICE) quality standards for specialist neonatal care.<br><br>Validation: No content validity or reliability testing reported.<br><br>5 questions ("yes or no")	**Small numbers. No data indicating statistical analysis conducted or evidence of statistically significant results.**<br><br>*"I was receiving regular communication from staff"*<br>94% - 1 month post diary cohort<br>93% - 15 months post diary cohort<br>77% - pre diary cohort<br><br>*"My questions and concerns were being addressed"*<br>100% - 1 month post diary cohort<br>93% - 15 months post diary cohort<br>91% - pre diary cohort<br><br>"I feel more involved in my baby's care"<br>92% - 1 month post diary cohort<br>100% - 15 months post diary cohort<br>88% - pre diary cohort	**Yes.**<br><br>The intervention concept was created by the project leaders following analysis of baseline survey results and used after multi-disciplinary input and discussion with staff and parents.	**3**
7. Voos and Park. (2014), USA	Not stated / 62	Not stated / level III	Non-randomised, Convenience sampling.<br><br>*Unit level effect:* Two different time	Intervention: **OU (Open Unit) policy.**<br><br>Parents were allowed access to their baby 24 hours a day, 7 days a week.<br><br>Control: Parents pre-OU	***Parent satisfaction with how much time parents get to spend with their baby***	After babies were discharged (once)<br><br>- After pre-OU parents were discharged<br><br>- After post-OU parents were	*Single question (From a validated questionnaire)*<br><br>The question *"Did you get to spend as much time as you wanted with your baby?"* was used from the NRC (National Research Corporation) Picker parent	**Small numbers. No data indicating statistical analysis conducted or evidence of statistically significant results.**<br><br>*"Did you get to spend as much time as you wanted with your baby?" Yes.*	**Yes.**<br><br>The NICU has a Family-centered care committee including	**3**

https://mc.manuscriptcentral.com/bmjpo

Confidential: For Review Only

			periods	implementation received routine care. The unit was closed to parents during nurse change of shift in mornings and evenings.		discharged	survey.<br><br>1 question ("yes or no")	Pre OU 78% (18/23)<br>Post OU 92% (36/39)	parents, which conducted this project.	
8. Segre et al. (2013), USA	Mothers /23	Mean (SD) 31.57 (5.30) / level III	For the outcome of parent satisfaction:<br><br>Non-Randomised, Convenience sampling.<br><br>*Group level effect:* Intervention/ control groups<br><br>Post-intervention group testing only	<u>Intervention:</u> **(LV) Listening visits.**<br><br>Mothers met with the LV provider for up to six 50-min LV sessions, conducted in a private hospital, every 2–3 days, within 1-month. Visits entailed greeting, debriefing, updating on current issues, working an agenda through listening and problem solving, and providing closure through summary.<br><br><u>Control:</u> Women who did not meet the specific criteria (e.g. minimum score on depression scale) were not invited to join the treatment trial and received routine NICU care/support instead.	***Satisfaction with the treatment and the outcome.***	During babies' admission (once)<br><br>- Not stated exactly when<br><br>No pre-intervention parent satisfaction data available for comparison.	*Satisfaction questionnaire*<br><br>The *Client Satisfaction Questionnaire* was used.<br><br><u>Validation:</u> Partially reported. Authors stated reliability testing took place; no information on content validity provided.<br><br>8 questions.<br><br><u>Format of questions</u>: not stated	**Only the intervention group was assessed and only post-intervention.**<br><br>The authors reported:<br><br>*"The majority of women who received LVs were highly satisfied with the intervention".*<br><br>*"The average score for the Client Satisfaction Questionnaire was 29.91, comparable to levels of satisfaction reported by clients receiving depression treatment from a mental health professional."*<br><br>*"91.3% of our participants rated the quality of help they received as excellent."*	**No**	**4**
9. Palma et al. (2012), USA	Not stated / 26 families returned the survey containing the satisf. measure)	Not stated / level II	Non-randomised, Convenience sampling.<br><br>*Unit level effect:* Two different time periods	<u>Intervention:</u> **YBDU (Your Baby's Daily Update). A daily parent update letter generated from the Electronic Medical Record (EMR).**<br><br>Parents were given daily YBDU reports, printed automatically from the EMR. The YBDU included information about an infant's status during the past 24 hours and a hand-written update by the infant's care provider.<br><br><u>Control:</u> Parents received routine care and usual verbal updates (6 months pre- adoption of YBDU).	***Satisfaction with YBDU***	During babies' admission (once)<br><br>- Not stated exactly when<br><br>No pre-intervention parent satisfaction data available for comparison (different parent groups pre and post intervention).	*Satisfaction questionnaire*<br><br>A questionnaire including items regarding adoption of and satisfaction with YBDU was used.<br><br><u>Validation:</u> No content validity or reliability testing reported.<br><br><u>Number and format of questions:</u> not stated.	**Only the intervention group was assessed and only post-intervention.**<br><br>The authors reported:<br>*"When asked to rate the statement "I like receiving Your Baby's Daily Update", 96% of families who used YBDU as an information source responded with the highest rating, "always"."*	**No**	**4**
10. Voos et al.	Not stated /28	Not stated / level not	Non-randomised,	<u>Intervention:</u> **Family-centered rounds**	***Global satisfaction with***	During babies' admission (twice)	*Satisfaction questionnaire (Validated)*	**A subset of NIPS items related to communication**	**No**	**1**

Confidential: For Review Only

(2011), USA		stated	Convenience sampling.<br><br>*Unit level effect:*<br>Two different time periods	**(FCRs).**<br><br>Parents were invited to attend rounds and choose their level of involvement (attend every day/not at all/periodically). For confidentiality concerns, parents were asked to step outside while rounds of others' infants took place. The staff augmented FCRs by meeting with parents again after rounds if needed.<br><br><u>Control:</u> Parents received routine care. Prior to FCR implementation parents were asked to leave the unit during rounds.	***the NICU experience***	- Prior to FCR<br><br>- 6 months after starting FCR	*The NIPS* questionnaire.<br><br>24 questions: looking at satisfaction in different areas of the NICU (medical caregivers, communication, tests, and procedures).<br><br>Likert scale (1-7 points).	**(i.e. being kept informed as to changes in the infant's condition, meeting with physicians, and information about long-term expectations) yielded a significant increase from pre to post FCR scores.**<br><br>**post-FCR pre-FCR p-value**<br>**NIPS score** 5.5 4.4 <0.01<br><br>The average score on the NIPS did not change significantly.		
11. Weiss et al. (2010), USA	Mothers /84	Mean (SD)<br><br>Pre-interv group: 32 (4.4)<br><br>Post-interv group: 32 (9)<br><br>/ level III	Non-randomised, Convenience sampling<br><br>*Unit level effect:*<br>Two different time periods	<u>Intervention:</u><br>**An intervention to increase PMP (Principal Medical Providers) availability and communication frequency.**<br><br>(1) A brief education module for PMPs was introduced (2) parents received a contact card with PMP names, job descriptions and contact information (3) a poster of the faces, names and titles of the PMPs was placed at NICU entrance.<br><br><u>Control:</u> Parents received routine care in the pre-intervention cohort, without the above.	***Parent satisfaction with physician and nurse practitioner communication***	During babies' admission (twice)<br><br>- Pre-intervention<br><br>- Post-intervention	*Satisfaction Questionnaire (Validated)*<br><br>A pilot survey written by Press Ganey and the Picker Institute was used and revised based on parent responses.<br><br>6 open-ended questions (Quantity of communication)<br><br>6 Likert scale questions (range questions (Availability, understanding, reciprocity, empathy, overall satisfaction)	**Overall satisfaction, based on the ordinal analysis of the five-point Likert scale, was significantly higher after the intervention (P<0.01).**<br><br>**Overall satisfaction, dichotomised into a satisfied subgroup and a dissatisfied subgroup for each cohort, was also significantly increased after the intervention.**<br><br>**post –interv pre-interv**<br>**Very satisfied/ Somewhat satisfied** 97%(32/33) 74%(37/50)<br><br>**p-value** <0.01	**No**<br><br>Authors stated that only after trialing the intervention on many parents (both satisfied and unsatisfied) gave suggestions to improve it.	**1**
12. Foster et al. (2008), Australia	Mothers and fathers /93<br><br>5 Special Care Nurseries	Mean (SD)<br><br>Headbox: 36.5 (2.6)<br><br>CPAP: 36 (3)<br><br>/level I	Non-randomised, Convenience sampling<br><br>*Group level effect:*<br>Intervention 1/ intervention 2 groups	<u>Intervention 1:</u> **Infants received headbox oxygen treatment for respiratory distress.**<br><br><u>Intervention 2:</u> **Infants received continuous oxygen positive airway pressure (CPAP) treatment for respiratory distress.**	***Satisfaction with treatment (i.e. headbox oxygen or CPAP)***	During babies' admission (once)<br><br>- Within 5 days of the babies' admission<br><br>No pre-intervention parent satisfaction data available for comparison.	*Single question*<br><br><u>Validation:</u> No content validity or reliability testing reported.<br><br>1 likert scale question (1 not at all satisfied-5 extremely satisfied).	**Parents with babies receiving CPAP rated their satisfaction with the baby's treatment statistically significantly higher than the headbox group mean rating.**<br>**Headbox CPAP**<br>**Mean (SD)** 3.71 (1.31) 4.51 (0.79)<br>**p-value** 0.001	**No**	**1**

Confidential: For Review Only

			Post intervention testing only					The CPAP group averaged between *very and extremely satisfied* compared with parents of babies receiving headbox, who averaged between *satisfied and very satisfied ratings.*		
13. Byers et al. (2006), USA	Only mothers reported /35	Preterm infants<br><br>Mean (SD)<br><br>Control: 28.9 (3.44)<br><br>Interv: 28.6 (3.37)<br><br>/ level II/III	For the outcome of parent satisfaction:<br><br>Non-randomised, Convenience sampling<br><br>*Group level effect:* Intervention/ control groups<br><br>Post-intervention testing only	Intervention: **Infants received individualised, developmentally supportive family-centered care.**<br><br>Infants received care within the framework and philosophy of individualised, developmentally supportive family-centered interventions.<br><br>Control: Infants received the traditional NICU standard of care.	***Parent satisfaction relating to:***<br>*- parental perceptions of staff caring*<br>*- education received*<br>*- preparation for the parental role*<br>*- overall satisfaction with the NICU experience*	During babies' admission (once)<br><br>- On the day before discharge<br><br>No pre-intervention parent satisfaction data available for comparison.	*Satisfaction questionnaire*<br><br>The *NICU's parental satisfaction tool* was used.<br><br>Validation: Partially reported. Authors stated content validity testing took place, but "because of the disparate nature of the items, survey reliability was not assessed".<br><br>11 questions<br><br>Likert scale (1-5 strongly agree)	**Independent t-test analysis of parent satisfaction/perception scores showed no significant difference between groups.**<br><br>Example statement: "*I was satisfied with the car my baby and I received in the NICU"*<br><br>**Interv** / **Control**<br>**Mean (SD)** 4.94(0.23) / 4.71(0.47)<br>**p-value** 0.064<br><br>Both groups reported very high satisfaction with their NICU experience (4.4-5.0)	**No**	**2**
14. Mills et al. (2006), USA	Not stated/ not stated<br><br>Parents of infants from 6 hospitals	Not stated / level not stated	Implementation project<br><br>Plan Do Study Act (PDSA) quality improvement testing	Intervention: **5 potentially better practices (PBPs) in the area of discharge planning.**<br><br>The project team iteratively implemented 5 PBPs:<br>1. Created an easy-to-use, easy-to-access discharge planning tool kit.<br>2. Restructured communication tools and processes to reflect a "plan for the day, the stay, and the way" to discharge.<br>3. Maximised the impact and use of caregiver educational tools, and updated materials and delivery systems for caregiver education.<br>4. Used various continuous quality improvement tools and processes to ensure parent/caregiver and staff satisfaction.<br>5. Analysed and enhanced interactions with and transfers into	***General satisfaction***<br>*- with care*<br>*- parents' feelings about preparedness for discharge*<br>*- ability and confidence in feeding*<br>*- familiarity with their infant*<br>*- feeling like a parent*<br>*- participation in care*<br>*- adequacy of information from staff about medical and care issues*	During babies' admission (4 times)<br><br>- Not reported exactly when	*Satisfaction questionnaire*<br><br>The Internet-based parent satisfaction survey *"howsyourbaby.com"* that was developed especially for this NICU population was used.<br><br>Validation: No content validity or reliability testing reported.<br><br>Number and format of questions: not stated.	Through multiple rapid-cycle projects, the project's collaborative group made changes within the 5 PBP plans.<br><br>**Parent satisfaction measures were used to longitudinally monitor the changes made, rather than make direct group comparison. No data indicating statistical analysis conducted or evidence of statistically significant results.**<br><br>Parent satisfaction survey results (all centers combined) were high across 4 measurement quartiles. No specific interquartile analysis was reported.<br><br>Parent readiness for discharge was high at the beginning and throughout the collaborative. Parents' receiving *"just the right amount of information"* regarding car seat trials and safe sleep demonstrated some variability throughout the collaborative.	**No**	**3**

Confidential: For Review Only

				the community.<br><br><u>Control:</u> N/A. No discrete control group. PDSA quality improvement methodology was applied to parent participants.						
15. Wielenga et al. (2006), The Netherlands	Mothers and fathers / 46	Mean (SD)<br><br>Control: 28.5 (26.0–29.9)<br><br>Interv: 28.3 (25.6-29.9)<br><br>/ level III	Non-randomised, Convenience sampling<br><br>*Unit level effect:* Two different time periods	<u>Intervention:</u> **The Newborn Individualised Developmental Care and Assessment Program (NIDCAP).**<br><br>Infants received care according to NIDCAP principles and parents were taught how to provide it. Caregiving plans were designed based on the infant's current developmental stage, medical condition and family needs. Caregivers learnt to watch sensitively and note the infant's reactions to different types of handling and care, making continuous adjustments.<br><br><u>Control:</u> Infants received traditional neonatal care practiced at that time.	***Parent satisfaction relating to:***<br>*-Overall rating*<br>*-Care of the baby*<br>*-Communication with staff*<br>*-Involvement in care -Being prepared -Support*<br>*-Being a parent*<br>*-Being near your baby -Total score*	After babies were discharged (on day of discharge/ transfer)<br><br>- Pre NIDCAP cohort<br><br>- Post NIDCAP cohort	*Satisfaction questionnaire (Validated)*<br><br>The *NICU-PSF* was used and translated from English to Dutch.<br><br>62 questions<br><br>Closed and open-ended questions.<br><br>Different rating scales used (5-point rating scale from "extremely satisfied" to "not at all satisfied" or "excellent" to "poor").<br><br>Total score range (50-243 points)	**The intervention group's mean total score was significantly higher than the control.**<br><br>**Interv Control**<br>**Mean (SD)**<br>185.67(17.74) 174.04(20.98)<br><br>**p-value** 0.041<br><br>Almost all separate concepts showed an increase in their mean scores. The concept of "*being a parent"* had a slightly lower mean score (9.39, SD = 1.73) in the intervention group than in the control group (9.78, SD = 2.09).<br><br>**The concept of *"preparedness"* showed statistically significant difference**:<br><br>**Interv Control**<br>**Mean** 16.38 13.83<br>**p-value** 0.038	**No**	**1**
16. Penticuff and Arheart. (2005), USA	Dyads (both parents or mother with her support person)/ 122 mothers<br><br>Results based only on mothers' data.	Not stated / Level III	A repeated measures design<br><br>- First 2 years (control group data collection)<br><br>- Year 3 (staff training)<br><br>- Year 4 (implementing the intervention)<br><br>- Year 5 (collecting data from the intervention group)<br><br>*Unit level*	<u>Intervention:</u> **The Newborn Individualised IPC- CPM intervention (Infant Progress Chart) - (Care Planning Meetings).**<br><br>Both the mother and father (or the mother and her designated support person) were shown how to use the Infant Progress Chart and attended 3 Care Planning Meetings (with neonatologists/Neonatal Nurse Practitioners).<br><br><u>Control:</u> During the control phase, professionals carried out usual communication and interaction with control	***Satisfaction with participation in decision making*** **was measured by 5 collaboration indices:**<br><br>Satisfaction with (1) Care<br>(2) Relationships with professionals<br>(3) Decision input<br>(4) The process of decision making<br>(5) Decisions made	During babies' admission (three times)<br><br>- Within 0–3 days<br><br>- 9– 12 days<br><br>- 25–28 days of an infant's admission to the NICU	*Three satisfaction questionnaires*<br><br>1. Two subscales of the investigator-designed *"Parents' Understanding of Infant Care and Outcomes Questionnaire"* were used to measure Satisfaction with Care (1).<br><br><u>Validation:</u> Partially reported. Authors stated content validity testing took place; no information on reliability testing provided.<br><br>30 questions.<br><br>Five-point Likert scale.<br><br>2. A subscale of the investigator-designed	**The intervention group was more satisfied with the amount of decision input they had (3) and with the process by which medical decisions were made (4).**<br><br>**Interv Control p-value**<br>Decision input amount (3)<br>**Mean** 33.44 30.05 0.058<br><br>Process of decision making (4)<br>**Mean** 120.20 104.95 0.012<br><br>There were no statistically significant differences between control and intervention groups in satisfaction with their infants' care (1), with relationships with NICU professionals (2) and with the decisions made for infant treatment (5).	**No**	1

Confidential: For Review Only

			effect: Two different time periods	group parents.			*"Relationships with Professional and Decision Input Questionnaire"* was used to measure Satisfaction with relationships (2).<br><br>Validation: Partially reported. Authors stated content validity testing took place; no information on reliability testing provided.<br>12 questions.<br><br>Five-point Likert scale<br><br>3. Validated.<br>The *"Collaboration and Satisfaction About Care Questionnaire"* developed by Baggs, was used to measure Satisfaction with decision input (3), with decision process (4) and with decisions made (5).<br><br>9 questions.<br><br>7-point scale, (1 strongly disagree -7 strongly agree)			
17. Byers et al. (2003), USA	Mothers/ 19	Mean (SD)<br><br>Control: 29 (2.00)<br><br>Interv: 28.9 (2.42)<br><br>/ level II-III	For the outcome of parent satisfaction:<br><br>Non-randomised, Convenience sampling<br><br>*Group level effect:* Intervention/ control groups<br><br>Pre and post-intervention testing	Intervention: **Co-bedding premature multiple-gestation infants in incubators.**<br><br>Infants were nursed in the same incubator using a co-bedding protocol (e.g. recording all of the care provided to one infant before providing care to the second infant)<br><br>Control: Single-bedding premature multiple-gestation infants in incubators.	***Parent satisfaction related to:***<br>*- staff concern*<br>*- support of family*<br>*- staff explanations*<br>*- infant environment,*<br>*- comfort with feeding*<br>*- kangaroo care encouragement*<br>*- staff explanation of signs of infant stress*<br>*- visiting schedule*<br>*- overall satisfaction with the NICU experience*	During babies' admission (twice)<br><br>- At baseline<br><br>- 5 days later	*Satisfaction questionnaire*<br><br>The NICU's standard parental satisfaction tool was used.<br><br>Validation: Partially reported. Authors stated content validity testing took place, but because of the disparate nature of the items, survey reliability could not be assessed.<br><br>11 questions.<br><br>5-point Likert-type scale.	**The only significant difference for a post-intervention item was a higher score for the item "Attempts were made to create a quiet environment for my baby."**<br><br>**Interv Control p-value**<br>**Mean** 4.80 3.89 0.033<br><br>Independent t-tests comparing the co-bedded and control group parental scores found no significant differences in their parental satisfaction scores, except for higher baseline parental satisfaction scores (p=0.029) in the co-bedded group.	**No**	**1**

Confidential: For Review Only

18. Polizzi et al. (2003), USA	Mothers and fathers/ 33	Mean (SD)<br><br>Control: 32.97 (1.9)<br><br>Interv: 33.08 (1.31)<br><br>/ level III	A retrospective, comparative, descriptive design.<br><br>*Unit level effect*	Intervention: **Co-bedding multiple-gestation infants in the NICU.**<br><br>Multiple-gestation infants were nursed in the same incubator or crib. The intervention was evaluated retrospectively after implementation of a co-bedding practice protocol.<br><br>Control: Traditionally-bedded group (babies were routinely placed in separate incubators or cribs)	***Parental satisfaction as measured by 9 questions relating to parent perceptions and their baby's care***	After babies were discharged (once)<br><br>- All parents were mailed the survey. A second survey was sent to those who did not respond after 2 months<br><br>No pre-intervention parent satisfaction data available for comparison.	*Satisfaction questionnaire*<br><br>The *parental perception/ satisfaction tool* was used.<br><br>Validation: Partially reported. Authors stated content validity testing took place; no information on reliability testing provided. 6/9 questions were from a similar tool that was validated by the Vermont Oxford NICU Quality Improvement Initiative.<br><br>9 questions (such as *"I was satisfied with the care my babies received in the hospital")*.<br><br>Likert (1 strongly disagree- 5 strongly agree)	Mothers reported overall satisfaction with the NICU care and staff, as well as adequacy of their ability to care for their infants after discharge, with scores ranging from 4.19 to 4.71.<br><br>**The only survey item score that was significantly different between groups was for the item *"I was encouraged by the hospital staff to bond with my babies."***<br><br>**Interv Control p-value**<br>**Mean** 4.71 4.36 0.049	**No**	**1**
19. Legault and Goulet. (1995), Canada	Mothers/ 61 completed both tests	Mean (range)<br><br>30 (24-35)<br><br>/ level II	Time-series design<br><br>*Group level effect:* Same group exposed to both methods with post-method testing only.	Intervention: **Kangaroo method of removing an infant from an incubator.**<br><br>Mothers were taught the "kangaroo method" (skin-to-skin contact): infant wears a diaper/head cap and is placed in a vertical position on the parent's bared chest. A blanket covers the infant and the parent's clothing is fastened around the infant. The parent sits in a rocking chair, inclined so that the infant's head is at 60'.<br><br>Control: Traditional method. Newborns wearing a diaper and a head cap, are wrapped in a blanket and placed in their parent's arms.	***Mothers' satisfaction with:***<br>*- Each method of removing an infant from incubator*<br>*- Her feelings after each method*	During babies' admission (twice)<br><br>- After the intervention<br><br>- After the control method<br><br>No pre-intervention parent satisfaction data available for comparison.	*Satisfaction questionnaire*<br><br>The *"Maternal Satisfaction Questionnaire"* was used. It was developed by integrating components described by Affonso et al and the clinical experience of the investigators.<br><br>Validation: Partially reported. Authors stated content validity testing took place; no information on reliability testing provided.<br><br>15 questions<br><br>Likert (1 very much-5 don't know)<br><br>An open-ended question invited the mother to list and explain anything else related to her experience.	**Regardless of the method tested, mothers expressed high levels of satisfaction (it was the first time since giving birth that they could hold their infants).**<br><br>Three statements proved more powerful in discriminating between the methods:<br><br>**Rated higher after the kangaroo method test:**<br>- *"I like the contact with my baby's skin"*<br>(p=0.0001)<br><br>**Rated higher after the traditional method test:**<br>- *"I like to talk to and whisper to my baby" (p = 0.015)*<br>- *"I looked into my baby's eyes and stared at his/her face" (p=0.0001)*	**No**	**1**

Confidential: For Review Only
