# Supplementary material for: Interventions to improve quantitative measures of parent satisfaction in neonatal care: a systematic review
Source: BMJ Paediatr Open. 2020 Mar 15;4(1):e000613. doi: 10.1136/bmjpo-2019-000613 (PMC7073789; doi:10.1136/bmjpo-2019-000613)
Supplement: Reviewer comments [file bmjpo-2019-000613.reviewer_comments.pdf]

PEER REVIEW HISTORY

BMJ Paediatrics Open publishes all reviews undertaken for accepted manuscripts. Reviewers are asked to complete a checklist review form and are provided with free text boxes to elaborate on their assessment. These free text comments are reproduced below.

There is no peer review to display for this article.
